# Elevated Levels of Arachidonic Acid-Derived Lipids Including Prostaglandins and Endocannabinoids Are Present Throughout ABHD12 Knockout Brains: Novel Insights Into the Neurodegenerative Phenotype

**DOI:** 10.3389/fnmol.2019.00142

**Published:** 2019-05-31

**Authors:** Emma Leishman, Ken Mackie, Heather B. Bradshaw

**Affiliations:** ^1^Program in Neuroscience, Indiana University Bloomington, Bloomington, IN, United States; ^2^Department of Psychological and Brain Sciences, Indiana University Bloomington, Bloomington, IN, United States; ^3^Gill Center for Biomolecular Science, Indiana University Bloomington, Bloomington, IN, United States

**Keywords:** ABHD12, PHARC, lipidomics, arachidonic acid, endogenous cannabinoid, aging, neurodegeneration, mouse brain

## Abstract

Derived from arachidonic acid (AA), the endogenous cannabinoid (eCB) 2-arachidonoyl glycerol (2-AG) is a substrate for α/β hydrolase domain-12 (ABHD12). Loss-of-function mutations of ABHD12 are associated with the neurodegenerative disorder polyneuropathy, hearing loss, ataxia, retinitis pigmentosa, and cataract (PHARC). ABHD12 knockout (KO) mice show PHARC-like behaviors in older adulthood. Here, we test the hypothesis that ABHD12 deletion age-dependently regulates bioactive lipids in the CNS. Lipidomics analysis of the brainstem, cerebellum, cortex, hippocampus, hypothalamus, midbrain, striatum and thalamus from male young (3–4 months) and older (7 months) adult ABHD12 KO and age-matched wild-type (WT) mice was performed on over 80 lipids via HPLC/MS/MS, including eCBs, lipoamines, 2-acyl glycerols, free fatty acids, and prostaglandins (PGs). Aging and ABHD12 deletion drove widespread changes in the CNS lipidome; however, the effects of ABHD12 deletion were similar between old and young mice, meaning that many alterations in the lipidome precede PHARC-like symptoms. AA-derived lipids were particularly sensitive to ABHD12 deletion. 2-AG increased in the striatum, hippocampus, cerebellum, thalamus, midbrain, and brainstem, whereas the eCB *N*-arachidonoyl ethanolamine (AEA) increased in all 8 brain regions, along with at least 2-PGs. Aging also had a widespread effect on the lipidome and more age-related changes in bioactive lipids were found in ABHD12 KO mice than WT suggesting that ABHD12 deletion exacerbates the effects of age. The most robust effects of aging (independent of genotype) across the CNS were decreases in *N*-acyl GABAs and *N*-acyl glycines. In conclusion, levels of bioactive lipids are dynamic throughout adulthood and deleting ABHD12 disrupts the wider lipidome, modulating multiple AA-derived lipids with potential consequences for neuropathology.

## Introduction

Endogenous cannabinoids (eCBs) such as AEA (Devane et al., 1992) and 2-AG (Mechoulam et al., 1995; Sugiura et al., 1995) activate the cannabinoid receptors, CB_1_ and CB_2_ (Felder et al., 1995). Linked to wider lipid signaling, eCBs are derived from AA-containing membrane phospholipids like NAPEs and DAGs. AEA is synthesized through NAPE-PLD (Okamoto et al., 2004) and metabolized through FAAH (Cravatt et al., 2001). DAGL synthesizes 2-AG from its DAG precursor (Sugiura et al., 2006). 2-AG is metabolized to AA by MAGL, which is quantitatively important step in the production of AA into PGs through COX enzymes (Kozak et al., 2002; Nomura et al., 2011; Leishman et al., 2016a). 2-AG is also metabolized to AA to a lesser extent by ABHD6 and ABHD12, though their relationship with PG production is unknown (Blankman et al., 2007; Savinainen et al., 2012). These canonical pathways also regulate the structural analogs of AEA and 2-AG, called lipoamines and 2-acyl glycerols, respectively (Ueda et al., 2013). Previous data from our lab showed that deletion of FAAH had the most profound effect on AA-derived lipoamines, with no effect on PGs, whereas NAPE-PLD and MAGL deletion had differential effects on lipoamines, but more consistent effects on PG levels (Leishman et al., 2016a,b). Those data highlight the interconnectedness of the CNS lipidome as they relate to specific lipid-active enzymes. Our studies here will explore how this interconnectedness extends to ABHD12.

Expressed throughout the PNS and CNS, including in both resting and activated microglia (Zhang et al., 2014), the serine hydrolase ABHD12 is responsible for approximately 9% of total brain 2-AG hydrolysis (Blankman et al., 2007). It was later discovered that ABHD12 acts as a lysoPS lipase in the mouse brain (Blankman et al., 2013). ABHD12 KO mice had WT levels of 2-AG in the whole brain, but had a pro-inflammatory phenotype including increased microglial activation in the cerebellum (Blankman et al., 2013). LysoPS lipids that activate TLR2 and microglia were increased in ABHD12 KO mice in several brain regions, including the cerebellum, cortex, and hippocampus (Blankman et al., 2013). With emergent roles in immune regulation, including in the CNS, lysoPS lipids are structurally related to PS membrane phospholipids, but have 1 fewer acyl moiety compared to PS (Frasch and Bratton, 2012). Blankman et al. (2013) measured 12 targeted species of lysoPS in ABHD12 KO and WT mice, each containing a different fatty acid moiety. All of the lysoPS species measured were upregulated in ABHD12 KO, with the exception of 22:6 lysoPS (Blankman et al., 2013). PGs were not measured in these experiments (Blankman et al., 2013), although previous work has suggested that lysoPS signaling can stimulate PG production (Frasch and Bratton, 2012), leaving unanswered questions as to how ABHD12 activity might relate to PGs. In older adulthood, ABHD12 KO mice show deficits in hearing, motor coordination and vision, and have a blunted startle response (Blankman et al., 2013). However, the elevated levels of lysoPS lipids were detected in the brains of ABHD12 KO mice at 2–6 months, preceding the behavioral deficits (Blankman et al., 2013).

ABHD12 is implicated in a human genetic disease (Metzler, 2011). This disease, PHARC, is named for its symptoms: polyneuropathy, hearing loss, ataxia, retinitis pigmentosa, and cataracts (Fiskerstrand et al., 2010). PHARC is a rare, progressive disease with no current cure (Fiskerstrand et al., 2010). Symptoms begin to appear in teenage years and continue to worsen (Fiskerstrand et al., 2010). First, sensorimotor fibers demyelinate, and the cerebellum and retina begin to atrophy (Fiskerstrand et al., 2010). PHARC patients have plasma 2-AG levels and CB_1_ functioning equivalent to healthy controls (Fiskerstrand et al., 2010). Each one of the several mutations in ABHD12 reported to cause PHARC completely nullifies the function of ABHD12 (Chen D.H. et al., 2013). Thus, the ABHD12 KO mouse is a suitable model for PHARC (Blankman et al., 2013). Like in humans, PHARC-like symptoms do not appear until later in life in ABHD12 KO mice and get progressively worse with age (Blankman et al., 2013). ABHD12 is also present in zebrafish and is highly expressed in the brain (Oltrabella et al., 2017). In particular, ABHD12 is co-localized with myelin basic protein (Tingaud-Sequeira et al., 2017). In zebrafish, deletion of ABHD12 also caused a PHARC-like syndrome (Tingaud-Sequeira et al., 2017). Although neuroinflammation and demyelination play a role (Blankman et al., 2013; Tingaud-Sequeira et al., 2017), it is not fully understood how deficiencies in ABHD12 cause PHARC (Tingaud-Sequeira et al., 2017).

The expression of eCB proteins and ligands is remarkably dynamic throughout the life span (Lee et al., 2013; Feliszek et al., 2016). For example, levels of 2-AG were lower in the hippocampus of older adult mice than in the young adult (Piyanova et al., 2015). The decrease in 2-AG was associated with reduced DAGL mRNA and protein and reduced ABHD6 protein (Piyanova et al., 2015), although ABHD12 was not measured (Piyanova et al., 2015). MAGL mRNA, but not protein, decreased in the older adult hippocampus. However, MAGL enzymatic activity increased, highlighting how enzyme activity is not tightly coupled to levels of gene expression (Piyanova et al., 2015). Enzymes that synthesize and metabolize AEA also vary with age. For example, the expression and activity of NAPE-PLD, an enzyme critical for maintaining brain levels of AEA and other NAEs (Leishman et al., 2016a), increased with age in the rat brain, as did FAAH (Morishita et al., 2005). Activity of enzymes upstream of NAPE-PLD that generate NAE precursors might also decrease with age (Moesgaard et al., 2003). Given that eCB system enzymes also metabolize structurally similar bioactive lipids, it is likely that levels of these similar lipids are also dynamic throughout the lifespan (Leishman et al., 2016a,b).

Lipoamines, 2-acyl glycerols, PGs, and AA all have important roles in neurophysiology and neuropathology, and interact with microglia, which are involved in PHARC pathology (Blankman et al., 2013). To test the hypothesis that ABHD12 deletion widely affects bioactive lipids, levels of over 80 lipids were compared between ABHD12 KO and WT mice in 8 different brain areas. A regional approach was taken because, although the contribution of ABHD12 to whole brain 2-AG hydrolysis has been evaluated (Blankman et al., 2007), it is still unknown whether ABHD12 contributes to more localized 2-AG hydrolysis. To test the hypothesis that these disruptions in the lipidome are present before PHARC-like symptomology in ABHD12 KO mice, the effects of ABHD12 deletion on lipid levels in the 8 brain areas were compared between younger pre-symptomatic adult and older adult mice. A second level of analysis includes the effect of age on both the WT CNS lipidome and the interplay between age and ABHD12 deletion. Overall, there was an upregulation of multiple AA-derived lipids in younger and older adult mouse brain areas in ABHD12 KOs that appears to be exacerbated with age, suggesting a pro-inflammatory lipid endophenotype that may contribute to microglial activation and neurodegeneration.

## Materials and Methods

### Mice and Tissue Collection

All animal procedures were approved by the Bloomington Institutional Animal Care and Use Committee of Indiana University. Mice were all male and from the C57BL6/J background. 6 younger adult WT mice (3–4 months old) and 6 older adult WT mice (7 months old) were compared with age-matched ABHD12 KO mice (6 younger adult and 9 older adult ABHD12 KO mice). Mice from each age/genotype were obtained from at least two litters. The ABHD12 mice establishing the KO colony were generously provide by Dr. Ben Cravatt (Blankman et al., 2007) in 2011. This line was generated using C57BL/6 ES cells and have been backcrossed into C57BL/6J mice >10 times. Genetic vigor of our KO colony has been maintained by backcrossing KOs into C57BL/6J mice every ∼18 months. WT and KO mice were housed 2–4 animals per cage, with a 12 h light cycle (lights on from 0700 to 1900). Brains were harvested ∼6 h into the light cycle. Temperature was set at 23+/-2°C and humidity range was 30–70%. Cages were Allentown IVC cages (28×18×13 cm), contained within a ventilated rack and lined with corncob bedding (Teklad #7092). Mice were fed *ad libitum* Teklad 18% protein rodent diet (cat# 2918 or 2018). After mice were sacrificed via rapid decapitation, brains were immediately removed, flash-frozen in liquid nitrogen, and then stored at -80°C until dissections were performed. Brains were dissected on an ice-cold dissection plate into these 8 discrete regions: STR, HIPP, CER, THAL, CTX, HYP, MID, and STEM. These abbreviations for these brain areas will be used exclusively when discussing the results generated by these specific dissections. Each dissected area was immediately placed in liquid nitrogen and then stored at -80°C until used for lipid extraction.

### Lipid Extraction and High-Pressure Liquid Chromatography Coupled to Tandem Mass Spectrometry (HPLC/MS/MS)

Lipid extracts were performed on brain regions as previously described (Bradshaw et al., 2006; Stuart et al., 2013; Raboune et al., 2014; Leishman et al., 2016a,b, 2017, 2018). First, samples were flash-frozen in liquid nitrogen, weighed, and transferred to a centrifuge tube. The mass of the largest sample was multiplied by 50 and this many mL of HPLC-grade methanol (Thermo Fisher Scientific, Fair Lawn, NJ, United States) was added to the tube. Tubes were spiked with 500 picomoles d_8_AEA (Cayman Chemical, Ann Arbor, MI, United States). After sitting on ice in darkness for 2 h, samples were individually homogenized and centrifuged at 19,000*g* for 20 min at 20°C. The supernatants were decanted and diluted with 3 volumes of HPLC water (Fisher). Lipids were partially purified on C-18 solid phase extraction columns (Agilent, Palo Alto, CA, United States). A series of 4 elutions with 1.5 mL of 60, 75, 85, and 100% methanol were collected for analysis (Stuart et al., 2013; Leishman et al., 2016a,b, 2018).

Following published protocols (Bradshaw et al., 2006; Tan et al., 2006; Smoum et al., 2010; Stuart et al., 2013; Tortoriello et al., 2013; Raboune et al., 2014; Leishman et al., 2016a,b, 2017, 2018); samples were analyzed using an Applied Biosystems API 3000 triple quadrupole mass spectrometer with electrospray ionization (Foster City, CA, United States). Using an Agilent XDB-C18 reversed phase analytical column and optimized mobile phase gradients, 20 μL from each elution were chromatographed. Mobile phase A: 20% methanol, 80% water (v/v) and 1 mM ammonium acetate (Sigma, St. Louis, MO, United States). Mobile phase B: 100% methanol, 1 mM ammonium acetate. Two Shimadzu 10ADvp pumps (Columbia, MD, United States) provided the pressure for gradient elution. Every method run began with 0% mobile phase B, reached 100% mobile phase B flowing at 0.2 mL/min, and gradually returned to 0% mobile phase B.

### Data Analysis and Statistical Procedures

Multiple reactions monitoring HPLC/MS/MS methods tailored for groups of structurally similar compounds were used to detect the ∼80 lipids in the screening library (Supplementary Figure 1). This screening library will be referred to as the lipidome when discussing results from this study. HPLC/MS/MS data were analyzed using Analyst software (Applied Biosystems) (Bradshaw et al., 2006; Tan et al., 2006; Stuart et al., 2013; Tortoriello et al., 2013; Raboune et al., 2014; Leishman et al., 2016a,b, 2017, 2018). Chromatograms were generated by determining the retention time of analytes from the analytical column with a [M-1] or [M+1] parent ion peak and a fragment ion peak corresponding to the programmed values.

Extraction efficiency was calculated with the d_8_AEA spiked recovery vial as previously described and which demonstrated equivalent recovery rates for other deuterated lipids (Bradshaw et al., 2006; Tan et al., 2006; Stuart et al., 2013; Tortoriello et al., 2013; Raboune et al., 2014; Leishman et al., 2016a,b, 2017, 2018). For each individual lipid in each of the areas, concentrations in moles per gram adjusted for extraction efficiency were compared using a 2-way ANOVA to determine the effect of age and genotype. In the case of a significant result, a one-way ANOVA with a *post hoc* Fisher’s LSD was performed to determine differences between the 4 groups (WT young, ABHD12 KO young, WT old, and ABHD12 KO old). All statistical tests were carried out using SPSS (IBM, Armonk, NY, United States). Statistical significance was defined as *p* < 0.05 and *p* < 0.10. Analyzed data are represented in tabular format illustrating both the direction and magnitude of change in lipid levels between WT and KO and between old and young adults (key and explanation of calculations found in Supplementary Figure 2). These data analysis techniques have been reviewed by the Department of Statistics at Indiana University and were validated as a logical system to compare means across these groups.

## Results

### Signal Detection in Young and Old Adult WT and ABHD12 KO Brains

Of the 77 lipoamines in our screening library (Supplementary Figure 1), over 50 were detected in most brain regions in both the WT and ABHD12 KO mice at each developmental time point (young and older adult). Within each brain area, the same number of lipids was detected in each group (young adult WT, older adult WT, young adult ABHD12 KO, and older adult ABHD12 KO). Specifically, there were 680 total discrete measures in endogenous lipids that could have been detected in each group (85 lipids in 8 brain regions) and 546 of these measures were detected, summing across the 8 brain areas. More lipids were detected in larger regions such as the CTX, STEM, and CER, which all had 72 detected lipids, and fewer in smaller regions like the HYP (56 lipids) and STR (66 lipids). At least one member of each of the NAE, *N*-acyl glycine, *N*-acyl taurine, free fatty acid, and 2-acyl glycerol species analyzed were detected in all brain regions.

### Overall Effect of Aging and ABHD12 Deletion on the Brain Lipidome

**FIGURE 1:**
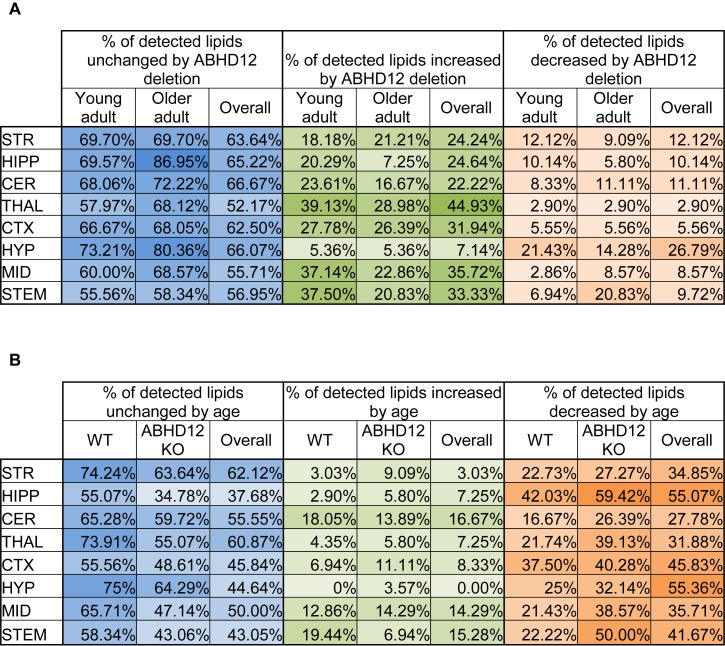
Overall effects of ABHD12 deletion and age on the brain lipidome. **(A)** Shows the percentage of lipids detected in brain areas from ABHD12 knockout (KO) mice that were not significantly different from wild-type (WT; % unchanged; blue), the percentage of lipids detected that significantly increased in concentration in each of the KO brain areas relative to WT (% increased; green) and the percentage of lipids detected that significantly decreased in concentration in the KO brain areas relative to WT (% decreased; orange). Percentages are shown for the comparison between WT and KO in young adult mice only (Young adult), and for the comparison between WT and KO in older adult mice only (Older adult), and for the comparison between WT and KO with younger and older adult mice combined into a single group (Overall). The darkest colors are the maximum values and the lightest are the minimum. STR, striatum; HIPP, hippocampus; CER, cerebellum; THAL, thalamus; CTX, cortex; HYP, hypothalamus; MID, midbrain; STEM, brainstem. As an example demonstrating how percentages were calculated, 70 lipids were detected in the MID. In the MID of young adult ABHD12 KO mice, 28 of these were significantly different relative to the young adult WT MID. To calculate the % unchanged, the percentage of those detected lipids that changed is subtracted from 100%. In this case, 28/70 ^∗^ 100% = 40%, and 100%–40% = 60%. The 60% is shown in a blue-shaded in the Figure. Of the changes in the MID, 26 of these were increases. 26/70 ^∗^ 100% = 37.14%, which is the % increased shown in a green-shaded cell. 2 of the changes were decreases, which is 2.86% of the detected lipids in the MID. This is shown as the % decreased in an orange-shaded cell. The %unchanged, %increased, and %decreased should total 100% when summing across a brain region. In this case, 60% + 37.14% + 2.86% = 100%. **(B)** Shows the percentage of lipids detected in brain areas from older adult mice that were not significantly different from younger adult mice (% unchanged; blue), the percentage of lipids detected that significantly increased in concentration in older adult brain areas relative to young adult (% increased; green) and the percentage of lipids detected that significantly decreased in concentration in the older adult brain areas relative to young adult (% decreased; orange). Percentages are shown for the comparison between young and older adult mice in wild-type mice only (WT), for the comparison between young and older adult mice in ABHD12 knockout only (ABHD12 KO), and for the comparison between young and older adult mice with WT and ABHD12 KO mice combined into a single group (Overall). The darkest colors are the maximum values and the lightest are the minimum. STR, striatum; HIPP, hippocampus; CER, cerebellum; THAL, thalamus; CTX, cortex; HYP, hypothalamus; MID, midbrain; STEM, brainstem. Percentages were calculated as above, but the number of lipids that changed with age was used instead of genotype.

The percentage of the detected lipids that were significantly modified differed as a function of age and genotype, as well as brain region (Figure 1). Across the 8 brain regions, comparing ABHD12 KO to WT only in young adult brain areas, 35% of detected lipids differed by genotype and 76% of the changes were increases relative to WT. The STEM had the most changes and the STR had the fewest (Figure 1A and Supplementary Figure 3). Only 28% of detected lipids differed with genotype in the older adult brain. However, like in WT, most of the changes were increases. The STEM also had the most genotype effects in older adult mice, whereas the HIPP had the fewest (Figure 1A and Supplementary Figure 4). When young and old adult mice were combined into genotype groups (WT and ABHD12 KO), 39% of detected lipids changed and 73% of those were increases (Figure 1A and Supplementary Figure 5).

Unlike with genotype where most of the changes were increases, most of the age-driven changes were decreases. 35% of detected lipids changed with age in WT mice, with 75% of the changes being decreases. The HIPP had the most changes and the HYP had the fewest (Figure 1B and Supplementary Figure 6). Age affected more lipids in ABHD12 KO brains, with 48% of the lipids changing, and 81% of these changes were decreases. Like in WT, the HIPP had the most changes and the HYP had the fewest (Figure 1B and Supplementary Figure 7). More age-driven changes were measured when WT and ABHD12 KO mice were combined into groups based on age (young adult and older adult), with 50% of the lipids detected changing. Almost 90% of these changes were decreases and the most were measured in the HIPP (Figure 1B and Supplementary Figure 8). In summary, age tends to decrease levels of endogenous lipids in a region-dependent manner and ABHD12 KO animals have more age-related effects.

Full lists of analyte levels in each of the brain regions and the statistical analyses are available in Supplementary Tables 1–109. Given that so many age and genotype-related changes were measured in the ∼20,000 data points collected in this study, the next section will focus on only a subset of these lipids. Heatmaps of all the group differences can be found in Supplementary Figures 3–8, which follow a key outlined in Supplementary Figure 2.

### Levels of 2-Acyl Glycerols Across the Brain Change Primarily as a Function of Genotype

**FIGURE 2:**
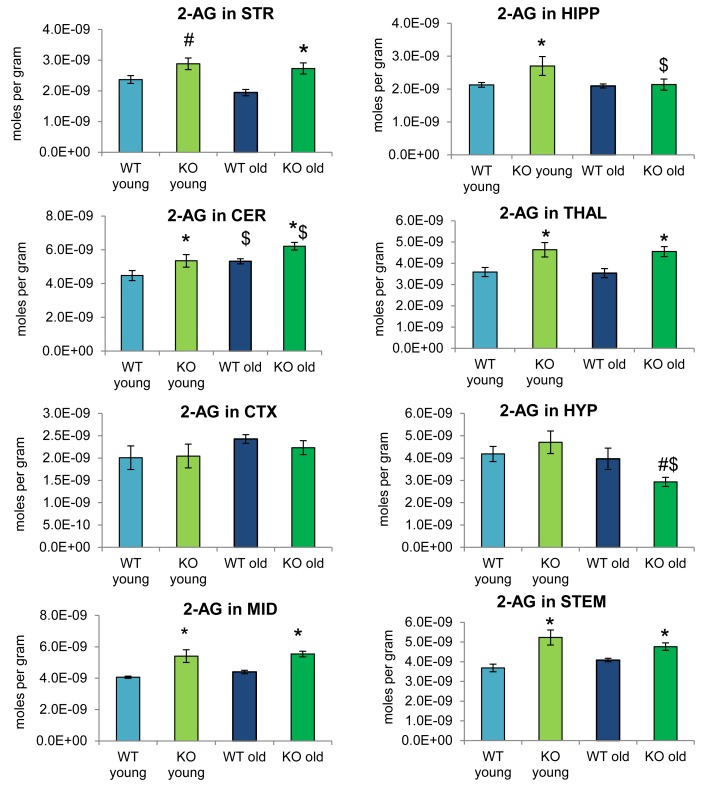
2-arachidonoyl glycerol (2-AG) levels were upregulated relative to wild-type (WT) in most ABHD12 knockout (KO) brain areas, while age had minimal effects on 2-AG levels. Bar graphs show mean 2-AG levels in brain regions from young adult WT mice (WT young, light blue bars), young adult ABHD12 KO mice (KO young, light green bars), older adult WT mice (WT old, dark blue bars), and older adult ABHD12 KO mice (KO old, dark green bars). 2-AG concentrations are expressed in moles per gram of tissue. Error bars are ± standard error of the mean. ^∗^ indicates that means were significantly different in ABHD12 KO relative to WT mice of the same age at *p* < 0.05 whereas ^#^ indicates that means were significantly different in ABHD12 KO mice at *p* < 0.10. ^$^ indicates that means were significantly different in older adults relative to younger adults of the same genotype at *p* < 0.05. Brain areas are striatum (STR), hippocampus (HIPP), cerebellum (CER), thalamus (THAL), cortex (CTX), hypothalamus (HYP), midbrain (MID), and brainstem (STEM).

**FIGURE 3:**
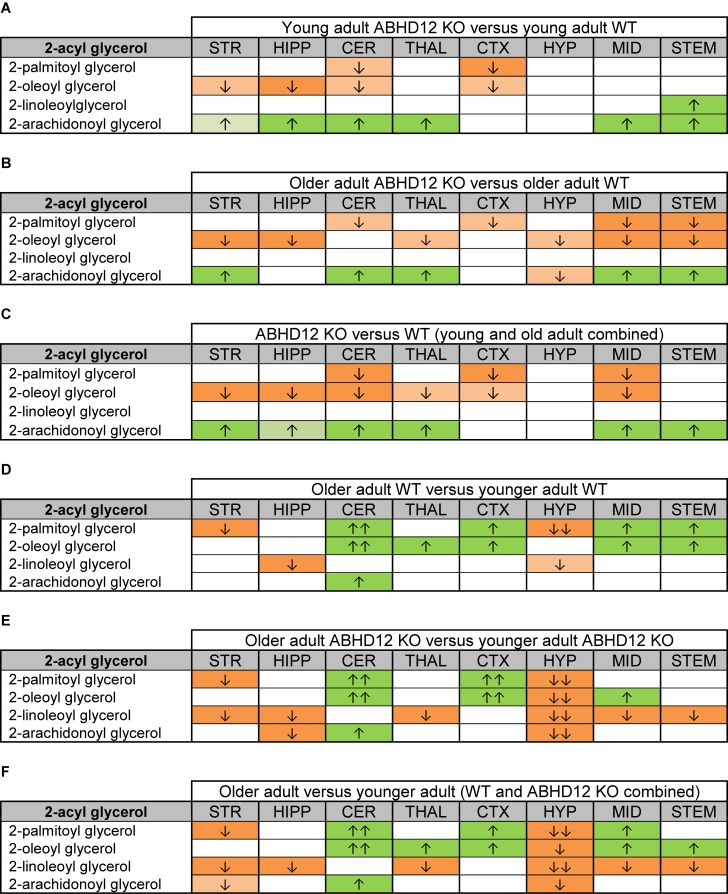
Heatmaps of effects of ABHD12 deletion and age on levels of 2-acyl glycerols in 8 brain regions. Cells with shaded arrows indicate a change for that lipid in the indicated brain area relative to the same control area. The arrow color indicates the direction of a significant result relative to control. Green colors represent increases, whereas orange colors represent decreases. Darker colors represent changes of *p* < 0.05 and lighter colors represent changes of *p* < 0.10. The number of arrows indicates the magnitude of the difference. One arrow indicates a magnitude difference of less than 1.5 fold and 2 arrows indicate a 1.5–1.99 fold change, whereas a blank cell indicates that there was no change in the lipid’s level. See Methods and Supplementary Figure 2 for more detailed description of analysis. Brain regions analyzed were the striatum (STR), hippocampus (HIPP), cerebellum (CER), thalamus (THAL), cortex (CTX), hypothalamus (HYP), midbrain (MID), and brainstem (STEM). **(A)** Shows the effect of ABHD12 deletion in younger adult brain areas, whereas **(B)** shows the effect of ABHD12 deletion in older adult brain areas. **(C)** Shows the main effect of ABHD12 deletion, combining older and younger adult mice into groups based on genotype. **(D)** Shows the effect of aging in WT mice, whereas **(E)** shows the effect of aging in ABHD12 KO mice. **(F)** Shows the main effect of aging, combining WT and ABHD12 KO mice into groups based on age.

Figure 2 provides bar graph representations of levels of 2-AG across the brain in the 4 different groups studied (WT and ABHD12 KO young adult mice and WT and ABHD12 KO older adult mice). Overall, levels of 2-AG were significantly elevated in both younger and older ABHD12 KO mice across the brain with only a few exceptions, supporting the hypothesis that ABHD12 is a biologically relevant metabolic enzyme for 2-AG. Figure 3 provides a representation of the statistical output of the analyses of the interactions of these 4 groups along genotype and age with the addition of the other 2-acyl glycerol lipids measured here: 2-LG, 2-OG, and 2-PG. These lipids were not affected by ABHD12 deletion in the same way as 2-AG. Instead, there were region-dependent decreases in 2-PG and 2-OG in ABHD12 KO mice, with more decreases in these lipids occurring in the older adult ABHD12 KO mice, whereas 2-LG was relatively unaffected (Figure 2, 3A–C and Supplementary Figures 3–5).

It is interesting to note that the only effect of age on 2-AG levels in the WT mice was an increase in the CER, which was mirrored in the ABHD12 KO mouse. The only additional change in 2-AG the ABHD12 KO mice with age was a decrease in the HIPP and the HYP in the older mice, which effectively brought them down to the level of the older WT. Compared to 2-AG, the other 2-acyl glycerols were more affected with age. More age-related differences in 2-acyl glycerols were measured in the ABHD12 KO animals. For example, 2-LG decreased with age in 6 regions of the ABHD12 KO brain but only in 2 regions of the WT brain. Older adult ABHD12 KO mice had lower levels of 2-acyl glycerols in the HYP, which was not the case in WT (Figure 2, 3D–F and Supplementary Figures 6–8). In summary, ABHD12 KO mice have significantly elevated levels of 2-AG in most brain regions, but ABHD12 deletion does not produce similar increases in 2-PG, 2-OG, or 2-LG. In contrast, levels of 2-PG, 2-OG, and 2-LG change more with aging, whereas few age-related changes in 2-AG were observed.

### Effects of ABHD12 Deletion and Aging on Levels of NAEs

**FIGURE 4:**
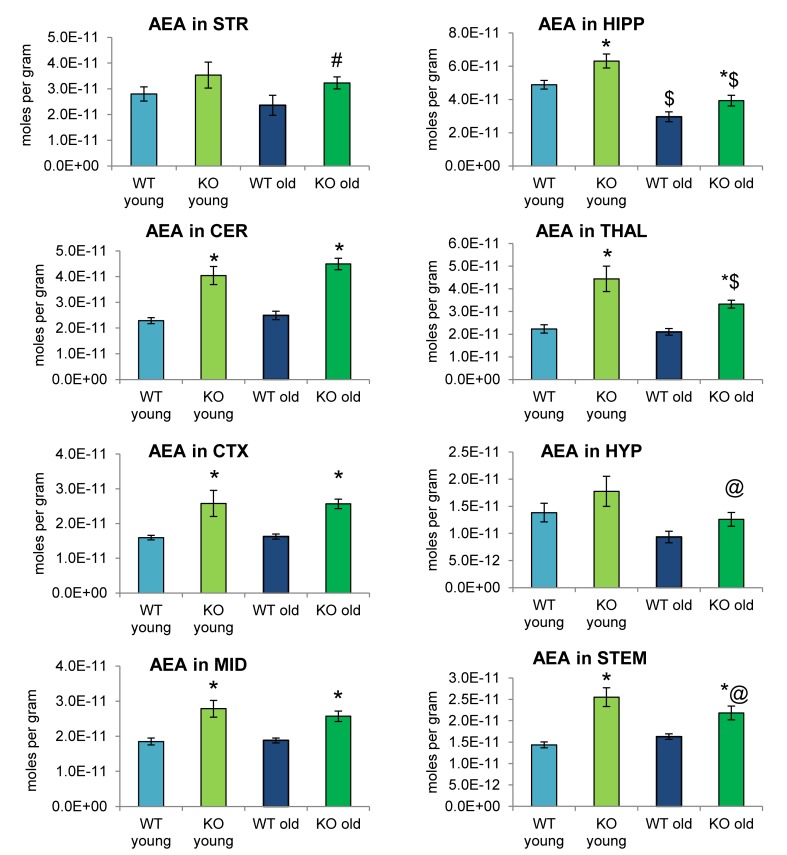
*N*-arachidonoyl ethanolamine (AEA) levels were upregulated relative to wild-type (WT) in most ABHD12 knockout (KO) brain areas, while age had minimal effects on AEA levels. Bar graphs show mean AEA levels in brain regions from young adult WT mice (WT young, light blue bars), young adult ABHD12 KO mice (KO young, light green bars), old adult WT mice (WT old, dark blue bars), and old adult ABHD12 KO mice (KO old, dark green bars). AEA concentrations are expressed in moles per gram of tissue. Error bars are ± standard error of the mean. ^∗^ indicates that means were significantly different in ABHD12 KO relative to WT mice of the same age at *p* < 0.05 whereas ^#^ indicates that means were significantly different in ABHD12 KO mice at *p* < 0.10. ^$^ indicates that means were significantly different in older adults relative to younger adults of the same genotype at *p* < 0.05 whereas ^@^ indicates that the means were significantly different in older adults relative to younger adults of the same genotype at *p* < 0.10. Brain areas are striatum (STR), hippocampus (HIPP), cerebellum (CER), thalamus (THAL), cortex (CTX), hypothalamus (HYP), midbrain (MID), and brainstem (STEM).

**FIGURE 5:**
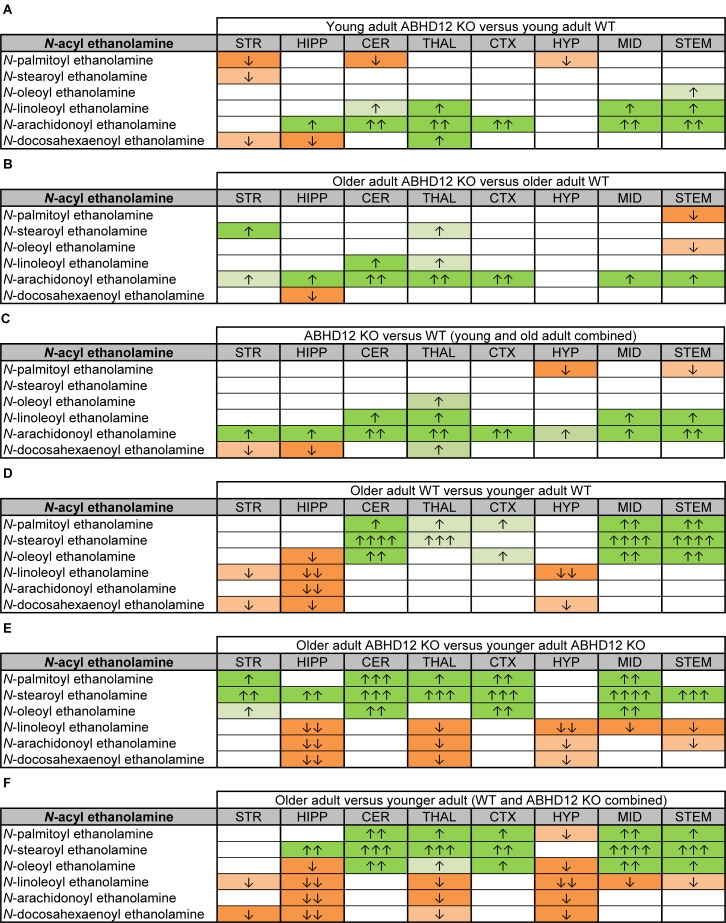
Heatmaps of effects of ABHD12 deletion and age on levels of *N*-acyl ethanolamines in 8 brain regions. Cells with shaded arrows indicate a change for that lipid in the indicated brain area relative to the same control area. The arrow color indicates the direction of a significant result relative to control. Green colors represent increases, whereas orange colors represent decreases. Darker colors represent changes of *p* < 0.05 and lighter colors represent changes of *p* < 0.10. The number of arrows indicates the magnitude of the difference. One arrow indicates a magnitude difference of less than 1.5 fold, 2 arrows indicate a 1.5–1.99 fold change, 3 arrows indicate a 2–2.99 fold change, and 4 arrows indicate a 3–9.99 fold change. A blank cell indicates that there was no change in the lipid’s level. See Methods and Supplementary Figure 2 for more detailed description of analysis. Brain regions analyzed were the striatum (STR), hippocampus (HIPP), cerebellum (CER), thalamus (THAL), cortex (CTX), hypothalamus (HYP), midbrain (MID), and brainstem (STEM). **(A)** Shows the effect of ABHD12 deletion in younger adult brain areas, whereas **(B)** shows the effect of ABHD12 deletion in older adult brain areas. **(C)** Shows the main effect of ABHD12 deletion, combining older and younger adult mice into groups based on genotype. **(D)** Shows the effect of aging in WT mice, whereas **(E)** shows the effect of aging in ABHD12 KO mice. **(F)** Shows the main effect of aging, combining WT and ABHD12 KO mice into groups based on age.

Analogous to Figure 2, 4 shows mean levels of the eCB AEA in each brain region from each of the 4 groups analyzed. Levels of AEA were significantly elevated in most brain regions of the ABHD12 KO brain in both age groups. In addition to AEA, there are 5 NAEs in the screening library that were affected by ABHD12 deletion and by aging and changes in these additional NAEs are shown in Figure 5. Unlike the consistent upregulation of AEA in ABHD12 KO mice, the effects of ABHD12 deletion on other NAEs were inconsistent, with isolated increases and decreases (Figure 4, 5A–C and Supplementary Figures 3–5).

Aging had little effect on AEA in WT animals, decreasing in the HIPP only. In contrast, AEA decreased in 4 of 8 regions in ABHD12 KO animals. Interestingly, age-related changes in NAEs containing saturated (*N*-palmitoyl ethanolamine and *N*-stearoyl ethanolamine) or monounsaturated (*N*-oleoyl ethanolamine) acyl moieties tended to be increases, whereas changes in NAEs containing polyunsaturated acyl chains (*N*-linoleoyl ethanolamine, AEA, *N*-docosahexaenoyl ethanolamine) tended to be decreases. The exception was the HYP, where any change in an NAE was a decrease in the older adult mice relative to younger adult (Figure 4, 5D–F and Supplementary Figures 6–8). In summary, ABHD12 deletion upregulated levels of AEA in most brain regions, but other NAEs did not follow this pattern. Outside of the HYP, whether NAEs increase or decrease with age was mainly dependent on the degree of unsaturation of the fatty acid conjugate. Those conjugates with long-chain, unsaturated fatty acids decreased whereas, those with shorter chain saturated fatty acids increased.

### Effects of ABHD12 Deletion on Lipids Derived From AA

**FIGURE 6:**
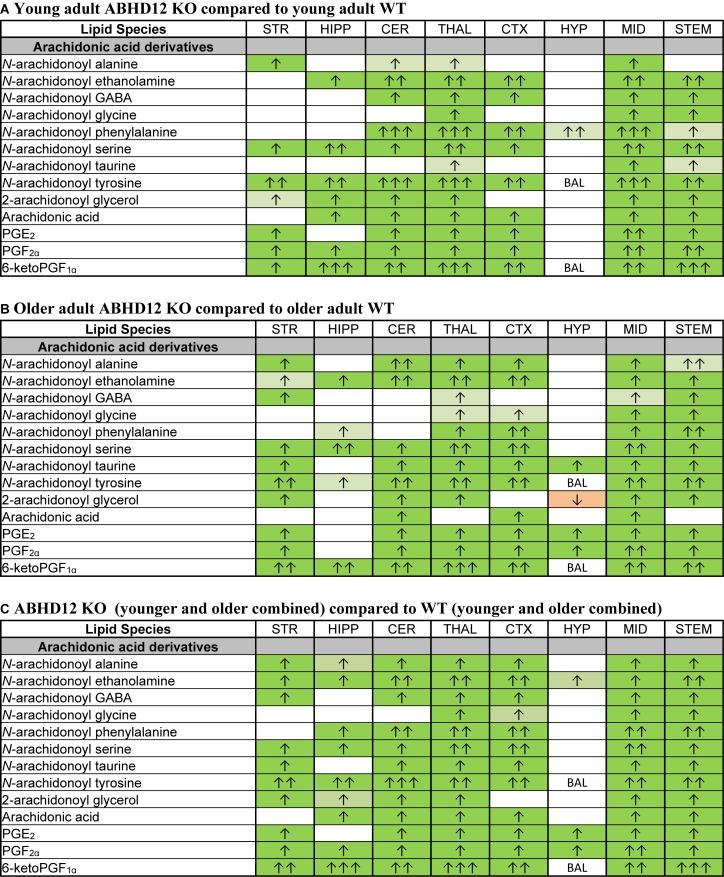
Genotype effects on arachidonic acid-derived CNS lipids. Heatmaps show effects of ABHD12 deletion on levels of arachidonic acid and targeted arachidonic acid-derived lipids in the striatum (STR), hippocampus (HIPP), cerebellum (CER), thalamus (THAL), cortex (CTX), hypothalamus (HYP), midbrain (MID), and brainstem (STEM). Cells with shaded arrows indicate a change for that lipid in the indicated ABHD12 knockout (KO) brain area relative to the same wild-type (WT) control area. The arrow color indicates the direction of a significant result in the KO brain area relative to WT. Green colors represent increases, whereas orange colors represent decreases. Darker colors represent changes of *p* < 0.05 and lighter colors represent changes of *p* < 0.10. The number of arrows indicates the magnitude of the difference. One arrow indicates a magnitude difference of less than 1.5 fold, 2 arrows indicate a 1.5–1.99 fold change, and 3 arrows indicate a 2–2.99 fold change. A blank cell indicates that there was no change in the lipid’s level. BAL, below analytical limit; meaning that the indicated analyte was not detected in all samples from that region. See Methods and Supplementary Figure 2 for more detailed description of analysis. **(A)** Effects of genotype in younger adult animals. **(B)** Effects of genotype in older adult animals. **(C)** ABHD12 KO (young and older adult animals combined) compared to WT (young and older adult animals combined).

Of all the lipids in the screening library, those containing or derived from AA were most affected by ABHD12 deletion. When considering just the young adult mice, all of the changes in AA-derived lipids were increases relative to WT. There were elevations in all of the detected AA-derived lipids in the ABHD12 KO THAL and MID, including 8 AA-derived lipoamines, 2-AG, AA, and all 3 PGs. The HYP stood out because only *N*-arachidonoyl phenylalanine increased in this area (Figure 6A and Supplementary Figure 3). When comparing the effects of ABHD12 deletion on AA-derived lipids in older adult brain areas, there were also many increases in AA-derived lipids. However, in this comparison, only the MID had increases in all of the detected AA-derived lipids. The HYP also stood out in older adults because it was the only area where a decrease in an AA-derived lipid was associated with ABHD12 deletion (Figure 6B and Supplementary Figure 4). When age groups were combined, AEA and PGF_2α_ were upregulated in every KO brain area relative to WT and every detected AA-derived lipid was upregulated in the THAL, MID, and STEM (Figure 6C and Supplementary Figure 5). In summary, bioactive lipids derived from AA are preferentially upregulated with ABHD12 deletion and were more likely to be affected than those derived from other fatty acids.

### Effects of Age on Levels of AA-Derived Lipids

**FIGURE 7:**
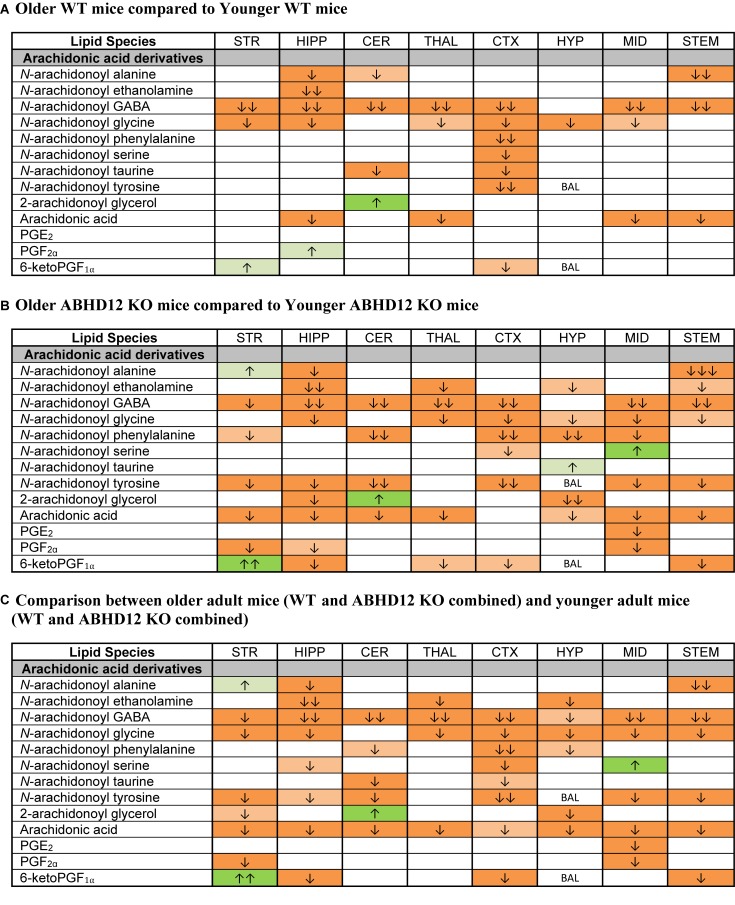
Age effects on arachidonic acid-derived CNS lipids. Heatmaps show effects of age on levels of arachidonic acid and arachidonic acid-derived lipids in the striatum (STR), hippocampus (HIPP), cerebellum (CER), thalamus (THAL), cortex (CTX), hypothalamus (HYP), midbrain (MID), and brainstem (STEM). Cells with shaded arrows indicate a change for that lipid in the indicated older adult brain area relative to the same younger adult area. The arrow color indicates the direction of a significant result in the older adult brain region relative to younger adult levels in the same region. Green colors represent increases, whereas orange colors represent decreases. Darker colors represent changes of *p* < 0.05 and lighter colors represent changes of *p* < 0.10. The number of arrows indicates the magnitude of the difference. One arrow indicates a magnitude difference of less than 1.5 fold, 2 arrows indicate a 1.5–1.99 fold change, and 3 arrows indicate a 2–2.99 fold change. A blank cell indicates that there was no change in the lipid’s level due to age. BAL, below analytical limits. See Methods and Supplementary Figure 2 for more detailed description of analysis. **(A)** Shows the effect of aging in wild-type (WT) mice, whereas **(B)** shows the effect of aging in ABHD12 knockout (KO) mice. **(C)** Shows the comparison between older adult mice (WT and ABHD12 KO combined) and younger adult mice (WT and ABHD12 KO combined).

Whereas ABHD12 deletion tended to increase AA and AA-derived lipids, aging was more likely to decrease these lipids, both in WT and in ABHD12 KO animals. In WT animals, any age-related change in an AA-derived lipoamine was a decrease. The most consistent effect of aging was on A-GABA, which decreased in every region except the HYP. By contrast, there was very little effect of age on PG levels in WT mice (Figure 7A and Supplementary Figure 6). Twenty-three more age-related changes in levels of AA and AA-derived lipids were shown in ABHD12 KO mice. *N*-arachidonoyl tyrosine and AA were more affected by age in ABHD12 KO brain as well as isolated age-related increases in AA-derived lipoamines which were not measured in WT mice (Figure 7B and Supplementary Figure 7). When data were combined by age group, there were significant decreases in A-GABA and AA in every brain region (Figure 7C and Supplementary Figure 8). In summary, the effects of aging are genotype and region-dependent, but there is an overall tendency for AA-derived lipids to decrease with age. This also means that ABHD12 deletion still increased concentrations of AA-derived lipids even when baseline levels were lower in older adult mice.

### Levels of *N*-Acyl GABAs and *N*-Acyl Glycines Decrease With Age

**FIGURE 8:**
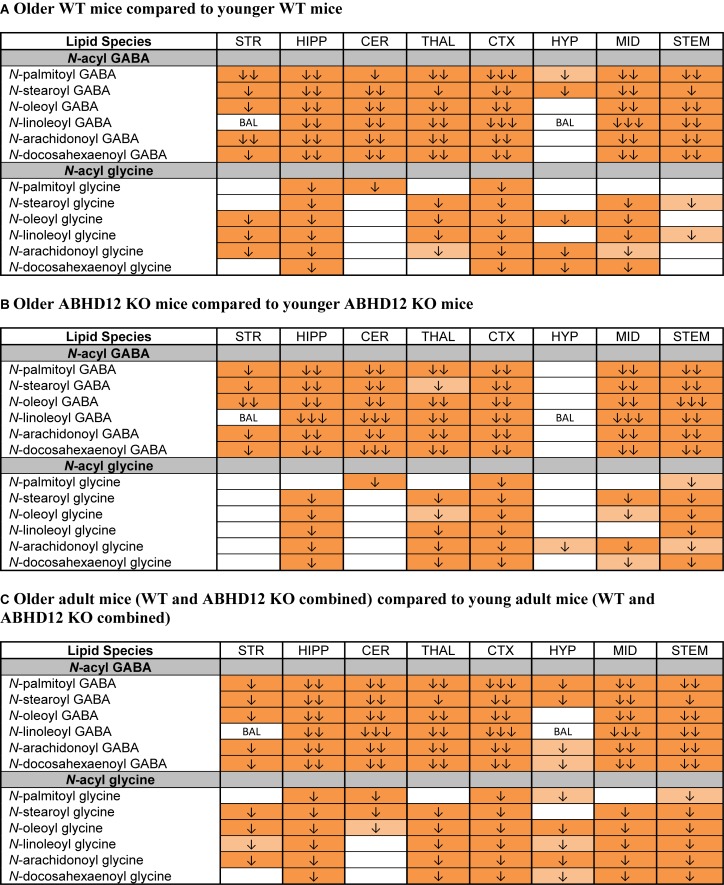
Levels of *N*-acyl GABAs and *N*-acyl glycines decline with age. Heatmaps show effects of aging on levels of targeted *N*-acyl GABAs and *N*-acyl glycines in the striatum (STR), hippocampus (HIPP), cerebellum (CER), thalamus (THAL), cortex (CTX), hypothalamus (HYP), midbrain (MID), and brainstem (STEM). Cells with shaded arrows indicate a change for that lipid in the indicated older adult brain area relative to the same younger adult control area. The arrow color indicates the direction of a significant result relative to young adult animals. Green colors represent increases, whereas orange colors represent decreases. Darker colors represent changes of *p* < 0.05 and lighter colors represent changes of *p* < 0.10. The number of arrows indicates the magnitude of the difference. One arrow indicates a magnitude difference of less than 1.5 fold, 2 arrows indicate a 1.5–1.99 fold change, and 3 arrows indicate a 2–2.99 fold change. A blank cell indicates that there was no change in the lipid’s level. BAL, below analytical limit; meaning that the indicated analyte was not detected in all samples from that region. See Methods and Supplementary Figure 2 for more detailed description of analysis. **(A)** Shows how age affected levels of *N*-acyl GABAs and *N*-acyl glycines in wild-type (WT) animals. **(B)** Shows how age affected levels of *N*-acyl GABAs and *N*-acyl glycines in ABHD12 knockout (KO) animals. **(C)** Shows the comparison between older adult mice (WT and ABHD12 KO combined) and younger adult mice (WT and ABHD12 KO combined).

The most robust overall effect of aging was the significant reductions in levels of *N*-acyl GABA and *N*-acyl glycine families of signaling lipids both WT and ABHD12 KO animals (Figure 8 and Supplementary Figure 6). Like in WT mice, all 6 *N*-acyl GABAs were downregulated in the older adult ABHD12 KO HIPP, CER, THAL, CTX, MID, and STEM. All 6 *N*-acyl glycines were downregulated in the ABHD12 KO CTX and STEM (Figure 8B and Supplementary Figure 7). When ABHD12 KO and WT mice were combined into age groups, all 6 *N*-acyl GABAs decreased with age in the HIPP, CER, THAL, CTX, MID, and STEM, and all 6 *N*-acyl glycines decreased in the HIPP, CTX, and STEM (Figure 8C and Supplementary Figure 8). In summary, levels of *N*-acyl GABAs and *N*-acyl glycines strongly decline with age, regardless of the identity of the *N*-acyl moiety and regardless of genotype.

## Discussion

Here we show that deletion of ABHD12 causes widespread disruption to the mouse brain lipidome with increases in AA-derived lipids such as eCBs, AA-derived lipoamines, AA itself, and PGs being the primary effect. These changes in the lipidome are present in younger adult ABHD12 KO mice, which do not yet display PHARC-like symptoms, as well as in older adult ABHD12 KO mice, and may support neuroinflammation and contribute to the development of PHARC-like symptoms.

### ABHD12 Deletion Affects 2-AG Levels and Upregulates Other AA-Derived Lipids

Although 2-AG is a substrate for ABHD12 (Blankman et al., 2013), ABHD12 is hypothesized to be responsible for only 9% (Savinainen et al., 2012) to 0% of hydrolysis of the brain’s 2-AG (Blankman et al., 2013). In the current study, 2-AG increased in 6 of 8 brain areas in ABHD12 KO mice. The magnitude of the increase in 2-AG in ABHD12 KO brain areas was much smaller than the magnitude increase in 2-AG in brain areas from mice missing the canonical 2-AG hydrolyzing enzyme, MAGL (Leishman et al., 2016a), which is hypothesized to be responsible for over 90% of whole brain 2-AG hydrolysis (Blankman et al., 2007), consistent with the idea that ABHD12 hydrolyzes only a small fraction of 2-AG. As ABHD12 deletion significantly impacts 2-AG levels only in certain brain areas, it likely has regional specialization. Indeed, the expression of ABHD12 is not uniform across the brain and varies by brain region (Fiskerstrand et al., 2010). However, in areas where levels of 2-AG did not increase in ABHD12 KO mice, like the CTX and HYP, ABHD12 expression is relatively high (Fiskerstrand et al., 2010), suggesting that there needs to be further investigation as to how the 2-AG hydrolysis activity of ABHD12 varies by region. It is possible that microglia are driving the increase in 2-AG in that it has been suggested that ABHD12 contributes to 2-AG hydrolysis preferentially in microglia (Viader et al., 2016). Microglia from ABHD12 KO brains had elevated 2-AG; however, AA and PGs in ABHD12 KO microglia were not significantly different than WT microglia (Viader et al., 2016), suggesting that multiple cell types are contributing to the increases in AA-derived lipids in ABHD12 KO.

Beyond 2-AG, other lipids derived from AA were especially sensitive to ABHD12 deletion, suggesting a change upstream in the AA cascade is driving the changes in the lipidome. One upstream change in ABHD12 KO that might promote the increased levels of AA-derived lipids is in levels of PS lipids, which are similar to lysoPS lipids but have an additional acyl group that can be removed by lipases (Frasch and Bratton, 2012). AA-containing PS lipids were upregulated in ABHD12 KO brain tissues (Blankman et al., 2013). In contrast, the 11 other PS lipids measured, which contained other fatty acids, did not change or were downregulated in ABHD12 KO brains (Blankman et al., 2013). Although AA-containing PS phospholipids were upregulated in ABHD12 KOs, ABHD12 did not have any enzymatic activity on substrates with 2 acyl chains, such as PS and DAG (Blankman et al., 2013); eliminating the possibility that ABHD12 is a PS lipase directly responsible for the formation of lysoPS lipids that are upregulated in ABHD12 KO. Thus, the identity of the PS lipase(s) that generated the increased lysoPS in ABHD12 KO was investigated and ABHD16A was identified as a novel brain PS lipase (Kamat et al., 2015). Pharmacological and genetic disruption of ABHD16A reduced lysoPS in the mouse brain and in lymphoblasts derived from patients with PHARC, but did not change eicosanoids (Kamat et al., 2015).

Elevated levels of AA-enriched PS lipids in ABHD12 KO mice might underlie the increase in AEA levels that we measured throughout the ABHD12 KO brain. PS can be converted to PE, a substrate for NAE biosynthesis, via a decarboxylase enzyme in the mitochondria (Vance and Tasseva, 2013). PE is then converted to the NAE precursor, NAPE, via a calcium dependent *N*-acyl transferase recently identified as cytosolic PLA_2_G4E (Ogura et al., 2016). Upregulated AA-containing PS lipids may contribute to the increase in free AA and PGs via PLA_2_. PLA_2_ releases AA from phospholipid pools. COX-2 oxidizes AA and forms the endoperoxide PGH_2_. PG synthases form the PGs (PGE_2_, PGD_2_, and PGF_2α_) and prostacyclin (PGI_2_) (Funk, 2001).

### Comparison Between ABHD12 Deletion and Deletion of Other eCB System Proteins

Using the same methodology, we previously examined the effects of deleting MAGL, FAAH, NAPE-PLD, and CB_1_ on the lipidome in 8 regions of the young adult male mouse brain (Leishman et al., 2016a,b). Collapsing across the 8 brain regions, 48% of detected lipids changed in FAAH KO (211 changes out of 444 lipids measured), 34% of detected lipids changed in MAGL KO (139/406), and 25% of detected lipids changed in NAPE-PLD KO (109/428) (Leishman et al., 2016a,b). CB_1_ deletion had less of an impact on the lipidome, with 22% of detected lipids changing across the 8 brain areas (102/457), and levels of eCBs did not change in CB_1_ KO (Leishman et al., 2016a). In young male mice (the same age and sex as our previous KO studies), ABHD12 deletion affected 35% of affected lipids (192/546), meaning that around the same proportion of the lipidome changed with ABHD12 and MAGL deletion and that deletion of an enzyme that is only responsible for a smaller portion of eCB metabolism (ABHD12) still has profound effects on the eCB-related lipidome. Consistent with the hypothesis that ABHD12 is mostly functioning as an lysoPS lipase (Blankman et al., 2013), and that its deletion increases lipids upstream of lipids measured in this study, like lysoPS – which could be precursors for lipids like AA and eCBs, ABHD12 deletion had more changes in lipid levels that were increases compared to other enzyme deletions (Leishman et al., 2016a,b). 76% of the changes in lipid levels measured across the 8 regions of ABHD12 KO were increases (146 increases out of 192 changes), whereas 66% were increases in FAAH KO (140/211), 53% were increases in MAGL KO (73/139), and only 30% were increases in NAPE-PLD KO (33/109) (Leishman et al., 2016a,b).

Comparing the effects of ABHD12 deletion and MAGL deletion reveals important differences between the 2 enzymes (Blankman et al., 2007). It has been suggested that ABHD12 deletion impairs MAGL activity because ABHD12 knockdown in ABHD12-transfected HEK cells decreased free glycerol after incubation with 2-AG, which were presumed to be a product of hydrolysis via MAGL (Tingaud-Sequeira et al., 2017). However, the effects of ABHD12 deletion on levels of other AA-derived lipids did not resemble MAGL deletion, wherein AA and PGs strongly decreased (Nomura et al., 2011; Leishman et al., 2016a). In contrast, AA and PGs increased in ABHD12 KO brain areas. Furthermore, the other 2-acyl glycerols in the screening library, like 2-LG, increased after MAGL deletion, demonstrating that MAGL is responsible for hydrolysis of monoacylglycerols with non-AA acyl groups (Leishman et al., 2016a). 2-LG did not change in ABHD12 KO. Thus, the increase in 2-AG in ABHD12 KO is most likely not due to an overall decrease in monoacylglycerol hydrolysis activity, but a small contribution of ABHD12 to 2-AG hydrolysis cannot be fully ruled out.

### The Generation of a Neuroinflammatory Phenotype ABHD12 KO Mice

α/β Hydrolase domain-12 deletion is pro-inflammatory, evidenced by previous reports of increased inflammatory cytokines in ABHD12 KO mice (Blankman et al., 2013). ABHD12 is mostly found in microglia, which are an important cell type for regulating neuroinflammation (Blankman et al., 2013; Zhang et al., 2014). However, microglia also contribute to healthy brain development and to synapse formation (Kettenmann et al., 2011) as well as pruning of both healthy and ischemic synapses (Wake et al., 2009). Although microglia can actively phagocytose synapses, they can also secrete growth factors that contribute to neuronal growth and survival (Baker et al., 2004). TLR2 is a receptor expressed on microglia that is part of the immune response that regulates microglial phagocytosis. (Kettenmann et al., 2011). It was previously shown that brain levels of lysoPS TLR2 ligands were upregulated with ABHD12 deletion (Blankman et al., 2013; Ogasawara et al., 2018). Consistent with the neuroinflammation and neurodegeneration hypothesis of PHARC, there was increased microglial activation in ABHD12 KO mouse brains (Blankman et al., 2013).

Apart from the changes in lysoPS lipids in ABHD12 KO (Blankman et al., 2013), many of the lipids that changed in ABHD12 KO mice have roles in inflammation and microglial activation. For example, certain PGs, which can promote neuroinflammation (Nomura et al., 2011), increased in every brain region of old and young adult ABHD12 KO mice. The consistent increase in PG levels suggests that ABHD12 may be important for microglia regulation of PG production (Cantaut-Belarif et al., 2017) and PGs have an important role in microglial activation by acting at PG receptors expressed on microglia (Nagano et al., 2017). As PGs were upregulated in young and old ABHD12 KO brains, it can be inferred that the increases in PGs are not a consequence of PHARC symptoms. Instead, there is a lipid environment in young adult ABHD12 KO brains that may support the development of PHARC-like symptoms by increasing inflammation. Follow up studies should examine whether changes in the lipidome in ABHD12 KO are present at younger developmental time points, as these might cause organizational differences in neurocircuitry that are then later activated to contribute to PHARC-like symptoms. Suggesting a potential role of ABHD12 in early development that could be further explored, ABHD12 expression is very high during early organ development in zebrafish (Oltrabella et al., 2017).

Beyond PGs, other changes in the ABHD12 KO lipidome might support microglial activation and migration, which are markers of neuroinflammation. For example, the AEA metabolite (Bradshaw et al., 2009) NAGly was upregulated in ABHD12 KO brains. NAGly drives microglial migration through a GPR18-dependent mechanism (McHugh et al., 2010), and alters microglial morphology (McHugh et al., 2010, 2012). In addition to stimulating microglial migration, exposure to NAGly causes the production of pro-inflammatory cytokines in cultured BV-2 microglial cells (McHugh et al., 2014). Upregulated NAGly could potentially contribute to the higher levels of cytokines previously measured in the ABHD12 KO brain (Blankman et al., 2013). The finding that PGs and NAGly increase in ABHD12 KO brain areas is novel; however, it is consistent with the concept that ABHD12 deletion is pro-inflammatory (Blankman et al., 2013).

Analytical methods that measured eCB content in rodent brain tissue were applied to human brain tissue and reported similar ranges of eCB levels between species (Malinen et al., 2009; Lehtonen et al., 2010, 2011). Reflecting the animal literature, studies on post-mortem tissue from patients with neurodegenerative disorders revealed a neuroinflammatory phenotype, often accompanied by elevated AA and PG signaling (Bilkei-Gorzo, 2012; Di Marzo et al., 2015). For example, AA levels were increased in Alzheimer’s disease brain tissue (Furman et al., 2018). CNS tissue from Alzheimer’s disease patients contained markers of neuroinflammation, such as activated microglia and pro-inflammatory cytokines, which correlated with increased expression of biosynthetic enzymes for PGs (Rao et al., 2011). There is also evidence of an inflammatory component to schizophrenia (Rao et al., 2013), possibly accompanied by a dysregulation of eCB levels (Muguruza et al., 2013). In the prefrontal cortex, cerebellum, and hippocampus of schizophrenia patients, levels of 2-AG were increased compared to control (Muguruza et al., 2013). Another analysis of the human prefrontal cortex reported increases in 2-AG in tissue from schizophrenia patients (Yu et al., 2018). Furthermore, expression of PLA_2_ and COX-2 was elevated in the prefrontal cortex of patients with schizophrenia (Rao et al., 2013). As PHARC is a disorder hypothesized to have a neuroinflammatory component, and increased 2-AG, AA, and PG signaling is correlated with neuroinflammation in rodents and humans, it is possible that PHARC patients have similar disruptions in CNS lipid levels as ABHD12 KO mice.

### Signaling Consequences of Elevated eCB Levels in ABHD12 KO

Potentially affecting signaling via CB_1_ and CB_2_ cannabinoid receptors, AEA and 2-AG increased with ABHD12 deletion. The effects of upregulated eCB levels may be cell type dependent because expression of CB_2_ is primarily restricted to the periphery (Atwood and Mackie, 2010) and microglia (Zhang et al., 2003), whereas expression of CB_1_ in microglia is very low (Kettenmann et al., 2011). Alterations in CB_1_ signaling might have downstream effects on the lipidome to support some of the other observed changes in lipid levels in ABHD12 KO. For example, CB_1_ activation can drive the release of PGs by activating COX-2 (Chen R. et al., 2013). It would be interesting to know whether blocking CB_1_ could prevent the increases in PGs in ABHD12 KO. Prolonged exposure to CB_1_ agonists can also drive neuroinflammation, as evidenced by microglial activation in the cerebellum of mice chronically exposed to THC (Cutando et al., 2013). Similar neuroinflammation is observed in ABHD12 KO mice (Blankman et al., 2013).

### Declining Availability of GABA May Underlie Age-Related Decreases in *N*-Acyl GABAs

In both WT and ABHD12 KO mice, *N*-acyl GABAs robustly decreased across the brain with age. A possible mechanism is that there is reduced availability of GABA as a substrate for *N*-acyl GABA biosynthesis. Supporting this hypothesis, basal and evoked GABA release from the inferior colliculus was diminished in older rats (Caspary et al., 1990) and a reduction in GABA release from the aged rat hippocampus was measured in a different study (Stanley et al., 2012). There is also evidence for age-related decreases in GABA in the human CNS, as individuals over 40 had lower CSF levels of both free and conjugated GABA (Ferraro and Hare, 1985). An age-related decline in GABA was also measured in the human brain using magnetic resonance spectroscopy (Gao et al., 2013). Thus, a decline in GABA levels may be a translational biomarker of aging It remains to be tested whether age-related decreases in *N*-acyl GABAs are a feature of the mammalian CNS or if they are specific to mice.

## Conclusion

α/β Hydrolase domain-12 deletion causes widespread disruption to the mouse brain lipidome, with a tendency to increase AA-derived lipids, including eCBs. Therefore, a “minor” player of the eCB system in terms of whole brain 2-AG enzymatic activity can still have profound effects on levels of eCBs and related lipids. Prolonged exposure to upregulated levels of AA-derived lipids may support neuroinflammation and contribute to the development of PHARC-like symptoms. Given that the changes in the lipidome between ABHD12 KO and WT mice were present during young adulthood, before PHARC symptoms develop in mice, these changes in the lipidome might be a potential target for the prevention of PHARC.

## Ethics Statement

All animal procedures were approved by the Bloomington Institutional Animal Care and Use Committee of Indiana University.

## Author Contributions

EL performed all lipid extractions, collected and analyzed HPLC/MS/MS data, assisted with tissue collection, and prepared the manuscript. KM provided the mice and assisted with the manuscript preparation. HB designed the experiments, collected the tissues, performed the dissections, and prepared the manuscript.

## Conflict of Interest Statement

HB is on the Advisory Board for Phytecs and consults on how endogenous cannabinoids function in the central nervous system. Phytecs had no financial contribution to the current work. The remaining authors declare that the research was conducted in the absence of any commercial or financial relationships that could be construed as a potential conflict of interest.

## Abbreviations

2-AG: 2-arachidonoyl glycerol
2-LG: 2-linoleoyl glycerol
2-OG: 2-oleoyl glycerol
2-PG: 2-palmitoyl glycerol
AA: arachidonic acid
ABHD6: α/β hydrolase domain-6
ABHD12: α/β hydrolase domain-12
ABHD16A: α/β hydrolase domain-16A
AEA: *N*-arachidonoyl ethanolamine
A-GABA: *N*-arachidonoyl GABA
CER: cerebellum (when discussing results from this study)
COX: cyclooxygenase
CTX: cortex (when discussing results from this study)
d_8_AEA: deuterium labeled *N*-arachidonoyl ethanolamine
DAG: diacylglycerol
DAGL: diacylglycerol lipase
eCB: endogenous cannabinoid
FAAH: fatty acid amide hydrolase
HIPP: hippocampus (when discussing results from this study)
HPLC/MS/MS: high-pressure liquid chromatography coupled to tandem mass spectrometry
HYP: hypothalamus (when discussing results from this study)
KO: knockout
lysoPS: lysophosphatidylserine
MAGL: monoacylglycerol lipase
MID: midbrain (when discussing results from this study)
NAE: *N*-acyl ethanolamine
NAGly: *N*-arachidonoyl glycine
NAPE: *N*-acyl phosphatidyl ethanolamine
NAPE-PLD: *N*-acyl phosphatidyl ethanolamine-specific phospholipase D
PE: phosphatidyl ethanolamine
PG: prostaglandin
PHARC: polyneuropathy, hearing loss, ataxia, retinitis pigmentosa, and cataracts
PLA_2_: phospholipase A_2_
PLA_2_G4E: phospholipase A_2_ group IVE
PS: phosphatidylserine
STEM: brainstem (when discussing results from this study)
STR: striatum (when discussing results from this study)
THAL: thalamus (when discussing results from this study)
THC: Δ^9^-tetrahydrocannabinol
TLR2: toll-like receptor 2
WT: wild-type
