# Supplementary material for: Elevated Levels of Arachidonic Acid-Derived Lipids Including Prostaglandins and Endocannabinoids Are Present Throughout ABHD12 Knockout Brains: Novel Insights Into the Neurodegenerative Phenotype
Source: Front Mol Neurosci. 2019 May 31;12:142. doi: 10.3389/fnmol.2019.00142 (PMC6555221; doi:10.3389/fnmol.2019.00142)
Supplement: Supplementary file 1 [file Data_Sheet_1.docx]

Supplementary Material

Elevated Levels of Arachidonic Acid-Derived Lipids Including Prostaglandins and Endocannabinoids are Present throughout ABHD12 Knockout Brains: Novel Insights into the Neurodegenerative Phenotype

Emma Leishman, Ken Mackie, Heather B Bradshaw*

*** Correspondence:** Corresponding Author: hbbradsh@indiana.edu

# Supplementary Figures and Tables

8 Supplementary Figures and 109 Supplementary Tables.

## Supplementary Figures

**Supplemental Figure 1**

| ***N*-acyl alanine** | **[M - H]^-^** | **Fragment** |  | ***N*-acyl proline** | **[M - H]^-^** | **Fragment** |
| --- | --- | --- | --- | --- | --- | --- |
| *N*-palmitoyl alanine | **326.5** | **88.09** |  | *N*-palmitoyl proline | **352.53** | **114.12** |
| *N*-stearoyl alanine | **354.55** | **88.09** |  | *N*-stearoyl proline | **380.59** | **114.12** |
| *N*-oleoyl alanine | **352.53** | **88.09** |  | *N-*oleoyl proline | **378.31** | **114.12** |
| *N*-linoleoyl alanine | **350.52** | **88.09** |  | *N*-linoleoyl proline | **376.56** | **114.12** |
| *N*-arachidonoyl alanine | **374.5** | **88.09** |  | *N*-arachidonoyl proline | **400.58** | **114.12** |
| *N*-docosahexaenoyl alanine | **398.56** | **88.09** |  | *N*-docosahexaenoyl proline | **424.6** | **114.12** |
| ***N*-acyl dopamine** | **[M - H]^-^** | **Fragment** |  | ***N*-acyl serine** | **[M - H]^-^** | **Fragment** |
| *N*-oleoyl dopamine | **416.3** | **123.2** |  | *N*-palmitoyl serine | **342.3** | **74** |
| *N*-arachidonoyl dopamine | **438.4** | **123.2** |  | *N*-stearoyl serine | **370.3** | **74** |
| ***N*-acyl ethanolamine** | **[M + H]^+^** | **Fragment** |  | *N*-oleoyl serine | **368.3** | **74** |
| *N*-palmitoyl ethanolamine | **300.29** | **62.1** |  | *N*-linoleoyl serine | **366.27** | **74** |
| *N*-stearoyl ethanolamine | **328.3** | **62.1** |  | *N*-arachidonoyl serine | **390.3** | **74** |
| *N*-oleoyl ethanolamine | **326.3** | **62.1** |  | *N*-docosahexaenoyl serine | **414.3** | **74** |
| *N*-linoleoyl ethanolamine | **324.3** | **62.1** |  | ***N*-acyl taurine** | **[M - H]^-^** | **Fragment** |
| *N*-arachidonoyl ethanolamine | **348.3** | **62.1** |  | *N*-palmitoyl taurine | **362.6** | **124** |
| *N*-docosahexaenoyl ethanolamine | **372.6** | **62.1** |  | *N*-stearoyl taurine | **390.6** | **124** |
| ***N*-acyl GABA** | **[M - H]^-^** | **Fragment** |  | *N*-oleoyl taurine | **388.6** | **124** |
| *N*-palmitoyl GABA | **340.54** | **102.1** |  | *N*-arachidonoyl taurine | **410.6** | **124** |
| *N*-stearoyl GABA | **368.58** | **102.1** |  | ***N*-acyl tryptophan** | **[M - H]^-^** | **Fragment** |
| *N*-oleoyl GABA | **366.57** | **102.1** |  | *N*-palmitoyl tryptophan | **441.63** | **203.1** |
| *N*-linoleoyl GABA | **364.54** | **102.1** |  | *N*-stearoyl tryptophan | **469.68** | **203.1** |
| *N*-arachidonoyl GABA | **388.57** | **102.1** |  | *N*-oleoyl tryptophan | **467.67** | **203.1** |
| *N*-docosahexaenoyl GABA | **412.59** | **102.1** |  | *N*-linoleoyl tryptophan | **465.65** | **203.1** |
| ***N*-acyl glycine** | **[M - H]^-^** | **Fragment** |  | *N*-arachidonoyl tryptophan | **489.67** | **203.1** |
| *N*-palmitoyl glycine | **312.26** | **74.2** |  | *N*-docosahexaenoyl tryptophan | **513.69** | **203.1** |
| *N*-stearoyl glycine | **340.3** | **74.2** |  | ***N*-acyl tyrosine** | **[M - H]^-^** | **Fragment** |
| *N*-oleoyl glycine | **338.3** | **74.2** |  | *N*-palmitoyl tyrosine | **418.59** | **180.18** |
| *N*-linoleoyl glycine | **336.3** | **74.2** |  | *N*-stearoyl tyrosine | **446.65** | **180.18** |
| *N*-arachidonoyl glycine | **360.3** | **74.2** |  | *N-*oleoyl tyrosine | **444.63** | **180.18** |
| *N*-docosahexaenoyl glycine | **384.3** | **74.2** |  | *N*-linoleoyl tyrosine | **442.61** | **180.18** |
| ***N*-acyl leucine** | **[M - H]^-^** | **Fragment** |  | *N*-arachidonoyl tyrosine | **466** | **180.18** |
| *N*-palmitoyl leucine | **368.58** | **130.1** |  | *N*-docosahexaenoyl tyrosine | **490.66** | **180.18** |
| *N*-stearoyl leucine | **396.63** | **130.1** |  | ***N*-acyl valine** | **[M - H]^-^** | **Fragment** |
| *N*-oleoyl leucine | **394.61** | **130.1** |  | *N*-palmitoyl valine | **354.31** | **116.31** |
| *N*-linoleoyl leucine | **392.6** | **130.1** |  | *N*- stearoyl valine | **382.6** | **116.14** |
| *N*-docosahexaenoyl leucine | **440.64** | **130.1** |  | *N*-oleoyl valine | **380.59** | **116.14** |
| ***N*-acyl methionine** | **[M - H]^-^** | **Fragment** |  | *N*-linoleoyl valine | **378.58** | **116.14** |
| *N*-palmitoyl methionine | **386.62** | **148.2** |  | *N*-docosahexaenoyl valine | **426.62** | **116.14** |
| *N*-stearoyl methionine | **414.64** | **148.2** |  | **2-acyl glycerol** | **[M + H]^+^** | **Fragment** |
| *N*-oleoyl methionine | **412.65** | **148.2** |  | 2-palmitoyl glycerol | **331.5** | **239.5** |
| *N*-linoleoyl methionine | **410.64** | **148.2** |  | 2-oleoyl glycerol | **357.5** | **265.2** |
| *N*-arachidonoyl methionine | **434.66** | **148.2** |  | 2-linoleoyl glycerol | **355.5** | **245** |
| *N*-docosahexaenoyl methionine | **458.68** | **148.2** |  | 2-arachidonoyl glycerol | **379.3** | **287.5** |
| ***N*-acyl phenylalanine** | **[M - H]^-^** | **Fragment** |  | **Free Fatty Acids** | **[M - H]^-^** | **Fragment** |
| *N*-palmitoyl phenylalanine | **402.59** | **164.1** |  | Oleic acid | **281.5** | **263** |
| *N*-stearoyl phenylalanine | **430.65** | **164.1** |  | Linoleic acid | **279.5** | **261** |
| *N*-oleoyl phenylalanine | **428.63** | **164.1** |  | Arachidonic acid | **303.5** | **285** |
| *N*-linoleoyl phenylalanine | **426.61** | **164.1** |  | **PhosphoNAEs** | **[M - H]^-^** | **Fragment** |
| *N*-arachidonoyl phenylalanine | **450.64** | **164.1** |  | PhosphoLEA | **403.5** | **58.5** |
| *N*-docosahexaenoyl phenylalanine | **474.66** | **164.1** |  | **Prostaglandins** | **[M - H]^-^** | **Fragment** |
|  |  |  |  | PGE_2_ | **351.2** | **315** |
|  |  |  |  | PGF_2α_ | **353.3** | **309.2** |
|  |  |  |  | 6-keto-PGF_1α_ | **369.3** | **206.9** |

**Supplemental Figure 1:** **Lipids in HPLC/MS/MS screening library with parent ion and fragment ion masses.** Lipids are grouped by amide family and lipids in these families are screened for simultaneously using a multiple reactions monitoring (MRM) method. Negative ionization mode, resulting in a [M – H]^-^ parent ion, is used for all methods except the *N*-acyl ethanolamine and 2-acyl glycerol methods, which uses positive ionization and generates a [M + H]^+^ parent ion. Chromatograms were generated by determining the retention time of analytes with parent ion peaks and fragmentation ion peaks corresponding to the programmed values. The retention time was then compared to the retention time of a standard for the suspected compound. If the retention times matched, then the concentration of the compound was determined by calculating the area under the curve for the unknown and comparing it to the calibration curve obtained from the standards. Therefore, unknown lipids are matched to known standards according to retention time from the analytical column and according to their mass fingerprint. Only the striatum was analyzed for *N-*acyl dopamines.

**Supplemental Figure 2**

| Statistical inference | | Increases | | Decreases | |
| --- | --- | --- | --- | --- | --- |
| Decrease relative to control (p≤.05) |  | ↑↑↑↑↑ | 10 or more times higher than control | ↓ | 1-1.49 times lower than control |
| Decrease relative to control (.05<p≤.10) |  | ↑↑↑↑ | 3-9.99 times higher than control | ↓↓ | 1.50-1.99 times lower than control |
| Increase relative to control (.05<p≤.10) |  | ↑↑↑ | 2-2.99 times higher than control | ↓↓↓ | 2-2.99 times lower than control |
| Increase relative to control (p≤.05) |  | ↑↑ | 1.50-1.99 times higher than control | ↓↓↓↓ | 3-9.99 times lower than control |
| Below analytical limit | *BAL* | ↑ | 1-1.49 times higher than control | ↓↓↓↓↓ | 10 or more times lower than control |

**Supplemental Figure 2: Key for heatmaps showing genotype and age-related changes in lipid levels in brain regions of young and older adult wild-type (WT) and ABHD12 knockout (KO) mice.** The arrow color (left column) indicates the direction of a significant result relative to control. In the case of the KO versus WT comparisons, the control is the WT level, whereas the control is the young adult level for age comparisons. Green colors represent increases and orange color represents decreases in a lipid’s concentration. The number of arrows (right columns) indicates the magnitude of the difference. To determine the magnitude change and therefore the number of arrows to assign each significant difference, the mean level of a particular lipid in a specific region of the experimental mice was divided by that same lipid’s mean level in the same brain region of the corresponding control mice. For example, the average level of *N*-arachidonoyl ethanolamine (AEA) in the cerebellum of ABHD12 KO mice (young and older adult mice combined into a single group) was 4.31x10^-11^ moles per gram and the average level of AEA in the cerebellum of the WT (young and older adult mice combined into a single group) was 2.39x10^-11^ moles per gram; 4.31x10^-11^ divided by 2.39x10^-11^ equals 1.8, meaning that AEA levels are over 1.5 times but not quite twice as high in the cerebellum of the ABHD12 KO mice and assigning it 2 up arrows in the figures because the magnitude of change was between 1.5 and 2 times higher than WT. For decreases the process was very similar: the mean level in the experimental mice was divided by the mean level in the corresponding control mice; however, the reciprocal of the decimal was taken to express a fold decrease (if the level in the experimental mouse is ½ of the control level then that is a 2 fold decrease). As an example the mean level of *N*-arachidonoyl GABA was 7.35x10^-12^ moles per gram in the cerebellum of the older adult mice (WT and ABHD12 KO mice combined into a single group) and 1.32x10^-11^ moles per gram in the corresponding young adult cerebellum (WT and ABHD12 KO mice combined into a single group). 7.35x10^-12^ divided by 1.32x10^-11^ is 0.557 and the reciprocal of 0.557 is 1.80, meaning that the decrease is between 1.5 and 2 times young adult levels and giving it 2 down arrows on our scale.

**Supplemental Figure 3**

|  | **Change with young ABHD12 KO (relative to young WT)** | | | | | | | |
| --- | --- | --- | --- | --- | --- | --- | --- | --- |
| **Lipid Species** | STR | HIPP | CER | THAL | CTX | HYP | MID | STEM |
| *N*-stearoyl alanine |  |  |  |  |  |  | ↑ |  |
| *N*-oleoyl alanine |  |  |  |  |  | ↓ |  |  |
| *N*-arachidonoyl alanine | ↑ |  | ↑ | ↑ |  |  | ↑ |  |
| *N*-docosahexaenoyl alanine | BAL | BAL | BAL | BAL |  | BAL | BAL | ↑ |
| *N*-palmitoyl ethanolamine | ↓ |  | ↓ |  |  | ↓ |  |  |
| *N*-stearoyl ethanolamine | ↓ |  |  |  |  |  |  |  |
| *N*-oleoyl ethanolamine |  |  |  |  |  |  |  | ↑ |
| *N*-linoleoyl ethanolamine |  |  | ↑ | ↑ |  |  | ↑ | ↑ |
| *N*-arachidonoyl ethanolamine |  | ↑ | ↑↑ | ↑↑ | ↑↑ |  | ↑↑ | ↑↑ |
| *N*-docosahexaenoyl ethanolamine | ↓ | ↓ |  | ↑ |  |  |  |  |
| *N*-palmitoyl GABA | ↓ | ↓ |  |  | ↓ | ↓ | ↓ |  |
| *N*-stearoyl GABA |  |  |  |  |  | ↓ |  |  |
| *N*-oleoyl GABA |  |  |  |  |  | ↓ |  |  |
| *N*-arachidonoyl GABA |  |  | ↑ | ↑ | ↑ |  | ↑ | ↑ |
| *N*-palmitoyl glycine |  |  |  |  |  | ↓ |  |  |
| *N*-stearoyl glycine |  |  |  |  |  | ↓ |  |  |
| *N*-oleoyl glycine |  |  |  |  |  | ↓ |  |  |
| *N*-arachidonoyl glycine |  |  |  | ↑ |  |  | ↑ | ↑ |
| *N*-palmitoyl leucine |  |  |  |  |  |  |  | ↓ |
| *N*-stearoyl leucine |  |  | ↑ |  | ↑ |  |  |  |
| *N*-docosahexaenoyl leucine | ↓ |  |  | ↓↓ | ↓ | ↑↑ |  |  |
| *N*-stearoyl methionine | ↑↑ | ↑ | ↑ | ↑ |  | BAL |  |  |
| *N*-palmitoyl phenylalanine |  |  |  | ↑ |  |  | ↑↑ |  |
| *N*-stearoyl phenylalanine | ↑ | ↑ | ↑ | ↑↑ |  | ↑ | ↑ | ↑ |
| *N*-oleoyl phenylalanine |  | ↑ | ↑ | ↑ | ↑ |  | ↑↑ |  |
| *N*-linoleoyl phenylalanine | BAL | BAL |  | BAL | ↑↑ | BAL |  | BAL |
| *N*-arachidonoyl phenylalanine |  |  | ↑↑↑ | ↑↑↑ | ↑↑ | ↑↑ | ↑↑↑ | ↑ |
| *N*-docosahexaenoyl phenylalanine |  |  |  | ↑↑ | ↑↑ | BAL | ↑↑ | ↑↑ |
| *N*-palmitoyl proline |  | ↑ |  |  |  |  |  |  |
| *N*-stearoyl proline |  | ↑ |  |  | ↑ |  |  |  |
| *N*-palmitoyl serine | ↓ | ↓ | ↓ |  |  | ↓ |  | ↓ |
| *N*-oleoyl serine | ↓ | ↓ | ↓ |  |  |  |  | ↓ |
| *N*-linoleoyl serine |  |  |  |  |  | BAL |  | ↑ |
| *N*-arachidonoyl serine | ↑ | ↑↑ | ↑ | ↑↑ | ↑ |  | ↑↑ | ↑↑ |
| *N*-docosahexaenoyl serine |  |  |  | ↑↑ | ↑ |  |  |  |
| *N*-palmitoyl taurine |  |  | ↓ | ↓ |  | ↓↓ | ↓ | ↓ |
| *N*-stearoyl taurine |  | ↓ |  |  |  | ↓↓ |  | ↓ |
| *N-*oleoyl taurine |  | ↓ |  |  |  | ↓ |  |  |
| *N*-arachidonoyl taurine |  |  |  | ↑ |  |  | ↑ | ↑ |
| *N*-palmitoyl tryptophan |  |  |  | ↑↑ |  | BAL | ↑↑ |  |
| *N*-stearoyl tryptophan |  |  |  | ↑ | ↑ |  |  |  |
| *N*-oleoyl tryptophan | BAL | BAL |  | BAL |  | BAL | BAL | ↑↑↑ |
| *N*-palmitoyl tyrosine |  | ↑ |  | ↑↑ |  |  | ↑↑ | ↑ |
| *N*-stearoyl tyrosine | ↑ |  |  | ↑↑ | ↑ | BAL | ↑ | ↑↑ |
| *N-*oleoyl tyrosine | ↑↑ |  | ↑ | ↑ | ↑↑ | BAL | ↑↑ | ↑ |
| *N*-arachidonoyl tyrosine | ↑↑ | ↑↑ | ↑↑↑ | ↑↑↑ | ↑↑ | BAL | ↑↑↑ | ↑↑ |
| *N*-docosahexaenoyl tyrosine | BAL | ↑↑ |  | ↑↑ | ↑ | BAL | ↑↑ | ↑↑↑ |
| *N*-palmitoyl valine |  |  |  |  |  |  | ↑ |  |
| *N*- stearoyl valine | ↑ |  |  |  |  |  |  | ↑ |
| *N*-oleoyl valine |  |  |  |  |  | BAL |  | ↑ |
| 2-palmitoyl glycerol |  |  | ↓ |  | ↓ |  |  |  |
| 2-oleoyl glycerol | ↓ | ↓ | ↓ |  | ↓ |  |  |  |
| 2-linoleoyl glycerol |  |  |  |  |  |  |  | ↑ |
| 2-arachidonoyl glycerol | ↑ | ↑ | ↑ | ↑ |  |  | ↑ | ↑ |
| Oleic acid |  |  |  |  | ↑ |  |  |  |
| Arachidonic acid |  | ↑ | ↑ | ↑ | ↑ |  | ↑ | ↑ |
| phosphoLEA |  |  |  |  |  |  | ↑↑ | ↑↑ |
| PGE_2_ | ↑ |  | ↑ | ↑ | ↑ |  | ↑↑ | ↑ |
| PGF_2α_ | ↑ | ↑ | ↑ | ↑ | ↑ |  | ↑↑ | ↑↑ |
| 6-ketoPGF_1α_ | ↑ | ↑↑↑ | ↑↑ | ↑↑↑ | ↑↑ | BAL | ↑↑ | ↑↑↑ |

**Supplemental Figure 3: Effects of ABHD12 deletion on CNS lipid levels in young adult mice.** Cells with shaded arrows indicate a change for that lipid in the young adult ABHD12 knockout (KO) brain area relative to the same young adult wild-type (WT) area. The arrow color indicates the direction of a significant result relative to young adult WT. Green colors represent increases, whereas orange colors represent decreases in a lipid’s concentration, with darker colors indicating a significant change of p < 0.05 and light colors representing a change of p < 0.10. The number of arrows indicates the magnitude of the difference between ABHD12 KO and WT. One arrow indicates a magnitude difference of less than 1.5 fold, 2 arrows indicate a 1.5-1.99 fold change, and 3 arrows indicate a 2-2.99 fold change. BAL stands for “Below Analytical Limit,” whereas a blank cell indicates that there was no change in the lipid’s level due to ABHD12 deletion. Only lipids that were significantly affected by ABHD12 deletion in at least 1 brain area are shown here. See Methods and Supplemental Figure 2 for more detailed description of analysis. Brain areas analyzed were the striatum (STR), hippocampus (HIPP), cerebellum (CER), thalamus (THAL), cortex (CTX), hypothalamus (HYP), midbrain (MID), and brainstem (STEM).

**Supplemental Figure 4**

|  | **Change with old ABHD12 KO (relative to old WT)** | | | | | | | |
| --- | --- | --- | --- | --- | --- | --- | --- | --- |
| **Lipid Species** | STR | HIPP | CER | THAL | CTX | HYP | MID | STEM |
| *N*-stearoyl alanine |  |  |  | ↑ | ↑ |  | ↑ |  |
| *N*-oleoyl alanine | ↓ |  | ↓ |  | ↓ | ↓ | ↓ | ↓ |
| *N*-linoleoyl alanine | BAL |  |  | BAL | BAL | BAL | BAL | ↓ |
| *N*-arachidonoyl alanine | ↑ |  | ↑↑ | ↑ | ↑ |  | ↑ | ↑↑ |
| *N*-palmitoyl ethanolamine |  |  |  |  |  |  |  | ↓ |
| *N*-stearoyl ethanolamine | ↑ |  |  | ↑ |  |  |  |  |
| *N*-oleoyl ethanolamine |  |  |  |  |  |  |  | ↓ |
| *N*-linoleoyl ethanolamine |  |  | ↑ | ↑ |  |  |  |  |
| *N*-arachidonoyl ethanolamine | ↑ | ↑ | ↑↑ | ↑↑ | ↑↑ |  | ↑ | ↑ |
| *N*-docosahexaenoyl ethanolamine |  | ↓ |  |  |  |  |  |  |
| *N*-palmitoyl GABA |  |  |  |  |  |  |  | ↓ |
| *N*-stearoyl GABA |  |  |  |  |  |  |  | ↓ |
| *N*-oleoyl GABA |  |  |  |  |  |  |  | ↓ |
| *N*-arachidonoyl GABA | ↑ |  |  | ↑ |  |  | ↑ | ↑ |
| *N*-docosahexaenoyl GABA | ↓ |  |  |  |  |  |  |  |
| *N*-palmitoyl glycine |  |  |  | ↑ |  |  |  |  |
| *N*-arachidonoyl glycine |  |  |  | ↑ | ↑ |  | ↑ | ↑ |
| *N*-palmitoyl leucine |  |  | ↓ |  |  |  |  |  |
| *N*-stearoyl leucine |  |  |  |  | ↑ |  |  |  |
| *N*-oleoyl leucine |  |  | ↓ |  |  |  |  |  |
| *N*-linoleoyl leucine | ↓↓ |  |  |  |  | BAL |  |  |
| *N*-docosahexaenoyl leucine |  | ↓ | ↓ | ↓ | ↓ |  | ↓↓ |  |
| *N*-stearoyl methionine | ↑ |  |  |  |  | BAL |  |  |
| *N*-oleoyl methionine | ↓ |  |  |  |  | BAL |  |  |
| *N*-oleoyl phenylalanine |  |  |  |  |  | ↓ |  |  |
| *N*-arachidonoyl phenylalanine |  | ↑ |  | ↑ | ↑↑ |  | ↑ | ↑↑ |
| *N*-palmitoyl proline |  |  |  |  | ↑ |  |  |  |
| *N*-stearoyl proline |  |  |  |  | ↑ |  |  |  |
| *N*-palmitoyl serine |  |  | ↓ |  |  | ↓ | ↓ | ↓ |
| *N*-oleoyl serine |  |  |  |  |  | ↓ | ↓ | ↓ |
| *N*-arachidonoyl serine | ↑ | ↑↑ | ↑ | ↑↑ | ↑↑ |  | ↑↑ | ↑ |
| *N*-docosahexaenoyl serine |  |  |  | ↑ |  |  |  | ↑ |
| *N*-palmitoyl taurine |  | ↓ | ↓ |  |  | ↓ |  | ↓ |
| *N*-stearoyl taurine | ↑ |  |  | ↑ | ↑ |  |  | ↓ |
| *N-*oleoyl taurine |  |  |  | ↑ | ↑ |  |  | ↓ |
| *N*-arachidonoyl taurine | ↑ |  | ↑ | ↑ | ↑ | ↑ | ↑ | ↑ |
| *N*-stearoyl tryptophan |  |  | ↓ |  |  |  |  |  |
| *N*-oleoyl tryptophan | BAL | BAL | ↑↑ | BAL |  | BAL | BAL | ↑↑ |
| *N-*oleoyl tyrosine |  |  |  |  |  | BAL |  | ↓ |
| *N*-arachidonoyl tyrosine | ↑↑ | ↑ | ↑↑ | ↑↑ | ↑↑ | BAL | ↑↑ | ↑↑ |
| *N*-docosahexaenoyl tyrosine | BAL |  |  | ↑↑ | ↑↑ | BAL |  |  |
| *N*-palmitoyl valine | ↓↓ |  |  |  | ↓ |  |  |  |
| *N*- stearoyl valine | ↑ |  |  |  | ↑ |  |  |  |
| 2-palmitoyl glycerol |  |  | ↓ |  | ↓ |  | ↓ | ↓ |
| 2-oleoyl glycerol | ↓ | ↓ |  | ↓ |  | ↓ | ↓ | ↓ |
| 2-arachidonoyl glycerol | ↑ |  | ↑ | ↑ |  | ↓ | ↑ | ↑ |
| Linoleic acid |  |  |  |  |  | ↓ | ↑ |  |
| Arachidonic acid |  |  | ↑ |  | ↑ |  | ↑ |  |
| phosphoLEA |  |  |  |  |  |  | ↑ | ↑ |
| PGE_2_ | ↑ |  | ↑ | ↑ | ↑ | ↑ | ↑ | ↑ |
| PGF_2α_ | ↑ |  | ↑ | ↑ | ↑ | ↑ | ↑↑ | ↑ |
| 6-ketoPGF_1α_ | ↑↑ | ↑↑ | ↑↑ | ↑↑↑ | ↑↑ | BAL | ↑↑ | ↑↑ |

**Supplemental Figure 4: Effects of ABHD12 deletion on CNS lipid levels in older adult mice.** Cells with shaded arrows indicate a change for that lipid in the older adult ABHD12 knockout (KO) brain area relative to the same older adult wild-type (WT) area. The arrow color indicates the direction of a significant result relative to WT. Green colors represent increases, whereas orange colors represent decreases in a lipid’s concentration, with darker colors indicating a significant change of p < 0.05 and light colors representing a change of p < 0.10. The number of arrows indicates the magnitude of the difference between ABHD12 KO and WT. One arrow indicates a magnitude difference of less than 1.5 fold, 2 arrows indicate a 1.5-1.99 fold change, and 3 arrows indicate a 2-2.99 fold change. BAL stands for “Below Analytical Limit,” whereas a blank cell indicates that there was no change in the lipid’s level due to ABHD12 deletion. Only lipids that were significantly affected by ABHD12 deletion in at least 1 brain area are shown here. See Methods and Supplemental Figure 2 for more detailed description of analysis. Brain areas analyzed were the striatum (STR), hippocampus (HIPP), cerebellum (CER), thalamus (THAL), cortex (CTX), hypothalamus (HYP), midbrain (MID), and brainstem (STEM).

**Supplemental Figure 5**

|  | **Change with ABHD12 KO (relative to WT)** | | | | | | | |
| --- | --- | --- | --- | --- | --- | --- | --- | --- |
| **Lipid Species** | STR | HIPP | CER | THAL | CTX | HYP | MID | STEM |
| *N*-stearoyl alanine |  |  |  | ↑ | ↑ |  | ↑ |  |
| *N*-oleoyl alanine | ↓ |  | ↓ |  |  | ↓ |  |  |
| *N*-arachidonoyl alanine | ↑ | ↑ | ↑ | ↑ | ↑ |  | ↑ | ↑ |
| *N*-palmitoyl ethanolamine |  |  |  |  |  | ↓ |  | ↓ |
| *N*-oleoyl ethanolamine |  |  |  | ↑ |  |  |  |  |
| *N*-linoleoyl ethanolamine |  |  | ↑ | ↑ |  |  | ↑ | ↑ |
| *N*-arachidonoyl ethanolamine | ↑ | ↑ | ↑↑ | ↑↑ | ↑↑ | ↑ | ↑ | ↑↑ |
| *N*-docosahexaenoyl ethanolamine | ↓ | ↓ |  | ↑ |  |  |  |  |
| *N*-palmitoyl GABA | ↓ |  |  |  | ↓ | ↓ | ↓ | ↓ |
| *N*-stearoyl GABA |  |  |  |  |  | ↓ |  |  |
| *N*-oleoyl GABA |  | ↓ |  |  |  | ↓ |  |  |
| *N*-arachidonoyl GABA | ↑ |  | ↑ | ↑ | ↑ |  | ↑ | ↑ |
| *N*-docosahexaenoyl GABA | ↓ |  |  |  |  |  |  |  |
| *N*-palmitoyl glycine |  |  |  |  |  | ↓ |  |  |
| *N*-stearoyl glycine |  |  |  |  |  | ↓ |  |  |
| *N*-oleoyl glycine |  |  |  |  |  | ↓ |  |  |
| *N*-arachidonoyl glycine |  |  |  | ↑ | ↑ |  | ↑ | ↑ |
| *N*-stearoyl leucine | ↑ |  |  |  | ↑ |  |  |  |
| *N*-oleoyl leucine |  |  | ↓ |  |  |  |  |  |
| *N*-docosahexaenoyl leucine | ↓ | ↓ | ↓ | ↓ | ↓ | ↑ | ↓ |  |
| *N*-stearoyl methionine | ↑ | ↑ | ↑ | ↑ | ↑ |  |  |  |
| *N*-palmitoyl phenylalanine |  |  |  | ↑ |  |  | ↑ |  |
| *N*-stearoyl phenylalanine | ↑ | ↑ | ↑ | ↑ |  |  | ↑ | ↑ |
| *N*-oleoyl phenylalanine |  | ↑ |  | ↑ |  | ↓ | ↑ |  |
| *N*-linoleoyl phenylalanine | BAL | BAL |  | BAL | ↑↑ | BAL |  | BAL |
| *N*-arachidonoyl phenylalanine |  | ↑ | ↑↑ | ↑↑ | ↑↑ |  | ↑↑ | ↑↑ |
| *N*-docosahexaenoyl phenylalanine |  |  |  | ↑ | ↑ | BAL | ↑ | ↑ |
| *N*-palmitoyl proline |  | ↑ |  |  |  |  |  |  |
| *N*-stearoyl proline |  | ↑ |  | ↑ | ↑ |  |  |  |
| *N*-palmitoyl serine | ↓ | ↓ | ↓ |  |  | ↓ | ↓ | ↓ |
| *N*-oleoyl serine | ↓ |  | ↓ |  |  | ↓ |  | ↓ |
| *N*-linoleoyl serine |  | ↑ |  |  |  | BAL |  | ↑ |
| *N*-arachidonoyl serine | ↑ | ↑ | ↑ | ↑↑ | ↑↑ |  | ↑↑ | ↑ |
| *N*-docosahexaenoyl serine |  |  |  | ↑ | ↑ |  |  | ↑ |
| *N*-palmitoyl taurine |  | ↓ | ↓ |  |  | ↓ | ↓ | ↓ |
| *N*-stearoyl taurine |  |  |  | ↑ |  | ↓ |  | ↓ |
| *N-*oleoyl taurine |  | ↓ |  |  |  | ↓ |  | ↓ |
| *N*-arachidonoyl taurine | ↑ |  | ↑ | ↑ | ↑ |  | ↑ | ↑ |
| *N*-stearoyl tryptophan |  |  |  | ↑ | ↑ |  |  |  |
| *N*-oleoyl tryptophan | BAL | BAL | ↑↑ | BAL |  | BAL | BAL | ↑↑↑ |
| *N*-palmitoyl tyrosine |  |  |  | ↑ |  |  | ↑ |  |
| *N*-stearoyl tyrosine | ↑ |  |  | ↑ |  | BAL | ↑ | ↑ |
| *N-*oleoyl tyrosine | ↑ | ↑ |  | ↑ |  | BAL | ↑ |  |
| *N*-arachidonoyl tyrosine | ↑↑ | ↑↑ | ↑↑↑ | ↑↑ | ↑↑ | BAL | ↑↑ | ↑↑ |
| *N*-docosahexaenoyl tyrosine | BAL | ↑ |  | ↑↑ | ↑ | BAL | ↑ | ↑↑ |
| *N*-palmitoyl valine |  |  |  |  |  |  | ↑ |  |
| *N*- stearoyl valine | ↑ |  |  | ↑ | ↑ |  |  | ↑ |
| *N*-oleoyl valine |  |  |  |  |  | BAL |  | ↑ |
| 2-palmitoyl glycerol |  |  | ↓ |  | ↓ |  | ↓ |  |
| 2-oleoyl glycerol | ↓ | ↓ | ↓ | ↓ | ↓ |  | ↓ |  |
| 2-arachidonoyl glycerol | ↑ | ↑ | ↑ | ↑ |  |  | ↑ | ↑ |
| Oleic acid |  |  |  |  | ↑ | ↓ |  |  |
| Arachidonic acid |  | ↑ | ↑ | ↑ | ↑ |  | ↑ | ↑ |
| phosphoLEA |  |  |  |  |  |  | ↑ | ↑ |
| PGE_2_ | ↑ |  | ↑ | ↑ | ↑ | ↑ | ↑ | ↑ |
| PGF_2α_ | ↑ | ↑ | ↑ | ↑ | ↑ | ↑ | ↑↑ | ↑ |
| 6-ketoPGF_1α_ | ↑↑ | ↑↑↑ | ↑↑ | ↑↑↑ | ↑↑ | BAL | ↑↑ | ↑↑↑ |

**Supplemental Figure 5: Changes in CNS lipid levels in ABHD12 knockout (KO), with younger and older mice combined, compared to wild-type (WT), with younger and older mice combined.** Cells with shaded arrows indicate a change for that lipid in the ABHD12 KO brain area relative to the same WT area. The arrow color indicates the direction of a significant result relative to WT. Green colors represent increases, whereas orange colors represent decreases in a lipid’s concentration, with darker colors indicating a significant change of p < 0.05 and light colors representing a change of p < 0.10. The number of arrows indicates the magnitude of the difference between ABHD12 KO and WT. One arrow indicates a magnitude difference of less than 1.5 fold, 2 arrows indicate a 1.5-1.99 fold change, and 3 arrows indicate a 2-2.99 fold change. BAL stands for “Below Analytical Limit,” whereas a blank cell indicates that there was no change in the lipid’s level due to ABHD12 deletion. Only lipids that were significantly affected by ABHD12 deletion in at least 1 brain area are shown here. See Methods and Supplemental Figure 2 for more detailed description of analysis. Brain areas analyzed were the striatum (STR), hippocampus (HIPP), cerebellum (CER), thalamus (THAL), cortex (CTX), hypothalamus (HYP), midbrain (MID), and brainstem (STEM).

**Supplemental Figure 6**

|  | **Change with old WT (relative to young WT)** | | | | | | | |
| --- | --- | --- | --- | --- | --- | --- | --- | --- |
| **Lipid Species** | STR | HIPP | CER | THAL | CTX | HYP | MID | STEM |
| *N*-palmitoyl alanine |  | ↓↓ |  |  |  | ↓ |  | ↓ |
| *N*-stearoyl alanine |  | ↓↓ |  |  |  |  |  | ↓ |
| *N*-oleoyl alanine | ↑ | ↓ | ↑ |  | ↑ |  | ↑ |  |
| *N*-linoleoyl alanine | BAL | ↓↓ |  | BAL | BAL | BAL | BAL |  |
| *N*-arachidonoyl alanine |  | ↓ | ↓ |  |  |  |  | ↓↓ |
| *N*-palmitoyl ethanolamine |  |  | ↑ | ↑ | ↑ |  | ↑↑ | ↑↑ |
| *N*-stearoyl ethanolamine |  |  | ↑↑↑↑ | ↑↑↑ |  |  | ↑↑↑↑ | ↑↑↑↑ |
| *N*-oleoyl ethanolamine |  | ↓ | ↑↑ |  | ↑ |  | ↑↑ | ↑↑ |
| *N*-linoleoyl ethanolamine | ↓ | ↓↓ |  |  |  | ↓↓ |  |  |
| *N*-arachidonoyl ethanolamine |  | ↓↓ |  |  |  |  |  |  |
| *N*-docosahexaenoyl ethanolamine | ↓ | ↓ |  |  |  | ↓ |  |  |
| *N*-palmitoyl GABA | ↓↓ | ↓↓ | ↓ | ↓↓ | ↓↓↓ | ↓ | ↓↓ | ↓↓ |
| *N*-stearoyl GABA | ↓ | ↓↓ | ↓↓ | ↓ | ↓↓ | ↓ | ↓↓ | ↓ |
| *N*-oleoyl GABA | ↓ | ↓↓ | ↓↓ | ↓↓ | ↓↓ |  | ↓↓ | ↓↓ |
| *N*-linoleoyl GABA | BAL | ↓↓ | ↓↓ | ↓↓ | ↓↓↓ | BAL | ↓↓↓ | ↓↓ |
| *N*-arachidonoyl GABA | ↓↓ | ↓↓ | ↓↓ | ↓↓ | ↓↓ |  | ↓↓ | ↓↓ |
| *N*-docosahexaenoyl GABA | ↓ | ↓↓ | ↓↓ | ↓↓ | ↓↓ |  | ↓↓ | ↓↓ |
| *N*-palmitoyl glycine |  | ↓ | ↓ |  | ↓ |  |  |  |
| *N*-stearoyl glycine |  | ↓ |  | ↓ | ↓ |  | ↓ | ↓ |
| *N*-oleoyl glycine | ↓ | ↓ |  | ↓ | ↓ | ↓ | ↓ |  |
| *N*-linoleoyl glycine | ↓ | ↓ |  | ↓ | ↓ |  | ↓ | ↓ |
| *N*-arachidonoyl glycine | ↓ | ↓ |  | ↓ | ↓ | ↓ | ↓ |  |
| *N*-docosahexaenoyl glycine |  | ↓ |  |  | ↓ | ↓ | ↓ |  |
| *N*-palmitoyl leucine |  | ↓ |  |  |  | ↓ |  |  |
| *N*-stearoyl leucine |  |  |  |  |  |  | ↓ | ↓ |
| *N*-oleoyl leucine |  | ↓ |  |  |  |  |  |  |
| *N*-linoleoyl leucine |  | ↓ | ↓↓ |  |  | BAL |  |  |
| *N*-docosahexaenoyl leucine |  |  | ↑ | ↓ |  |  |  |  |
| *N*-oleoyl methionine |  |  |  |  |  | BAL |  | ↓ |
| *N*-palmitoyl phenylalanine |  |  |  |  | ↓↓↓ |  |  |  |
| *N*-stearoyl phenylalanine |  |  |  |  | ↓↓ |  |  | ↑ |
| *N*-oleoyl phenylalanine |  |  |  |  | ↓↓ |  |  |  |
| *N*-arachidonoyl phenylalanine |  |  |  |  | ↓↓ |  |  |  |
| *N-*oleoyl proline | BAL | BAL | ↑ |  |  | BAL |  |  |
| *N*-palmitoyl serine |  |  | ↑ |  |  |  | ↑ | ↑ |
| *N*-stearoyl serine |  |  | ↑ |  |  |  | ↑ | ↑ |
| *N*-oleoyl serine |  |  |  |  |  |  | ↑ | ↑ |
| *N*-linoleoyl serine |  | ↓↓ |  |  | ↓ | BAL |  |  |
| *N*-arachidonoyl serine |  |  |  |  | ↓ |  |  |  |
| *N*-docosahexaenoyl serine | ↓ |  |  |  |  |  |  |  |
| *N*-palmitoyl taurine | ↓ | ↑ | ↓ |  | ↓ |  |  |  |
| *N*-stearoyl taurine | ↓ |  | ↓ |  |  |  |  | ↑ |
| *N-*oleoyl taurine |  |  |  | ↓ | ↓ | ↓ |  | ↑ |
| *N*-arachidonoyl taurine |  |  | ↓ |  | ↓ |  |  |  |
| *N*-palmitoyl tyrosine |  |  |  |  | ↓↓ |  |  |  |
| *N-*oleoyl tyrosine |  |  |  |  |  | BAL |  | ↑ |
| *N*-arachidonoyl tyrosine |  |  |  |  | ↓↓ | BAL |  |  |
| *N*-docosahexaenoyl tyrosine | BAL |  |  |  | ↓↓↓ | BAL |  | ↑ |
| *N*-palmitoyl valine |  |  | ↑ |  |  |  |  |  |
| *N*- stearoyl valine |  |  |  |  | ↓ |  |  |  |
| 2-palmitoyl glycerol | ↓ |  | ↑↑ |  | ↑ | ↓↓ | ↑ | ↑ |
| 2-oleoyl glycerol |  |  | ↑↑ | ↑ | ↑ |  | ↑ | ↑ |
| 2-linoleoyl glycerol |  | ↓ |  |  |  | ↓ |  |  |
| 2-arachidonoyl glycerol |  |  | ↑ |  |  |  |  |  |
| Oleic acid | ↓ | ↓ |  | ↓ |  | ↓ | ↓ | ↓ |
| Linoleic acid |  | ↓ |  | ↓ | ↓ | ↓ | ↓ | ↓ |
| Arachidonic acid |  | ↓ |  | ↓ |  |  | ↓ | ↓ |
| phosphoLEA |  |  | ↑ |  |  |  |  | ↑ |
| PGF_2α_ |  | ↑ |  |  |  |  |  |  |
| 6-ketoPGF_1α_ | ↑ |  |  |  | ↓ | BAL |  |  |

**Supplemental Figure 6: Effects of aging on CNS lipid levels in wild-type (WT) mice.** Cells with shaded arrows indicate a change for that lipid in the older adult WT brain area relative to the same young adult WT area. The arrow color indicates the direction of a significant result relative to young adults. Green colors represent increases, whereas orange colors represent decreases in a lipid’s concentration, with darker colors indicating a significant change of p < 0.05 and light colors representing a change of p < 0.10. The number of arrows indicates the magnitude of the difference between older and young adult WT. One arrow indicates a magnitude difference of less than 1.5 fold, 2 arrows indicate a 1.5-1.99 fold change, 3 arrows indicate a 2-2.99 fold change, and 4 arrows indicate a 3-9.99 fold change. BAL stands for “Below Analytical Limit,” whereas a blank cell indicates that there was no change in the lipid’s level due to age. Only lipids that were significantly affected by age in at least 1 brain area are shown here. See Methods and Supplemental Figure 2 for more detailed description of analysis. Brain regions analyzed were the striatum (STR), hippocampus (HIPP), cerebellum (CER), thalamus (THAL), cortex (CTX), hypothalamus (HYP), midbrain (MID), and brainstem (STEM).

**Supplemental Figure 7**

|  | **Change with old ABHD12 KO (relative to young ABHD12 KO)** | | | | | | | |
| --- | --- | --- | --- | --- | --- | --- | --- | --- |
| **Lipid Species** | STR | HIPP | CER | THAL | CTX | HYP | MID | STEM |
| *N*-palmitoyl alanine |  | ↓↓ |  |  | ↑ | ↓ |  | ↓ |
| *N*-stearoyl alanine |  | ↓↓ |  |  | ↑ |  |  |  |
| *N*-oleoyl alanine |  | ↓↓ |  |  |  |  |  | ↓ |
| *N*-linoleoyl alanine | BAL | ↓↓ |  | BAL | BAL | BAL | BAL | ↓ |
| *N*-arachidonoyl alanine | ↑ | ↓ |  |  |  |  |  | ↓↓↓ |
| *N*-docosahexaenoyl alanine | BAL | BAL | BAL | BAL |  | BAL | BAL | ↓↓ |
| *N*-palmitoyl ethanolamine | ↑ |  | ↑↑↑ | ↑ | ↑↑ |  | ↑↑ |  |
| *N*-stearoyl ethanolamine | ↑↑ | ↑↑ | ↑↑↑↑ | ↑↑↑ | ↑↑↑ |  | ↑↑↑↑ | ↑↑↑ |
| *N*-oleoyl ethanolamine | ↑ |  | ↑↑ |  | ↑↑ |  | ↑↑ |  |
| *N*-linoleoyl ethanolamine |  | ↓↓ |  | ↓ |  | ↓↓ | ↓ | ↓ |
| *N*-arachidonoyl ethanolamine |  | ↓↓ |  | ↓ |  | ↓ |  | ↓ |
| *N*-docosahexaenoyl ethanolamine |  | ↓↓ |  | ↓ |  | ↓ |  |  |
| *N*-palmitoyl GABA | ↓ | ↓↓ | ↓↓ | ↓↓ | ↓↓ |  | ↓↓ | ↓↓ |
| *N*-stearoyl GABA | ↓ | ↓↓ | ↓↓ | ↓ | ↓↓ |  | ↓↓ | ↓↓ |
| *N*-oleoyl GABA | ↓↓ | ↓↓ | ↓↓ | ↓↓ | ↓↓ |  | ↓↓ | ↓↓↓ |
| *N*-linoleoyl GABA | BAL | ↓↓↓ | ↓↓↓ | ↓↓ | ↓↓ | BAL | ↓↓↓ | ↓↓ |
| *N*-arachidonoyl GABA | ↓ | ↓↓ | ↓↓ | ↓↓ | ↓↓ |  | ↓↓ | ↓↓ |
| *N*-docosahexaenoyl GABA | ↓ | ↓↓ | ↓↓↓ | ↓↓ | ↓↓ |  | ↓↓ | ↓↓ |
| *N*-palmitoyl glycine |  |  | ↓ |  | ↓ |  |  | ↓ |
| *N*-stearoyl glycine |  | ↓ |  | ↓ | ↓ |  | ↓ | ↓ |
| *N*-oleoyl glycine |  | ↓ |  | ↓ | ↓ |  | ↓ | ↓ |
| *N*-linoleoyl glycine |  | ↓ |  | ↓ | ↓ |  |  | ↓ |
| *N*-arachidonoyl glycine |  | ↓ |  | ↓ | ↓ | ↓ | ↓ | ↓ |
| *N*-docosahexaenoyl glycine |  | ↓ |  | ↓ | ↓ |  | ↓ | ↓ |
| *N*-palmitoyl leucine |  | ↓ |  |  |  |  |  |  |
| *N*-stearoyl leucine |  | ↓ | ↓ |  |  | ↓ |  |  |
| *N*-oleoyl leucine |  |  |  |  |  |  |  | ↓ |
| *N*-linoleoyl leucine |  | ↓↓ |  |  |  | BAL | ↓↓ |  |
| *N*-docosahexaenoyl leucine |  | ↓ |  |  |  | ↓ | ↓↓ |  |
| *N*-stearoyl methionine | ↓ | ↓ |  | ↓ |  | BAL |  |  |
| *N*-oleoyl methionine |  |  |  |  |  | BAL |  | ↓↓ |
| *N*-palmitoyl phenylalanine | ↓ | ↓ | ↓ | ↓ | ↓↓↓ | ↓ | ↓↓ |  |
| *N*-stearoyl phenylalanine | ↓ | ↓ | ↓ | ↓ | ↓↓ | ↓ | ↓ |  |
| *N*-oleoyl phenylalanine |  | ↓ | ↓ | ↓ | ↓↓↓ |  | ↓ |  |
| *N*-linoleoyl phenylalanine | BAL | BAL |  | BAL | ↓↓ | BAL |  | BAL |
| *N*-arachidonoyl phenylalanine | ↓ |  | ↓↓ |  | ↓↓ | ↓↓ | ↓ |  |
| *N*-docosahexaenoyl phenylalanine | ↓ | ↓ |  |  | ↓↓ | BAL |  |  |
| *N*-palmitoyl proline |  |  |  |  | ↑ |  |  |  |
| *N*-stearoyl proline |  | ↓ |  |  |  | ↓ |  |  |
| *N-*oleoyl proline | BAL | BAL |  |  |  | BAL | ↑ |  |
| *N*-palmitoyl serine | ↑ | ↑ | ↑ |  |  |  | ↑ | ↑ |
| *N*-stearoyl serine |  |  | ↑ |  |  |  | ↑ | ↑ |
| *N*-oleoyl serine |  | ↑ | ↑ | ↑ |  |  | ↑ |  |
| *N*-linoleoyl serine |  | ↓ |  |  | ↓ | BAL |  | ↓ |
| *N*-arachidonoyl serine |  |  |  |  | ↓ |  | ↑ |  |
| *N*-docosahexaenoyl serine |  |  |  |  | ↓ |  | ↑ |  |
| *N*-palmitoyl taurine |  |  |  | ↑ |  |  |  | ↑ |
| *N*-stearoyl taurine |  | ↑ |  |  |  | ↑ |  | ↑ |
| *N*-arachidonoyl taurine |  |  |  |  |  | ↑ |  |  |
| *N*-palmitoyl tryptophan |  |  |  | ↓↓ |  | BAL |  | ↓↓ |
| *N*-stearoyl tryptophan |  |  | ↓ |  |  |  |  | ↓ |
| *N*-oleoyl tryptophan | BAL | BAL |  | BAL |  | BAL | BAL | ↓ |
| *N*-palmitoyl tyrosine |  | ↓ | ↓ | ↓ | ↓↓ |  | ↓ | ↓ |
| *N*-stearoyl tyrosine | ↓ | ↓ | ↓ | ↓ | ↓↓ | BAL |  |  |
| *N-*oleoyl tyrosine | ↓ |  | ↓↓ | ↓ | ↓↓↓ | BAL | ↓ | ↓↓ |
| *N*-arachidonoyl tyrosine | ↓ | ↓ | ↓↓ |  | ↓↓ | BAL | ↓ | ↓ |
| *N*-docosahexaenoyl tyrosine | BAL | ↓ |  |  | ↓↓ | BAL |  | ↓ |
| *N*-palmitoyl valine |  |  |  |  | ↓ |  | ↓ | ↓ |
| *N*- stearoyl valine |  |  |  |  |  |  |  | ↓ |
| 2-palmitoyl glycerol | ↓ |  | ↑↑ |  | ↑↑ | ↓↓ |  |  |
| 2-oleoyl glycerol |  |  | ↑↑ |  | ↑↑ | ↓↓ | ↑ |  |
| 2-linoleoyl glycerol | ↓ | ↓ |  | ↓ |  | ↓↓ | ↓ | ↓ |
| 2-arachidonoyl glycerol |  | ↓ | ↑ |  |  | ↓↓ |  |  |
| Oleic acid | ↓ | ↓ |  | ↓↓ | ↓ | ↓ | ↓ | ↓ |
| Linoleic acid |  | ↓ | ↓ | ↓ |  | ↓ | ↓ | ↓↓ |
| Arachidonic acid | ↓ | ↓ | ↓ | ↓ |  | ↓ | ↓ | ↓ |
| phosphoLEA |  |  | ↑ |  |  |  |  |  |
| PGE_2_ |  |  |  |  |  |  | ↓ |  |
| PGF_2α_ | ↓ | ↓ |  |  |  |  | ↓ |  |
| 6-ketoPGF_1α_ | ↑↑ | ↓ |  | ↓ | ↓ | BAL |  | ↓ |

**Supplemental Figure 7: Effects of aging on CNS lipid levels in ABHD12 knockout (KO) mice.** Cells with shaded arrows indicate a change for that lipid in the older adult ABHD12 KO brain area relative to the same young adult ABHD12 KO area. The arrow color indicates the direction of a significant result relative to young adults. Green colors represent increases, whereas orange colors represent decreases in a lipid’s concentration, with darker colors indicating a significant change of p < 0.05 and light colors representing a change of p < 0.10. The number of arrows indicates the magnitude of the difference between older and young adult. One arrow indicates a magnitude difference of less than 1.5 fold, 2 arrows indicate a 1.5-1.99 fold change, 3 arrows indicate a 2-2.99 fold change, and 4 arrows indicate a 3-9.99 fold change. BAL stands for “Below Analytical Limit,” whereas a blank cell indicates that there was no change in the lipid’s level due to age. Only lipids that were significantly affected by age in at least 1 brain area are shown here. See Methods and Supplemental Figure 2 for more detailed description of analysis. Brain areas analyzed were the striatum (STR), hippocampus (HIPP), cerebellum (CER), thalamus (THAL), cortex (CTX), hypothalamus (HYP), midbrain (MID), and brainstem (STEM).

**Supplemental Figure 8**

|  | **Change with old (relative to young)** | | | | | | | |
| --- | --- | --- | --- | --- | --- | --- | --- | --- |
| **Lipid Species** | STR | HIPP | CER | THAL | CTX | HYP | MID | STEM |
| *N*-palmitoyl alanine |  | ↓↓ |  |  |  | ↓ |  | ↓ |
| *N*-stearoyl alanine |  | ↓↓ |  |  | ↑ |  |  | ↓ |
| *N*-oleoyl alanine |  | ↓↓ |  |  |  |  |  | ↓ |
| *N*-linoleoyl alanine | BAL | ↓↓ |  | BAL | BAL | BAL | BAL |  |
| *N*-arachidonoyl alanine | ↑ | ↓ |  |  |  |  |  | ↓↓ |
| *N*-docosahexaenoyl alanine | BAL | BAL | BAL | BAL |  | BAL | BAL | ↓ |
| *N*-palmitoyl ethanolamine |  |  | ↑↑ | ↑ | ↑ | ↓ | ↑↑ | ↑ |
| *N*-stearoyl ethanolamine |  | ↑↑ | ↑↑↑ | ↑↑↑ | ↑↑ |  | ↑↑↑↑ | ↑↑↑ |
| *N*-oleoyl ethanolamine |  | ↓ | ↑↑ | ↑ | ↑ | ↓ | ↑↑ | ↑ |
| *N*-linoleoyl ethanolamine | ↓ | ↓↓ |  | ↓ |  | ↓↓ | ↓ | ↓ |
| *N*-arachidonoyl ethanolamine |  | ↓↓ |  | ↓ |  | ↓ |  |  |
| *N*-docosahexaenoyl ethanolamine | ↓ | ↓↓ |  | ↓ |  | ↓ |  |  |
| *N*-palmitoyl GABA | ↓ | ↓↓ | ↓↓ | ↓↓ | ↓↓↓ | ↓ | ↓↓ | ↓↓ |
| *N*-stearoyl GABA | ↓ | ↓↓ | ↓↓ | ↓ | ↓↓ | ↓ | ↓↓ | ↓ |
| *N*-oleoyl GABA | ↓ | ↓↓ | ↓↓ | ↓↓ | ↓↓ |  | ↓↓ | ↓↓ |
| *N*-linoleoyl GABA | BAL | ↓↓ | ↓↓↓ | ↓↓ | ↓↓↓ | BAL | ↓↓↓ | ↓↓ |
| *N*-arachidonoyl GABA | ↓ | ↓↓ | ↓↓ | ↓↓ | ↓↓ | ↓ | ↓↓ | ↓↓ |
| *N*-docosahexaenoyl GABA | ↓ | ↓↓ | ↓↓ | ↓↓ | ↓↓ | ↓ | ↓↓ | ↓↓ |
| *N*-palmitoyl glycine |  | ↓ | ↓ |  | ↓ | ↓ |  | ↓ |
| *N*-stearoyl glycine | ↓ | ↓ | ↓ | ↓ | ↓ |  | ↓ | ↓ |
| *N*-oleoyl glycine | ↓ | ↓ | ↓ | ↓ | ↓ | ↓ | ↓ | ↓ |
| *N*-linoleoyl glycine | ↓ | ↓ |  | ↓ | ↓ | ↓ | ↓ | ↓ |
| *N*-arachidonoyl glycine | ↓ | ↓ |  | ↓ | ↓ | ↓ | ↓ | ↓ |
| *N*-docosahexaenoyl glycine |  | ↓ |  | ↓ | ↓ | ↓ | ↓ | ↓ |
| *N*-palmitoyl leucine |  | ↓ |  |  |  | ↓ |  |  |
| *N*-stearoyl leucine |  | ↓ |  |  |  | ↓ | ↓ | ↓ |
| *N*-oleoyl leucine |  | ↓ |  |  |  |  |  | ↓ |
| *N*-linoleoyl leucine |  | ↓↓ | ↓↓ |  |  | BAL | ↓ |  |
| *N*-docosahexaenoyl leucine |  |  |  | ↓ |  | ↓ | ↓ |  |
| *N*-stearoyl methionine |  | ↓ |  | ↓ |  |  |  | ↓ |
| *N*-oleoyl methionine |  |  |  |  |  | BAL |  | ↓↓ |
| *N*-palmitoyl phenylalanine |  | ↓ | ↓ |  | ↓↓↓ | ↓ | ↓ |  |
| *N*-stearoyl phenylalanine | ↓ |  | ↓ |  | ↓↓ | ↓ | ↓ | ↑ |
| *N*-oleoyl phenylalanine |  | ↓ |  |  | ↓↓↓ |  | ↓ | ↑ |
| *N*-linoleoyl phenylalanine | BAL | BAL |  | BAL | ↓↓ | BAL |  | BAL |
| *N*-arachidonoyl phenylalanine |  |  | ↓ |  | ↓↓ | ↓ |  |  |
| *N*-docosahexaenoyl phenylalanine | ↓ | ↓ |  |  | ↓ | BAL |  |  |
| *N*-stearoyl proline |  |  | ↑ |  |  | ↓ |  |  |
| *N-*oleoyl proline | BAL | BAL | ↑ |  |  | BAL |  |  |
| *N*-palmitoyl serine |  | ↑ | ↑ |  |  |  | ↑ | ↑ |
| *N*-stearoyl serine |  |  | ↑ |  |  |  | ↑ | ↑ |
| *N*-oleoyl serine |  | ↑ | ↑ | ↑ |  |  | ↑ | ↑ |
| *N*-linoleoyl serine |  | ↓ |  |  | ↓ | BAL |  | ↓ |
| *N*-arachidonoyl serine |  | ↓ |  |  | ↓ |  | ↑ |  |
| *N*-docosahexaenoyl serine |  |  |  |  | ↓ | ↓ | ↑ |  |
| *N*-palmitoyl taurine |  | ↑ | ↓ |  | ↓ |  |  | ↑ |
| *N*-stearoyl taurine | ↓ | ↑ | ↓ |  |  |  |  | ↑ |
| *N-*oleoyl taurine |  |  |  | ↓ |  | ↓ |  |  |
| *N*-arachidonoyl taurine |  |  | ↓ |  | ↓ |  |  |  |
| *N*-palmitoyl tyrosine |  |  |  |  | ↓↓ |  |  |  |
| *N*-stearoyl tyrosine | ↓ | ↓ | ↓ | ↓ | ↓ | BAL |  |  |
| *N-*oleoyl tyrosine |  |  |  |  | ↓↓ | BAL |  |  |
| *N*-arachidonoyl tyrosine | ↓ | ↓ | ↓ |  | ↓↓ | BAL | ↓ | ↓ |
| *N*-docosahexaenoyl tyrosine | BAL |  |  |  | ↓↓ | BAL |  |  |
| *N*-palmitoyl valine |  |  |  |  |  |  |  | ↓ |
| *N*- stearoyl valine |  |  |  |  | ↓ |  |  |  |
| 2-palmitoyl glycerol | ↓ |  | ↑↑ |  | ↑ | ↓↓ | ↑ |  |
| 2-oleoyl glycerol |  |  | ↑↑ | ↑ | ↑ | ↓ | ↑ | ↑ |
| 2-linoleoyl glycerol | ↓ | ↓ |  | ↓ |  | ↓↓ | ↓ | ↓ |
| 2-arachidonoyl glycerol | ↓ |  | ↑ |  |  | ↓ |  |  |
| Oleic acid | ↓ | ↓ |  | ↓ | ↓ | ↓ | ↓ | ↓ |
| Linoleic acid | ↓ | ↓ | ↓ | ↓ | ↓ | ↓ | ↓ | ↓ |
| Arachidonic acid | ↓ | ↓ | ↓ | ↓ | ↓ | ↓ | ↓ | ↓ |
| phosphoLEA |  |  | ↑ |  |  |  |  |  |
| PGE_2_ |  |  |  |  |  |  | ↓ |  |
| PGF_2α_ | ↓ |  |  |  |  |  | ↓ |  |
| 6-ketoPGF_1α_ | ↑↑ | ↓ |  |  | ↓ | BAL |  | ↓ |

**Supplemental Figure 8: Changes in CNS lipid levels in older adult mice with wild-type (WT) and ABHD12 knockout (KO) combined compared to younger adult mice with WT and ABHD12 KO combined.** Cells with shaded arrows indicate a change for that lipid in the older adult brain area relative to the same young adult area. The arrow color indicates the direction of a significant result relative to young adults. Green colors represent increases, whereas orange colors represent decreases in a lipid’s concentration, with darker colors indicating a significant change of p < 0.05 and light colors representing a change of p < 0.10. The number of arrows indicates the magnitude of the difference between older and young adult. One arrow indicates a magnitude difference of less than 1.5 fold, 2 arrows indicate a 1.5-1.99 fold change, 3 arrows indicate a 2-2.99 fold change, and 4 arrows indicate a 3-9.99 fold change. BAL stands for “Below Analytical Limit,” whereas a blank cell indicates that there was no change in the lipid’s level due to age. Only lipids that were significantly affected by age in at least 1 brain area are shown here. See Methods and Supplemental Figure 2 for more detailed description of analysis. Brain regions analyzed were the striatum (STR), hippocampus (HIPP), cerebellum (CER), thalamus (THAL), cortex (CTX), hypothalamus (HYP), midbrain (MID), and brainstem (STEM).

**1.2 Supplemental Tables**

Supplemental Tables 1-69 display the mean levels of each lipid along with the standard deviation (SD) and standard error (SE) in brain areas from the following groups: younger adult wild-type mice (WT young), older adult wild-type mice (WT old), the combination of younger and older adult wild-type mice (WT), younger adult ABHD12 knockout mice (KO young), older adult ABHD12 knockout mice (KO old), the combination of younger and older adult ABHD12 knockout mice (ABHD12 KO), the combination of younger adult WT and ABHD12 knockout mice (Young), and the combination of older adult WT and ABHD12 knockout mice (Old). PISSR stands for “Present in Some Samples at Random,” and means that that particular lipid was not detected in all samples in that group and was not quantified for analysis, whereas BDL stands for “Below Detection Limit” and means that that particular lipid was not found in any samples from that group.

Supplemental Tables 70-77 are the outputs from ANOVAs testing the effect of genotype, age, and a genotype by age interaction on mean levels of each detected lipid in each brain area. Only lipids that were detected in all samples of each group in that brain area underwent statistical analysis. The F and p values are listed for each ANOVA and differences with p < 0.10 are in bold text. The effect of genotype compared WT and ABHD12 KO, combining older and younger adult mice within a genotype, yielding an n of 12 for WT (11 in hypothalamus) and an n of 15 for ABHD12 KO (13 in hypothalamus). In the column labeled “mag”, the magnitude of change for that lipid in the KO brain area relative to WT is listed for changes with p < 0.10. The effect of age compared younger and older adult mice, combining WT and ABHD12 KO adult mice within an age group, yielding an n of 12 for younger adults and an n of 15 for older adults (12 in hypothalamus). In the column labeled “mag”, the magnitude of change for that lipid in the older adult brain area relative to the young adult level is listed for changes with p < 0.10. The interaction tested for a main effect of group for each detected lipid in each brain area using the following groups: young adult WT (n=6), older adult WT (n=6; n=5 for hypothalamus), young adult ABHD12 KO (n=6), and older adult ABHD12 KO (n=9, n=7 for hypothalamus).

Supplemental Tables 78-109 are the outputs from the post-hoc LSD tests comparing mean levels of lipids detected in all samples in that brain area between groups. There were 4 groups of mice: young adult WT (n=6), older adult WT (n=6; n=5 for hypothalamus), young adult ABHD12 KO (n=6), and older adult ABHD12 KO (n=9, n=7 for hypothalamus). Only lipids that differed between groups at p < 0.10 are listed. For each brain area, data is first presented for the comparison between young ABHD12 KO mice and young WT mice, then for the comparison between old ABHD12 KO mice and old WT mice, then for the comparison between old WT mice and young WT mice, and finally for the comparison between old ABHD12 KO mice and young ABHD12 KO mice. The direction and magnitude of the differences between groups is also shown. The arrow color indicates the direction of a significant result. Green colors represent increases, whereas orange colors represent decreases in a lipid’s concentration, with darker colors indicating a significant change of p < 0.05 and light colors representing a change of p < 0.10. The number of arrows indicates the magnitude of the difference between groups. One arrow indicates a magnitude difference of less than 1.5 fold, 2 arrows indicate a 1.5-1.99 fold change, 3 arrows indicate a 2-2.99 fold change, and 4 arrows indicate a 3-9.99 fold change.

Supplemental Table 1: Mean levels of *N*-acyl alanines in the striatum and hippocampus

| Lipid Species | Group | Striatum | | | | Hippocampus | | | |
| --- | --- | --- | --- | --- | --- | --- | --- | --- | --- |
|  |  | N | Mean | SD | SE | N | Mean | SD | SE |
| *N*-palmitoyl alanine | WT young | 6 | 1.17E-11 | 4.34E-13 | 1.77E-13 | 6 | 1.30E-11 | 3.05E-12 | 1.24E-12 |
|  | WT old | 6 | 1.30E-11 | 4.80E-12 | 1.96E-12 | 6 | 7.12E-12 | 4.90E-13 | 2.00E-13 |
|  | WT | 12 | 1.23E-11 | 3.32E-12 | 9.58E-13 | 12 | 1.01E-11 | 3.72E-12 | 1.07E-12 |
|  | KO young | 6 | 1.15E-11 | 1.81E-12 | 7.41E-13 | 6 | 1.35E-11 | 3.11E-12 | 1.27E-12 |
|  | KO old | 9 | 1.25E-11 | 1.89E-12 | 6.30E-13 | 9 | 7.32E-12 | 1.87E-12 | 6.23E-13 |
|  | ABHD12 KO | 15 | 1.21E-11 | 1.86E-12 | 4.81E-13 | 15 | 9.78E-12 | 3.89E-12 | 1.01E-12 |
|  | Young | 12 | 1.16E-11 | 1.26E-12 | 3.64E-13 | 12 | 1.32E-11 | 2.95E-12 | 8.51E-13 |
|  | Old | 15 | 1.27E-11 | 3.21E-12 | 8.30E-13 | 15 | 7.24E-12 | 1.45E-12 | 3.74E-13 |
| *N*-stearoyl alanine | WT young | 6 | 1.46E-11 | 1.24E-12 | 5.08E-13 | 6 | 1.51E-11 | 2.32E-12 | 9.46E-13 |
|  | WT old | 6 | 1.43E-11 | 3.03E-12 | 1.24E-12 | 6 | 9.94E-12 | 9.53E-13 | 3.89E-13 |
|  | WT | 12 | 1.44E-11 | 2.21E-12 | 6.39E-13 | 12 | 1.25E-11 | 3.18E-12 | 9.19E-13 |
|  | KO young | 6 | 1.42E-11 | 1.77E-12 | 7.21E-13 | 6 | 1.62E-11 | 2.63E-12 | 1.07E-12 |
|  | KO old | 9 | 1.42E-11 | 1.89E-12 | 6.30E-13 | 9 | 1.06E-11 | 2.21E-12 | 7.38E-13 |
|  | ABHD12 KO | 15 | 1.42E-11 | 1.78E-12 | 4.59E-13 | 15 | 1.28E-11 | 3.66E-12 | 9.46E-13 |
|  | Young | 12 | 1.44E-11 | 1.47E-12 | 4.25E-13 | 12 | 1.57E-11 | 2.43E-12 | 7.03E-13 |
|  | Old | 15 | 1.42E-11 | 2.31E-12 | 5.95E-13 | 15 | 1.03E-11 | 1.80E-12 | 4.64E-13 |
| *N*-oleoyl alanine | WT young | 6 | 8.08E-12 | 5.98E-13 | 2.44E-13 | 6 | 9.62E-12 | 1.08E-12 | 4.40E-13 |
|  | WT old | 6 | 9.68E-12 | 1.83E-12 | 7.47E-13 | 6 | 6.58E-12 | 3.30E-13 | 1.35E-13 |
|  | WT | 12 | 8.88E-12 | 1.54E-12 | 4.46E-13 | 12 | 8.10E-12 | 1.76E-12 | 5.08E-13 |
|  | KO young | 6 | 8.11E-12 | 1.64E-12 | 6.70E-13 | 6 | 9.47E-12 | 1.86E-12 | 7.59E-13 |
|  | KO old | 9 | 7.88E-12 | 8.46E-13 | 2.82E-13 | 9 | 5.95E-12 | 9.22E-13 | 3.07E-13 |
|  | ABHD12 KO | 15 | 7.97E-12 | 1.18E-12 | 3.04E-13 | 15 | 7.36E-12 | 2.22E-12 | 5.72E-13 |
|  | Young | 12 | 8.09E-12 | 1.18E-12 | 3.40E-13 | 12 | 9.55E-12 | 1.45E-12 | 4.19E-13 |
|  | Old | 15 | 8.60E-12 | 1.56E-12 | 4.03E-13 | 15 | 6.20E-12 | 7.93E-13 | 2.05E-13 |
| *N*-linoleoyl alanine | WT young | 6 | PISSR |  |  | 6 | 2.28E-12 | 5.81E-13 | 2.37E-13 |
|  | WT old | 6 | PISSR |  |  | 6 | 1.43E-12 | 4.52E-13 | 1.84E-13 |
|  | WT | 12 | PISSR |  |  | 12 | 1.86E-12 | 6.67E-13 | 1.93E-13 |
|  | KO young | 6 | PISSR |  |  | 6 | 2.18E-12 | 7.40E-13 | 3.02E-13 |
|  | KO old | 9 | PISSR |  |  | 9 | 1.30E-12 | 3.50E-13 | 1.17E-13 |
|  | ABHD12 KO | 15 | PISSR |  |  | 15 | 1.65E-12 | 6.83E-13 | 1.76E-13 |
|  | Young | 12 | PISSR |  |  | 12 | 2.23E-12 | 6.37E-13 | 1.84E-13 |
|  | Old | 15 | PISSR |  |  | 15 | 1.35E-12 | 3.84E-13 | 9.91E-14 |
| *N*-arachidonoyl alanine | WT young | 6 | 6.26E-12 | 6.04E-13 | 2.47E-13 | 6 | 1.17E-11 | 1.63E-12 | 6.66E-13 |
|  | WT old | 6 | 6.98E-12 | 2.12E-12 | 8.66E-13 | 6 | 7.83E-12 | 4.02E-13 | 1.64E-13 |
|  | WT | 12 | 6.62E-12 | 1.53E-12 | 4.42E-13 | 12 | 9.77E-12 | 2.32E-12 | 6.69E-13 |
|  | KO young | 6 | 8.07E-12 | 1.67E-12 | 6.82E-13 | 6 | 1.25E-11 | 1.63E-12 | 6.67E-13 |
|  | KO old | 9 | 9.38E-12 | 1.05E-12 | 3.51E-13 | 9 | 8.84E-12 | 9.73E-13 | 3.24E-13 |
|  | ABHD12 KO | 15 | 8.86E-12 | 1.44E-12 | 3.72E-13 | 15 | 1.03E-11 | 2.22E-12 | 5.74E-13 |
|  | Young | 12 | 7.17E-12 | 1.53E-12 | 4.40E-13 | 12 | 1.21E-11 | 1.61E-12 | 4.65E-13 |
|  | Old | 15 | 8.42E-12 | 1.93E-12 | 4.98E-13 | 15 | 8.44E-12 | 9.28E-13 | 2.40E-13 |
| *N*-docosahexaenoyl alanine | WT young | 6 | PISSR |  |  | 6 | PISSR |  |  |
|  | WT old | 6 | PISSR |  |  | 6 | PISSR |  |  |
|  | WT | 12 | PISSR |  |  | 12 | PISSR |  |  |
|  | KO young | 6 | PISSR |  |  | 6 | PISSR |  |  |
|  | KO old | 9 | PISSR |  |  | 9 | PISSR |  |  |
|  | ABHD12 KO | 15 | PISSR |  |  | 15 | PISSR |  |  |
|  | Young | 12 | PISSR |  |  | 12 | PISSR |  |  |
|  | Old | 15 | PISSR |  |  | 15 | PISSR |  |  |

Supplemental Table 2: Mean levels of *N*-acyl alanines in the cerebellum and thalamus

| Lipid Species | Group | Cerebellum | | | | Thalamus | | | |
| --- | --- | --- | --- | --- | --- | --- | --- | --- | --- |
|  |  | N | Mean | SD | SE | N | Mean | SD | SE |
| *N*-palmitoyl alanine | WT young | 6 | 9.66E-12 | 1.15E-12 | 4.69E-13 | 6 | 6.83E-12 | 5.77E-13 | 2.36E-13 |
|  | WT old | 6 | 1.01E-11 | 2.03E-12 | 8.3E-13 | 6 | 6.42E-12 | 8.46E-13 | 3.46E-13 |
|  | WT | 12 | 9.87E-12 | 1.59E-12 | 4.59E-13 | 12 | 6.62E-12 | 7.24E-13 | 2.09E-13 |
|  | KO young | 6 | 9.62E-12 | 1.58E-12 | 6.43E-13 | 6 | 7.55E-12 | 1.27E-12 | 5.17E-13 |
|  | KO old | 9 | 9.55E-12 | 1.34E-12 | 4.48E-13 | 9 | 7.54E-12 | 2.07E-12 | 6.89E-13 |
|  | ABHD12 KO | 15 | 9.58E-12 | 1.39E-12 | 3.58E-13 | 15 | 7.54E-12 | 1.74E-12 | 4.48E-13 |
|  | Young | 12 | 9.64E-12 | 1.31E-12 | 3.79E-13 | 12 | 7.19E-12 | 1.01E-12 | 2.92E-13 |
|  | Old | 15 | 9.77E-12 | 1.61E-12 | 4.15E-13 | 15 | 7.09E-12 | 1.74E-12 | 4.49E-13 |
| *N*-stearoyl alanine | WT young | 6 | 2.19E-11 | 5.09E-12 | 2.08E-12 | 6 | 9.10E-12 | 9.98E-13 | 4.07E-13 |
|  | WT old | 6 | 2.31E-11 | 3.55E-12 | 1.45E-12 | 6 | 8.27E-12 | 1.14E-12 | 4.66E-13 |
|  | WT | 12 | 2.25E-11 | 4.23E-12 | 1.22E-12 | 12 | 8.68E-12 | 1.11E-12 | 3.21E-13 |
|  | KO young | 6 | 2.26E-11 | 4.69E-12 | 1.91E-12 | 6 | 1.03E-11 | 1.45E-12 | 5.92E-13 |
|  | KO old | 9 | 2.27E-11 | 3.59E-12 | 1.2E-12 | 9 | 1.05E-11 | 2.41E-12 | 8.02E-13 |
|  | ABHD12 KO | 15 | 2.27E-11 | 3.90E-12 | 1.01E-12 | 15 | 1.04E-11 | 2.02E-12 | 5.21E-13 |
|  | Young | 12 | 2.23E-11 | 4.68E-12 | 1.35E-12 | 12 | 9.69E-12 | 1.34E-12 | 3.86E-13 |
|  | Old | 15 | 2.29E-11 | 3.45E-12 | 8.9E-13 | 15 | 9.62E-12 | 2.25E-12 | 5.82E-13 |
| *N*-oleoyl alanine | WT young | 6 | 9.47E-12 | 1.42E-12 | 5.78E-13 | 6 | 5.12E-12 | 4.00E-13 | 1.63E-13 |
|  | WT old | 6 | 1.20E-11 | 2.78E-12 | 1.13E-12 | 6 | 5.64E-12 | 1.09E-12 | 4.43E-13 |
|  | WT | 12 | 1.07E-11 | 2.48E-12 | 7.16E-13 | 12 | 5.38E-12 | 8.27E-13 | 2.39E-13 |
|  | KO young | 6 | 8.68E-12 | 1.37E-12 | 5.61E-13 | 6 | 5.58E-12 | 7.15E-13 | 2.92E-13 |
|  | KO old | 9 | 8.51E-12 | 1.90E-12 | 6.34E-13 | 9 | 5.30E-12 | 7.92E-13 | 2.64E-13 |
|  | ABHD12 KO | 15 | 8.58E-12 | 1.66E-12 | 4.28E-13 | 15 | 5.41E-12 | 7.49E-13 | 1.93E-13 |
|  | Young | 12 | 9.07E-12 | 1.39E-12 | 4.02E-13 | 12 | 5.35E-12 | 6.04E-13 | 1.74E-13 |
|  | Old | 15 | 9.90E-12 | 2.82E-12 | 7.28E-13 | 15 | 5.44E-12 | 8.99E-13 | 2.32E-13 |
| *N*-linoleoyl alanine | WT young | 6 | 1.62E-12 | 9.14E-13 | 3.73E-13 | 6 | PISSR |  |  |
|  | WT old | 6 | 1.50E-12 | 5.63E-13 | 2.3E-13 | 6 | PISSR |  |  |
|  | WT | 12 | 1.56E-12 | 7.27E-13 | 2.1E-13 | 12 | PISSR |  |  |
|  | KO young | 6 | 1.70E-12 | 3.58E-13 | 1.46E-13 | 6 | PISSR |  |  |
|  | KO old | 9 | 1.36E-12 | 4.18E-13 | 1.39E-13 | 9 | PISSR |  |  |
|  | ABHD12 KO | 15 | 1.50E-12 | 4.19E-13 | 1.08E-13 | 15 | PISSR |  |  |
|  | Young | 12 | 1.66E-12 | 6.63E-13 | 1.91E-13 | 12 | PISSR |  |  |
|  | Old | 15 | 1.42E-12 | 4.66E-13 | 1.2E-13 | 15 | PISSR |  |  |
| *N*-arachidonoyl alanine | WT young | 6 | 4.29E-12 | 6.97E-13 | 2.85E-13 | 6 | 3.41E-12 | 6.31E-13 | 2.58E-13 |
|  | WT old | 6 | 3.57E-12 | 4.27E-13 | 1.74E-13 | 6 | 3.57E-12 | 1.04E-12 | 4.25E-13 |
|  | WT | 12 | 3.93E-12 | 6.70E-13 | 1.93E-13 | 12 | 3.49E-12 | 8.25E-13 | 2.38E-13 |
|  | KO young | 6 | 5.06E-12 | 5.96E-13 | 2.43E-13 | 6 | 4.65E-12 | 1.60E-12 | 6.51E-13 |
|  | KO old | 9 | 5.52E-12 | 8.67E-13 | 2.89E-13 | 9 | 4.77E-12 | 8.24E-13 | 2.75E-13 |
|  | ABHD12 KO | 15 | 5.34E-12 | 7.82E-13 | 2.02E-13 | 15 | 4.72E-12 | 1.14E-12 | 2.95E-13 |
|  | Young | 12 | 4.68E-12 | 7.37E-13 | 2.13E-13 | 12 | 4.03E-12 | 1.33E-12 | 3.83E-13 |
|  | Old | 15 | 4.74E-12 | 1.22E-12 | 3.14E-13 | 15 | 4.29E-12 | 1.07E-12 | 2.77E-13 |
| *N*-docosahexaenoyl alanine | WT young | 6 | PISSR |  |  | 6 | PISSR |  |  |
|  | WT old | 6 | PISSR |  |  | 6 | PISSR |  |  |
|  | WT | 12 | PISSR |  |  | 12 | PISSR |  |  |
|  | KO young | 6 | PISSR |  |  | 6 | PISSR |  |  |
|  | KO old | 9 | PISSR |  |  | 9 | PISSR |  |  |
|  | ABHD12 KO | 15 | PISSR |  |  | 15 | PISSR |  |  |
|  | Young | 12 | PISSR |  |  | 12 | PISSR |  |  |
|  | Old | 15 | PISSR |  |  | 15 | PISSR |  |  |

Supplemental Table 3: Mean levels of *N*-acyl alanines in the cortex and hypothalamus

| Lipid Species | Group | Cortex | | | | Hypothalamus | | | |
| --- | --- | --- | --- | --- | --- | --- | --- | --- | --- |
|  |  | N | Mean | SD | SE | N | Mean | SD | SE |
| *N*-palmitoyl alanine | WT young | 6 | 3.21E-12 | 4.96E-13 | 2.03E-13 | 6 | 1.69E-11 | 2.54E-12 | 1.04E-12 |
|  | WT old | 6 | 3.19E-12 | 4.30E-13 | 1.75E-13 | 5 | 1.41E-11 | 3.09E-12 | 1.38E-12 |
|  | WT | 12 | 3.20E-12 | 4.43E-13 | 1.28E-13 | 11 | 1.56E-11 | 3.05E-12 | 9.20E-13 |
|  | KO young | 6 | 3.10E-12 | 3.50E-13 | 1.43E-13 | 6 | 1.57E-11 | 8.23E-13 | 3.36E-13 |
|  | KO old | 9 | 3.55E-12 | 4.03E-13 | 1.34E-13 | 7 | 1.29E-11 | 1.66E-12 | 6.27E-13 |
|  | ABHD12 KO | 15 | 3.37E-12 | 4.34E-13 | 1.12E-13 | 13 | 1.42E-11 | 1.96E-12 | 5.44E-13 |
|  | Young | 12 | 3.15E-12 | 4.14E-13 | 1.19E-13 | 12 | 1.63E-11 | 1.91E-12 | 5.52E-13 |
|  | Old | 15 | 3.40E-12 | 4.38E-13 | 1.13E-13 | 12 | 1.34E-11 | 2.31E-12 | 6.67E-13 |
| *N*-stearoyl alanine | WT young | 6 | 8.01E-12 | 2.81E-13 | 1.15E-13 | 6 | 1.72E-11 | 3.16E-12 | 1.29E-12 |
|  | WT old | 6 | 8.23E-12 | 4.33E-13 | 1.77E-13 | 5 | 1.45E-11 | 3.54E-12 | 1.58E-12 |
|  | WT | 12 | 8.12E-12 | 3.67E-13 | 1.06E-13 | 11 | 1.60E-11 | 3.47E-12 | 1.05E-12 |
|  | KO young | 6 | 8.57E-12 | 5.57E-13 | 2.27E-13 | 6 | 1.52E-11 | 2.77E-12 | 1.13E-12 |
|  | KO old | 9 | 9.26E-12 | 8.33E-13 | 2.78E-13 | 7 | 1.43E-11 | 2.29E-12 | 8.65E-13 |
|  | ABHD12 KO | 15 | 8.99E-12 | 7.93E-13 | 2.05E-13 | 13 | 1.47E-11 | 2.46E-12 | 6.83E-13 |
|  | Young | 12 | 8.29E-12 | 5.14E-13 | 1.48E-13 | 12 | 1.62E-11 | 3.01E-12 | 8.70E-13 |
|  | Old | 15 | 8.85E-12 | 8.57E-13 | 2.21E-13 | 12 | 1.44E-11 | 2.72E-12 | 7.87E-13 |
| *N*-oleoyl alanine | WT young | 6 | 3.46E-12 | 3.03E-13 | 1.24E-13 | 6 | 9.76E-12 | 7.63E-13 | 3.11E-13 |
|  | WT old | 6 | 4.44E-12 | 1.23E-12 | 5.02E-13 | 5 | 1.03E-11 | 2.34E-12 | 1.05E-12 |
|  | WT | 12 | 3.95E-12 | 9.94E-13 | 2.87E-13 | 11 | 1.00E-11 | 1.60E-12 | 4.84E-13 |
|  | KO young | 6 | 3.63E-12 | 1.04E-12 | 4.26E-13 | 6 | 7.69E-12 | 8.81E-13 | 3.60E-13 |
|  | KO old | 9 | 3.58E-12 | 5.60E-13 | 1.87E-13 | 7 | 7.34E-12 | 1.09E-12 | 4.12E-13 |
|  | ABHD12 KO | 15 | 3.60E-12 | 7.54E-13 | 1.95E-13 | 13 | 7.50E-12 | 9.75E-13 | 2.70E-13 |
|  | Young | 12 | 3.55E-12 | 7.37E-13 | 2.13E-13 | 12 | 8.73E-12 | 1.34E-12 | 3.86E-13 |
|  | Old | 15 | 3.92E-12 | 9.53E-13 | 2.46E-13 | 12 | 8.58E-12 | 2.24E-12 | 6.47E-13 |
| *N*-linoleoyl alanine | WT young | 6 | PISSR |  |  | 6 | PISSR |  |  |
|  | WT old | 6 | PISSR |  |  | 5 | PISSR |  |  |
|  | WT | 12 | PISSR |  |  | 11 | PISSR |  |  |
|  | KO young | 6 | PISSR |  |  | 6 | PISSR |  |  |
|  | KO old | 9 | PISSR |  |  | 7 | PISSR |  |  |
|  | ABHD12 KO | 15 | PISSR |  |  | 13 | PISSR |  |  |
|  | Young | 12 | PISSR |  |  | 12 | PISSR |  |  |
|  | Old | 15 | PISSR |  |  | 12 | PISSR |  |  |
| *N*-arachidonoyl alanine | WT young | 6 | 2.13E-12 | 1.93E-13 | 7.9E-14 | 6 | 6.11E-12 | 1.32E-12 | 5.40E-13 |
|  | WT old | 6 | 1.79E-12 | 3.32E-13 | 1.36E-13 | 5 | 5.17E-12 | 1.58E-12 | 7.06E-13 |
|  | WT | 12 | 1.96E-12 | 3.14E-13 | 9.08E-14 | 11 | 5.68E-12 | 1.45E-12 | 4.38E-13 |
|  | KO young | 6 | 2.46E-12 | 2.99E-13 | 1.22E-13 | 6 | 5.51E-12 | 9.60E-13 | 3.92E-13 |
|  | KO old | 9 | 2.46E-12 | 5.32E-13 | 1.77E-13 | 7 | 5.07E-12 | 1.13E-12 | 4.27E-13 |
|  | ABHD12 KO | 15 | 2.46E-12 | 4.40E-13 | 1.14E-13 | 13 | 5.28E-12 | 1.04E-12 | 2.88E-13 |
|  | Young | 12 | 2.30E-12 | 2.94E-13 | 8.49E-14 | 12 | 5.81E-12 | 1.15E-12 | 3.31E-13 |
|  | Old | 15 | 2.19E-12 | 5.61E-13 | 1.45E-13 | 12 | 5.11E-12 | 1.27E-12 | 3.66E-13 |
| *N*-docosahexaenoyl alanine | WT young | 6 | 1.24E-12 | 3.04E-13 | 1.24E-13 | 6 | PISSR |  |  |
|  | WT old | 6 | 1.15E-12 | 4.40E-13 | 1.8E-13 | 5 | PISSR |  |  |
|  | WT | 12 | 1.19E-12 | 3.63E-13 | 1.05E-13 | 11 | PISSR |  |  |
|  | KO young | 6 | 1.19E-12 | 1.59E-13 | 6.48E-14 | 6 | PISSR |  |  |
|  | KO old | 9 | 1.42E-12 | 3.21E-13 | 1.07E-13 | 7 | PISSR |  |  |
|  | ABHD12 KO | 15 | 1.33E-12 | 2.85E-13 | 7.36E-14 | 13 | PISSR |  |  |
|  | Young | 12 | 1.21E-12 | 2.33E-13 | 6.72E-14 | 12 | PISSR |  |  |
|  | Old | 15 | 1.31E-12 | 3.82E-13 | 9.88E-14 | 12 | PISSR |  |  |

Supplemental Table 4: Mean levels of *N*-acyl alanines in the midbrain and brainstem

| Lipid Species | Group | Midbrain | | | | Brainstem | | | |
| --- | --- | --- | --- | --- | --- | --- | --- | --- | --- |
|  |  | N | Mean | SD | SE | N | Mean | SD | SE |
| *N*-palmitoyl alanine | WT young | 6 | 4.91E-12 | 6.26E-13 | 2.56E-13 | 6 | 6.47E-12 | 1.06E-12 | 4.32E-13 |
|  | WT old | 6 | 4.73E-12 | 3.89E-13 | 1.59E-13 | 6 | 5.16E-12 | 2.82E-13 | 1.15E-13 |
|  | WT | 12 | 4.82E-12 | 5.06E-13 | 1.46E-13 | 12 | 5.82E-12 | 1.01E-12 | 2.90E-13 |
|  | KO young | 6 | 5.06E-12 | 8.28E-13 | 3.38E-13 | 6 | 6.87E-12 | 9.71E-13 | 3.96E-13 |
|  | KO old | 9 | 5.25E-12 | 6.49E-13 | 2.16E-13 | 9 | 5.71E-12 | 8.38E-13 | 2.79E-13 |
|  | ABHD12 KO | 15 | 5.17E-12 | 7.03E-13 | 1.82E-13 | 15 | 6.17E-12 | 1.04E-12 | 2.69E-13 |
|  | Young | 12 | 4.98E-12 | 7.04E-13 | 2.03E-13 | 12 | 6.67E-12 | 9.91E-13 | 2.86E-13 |
|  | Old | 15 | 5.04E-12 | 6.03E-13 | 1.56E-13 | 15 | 5.49E-12 | 7.11E-13 | 1.84E-13 |
| *N*-stearoyl alanine | WT young | 6 | 6.29E-12 | 5.76E-13 | 2.35E-13 | 6 | 1.07E-11 | 1.87E-12 | 7.64E-13 |
|  | WT old | 6 | 6.57E-12 | 5.04E-13 | 2.06E-13 | 6 | 8.94E-12 | 4.39E-13 | 1.79E-13 |
|  | WT | 12 | 6.43E-12 | 5.36E-13 | 1.55E-13 | 12 | 9.80E-12 | 1.58E-12 | 4.55E-13 |
|  | KO young | 6 | 7.07E-12 | 7.85E-13 | 3.2E-13 | 6 | 1.11E-11 | 1.72E-12 | 7.01E-13 |
|  | KO old | 9 | 7.23E-12 | 9.01E-13 | 3E-13 | 9 | 1.01E-11 | 1.63E-12 | 5.45E-13 |
|  | ABHD12 KO | 15 | 7.17E-12 | 8.31E-13 | 2.15E-13 | 15 | 1.05E-11 | 1.68E-12 | 4.33E-13 |
|  | Young | 12 | 6.68E-12 | 7.73E-13 | 2.23E-13 | 12 | 1.09E-11 | 1.73E-12 | 4.98E-13 |
|  | Old | 15 | 6.97E-12 | 8.18E-13 | 2.11E-13 | 15 | 9.66E-12 | 1.40E-12 | 3.61E-13 |
| *N*-oleoyl alanine | WT young | 6 | 4.19E-12 | 7.18E-13 | 2.93E-13 | 6 | 6.01E-12 | 8.98E-13 | 3.67E-13 |
|  | WT old | 6 | 4.76E-12 | 6.54E-13 | 2.67E-13 | 6 | 6.15E-12 | 3.41E-13 | 1.39E-13 |
|  | WT | 12 | 4.48E-12 | 7.19E-13 | 2.07E-13 | 12 | 6.08E-12 | 6.52E-13 | 1.88E-13 |
|  | KO young | 6 | 4.24E-12 | 3.81E-13 | 1.56E-13 | 6 | 6.58E-12 | 1.07E-12 | 4.39E-13 |
|  | KO old | 9 | 4.09E-12 | 4.62E-13 | 1.54E-13 | 9 | 5.22E-12 | 7.83E-13 | 2.61E-13 |
|  | ABHD12 KO | 15 | 4.15E-12 | 4.23E-13 | 1.09E-13 | 15 | 5.76E-12 | 1.11E-12 | 2.87E-13 |
|  | Young | 12 | 4.22E-12 | 5.49E-13 | 1.58E-13 | 12 | 6.29E-12 | 9.90E-13 | 2.86E-13 |
|  | Old | 15 | 4.36E-12 | 6.24E-13 | 1.61E-13 | 15 | 5.59E-12 | 7.84E-13 | 2.02E-13 |
| *N*-linoleoyl alanine | WT young | 6 | PISSR |  |  | 6 | 8.73E-13 | 2.50E-13 | 1.02E-13 |
|  | WT old | 6 | PISSR |  |  | 6 | 1.03E-12 | 2.12E-13 | 8.67E-14 |
|  | WT | 12 | PISSR |  |  | 12 | 9.50E-13 | 2.35E-13 | 6.79E-14 |
|  | KO young | 6 | PISSR |  |  | 6 | 1.07E-12 | 3.82E-13 | 1.56E-13 |
|  | KO old | 9 | PISSR |  |  | 9 | 7.22E-13 | 1.81E-13 | 6.02E-14 |
|  | ABHD12 KO | 15 | PISSR |  |  | 15 | 8.63E-13 | 3.20E-13 | 8.26E-14 |
|  | Young | 12 | PISSR |  |  | 12 | 9.74E-13 | 3.25E-13 | 9.38E-14 |
|  | Old | 15 | PISSR |  |  | 15 | 8.44E-13 | 2.42E-13 | 6.25E-14 |
| *N*-arachidonoyl alanine | WT young | 6 | 2.55E-12 | 4.90E-13 | 2E-13 | 6 | 2.59E-12 | 6.13E-13 | 2.50E-13 |
|  | WT old | 6 | 2.48E-12 | 7.00E-13 | 2.86E-13 | 6 | 1.49E-12 | 7.02E-13 | 2.87E-13 |
|  | WT | 12 | 2.51E-12 | 5.77E-13 | 1.67E-13 | 12 | 2.04E-12 | 8.54E-13 | 2.47E-13 |
|  | KO young | 6 | 3.41E-12 | 6.98E-13 | 2.85E-13 | 6 | 3.34E-12 | 1.17E-12 | 4.79E-13 |
|  | KO old | 9 | 3.28E-12 | 6.69E-13 | 2.23E-13 | 9 | 2.29E-12 | 6.32E-13 | 2.11E-13 |
|  | ABHD12 KO | 15 | 3.34E-12 | 6.59E-13 | 1.7E-13 | 15 | 2.71E-12 | 1.00E-12 | 2.59E-13 |
|  | Young | 12 | 2.98E-12 | 7.32E-13 | 2.11E-13 | 12 | 2.97E-12 | 9.75E-13 | 2.81E-13 |
|  | Old | 15 | 2.96E-12 | 7.73E-13 | 2E-13 | 15 | 1.97E-12 | 7.56E-13 | 1.95E-13 |
| *N*-docosahexaenoyl alanine | WT young | 6 | PISSR |  |  | 6 | 1.68E-12 | 5.94E-13 | 2.43E-13 |
|  | WT old | 6 | PISSR |  |  | 6 | 1.47E-12 | 3.71E-13 | 1.51E-13 |
|  | WT | 12 | PISSR |  |  | 12 | 1.57E-12 | 4.85E-13 | 1.40E-13 |
|  | KO young | 6 | PISSR |  |  | 6 | 2.36E-12 | 7.71E-13 | 3.15E-13 |
|  | KO old | 9 | PISSR |  |  | 9 | 1.41E-12 | 3.64E-13 | 1.21E-13 |
|  | ABHD12 KO | 15 | PISSR |  |  | 15 | 1.79E-12 | 7.18E-13 | 1.85E-13 |
|  | Young | 12 | PISSR |  |  | 12 | 2.02E-12 | 7.45E-13 | 2.15E-13 |
|  | Old | 15 | PISSR |  |  | 15 | 1.44E-12 | 3.54E-13 | 9.15E-14 |

Supplemental Table 5: Mean levels of *N*-acyl dopamines

| Lipid species | Group | Striatum | | | |
| --- | --- | --- | --- | --- | --- |
|  |  | N | Mean | SD | SE |
| *N*-oleoyl dopamine | WT young | 5 | PISSR |  |  |
|  | WT old | 6 | PISSR |  |  |
|  | WT | 11 | PISSR |  |  |
|  | KO young | 6 | PISSR |  |  |
|  | KO old | 9 | PISSR |  |  |
|  | ABHD12 KO | 15 | PISSR |  |  |
|  | Young | 11 | PISSR |  |  |
|  | Old | 15 | PISSR |  |  |
| *N*-arachidonoyl dopamine | WT young | 5 | PISSR |  |  |
|  | WT old | 6 | PISSR |  |  |
|  | WT | 11 | PISSR |  |  |
|  | KO young | 6 | PISSR |  |  |
|  | KO old | 9 | PISSR |  |  |
|  | ABHD12 KO | 15 | PISSR |  |  |
|  | Young | 11 | PISSR |  |  |
|  | Old | 15 | PISSR |  |  |

Note: *N*-acyl dopamines not detected in any other brain area

Supplemental Table 6: Mean levels of *N*-acyl ethanolamines in the striatum and hippocampus

| Lipid Species | Group | Striatum | | | | Hippocampus | | | |
| --- | --- | --- | --- | --- | --- | --- | --- | --- | --- |
|  |  | N | Mean | SD | SE | N | Mean | SD | SE |
| *N*-palmitoyl ethanolamine | WT young | 6 | 8.61E-11 | 1.37E-11 | 5.58E-12 | 6 | 7.95E-11 | 1.06E-11 | 4.31E-12 |
|  | WT old | 6 | 7.70E-11 | 1.41E-11 | 5.74E-12 | 6 | 6.49E-11 | 1.88E-11 | 7.66E-12 |
|  | WT | 12 | 8.15E-11 | 1.41E-11 | 4.06E-12 | 12 | 7.22E-11 | 1.64E-11 | 4.74E-12 |
|  | KO young | 6 | 6.25E-11 | 1.23E-11 | 5.04E-12 | 6 | 6.66E-11 | 1.34E-11 | 5.49E-12 |
|  | KO old | 9 | 8.16E-11 | 1.66E-11 | 5.52E-12 | 9 | 5.96E-11 | 2.02E-11 | 6.74E-12 |
|  | ABHD12 KO | 15 | 7.40E-11 | 1.75E-11 | 4.51E-12 | 15 | 6.24E-11 | 1.76E-11 | 4.55E-12 |
|  | Young | 12 | 7.43E-11 | 1.75E-11 | 5.05E-12 | 12 | 7.31E-11 | 1.34E-11 | 3.86E-12 |
|  | Old | 15 | 7.98E-11 | 1.53E-11 | 3.94E-12 | 15 | 6.17E-11 | 1.91E-11 | 4.94E-12 |
| *N*-stearoyl ethanolamine | WT young | 5 | 1.89E-11 | 3.79E-12 | 1.69E-12 | 6 | 8.04E-12 | 3.95E-12 | 1.61E-12 |
|  | WT old | 6 | 1.55E-11 | 2.91E-12 | 1.19E-12 | 6 | 1.14E-11 | 4.57E-12 | 1.87E-12 |
|  | WT | 11 | 1.70E-11 | 3.62E-12 | 1.09E-12 | 12 | 9.73E-12 | 4.44E-12 | 1.28E-12 |
|  | KO young | 6 | 1.38E-11 | 4.10E-12 | 1.67E-12 | 6 | 7.60E-12 | 3.91E-12 | 1.60E-12 |
|  | KO old | 9 | 2.17E-11 | 4.89E-12 | 1.63E-12 | 9 | 1.28E-11 | 5.60E-12 | 1.87E-12 |
|  | ABHD12 KO | 15 | 1.85E-11 | 5.99E-12 | 1.55E-12 | 15 | 1.07E-11 | 5.52E-12 | 1.42E-12 |
|  | Young | 11 | 1.61E-11 | 4.60E-12 | 1.39E-12 | 12 | 7.82E-12 | 3.76E-12 | 1.08E-12 |
|  | Old | 15 | 1.92E-11 | 5.18E-12 | 1.34E-12 | 15 | 1.23E-11 | 5.09E-12 | 1.31E-12 |
| *N*-oleoyl ethanolamine | WT young | 6 | 8.93E-11 | 1.71E-11 | 6.98E-12 | 6 | 1.23E-10 | 3.16E-11 | 1.29E-11 |
|  | WT old | 6 | 8.83E-11 | 2.06E-11 | 8.41E-12 | 6 | 8.43E-11 | 3.09E-11 | 1.26E-11 |
|  | WT | 12 | 8.88E-11 | 1.81E-11 | 5.21E-12 | 12 | 1.03E-10 | 3.59E-11 | 1.04E-11 |
|  | KO young | 6 | 7.52E-11 | 1.29E-11 | 5.25E-12 | 6 | 1.08E-10 | 3.77E-11 | 1.54E-11 |
|  | KO old | 9 | 9.50E-11 | 2.14E-11 | 7.15E-12 | 9 | 8.32E-11 | 2.75E-11 | 9.15E-12 |
|  | ABHD12 KO | 15 | 8.71E-11 | 2.06E-11 | 5.31E-12 | 15 | 9.31E-11 | 3.31E-11 | 8.54E-12 |
|  | Young | 12 | 8.22E-11 | 1.62E-11 | 4.68E-12 | 12 | 1.15E-10 | 3.41E-11 | 9.83E-12 |
|  | Old | 15 | 9.23E-11 | 2.06E-11 | 5.33E-12 | 15 | 8.37E-11 | 2.78E-11 | 7.18E-12 |
| *N*-linoleoyl ethanolamine | WT young | 6 | 8.02E-11 | 1.54E-11 | 6.27E-12 | 6 | 7.72E-11 | 1.12E-11 | 4.56E-12 |
|  | WT old | 6 | 6.38E-11 | 1.79E-11 | 7.31E-12 | 6 | 3.99E-11 | 1.10E-11 | 4.51E-12 |
|  | WT | 12 | 7.20E-11 | 1.81E-11 | 5.22E-12 | 12 | 5.86E-11 | 2.22E-11 | 6.41E-12 |
|  | KO young | 6 | 7.21E-11 | 1.23E-11 | 5.03E-12 | 6 | 7.68E-11 | 1.24E-11 | 5.08E-12 |
|  | KO old | 9 | 6.62E-11 | 1.42E-11 | 4.72E-12 | 9 | 4.16E-11 | 1.23E-11 | 4.10E-12 |
|  | ABHD12 KO | 15 | 6.85E-11 | 1.33E-11 | 3.44E-12 | 15 | 5.57E-11 | 2.14E-11 | 5.54E-12 |
|  | Young | 12 | 7.62E-11 | 1.39E-11 | 4.02E-12 | 12 | 7.70E-11 | 1.13E-11 | 3.25E-12 |
|  | Old | 15 | 6.52E-11 | 1.52E-11 | 3.92E-12 | 15 | 4.09E-11 | 1.14E-11 | 2.95E-12 |
| *N*-arachidonoyl ethanolamine | WT young | 6 | 2.80E-11 | 6.83E-12 | 2.79E-12 | 6 | 4.89E-11 | 6.37E-12 | 2.60E-12 |
|  | WT old | 6 | 2.36E-11 | 9.49E-12 | 3.88E-12 | 6 | 2.95E-11 | 7.26E-12 | 2.96E-12 |
|  | WT | 12 | 2.58E-11 | 8.21E-12 | 2.37E-12 | 12 | 3.92E-11 | 1.20E-11 | 3.47E-12 |
|  | KO young | 6 | 3.54E-11 | 1.24E-11 | 5.05E-12 | 6 | 6.31E-11 | 1.04E-11 | 4.24E-12 |
|  | KO old | 9 | 3.23E-11 | 6.98E-12 | 2.33E-12 | 9 | 3.93E-11 | 9.73E-12 | 3.24E-12 |
|  | ABHD12 KO | 15 | 3.35E-11 | 9.22E-12 | 2.38E-12 | 15 | 4.88E-11 | 1.55E-11 | 3.99E-12 |
|  | Young | 12 | 3.17E-11 | 1.03E-11 | 2.97E-12 | 12 | 5.60E-11 | 1.11E-11 | 3.20E-12 |
|  | Old | 15 | 2.88E-11 | 8.91E-12 | 2.30E-12 | 15 | 3.54E-11 | 9.87E-12 | 2.55E-12 |
| *N*-docosahexaenoyl ethanolamine | WT young | 6 | 4.86E-11 | 6.38E-12 | 2.60E-12 | 6 | 5.47E-11 | 7.79E-12 | 3.18E-12 |
|  | WT old | 6 | 4.01E-11 | 9.62E-12 | 3.93E-12 | 6 | 3.78E-11 | 4.71E-12 | 1.92E-12 |
|  | WT | 12 | 4.43E-11 | 8.96E-12 | 2.59E-12 | 12 | 4.63E-11 | 1.07E-11 | 3.10E-12 |
|  | KO young | 6 | 4.12E-11 | 7.93E-12 | 3.24E-12 | 6 | 4.66E-11 | 5.01E-12 | 2.04E-12 |
|  | KO old | 9 | 3.68E-11 | 5.02E-12 | 1.67E-12 | 9 | 3.06E-11 | 2.48E-12 | 8.26E-13 |
|  | ABHD12 KO | 15 | 3.85E-11 | 6.47E-12 | 1.67E-12 | 15 | 3.70E-11 | 8.85E-12 | 2.28E-12 |
|  | Young | 12 | 4.49E-11 | 7.88E-12 | 2.27E-12 | 12 | 5.07E-11 | 7.55E-12 | 2.18E-12 |
|  | Old | 15 | 3.81E-11 | 7.09E-12 | 1.83E-12 | 15 | 3.35E-11 | 4.99E-12 | 1.29E-12 |

Supplemental Table 7: Mean levels of *N*-acyl ethanolamines in the cerebellum and thalamus

| Lipid Species | Group | Cerebellum | | | | Thalamus | | | |
| --- | --- | --- | --- | --- | --- | --- | --- | --- | --- |
|  |  | N | Mean | SD | SE | N | Mean | SD | SE |
| *N*-palmitoyl ethanolamine | WT young | 6 | 8.54E-11 | 2.03E-11 | 8.3E-12 | 6 | 9.76E-11 | 1.92E-11 | 7.82E-12 |
|  | WT old | 6 | 1.16E-10 | 2.08E-11 | 8.5E-12 | 6 | 1.20E-10 | 2.00E-11 | 8.15E-12 |
|  | WT | 12 | 1.01E-10 | 2.52E-11 | 7.29E-12 | 12 | 1.09E-10 | 2.21E-11 | 6.37E-12 |
|  | KO young | 6 | 6.02E-11 | 1.93E-11 | 7.87E-12 | 6 | 1.02E-10 | 1.89E-11 | 7.73E-12 |
|  | KO old | 9 | 1.22E-10 | 1.65E-11 | 5.49E-12 | 9 | 1.32E-10 | 2.56E-11 | 8.53E-12 |
|  | ABHD12 KO | 15 | 9.75E-11 | 3.58E-11 | 9.24E-12 | 15 | 1.20E-10 | 2.69E-11 | 6.94E-12 |
|  | Young | 12 | 7.28E-11 | 2.30E-11 | 6.64E-12 | 12 | 1.00E-10 | 1.83E-11 | 5.29E-12 |
|  | Old | 15 | 1.20E-10 | 1.79E-11 | 4.63E-12 | 15 | 1.27E-10 | 2.34E-11 | 6.05E-12 |
| *N*-stearoyl ethanolamine | WT young | 6 | 5.06E-12 | 3.66E-12 | 1.5E-12 | 6 | 5.57E-12 | 2.76E-12 | 1.13E-12 |
|  | WT old | 6 | 2.05E-11 | 1.30E-11 | 5.32E-12 | 6 | 1.37E-11 | 5.49E-12 | 2.24E-12 |
|  | WT | 12 | 1.28E-11 | 1.22E-11 | 3.51E-12 | 12 | 9.65E-12 | 5.95E-12 | 1.72E-12 |
|  | KO young | 6 | 9.99E-12 | 1.57E-11 | 6.42E-12 | 6 | 7.97E-12 | 5.62E-12 | 2.29E-12 |
|  | KO old | 9 | 2.31E-11 | 1.09E-11 | 3.64E-12 | 9 | 2.04E-11 | 1.01E-11 | 3.36E-12 |
|  | ABHD12 KO | 15 | 1.79E-11 | 1.42E-11 | 3.66E-12 | 15 | 1.54E-11 | 1.04E-11 | 2.69E-12 |
|  | Young | 12 | 7.52E-12 | 1.12E-11 | 3.23E-12 | 12 | 6.77E-12 | 4.40E-12 | 1.27E-12 |
|  | Old | 15 | 2.20E-11 | 1.14E-11 | 2.95E-12 | 15 | 1.77E-11 | 8.95E-12 | 2.31E-12 |
| *N*-oleoyl ethanolamine | WT young | 6 | 1.03E-10 | 3.33E-11 | 1.36E-11 | 6 | 1.13E-10 | 2.63E-11 | 1.07E-11 |
|  | WT old | 6 | 1.82E-10 | 6.79E-11 | 2.77E-11 | 6 | 1.38E-10 | 2.84E-11 | 1.16E-11 |
|  | WT | 12 | 1.43E-10 | 6.55E-11 | 1.89E-11 | 12 | 1.25E-10 | 2.91E-11 | 8.41E-12 |
|  | KO young | 6 | 1.10E-10 | 5.73E-11 | 2.34E-11 | 6 | 1.40E-10 | 3.25E-11 | 1.33E-11 |
|  | KO old | 9 | 1.80E-10 | 5.32E-11 | 1.77E-11 | 9 | 1.58E-10 | 3.30E-11 | 1.10E-11 |
|  | ABHD12 KO | 15 | 1.52E-10 | 6.36E-11 | 1.64E-11 | 15 | 1.50E-10 | 3.29E-11 | 8.49E-12 |
|  | Young | 12 | 1.07E-10 | 4.48E-11 | 1.29E-11 | 12 | 1.26E-10 | 3.15E-11 | 9.10E-12 |
|  | Old | 15 | 1.81E-10 | 5.71E-11 | 1.47E-11 | 15 | 1.50E-10 | 3.18E-11 | 8.22E-12 |
| *N*-linoleoyl ethanolamine | WT young | 6 | 4.83E-11 | 6.65E-12 | 2.71E-12 | 6 | 4.06E-11 | 8.03E-12 | 3.28E-12 |
|  | WT old | 6 | 4.71E-11 | 7.06E-12 | 2.88E-12 | 6 | 3.30E-11 | 4.35E-12 | 1.77E-12 |
|  | WT | 12 | 4.77E-11 | 6.57E-12 | 1.9E-12 | 12 | 3.68E-11 | 7.32E-12 | 2.11E-12 |
|  | KO young | 6 | 5.88E-11 | 1.39E-11 | 5.66E-12 | 6 | 5.70E-11 | 1.06E-11 | 4.34E-12 |
|  | KO old | 9 | 5.86E-11 | 9.17E-12 | 3.06E-12 | 9 | 4.10E-11 | 7.37E-12 | 2.46E-12 |
|  | ABHD12 KO | 15 | 5.87E-11 | 1.08E-11 | 2.79E-12 | 15 | 4.74E-11 | 1.17E-11 | 3.03E-12 |
|  | Young | 12 | 5.35E-11 | 1.17E-11 | 3.38E-12 | 12 | 4.88E-11 | 1.24E-11 | 3.59E-12 |
|  | Old | 15 | 5.40E-11 | 1.00E-11 | 2.58E-12 | 15 | 3.78E-11 | 7.36E-12 | 1.90E-12 |
| *N*-arachidonoyl ethanolamine | WT young | 6 | 2.28E-11 | 2.83E-12 | 1.15E-12 | 6 | 2.23E-11 | 4.55E-12 | 1.86E-12 |
|  | WT old | 6 | 2.49E-11 | 3.99E-12 | 1.63E-12 | 6 | 2.10E-11 | 3.57E-12 | 1.46E-12 |
|  | WT | 12 | 2.39E-11 | 3.47E-12 | 1E-12 | 12 | 2.17E-11 | 3.96E-12 | 1.14E-12 |
|  | KO young | 6 | 4.04E-11 | 8.62E-12 | 3.52E-12 | 6 | 4.44E-11 | 1.37E-11 | 5.59E-12 |
|  | KO old | 9 | 4.49E-11 | 6.67E-12 | 2.22E-12 | 9 | 3.32E-11 | 5.22E-12 | 1.74E-12 |
|  | ABHD12 KO | 15 | 4.31E-11 | 7.56E-12 | 1.95E-12 | 15 | 3.77E-11 | 1.07E-11 | 2.77E-12 |
|  | Young | 12 | 3.16E-11 | 1.10E-11 | 3.18E-12 | 12 | 3.34E-11 | 1.51E-11 | 4.36E-12 |
|  | Old | 15 | 3.69E-11 | 1.16E-11 | 2.99E-12 | 15 | 2.83E-11 | 7.64E-12 | 1.97E-12 |
| *N*-docosahexaenoyl ethanolamine | WT young | 6 | 6.31E-11 | 9.64E-12 | 3.94E-12 | 6 | 4.94E-11 | 7.27E-12 | 2.97E-12 |
|  | WT old | 6 | 6.45E-11 | 6.59E-12 | 2.69E-12 | 6 | 4.89E-11 | 8.91E-12 | 3.64E-12 |
|  | WT | 12 | 6.38E-11 | 7.91E-12 | 2.28E-12 | 12 | 4.92E-11 | 7.76E-12 | 2.24E-12 |
|  | KO young | 6 | 6.07E-11 | 9.05E-12 | 3.7E-12 | 6 | 6.27E-11 | 1.51E-11 | 6.16E-12 |
|  | KO old | 9 | 6.54E-11 | 7.23E-12 | 2.41E-12 | 9 | 5.01E-11 | 6.92E-12 | 2.31E-12 |
|  | ABHD12 KO | 15 | 6.35E-11 | 8.06E-12 | 2.08E-12 | 15 | 5.51E-11 | 1.22E-11 | 3.16E-12 |
|  | Young | 12 | 6.19E-11 | 9.01E-12 | 2.6E-12 | 12 | 5.60E-11 | 1.32E-11 | 3.82E-12 |
|  | Old | 15 | 6.51E-11 | 6.75E-12 | 1.74E-12 | 15 | 4.96E-11 | 7.49E-12 | 1.93E-12 |

Supplemental Table 8: Mean levels of *N*-acyl ethanolamines in the cortex and hypothalamus

| Lipid Species | Group | Cortex | | | | Hypothalamus | | | |
| --- | --- | --- | --- | --- | --- | --- | --- | --- | --- |
|  |  | N | Mean | SD | SE | N | Mean | SD | SE |
| *N*-palmitoyl ethanolamine | WT young | 6 | 2.93E-11 | 5.80E-12 | 2.37E-12 | 6 | 4.11E-11 | 1.01E-11 | 4.12E-12 |
|  | WT old | 6 | 3.90E-11 | 7.53E-12 | 3.08E-12 | 5 | 3.28E-11 | 5.95E-12 | 2.66E-12 |
|  | WT | 12 | 3.42E-11 | 8.20E-12 | 2.37E-12 | 11 | 3.74E-11 | 9.15E-12 | 2.76E-12 |
|  | KO young | 6 | 2.57E-11 | 8.64E-12 | 3.53E-12 | 6 | 3.10E-11 | 9.08E-12 | 3.71E-12 |
|  | KO old | 9 | 4.02E-11 | 9.90E-12 | 3.3E-12 | 7 | 2.65E-11 | 8.48E-12 | 3.20E-12 |
|  | ABHD12 KO | 15 | 3.44E-11 | 1.17E-11 | 3.02E-12 | 13 | 2.86E-11 | 8.70E-12 | 2.41E-12 |
|  | Young | 12 | 2.75E-11 | 7.26E-12 | 2.09E-12 | 12 | 3.60E-11 | 1.06E-11 | 3.05E-12 |
|  | Old | 15 | 3.97E-11 | 8.75E-12 | 2.26E-12 | 12 | 2.91E-11 | 7.92E-12 | 2.29E-12 |
| *N*-stearoyl ethanolamine | WT young | 6 | 3.86E-12 | 2.71E-12 | 1.11E-12 | 6 | 6.43E-12 | 1.20E-12 | 4.88E-13 |
|  | WT old | 6 | 5.47E-12 | 2.65E-12 | 1.08E-12 | 5 | 6.80E-12 | 1.37E-12 | 6.12E-13 |
|  | WT | 12 | 4.67E-12 | 2.69E-12 | 7.77E-13 | 11 | 6.60E-12 | 1.23E-12 | 3.69E-13 |
|  | KO young | 6 | 3.64E-12 | 2.22E-12 | 9.08E-13 | 6 | 5.29E-12 | 2.17E-12 | 8.86E-13 |
|  | KO old | 9 | 7.30E-12 | 4.30E-12 | 1.43E-12 | 7 | 6.70E-12 | 1.91E-12 | 7.22E-13 |
|  | ABHD12 KO | 15 | 5.84E-12 | 3.97E-12 | 1.03E-12 | 13 | 6.05E-12 | 2.08E-12 | 5.77E-13 |
|  | Young | 12 | 3.75E-12 | 2.37E-12 | 6.83E-13 | 12 | 5.86E-12 | 1.77E-12 | 5.12E-13 |
|  | Old | 15 | 6.57E-12 | 3.74E-12 | 9.65E-13 | 12 | 6.74E-12 | 1.64E-12 | 4.72E-13 |
| *N*-oleoyl ethanolamine | WT young | 6 | 4.49E-11 | 1.11E-11 | 4.52E-12 | 6 | 7.04E-11 | 2.15E-11 | 8.76E-12 |
|  | WT old | 6 | 5.92E-11 | 1.42E-11 | 5.78E-12 | 5 | 5.46E-11 | 1.07E-11 | 4.77E-12 |
|  | WT | 12 | 5.20E-11 | 1.42E-11 | 4.11E-12 | 11 | 6.33E-11 | 1.85E-11 | 5.59E-12 |
|  | KO young | 6 | 4.18E-11 | 1.25E-11 | 5.11E-12 | 6 | 5.90E-11 | 2.20E-11 | 9.00E-12 |
|  | KO old | 9 | 6.26E-11 | 1.44E-11 | 4.8E-12 | 7 | 4.57E-11 | 1.41E-11 | 5.32E-12 |
|  | ABHD12 KO | 15 | 5.43E-11 | 1.69E-11 | 4.36E-12 | 13 | 5.18E-11 | 1.87E-11 | 5.18E-12 |
|  | Young | 12 | 4.34E-11 | 1.14E-11 | 3.29E-12 | 12 | 6.47E-11 | 2.16E-11 | 6.23E-12 |
|  | Old | 15 | 6.12E-11 | 1.39E-11 | 3.59E-12 | 12 | 4.94E-11 | 1.31E-11 | 3.77E-12 |
| *N*-linoleoyl ethanolamine | WT young | 6 | 4.02E-11 | 1.23E-11 | 5.03E-12 | 6 | 2.14E-11 | 6.55E-12 | 2.68E-12 |
|  | WT old | 6 | 3.36E-11 | 4.64E-12 | 1.89E-12 | 5 | 1.32E-11 | 3.58E-12 | 1.60E-12 |
|  | WT | 12 | 3.69E-11 | 9.52E-12 | 2.75E-12 | 11 | 1.77E-11 | 6.69E-12 | 2.02E-12 |
|  | KO young | 6 | 4.40E-11 | 1.51E-11 | 6.15E-12 | 6 | 1.97E-11 | 7.28E-12 | 2.97E-12 |
|  | KO old | 9 | 3.95E-11 | 7.53E-12 | 2.51E-12 | 7 | 1.29E-11 | 4.01E-12 | 1.52E-12 |
|  | ABHD12 KO | 15 | 4.13E-11 | 1.09E-11 | 2.81E-12 | 13 | 1.60E-11 | 6.54E-12 | 1.81E-12 |
|  | Young | 12 | 4.21E-11 | 1.33E-11 | 3.83E-12 | 12 | 2.06E-11 | 6.66E-12 | 1.92E-12 |
|  | Old | 15 | 3.72E-11 | 7.02E-12 | 1.81E-12 | 12 | 1.30E-11 | 3.67E-12 | 1.06E-12 |
| *N*-arachidonoyl ethanolamine | WT young | 6 | 1.59E-11 | 1.60E-12 | 6.55E-13 | 6 | 1.38E-11 | 4.23E-12 | 1.73E-12 |
|  | WT old | 6 | 1.63E-11 | 1.85E-12 | 7.57E-13 | 5 | 9.34E-12 | 2.41E-12 | 1.08E-12 |
|  | WT | 12 | 1.61E-11 | 1.66E-12 | 4.8E-13 | 11 | 1.18E-11 | 4.09E-12 | 1.23E-12 |
|  | KO young | 6 | 2.58E-11 | 9.12E-12 | 3.72E-12 | 6 | 1.78E-11 | 6.84E-12 | 2.79E-12 |
|  | KO old | 9 | 2.57E-11 | 4.17E-12 | 1.39E-12 | 7 | 1.26E-11 | 3.36E-12 | 1.27E-12 |
|  | ABHD12 KO | 15 | 2.57E-11 | 6.29E-12 | 1.63E-12 | 13 | 1.50E-11 | 5.69E-12 | 1.58E-12 |
|  | Young | 12 | 2.09E-11 | 8.09E-12 | 2.34E-12 | 12 | 1.58E-11 | 5.80E-12 | 1.67E-12 |
|  | Old | 15 | 2.19E-11 | 5.82E-12 | 1.5E-12 | 12 | 1.12E-11 | 3.33E-12 | 9.60E-13 |
| *N*-docosahexaenoyl ethanolamine | WT young | 6 | 2.65E-11 | 2.66E-12 | 1.09E-12 | 6 | 2.69E-11 | 7.99E-12 | 3.26E-12 |
|  | WT old | 6 | 2.85E-11 | 2.80E-12 | 1.14E-12 | 5 | 2.01E-11 | 4.65E-12 | 2.08E-12 |
|  | WT | 12 | 2.75E-11 | 2.82E-12 | 8.13E-13 | 11 | 2.38E-11 | 7.29E-12 | 2.20E-12 |
|  | KO young | 6 | 2.78E-11 | 8.93E-12 | 3.65E-12 | 6 | 3.12E-11 | 6.35E-12 | 2.59E-12 |
|  | KO old | 9 | 2.92E-11 | 4.46E-12 | 1.49E-12 | 7 | 2.49E-11 | 4.22E-12 | 1.60E-12 |
|  | ABHD12 KO | 15 | 2.86E-11 | 6.35E-12 | 1.64E-12 | 13 | 2.78E-11 | 6.04E-12 | 1.67E-12 |
|  | Young | 12 | 2.71E-11 | 6.32E-12 | 1.82E-12 | 12 | 2.90E-11 | 7.24E-12 | 2.09E-12 |
|  | Old | 15 | 2.89E-11 | 3.78E-12 | 9.75E-13 | 12 | 2.29E-11 | 4.86E-12 | 1.40E-12 |

Supplemental Table 9: Mean levels of *N*-acyl ethanolamines in the midbrain and brainstem

| Lipid Species | Group | Midbrain | | | | Brainstem | | | |
| --- | --- | --- | --- | --- | --- | --- | --- | --- | --- |
|  |  | N | Mean | SD | SE | N | Mean | SD | SE |
| *N*-palmitoyl ethanolamine | WT young | 6 | 6.25E-11 | 1.33E-11 | 5.42E-12 | 6 | 1.16E-10 | 2.28E-11 | 9.30E-12 |
|  | WT old | 6 | 1.04E-10 | 1.69E-11 | 6.89E-12 | 6 | 1.97E-10 | 2.62E-11 | 1.07E-11 |
|  | WT | 12 | 8.33E-11 | 2.62E-11 | 7.55E-12 | 12 | 1.57E-10 | 4.85E-11 | 1.40E-11 |
|  | KO young | 6 | 6.06E-11 | 1.68E-11 | 6.84E-12 | 6 | 1.30E-10 | 2.73E-11 | 1.12E-11 |
|  | KO old | 9 | 9.33E-11 | 2.05E-11 | 6.84E-12 | 9 | 1.43E-10 | 3.08E-11 | 1.03E-11 |
|  | ABHD12 KO | 15 | 8.02E-11 | 2.48E-11 | 6.41E-12 | 15 | 1.38E-10 | 2.93E-11 | 7.56E-12 |
|  | Young | 12 | 6.15E-11 | 1.44E-11 | 4.17E-12 | 12 | 1.23E-10 | 2.50E-11 | 7.21E-12 |
|  | Old | 15 | 9.76E-11 | 1.93E-11 | 4.99E-12 | 15 | 1.65E-10 | 3.92E-11 | 1.01E-11 |
| *N*-stearoyl ethanolamine | WT young | 6 | 2.01E-12 | 7.14E-13 | 2.92E-13 | 6 | 9.63E-12 | 3.53E-12 | 1.44E-12 |
|  | WT old | 6 | 9.51E-12 | 1.83E-12 | 7.46E-13 | 6 | 3.83E-11 | 7.61E-12 | 3.11E-12 |
|  | WT | 12 | 5.76E-12 | 4.13E-12 | 1.19E-12 | 12 | 2.40E-11 | 1.60E-11 | 4.62E-12 |
|  | KO young | 6 | 3.28E-12 | 1.19E-12 | 4.85E-13 | 6 | 1.61E-11 | 8.05E-12 | 3.29E-12 |
|  | KO old | 9 | 1.14E-11 | 4.09E-12 | 1.36E-12 | 9 | 3.41E-11 | 1.63E-11 | 5.45E-12 |
|  | ABHD12 KO | 15 | 8.13E-12 | 5.18E-12 | 1.34E-12 | 15 | 2.69E-11 | 1.61E-11 | 4.16E-12 |
|  | Young | 12 | 2.65E-12 | 1.15E-12 | 3.31E-13 | 12 | 1.29E-11 | 6.83E-12 | 1.97E-12 |
|  | Old | 15 | 1.06E-11 | 3.41E-12 | 8.82E-13 | 15 | 3.58E-11 | 1.33E-11 | 3.44E-12 |
| *N*-oleoyl ethanolamine | WT young | 6 | 7.15E-11 | 1.73E-11 | 7.08E-12 | 6 | 1.13E-10 | 1.61E-11 | 6.58E-12 |
|  | WT old | 6 | 1.13E-10 | 1.83E-11 | 7.48E-12 | 6 | 1.92E-10 | 2.00E-11 | 8.15E-12 |
|  | WT | 12 | 9.23E-11 | 2.77E-11 | 7.98E-12 | 12 | 1.53E-10 | 4.44E-11 | 1.28E-11 |
|  | KO young | 6 | 7.74E-11 | 1.59E-11 | 6.49E-12 | 6 | 1.50E-10 | 3.55E-11 | 1.45E-11 |
|  | KO old | 9 | 1.20E-10 | 2.96E-11 | 9.88E-12 | 9 | 1.54E-10 | 4.81E-11 | 1.60E-11 |
|  | ABHD12 KO | 15 | 1.03E-10 | 3.27E-11 | 8.44E-12 | 15 | 1.52E-10 | 4.22E-11 | 1.09E-11 |
|  | Young | 12 | 7.44E-11 | 1.62E-11 | 4.67E-12 | 12 | 1.32E-10 | 3.24E-11 | 9.36E-12 |
|  | Old | 15 | 1.18E-10 | 2.52E-11 | 6.51E-12 | 15 | 1.69E-10 | 4.28E-11 | 1.10E-11 |
| *N*-linoleoyl ethanolamine | WT young | 6 | 2.59E-11 | 3.16E-12 | 1.29E-12 | 6 | 1.94E-11 | 1.79E-12 | 7.30E-13 |
|  | WT old | 6 | 2.41E-11 | 2.65E-12 | 1.08E-12 | 6 | 1.98E-11 | 2.49E-12 | 1.02E-12 |
|  | WT | 12 | 2.50E-11 | 2.93E-12 | 8.47E-13 | 12 | 1.96E-11 | 2.08E-12 | 6.01E-13 |
|  | KO young | 6 | 3.26E-11 | 8.35E-12 | 3.41E-12 | 6 | 2.79E-11 | 6.87E-12 | 2.81E-12 |
|  | KO old | 9 | 2.55E-11 | 3.92E-12 | 1.31E-12 | 9 | 2.00E-11 | 5.26E-12 | 1.75E-12 |
|  | ABHD12 KO | 15 | 2.83E-11 | 6.81E-12 | 1.76E-12 | 15 | 2.32E-11 | 6.99E-12 | 1.80E-12 |
|  | Young | 12 | 2.92E-11 | 6.96E-12 | 2.01E-12 | 12 | 2.36E-11 | 6.55E-12 | 1.89E-12 |
|  | Old | 15 | 2.50E-11 | 3.44E-12 | 8.87E-13 | 15 | 1.99E-11 | 4.25E-12 | 1.10E-12 |
| *N*-arachidonoyl ethanolamine | WT young | 6 | 1.85E-11 | 2.41E-12 | 9.83E-13 | 6 | 1.44E-11 | 1.69E-12 | 6.88E-13 |
|  | WT old | 6 | 1.88E-11 | 1.73E-12 | 7.08E-13 | 6 | 1.63E-11 | 1.60E-12 | 6.53E-13 |
|  | WT | 12 | 1.87E-11 | 2.01E-12 | 5.79E-13 | 12 | 1.53E-11 | 1.86E-12 | 5.36E-13 |
|  | KO young | 6 | 2.78E-11 | 5.86E-12 | 2.39E-12 | 6 | 2.55E-11 | 5.35E-12 | 2.18E-12 |
|  | KO old | 9 | 2.57E-11 | 4.45E-12 | 1.48E-12 | 9 | 2.18E-11 | 4.84E-12 | 1.61E-12 |
|  | ABHD12 KO | 15 | 2.66E-11 | 4.97E-12 | 1.28E-12 | 15 | 2.33E-11 | 5.21E-12 | 1.34E-12 |
|  | Young | 12 | 2.32E-11 | 6.48E-12 | 1.87E-12 | 12 | 1.99E-11 | 6.93E-12 | 2.00E-12 |
|  | Old | 15 | 2.30E-11 | 4.96E-12 | 1.28E-12 | 15 | 1.96E-11 | 4.70E-12 | 1.21E-12 |
| *N*-docosahexaenoyl ethanolamine | WT young | 6 | 5.03E-11 | 5.39E-12 | 2.2E-12 | 6 | 4.62E-11 | 5.15E-12 | 2.10E-12 |
|  | WT old | 6 | 5.15E-11 | 4.52E-12 | 1.85E-12 | 6 | 4.90E-11 | 6.43E-12 | 2.63E-12 |
|  | WT | 12 | 5.09E-11 | 4.78E-12 | 1.38E-12 | 12 | 4.76E-11 | 5.74E-12 | 1.66E-12 |
|  | KO young | 6 | 5.03E-11 | 8.49E-12 | 3.47E-12 | 6 | 5.02E-11 | 8.63E-12 | 3.52E-12 |
|  | KO old | 9 | 4.92E-11 | 6.75E-12 | 2.25E-12 | 9 | 4.69E-11 | 8.00E-12 | 2.67E-12 |
|  | ABHD12 KO | 15 | 4.96E-11 | 7.22E-12 | 1.86E-12 | 15 | 4.82E-11 | 8.13E-12 | 2.10E-12 |
|  | Young | 12 | 5.03E-11 | 6.78E-12 | 1.96E-12 | 12 | 4.82E-11 | 7.09E-12 | 2.05E-12 |
|  | Old | 15 | 5.01E-11 | 5.89E-12 | 1.52E-12 | 15 | 4.77E-11 | 7.25E-12 | 1.87E-12 |

Supplemental Table 10: Mean levels of *N*-acyl GABAs in the striatum and hippocampus

| Lipid Species | Group | Striatum | | | | Hippocampus | | | |
| --- | --- | --- | --- | --- | --- | --- | --- | --- | --- |
|  |  | N | Mean | SD | SE | N | Mean | SD | SE |
| *N*-palmitoyl GABA | WT young | 6 | 7.02E-12 | 8.85E-13 | 3.61E-13 | 6 | 6.04E-12 | 6.96E-13 | 2.84E-13 |
|  | WT old | 6 | 4.62E-12 | 8.76E-13 | 3.58E-13 | 6 | 3.56E-12 | 1.80E-13 | 7.37E-14 |
|  | WT | 12 | 5.82E-12 | 1.51E-12 | 4.36E-13 | 12 | 4.80E-12 | 1.38E-12 | 3.99E-13 |
|  | KO young | 6 | 5.84E-12 | 1.03E-12 | 4.20E-13 | 6 | 5.34E-12 | 9.51E-13 | 3.88E-13 |
|  | KO old | 9 | 4.40E-12 | 6.94E-13 | 2.31E-13 | 9 | 3.34E-12 | 6.78E-13 | 2.26E-13 |
|  | ABHD12 KO | 15 | 4.98E-12 | 1.09E-12 | 2.81E-13 | 15 | 4.14E-12 | 1.27E-12 | 3.28E-13 |
|  | Young | 12 | 6.43E-12 | 1.10E-12 | 3.18E-13 | 12 | 5.69E-12 | 8.73E-13 | 2.52E-13 |
|  | Old | 15 | 4.49E-12 | 7.49E-13 | 1.94E-13 | 15 | 3.43E-12 | 5.35E-13 | 1.38E-13 |
| *N*-stearoyl GABA | WT young | 6 | 1.11E-11 | 1.24E-12 | 5.06E-13 | 6 | 7.48E-12 | 9.79E-13 | 4.00E-13 |
|  | WT old | 6 | 7.82E-12 | 8.96E-13 | 3.66E-13 | 6 | 4.73E-12 | 6.59E-13 | 2.69E-13 |
|  | WT | 12 | 9.44E-12 | 1.99E-12 | 5.73E-13 | 12 | 6.11E-12 | 1.64E-12 | 4.74E-13 |
|  | KO young | 6 | 1.01E-11 | 1.85E-12 | 7.53E-13 | 6 | 7.65E-12 | 1.21E-12 | 4.96E-13 |
|  | KO old | 9 | 7.41E-12 | 1.16E-12 | 3.87E-13 | 9 | 5.06E-12 | 9.29E-13 | 3.10E-13 |
|  | ABHD12 KO | 15 | 8.51E-12 | 1.98E-12 | 5.10E-13 | 15 | 6.09E-12 | 1.66E-12 | 4.28E-13 |
|  | Young | 12 | 1.06E-11 | 1.57E-12 | 4.54E-13 | 12 | 7.57E-12 | 1.06E-12 | 3.05E-13 |
|  | Old | 15 | 7.58E-12 | 1.05E-12 | 2.71E-13 | 15 | 4.93E-12 | 8.22E-13 | 2.12E-13 |
| *N*-oleoyl GABA | WT young | 6 | 2.92E-12 | 3.54E-13 | 1.45E-13 | 6 | 4.19E-12 | 5.55E-13 | 2.27E-13 |
|  | WT old | 6 | 2.07E-12 | 5.76E-13 | 2.35E-13 | 6 | 2.53E-12 | 5.65E-13 | 2.31E-13 |
|  | WT | 12 | 2.49E-12 | 6.36E-13 | 1.83E-13 | 12 | 3.36E-12 | 1.02E-12 | 2.94E-13 |
|  | KO young | 6 | 2.84E-12 | 4.83E-13 | 1.97E-13 | 6 | 3.65E-12 | 7.85E-13 | 3.21E-13 |
|  | KO old | 9 | 1.81E-12 | 4.43E-13 | 1.48E-13 | 9 | 2.24E-12 | 3.28E-13 | 1.09E-13 |
|  | ABHD12 KO | 15 | 2.22E-12 | 6.82E-13 | 1.76E-13 | 15 | 2.80E-12 | 8.93E-13 | 2.30E-13 |
|  | Young | 12 | 2.88E-12 | 4.06E-13 | 1.17E-13 | 12 | 3.92E-12 | 7.06E-13 | 2.04E-13 |
|  | Old | 15 | 1.92E-12 | 4.97E-13 | 1.28E-13 | 15 | 2.35E-12 | 4.43E-13 | 1.14E-13 |
| *N*-linoleoyl GABA | WT young | 6 | PISSR |  |  | 6 | 7.72E-13 | 1.64E-13 | 6.70E-14 |
|  | WT old | 6 | PISSR |  |  | 6 | 4.98E-13 | 1.64E-13 | 6.68E-14 |
|  | WT | 12 | PISSR |  |  | 12 | 6.35E-13 | 2.12E-13 | 6.12E-14 |
|  | KO young | 6 | PISSR |  |  | 6 | 7.36E-13 | 2.95E-13 | 1.20E-13 |
|  | KO old | 9 | PISSR |  |  | 9 | 3.56E-13 | 1.17E-13 | 3.90E-14 |
|  | ABHD12 KO | 15 | PISSR |  |  | 15 | 5.08E-13 | 2.76E-13 | 7.12E-14 |
|  | Young | 12 | PISSR |  |  | 12 | 7.54E-13 | 2.28E-13 | 6.58E-14 |
|  | Old | 15 | PISSR |  |  | 15 | 4.13E-13 | 1.50E-13 | 3.88E-14 |
| *N*-arachidonoyl GABA | WT young | 6 | 1.09E-11 | 1.65E-12 | 6.76E-13 | 6 | 2.76E-11 | 4.62E-12 | 1.88E-12 |
|  | WT old | 6 | 7.16E-12 | 1.37E-12 | 5.61E-13 | 6 | 1.75E-11 | 2.67E-12 | 1.09E-12 |
|  | WT | 12 | 9.02E-12 | 2.42E-12 | 7.00E-13 | 12 | 2.25E-11 | 6.39E-12 | 1.84E-12 |
|  | KO young | 6 | 1.20E-11 | 1.82E-12 | 7.44E-13 | 6 | 2.80E-11 | 4.19E-12 | 1.71E-12 |
|  | KO old | 9 | 9.42E-12 | 1.45E-12 | 4.82E-13 | 9 | 1.75E-11 | 2.32E-12 | 7.74E-13 |
|  | ABHD12 KO | 15 | 1.05E-11 | 2.02E-12 | 5.21E-13 | 15 | 2.17E-11 | 6.18E-12 | 1.59E-12 |
|  | Young | 12 | 1.14E-11 | 1.76E-12 | 5.07E-13 | 12 | 2.78E-11 | 4.21E-12 | 1.21E-12 |
|  | Old | 15 | 8.52E-12 | 1.78E-12 | 4.61E-13 | 15 | 1.75E-11 | 2.37E-12 | 6.12E-13 |
| *N*-docosahexaenoyl GABA | WT young | 6 | 2.51E-12 | 2.96E-13 | 1.21E-13 | 6 | 1.91E-12 | 3.89E-13 | 1.59E-13 |
|  | WT old | 6 | 1.99E-12 | 4.00E-13 | 1.63E-13 | 6 | 1.16E-12 | 1.88E-13 | 7.66E-14 |
|  | WT | 12 | 2.25E-12 | 4.31E-13 | 1.25E-13 | 12 | 1.54E-12 | 4.90E-13 | 1.41E-13 |
|  | KO young | 6 | 2.17E-12 | 2.98E-13 | 1.22E-13 | 6 | 1.85E-12 | 3.02E-13 | 1.23E-13 |
|  | KO old | 9 | 1.54E-12 | 3.64E-13 | 1.21E-13 | 9 | 1.12E-12 | 2.24E-13 | 7.47E-14 |
|  | ABHD12 KO | 15 | 1.79E-12 | 4.57E-13 | 1.18E-13 | 15 | 1.41E-12 | 4.46E-13 | 1.15E-13 |
|  | Young | 12 | 2.34E-12 | 3.35E-13 | 9.66E-14 | 12 | 1.88E-12 | 3.34E-13 | 9.63E-14 |
|  | Old | 15 | 1.72E-12 | 4.30E-13 | 1.11E-13 | 15 | 1.14E-12 | 2.04E-13 | 5.27E-14 |

Supplemental Table 11: Mean levels of *N*-acyl GABAs in the cerebellum and thalamus

| Lipid Species | Group | Cerebellum | | | | Thalamus | | | |
| --- | --- | --- | --- | --- | --- | --- | --- | --- | --- |
|  |  | N | Mean | SD | SE | N | Mean | SD | SE |
| *N*-palmitoyl GABA | WT young | 6 | 1.41E-11 | 3.00E-12 | 1.22E-12 | 6 | 1.16E-11 | 1.95E-12 | 7.98E-13 |
|  | WT old | 6 | 9.48E-12 | 2.63E-12 | 1.07E-12 | 6 | 6.79E-12 | 7.62E-13 | 3.11E-13 |
|  | WT | 12 | 1.18E-11 | 3.63E-12 | 1.05E-12 | 12 | 9.19E-12 | 2.88E-12 | 8.32E-13 |
|  | KO young | 6 | 1.23E-11 | 3.14E-12 | 1.28E-12 | 6 | 1.03E-11 | 2.55E-12 | 1.04E-12 |
|  | KO old | 9 | 8.08E-12 | 2.04E-12 | 6.78E-13 | 9 | 6.53E-12 | 1.86E-12 | 6.19E-13 |
|  | ABHD12 KO | 15 | 9.75E-12 | 3.22E-12 | 8.32E-13 | 15 | 8.06E-12 | 2.83E-12 | 7.31E-13 |
|  | Young | 12 | 1.32E-11 | 3.08E-12 | 8.9E-13 | 12 | 1.10E-11 | 2.26E-12 | 6.54E-13 |
|  | Old | 15 | 8.64E-12 | 2.31E-12 | 5.97E-13 | 15 | 6.64E-12 | 1.48E-12 | 3.82E-13 |
| *N*-stearoyl GABA | WT young | 6 | 2.09E-11 | 5.90E-12 | 2.41E-12 | 6 | 1.32E-11 | 3.33E-12 | 1.36E-12 |
|  | WT old | 6 | 1.33E-11 | 1.20E-12 | 4.9E-13 | 6 | 8.85E-12 | 1.13E-12 | 4.63E-13 |
|  | WT | 12 | 1.71E-11 | 5.68E-12 | 1.64E-12 | 12 | 1.10E-11 | 3.27E-12 | 9.45E-13 |
|  | KO young | 6 | 2.02E-11 | 7.70E-12 | 3.14E-12 | 6 | 1.25E-11 | 2.61E-12 | 1.06E-12 |
|  | KO old | 9 | 1.33E-11 | 2.08E-12 | 6.92E-13 | 9 | 9.58E-12 | 3.63E-12 | 1.21E-12 |
|  | ABHD12 KO | 15 | 1.61E-11 | 5.99E-12 | 1.55E-12 | 15 | 1.07E-11 | 3.49E-12 | 9.01E-13 |
|  | Young | 12 | 2.06E-11 | 6.55E-12 | 1.89E-12 | 12 | 1.28E-11 | 2.87E-12 | 8.29E-13 |
|  | Old | 15 | 1.33E-11 | 1.73E-12 | 4.46E-13 | 15 | 9.29E-12 | 2.85E-12 | 7.37E-13 |
| *N*-oleoyl GABA | WT young | 6 | 1.02E-11 | 2.97E-12 | 1.21E-12 | 6 | 4.86E-12 | 7.79E-13 | 3.18E-13 |
|  | WT old | 6 | 6.17E-12 | 8.51E-13 | 3.48E-13 | 6 | 3.08E-12 | 3.70E-13 | 1.51E-13 |
|  | WT | 12 | 8.20E-12 | 2.98E-12 | 8.59E-13 | 12 | 3.97E-12 | 1.10E-12 | 3.17E-13 |
|  | KO young | 6 | 8.71E-12 | 3.56E-12 | 1.45E-12 | 6 | 4.87E-12 | 1.06E-12 | 4.31E-13 |
|  | KO old | 9 | 5.09E-12 | 1.09E-12 | 3.63E-13 | 9 | 3.11E-12 | 5.54E-13 | 1.85E-13 |
|  | ABHD12 KO | 15 | 6.54E-12 | 2.93E-12 | 7.55E-13 | 15 | 3.81E-12 | 1.17E-12 | 3.03E-13 |
|  | Young | 12 | 9.47E-12 | 3.23E-12 | 9.32E-13 | 12 | 4.87E-12 | 8.85E-13 | 2.55E-13 |
|  | Old | 15 | 5.52E-12 | 1.11E-12 | 2.87E-13 | 15 | 3.09E-12 | 4.74E-13 | 1.22E-13 |
| *N*-linoleoyl GABA | WT young | 6 | 1.36E-12 | 2.78E-13 | 1.14E-13 | 6 | 7.49E-13 | 8.85E-14 | 3.61E-14 |
|  | WT old | 6 | 6.93E-13 | 2.15E-13 | 8.77E-14 | 6 | 4.00E-13 | 1.22E-13 | 4.99E-14 |
|  | WT | 12 | 1.03E-12 | 4.21E-13 | 1.21E-13 | 12 | 5.74E-13 | 2.09E-13 | 6.02E-14 |
|  | KO young | 6 | 1.21E-12 | 3.79E-13 | 1.55E-13 | 6 | 6.68E-13 | 2.22E-13 | 9.08E-14 |
|  | KO old | 9 | 5.93E-13 | 3.15E-13 | 1.05E-13 | 9 | 3.61E-13 | 9.92E-14 | 3.31E-14 |
|  | ABHD12 KO | 15 | 8.39E-13 | 4.53E-13 | 1.17E-13 | 15 | 4.84E-13 | 2.18E-13 | 5.63E-14 |
|  | Young | 12 | 1.28E-12 | 3.27E-13 | 9.43E-14 | 12 | 7.08E-13 | 1.67E-13 | 4.81E-14 |
|  | Old | 15 | 6.33E-13 | 2.75E-13 | 7.11E-14 | 15 | 3.77E-13 | 1.07E-13 | 2.75E-14 |
| *N*-arachidonoyl GABA | WT young | 6 | 1.21E-11 | 1.39E-12 | 5.67E-13 | 6 | 1.16E-11 | 1.44E-12 | 5.86E-13 |
|  | WT old | 6 | 6.70E-12 | 8.08E-13 | 3.3E-13 | 6 | 5.95E-12 | 1.42E-12 | 5.82E-13 |
|  | WT | 12 | 9.42E-12 | 3.04E-12 | 8.78E-13 | 12 | 8.80E-12 | 3.27E-12 | 9.43E-13 |
|  | KO young | 6 | 1.43E-11 | 2.75E-12 | 1.12E-12 | 6 | 1.44E-11 | 2.07E-12 | 8.45E-13 |
|  | KO old | 9 | 7.79E-12 | 7.81E-13 | 2.6E-13 | 9 | 7.53E-12 | 1.77E-12 | 5.88E-13 |
|  | ABHD12 KO | 15 | 1.04E-11 | 3.75E-12 | 9.68E-13 | 15 | 1.03E-11 | 3.94E-12 | 1.02E-12 |
|  | Young | 12 | 1.32E-11 | 2.37E-12 | 6.84E-13 | 12 | 1.30E-11 | 2.24E-12 | 6.46E-13 |
|  | Old | 15 | 7.35E-12 | 9.42E-13 | 2.43E-13 | 15 | 6.90E-12 | 1.77E-12 | 4.58E-13 |
| *N*-docosahexaenoyl GABA | WT young | 6 | 3.91E-12 | 6.03E-13 | 2.46E-13 | 6 | 1.99E-12 | 5.49E-13 | 2.24E-13 |
|  | WT old | 6 | 2.59E-12 | 5.88E-13 | 2.4E-13 | 6 | 1.04E-12 | 1.20E-13 | 4.90E-14 |
|  | WT | 12 | 3.25E-12 | 8.91E-13 | 2.57E-13 | 12 | 1.52E-12 | 6.26E-13 | 1.81E-13 |
|  | KO young | 6 | 3.65E-12 | 6.10E-13 | 2.49E-13 | 6 | 2.06E-12 | 4.78E-13 | 1.95E-13 |
|  | KO old | 9 | 2.21E-12 | 4.68E-13 | 1.56E-13 | 9 | 1.32E-12 | 3.29E-13 | 1.10E-13 |
|  | ABHD12 KO | 15 | 2.79E-12 | 8.88E-13 | 2.29E-13 | 15 | 1.61E-12 | 5.33E-13 | 1.38E-13 |
|  | Young | 12 | 3.78E-12 | 5.94E-13 | 1.71E-13 | 12 | 2.03E-12 | 4.92E-13 | 1.42E-13 |
|  | Old | 15 | 2.36E-12 | 5.34E-13 | 1.38E-13 | 15 | 1.21E-12 | 2.95E-13 | 7.61E-14 |

Supplemental Table 12: Mean levels of *N*-acyl GABAs in the cortex and hypothalamus

| Lipid Species | Group | Cortex | | | | Hypothalamus | | | |
| --- | --- | --- | --- | --- | --- | --- | --- | --- | --- |
|  |  | N | Mean | SD | SE | N | Mean | SD | SE |
| *N*-palmitoyl GABA | WT young | 6 | 8.48E-12 | 3.25E-12 | 1.32E-12 | 6 | 1.04E-11 | 1.70E-12 | 6.94E-13 |
|  | WT old | 6 | 3.92E-12 | 4.30E-13 | 1.76E-13 | 5 | 8.48E-12 | 1.72E-12 | 7.69E-13 |
|  | WT | 12 | 6.20E-12 | 3.25E-12 | 9.37E-13 | 11 | 9.55E-12 | 1.92E-12 | 5.78E-13 |
|  | KO young | 6 | 6.32E-12 | 2.20E-12 | 8.99E-13 | 6 | 7.93E-12 | 2.14E-12 | 8.72E-13 |
|  | KO old | 9 | 3.51E-12 | 4.52E-13 | 1.51E-13 | 7 | 6.76E-12 | 1.26E-12 | 4.76E-13 |
|  | ABHD12 KO | 15 | 4.63E-12 | 1.97E-12 | 5.08E-13 | 13 | 7.30E-12 | 1.75E-12 | 4.85E-13 |
|  | Young | 12 | 7.40E-12 | 2.88E-12 | 8.3E-13 | 12 | 9.19E-12 | 2.26E-12 | 6.53E-13 |
|  | Old | 15 | 3.67E-12 | 4.76E-13 | 1.23E-13 | 12 | 7.48E-12 | 1.65E-12 | 4.76E-13 |
| *N*-stearoyl GABA | WT young | 6 | 1.36E-11 | 3.64E-12 | 1.48E-12 | 6 | 1.24E-11 | 1.96E-12 | 8.01E-13 |
|  | WT old | 6 | 7.81E-12 | 1.05E-12 | 4.29E-13 | 5 | 9.20E-12 | 1.53E-12 | 6.85E-13 |
|  | WT | 12 | 1.07E-11 | 3.94E-12 | 1.14E-12 | 11 | 1.10E-11 | 2.38E-12 | 7.19E-13 |
|  | KO young | 6 | 1.29E-11 | 4.25E-12 | 1.73E-12 | 6 | 8.89E-12 | 2.37E-12 | 9.70E-13 |
|  | KO old | 9 | 7.78E-12 | 1.07E-12 | 3.56E-13 | 7 | 8.62E-12 | 9.26E-13 | 3.50E-13 |
|  | ABHD12 KO | 15 | 9.82E-12 | 3.71E-12 | 9.57E-13 | 13 | 8.75E-12 | 1.67E-12 | 4.64E-13 |
|  | Young | 12 | 1.32E-11 | 3.79E-12 | 1.09E-12 | 12 | 1.07E-11 | 2.77E-12 | 8.01E-13 |
|  | Old | 15 | 7.79E-12 | 1.02E-12 | 2.64E-13 | 12 | 8.86E-12 | 1.19E-12 | 3.43E-13 |
| *N*-oleoyl GABA | WT young | 6 | 5.53E-12 | 1.67E-12 | 6.82E-13 | 6 | 3.45E-12 | 7.28E-13 | 2.97E-13 |
|  | WT old | 6 | 2.81E-12 | 2.11E-13 | 8.63E-14 | 5 | 2.82E-12 | 5.15E-13 | 2.30E-13 |
|  | WT | 12 | 4.17E-12 | 1.82E-12 | 5.25E-13 | 11 | 3.16E-12 | 6.93E-13 | 2.09E-13 |
|  | KO young | 6 | 4.70E-12 | 1.59E-12 | 6.5E-13 | 6 | 2.47E-12 | 9.26E-13 | 3.78E-13 |
|  | KO old | 9 | 2.67E-12 | 3.29E-13 | 1.1E-13 | 7 | 2.37E-12 | 5.45E-13 | 2.06E-13 |
|  | ABHD12 KO | 15 | 3.48E-12 | 1.42E-12 | 3.67E-13 | 13 | 2.42E-12 | 7.13E-13 | 1.98E-13 |
|  | Young | 12 | 5.11E-12 | 1.62E-12 | 4.66E-13 | 12 | 2.96E-12 | 9.44E-13 | 2.73E-13 |
|  | Old | 15 | 2.73E-12 | 2.88E-13 | 7.44E-14 | 12 | 2.55E-12 | 5.59E-13 | 1.61E-13 |
| *N*-linoleoyl GABA | WT young | 6 | 7.93E-13 | 2.97E-13 | 1.21E-13 | 6 | PISSR |  |  |
|  | WT old | 6 | 2.81E-13 | 1.33E-13 | 5.44E-14 | 5 | PISSR |  |  |
|  | WT | 12 | 5.37E-13 | 3.46E-13 | 9.98E-14 | 11 | PISSR |  |  |
|  | KO young | 6 | 6.48E-13 | 2.58E-13 | 1.05E-13 | 6 | PISSR |  |  |
|  | KO old | 9 | 3.34E-13 | 6.13E-14 | 2.04E-14 | 7 | PISSR |  |  |
|  | ABHD12 KO | 15 | 4.60E-13 | 2.26E-13 | 5.84E-14 | 13 | PISSR |  |  |
|  | Young | 12 | 7.21E-13 | 2.76E-13 | 7.96E-14 | 12 | PISSR |  |  |
|  | Old | 15 | 3.13E-13 | 9.60E-14 | 2.48E-14 | 12 | PISSR |  |  |
| *N*-arachidonoyl GABA | WT young | 6 | 1.31E-11 | 2.80E-12 | 1.14E-12 | 6 | 1.19E-11 | 2.18E-12 | 8.91E-13 |
|  | WT old | 6 | 7.13E-12 | 5.09E-13 | 2.08E-13 | 5 | 9.41E-12 | 2.01E-12 | 9.00E-13 |
|  | WT | 12 | 1.01E-11 | 3.66E-12 | 1.06E-12 | 11 | 1.08E-11 | 2.40E-12 | 7.22E-13 |
|  | KO young | 6 | 1.62E-11 | 4.49E-12 | 1.83E-12 | 6 | 1.06E-11 | 4.06E-12 | 1.66E-12 |
|  | KO old | 9 | 8.58E-12 | 6.60E-13 | 2.2E-13 | 7 | 8.96E-12 | 1.54E-12 | 5.80E-13 |
|  | ABHD12 KO | 15 | 1.16E-11 | 4.75E-12 | 1.23E-12 | 13 | 9.70E-12 | 2.96E-12 | 8.20E-13 |
|  | Young | 12 | 1.47E-11 | 3.93E-12 | 1.13E-12 | 12 | 1.13E-11 | 3.19E-12 | 9.20E-13 |
|  | Old | 15 | 8.00E-12 | 9.43E-13 | 2.43E-13 | 12 | 9.15E-12 | 1.68E-12 | 4.84E-13 |
| *N*-docosahexaenoyl GABA | WT young | 6 | 2.86E-12 | 8.22E-13 | 3.36E-13 | 6 | 1.68E-12 | 5.06E-13 | 2.07E-13 |
|  | WT old | 6 | 1.54E-12 | 2.30E-13 | 9.4E-14 | 5 | 1.27E-12 | 3.88E-13 | 1.74E-13 |
|  | WT | 12 | 2.20E-12 | 8.98E-13 | 2.59E-13 | 11 | 1.49E-12 | 4.85E-13 | 1.46E-13 |
|  | KO young | 6 | 2.63E-12 | 8.72E-13 | 3.56E-13 | 6 | 1.33E-12 | 4.45E-13 | 1.82E-13 |
|  | KO old | 9 | 1.42E-12 | 2.59E-13 | 8.63E-14 | 7 | 1.05E-12 | 2.57E-13 | 9.71E-14 |
|  | ABHD12 KO | 15 | 1.90E-12 | 8.27E-13 | 2.13E-13 | 13 | 1.18E-12 | 3.69E-13 | 1.02E-13 |
|  | Young | 12 | 2.75E-12 | 8.17E-13 | 2.36E-13 | 12 | 1.51E-12 | 4.90E-13 | 1.41E-13 |
|  | Old | 15 | 1.47E-12 | 2.47E-13 | 6.39E-14 | 12 | 1.14E-12 | 3.21E-13 | 9.26E-14 |

Supplemental Table 13: Mean levels of *N*-acyl GABAs in the midbrain and brainstem

| Lipid Species | Group | Midbrain | | | | Brainstem | | | |
| --- | --- | --- | --- | --- | --- | --- | --- | --- | --- |
|  |  | N | Mean | SD | SE | N | Mean | SD | SE |
| *N*-palmitoyl GABA | WT young | 6 | 1.04E-11 | 1.33E-12 | 5.44E-13 | 6 | 7.56E-12 | 1.08E-12 | 4.40E-13 |
|  | WT old | 6 | 6.46E-12 | 8.76E-13 | 3.57E-13 | 6 | 4.85E-12 | 3.34E-13 | 1.36E-13 |
|  | WT | 12 | 8.42E-12 | 2.32E-12 | 6.68E-13 | 12 | 6.21E-12 | 1.61E-12 | 4.63E-13 |
|  | KO young | 6 | 8.91E-12 | 1.98E-12 | 8.1E-13 | 6 | 7.13E-12 | 1.19E-12 | 4.87E-13 |
|  | KO old | 9 | 5.89E-12 | 7.00E-13 | 2.33E-13 | 9 | 4.03E-12 | 5.02E-13 | 1.67E-13 |
|  | ABHD12 KO | 15 | 7.10E-12 | 2.01E-12 | 5.18E-13 | 15 | 5.27E-12 | 1.77E-12 | 4.56E-13 |
|  | Young | 12 | 9.65E-12 | 1.79E-12 | 5.15E-13 | 12 | 7.35E-12 | 1.11E-12 | 3.20E-13 |
|  | Old | 15 | 6.12E-12 | 7.98E-13 | 2.06E-13 | 15 | 4.36E-12 | 6.00E-13 | 1.55E-13 |
| *N*-stearoyl GABA | WT young | 6 | 1.39E-11 | 2.60E-12 | 1.06E-12 | 6 | 9.75E-12 | 1.29E-12 | 5.25E-13 |
|  | WT old | 6 | 9.07E-12 | 1.31E-12 | 5.36E-13 | 6 | 7.09E-12 | 3.81E-13 | 1.56E-13 |
|  | WT | 12 | 1.15E-11 | 3.20E-12 | 9.23E-13 | 12 | 8.42E-12 | 1.66E-12 | 4.79E-13 |
|  | KO young | 6 | 1.32E-11 | 2.90E-12 | 1.19E-12 | 6 | 9.73E-12 | 1.11E-12 | 4.53E-13 |
|  | KO old | 9 | 8.74E-12 | 1.12E-12 | 3.72E-13 | 9 | 6.10E-12 | 3.94E-13 | 1.31E-13 |
|  | ABHD12 KO | 15 | 1.05E-11 | 2.98E-12 | 7.69E-13 | 15 | 7.55E-12 | 1.98E-12 | 5.10E-13 |
|  | Young | 12 | 1.36E-11 | 2.65E-12 | 7.65E-13 | 12 | 9.74E-12 | 1.15E-12 | 3.31E-13 |
|  | Old | 15 | 8.87E-12 | 1.16E-12 | 3.01E-13 | 15 | 6.50E-12 | 6.25E-13 | 1.61E-13 |
| *N*-oleoyl GABA | WT young | 6 | 5.87E-12 | 7.05E-13 | 2.88E-13 | 6 | 5.64E-12 | 8.64E-13 | 3.53E-13 |
|  | WT old | 6 | 3.23E-12 | 5.49E-13 | 2.24E-13 | 6 | 3.71E-12 | 2.51E-13 | 1.03E-13 |
|  | WT | 12 | 4.55E-12 | 1.51E-12 | 4.35E-13 | 12 | 4.67E-12 | 1.18E-12 | 3.40E-13 |
|  | KO young | 6 | 5.20E-12 | 1.36E-12 | 5.56E-13 | 6 | 5.91E-12 | 1.08E-12 | 4.40E-13 |
|  | KO old | 9 | 3.11E-12 | 3.55E-13 | 1.18E-13 | 9 | 2.91E-12 | 3.97E-13 | 1.32E-13 |
|  | ABHD12 KO | 15 | 3.94E-12 | 1.36E-12 | 3.52E-13 | 15 | 4.11E-12 | 1.68E-12 | 4.34E-13 |
|  | Young | 12 | 5.53E-12 | 1.09E-12 | 3.15E-13 | 12 | 5.78E-12 | 9.42E-13 | 2.72E-13 |
|  | Old | 15 | 3.15E-12 | 4.28E-13 | 1.11E-13 | 15 | 3.23E-12 | 5.25E-13 | 1.36E-13 |
| *N*-linoleoyl GABA | WT young | 6 | 8.07E-13 | 1.12E-13 | 4.58E-14 | 6 | 6.04E-13 | 1.00E-13 | 4.08E-14 |
|  | WT old | 6 | 3.70E-13 | 1.05E-13 | 4.28E-14 | 6 | 3.30E-13 | 1.35E-13 | 5.51E-14 |
|  | WT | 12 | 5.89E-13 | 2.51E-13 | 7.23E-14 | 12 | 4.67E-13 | 1.82E-13 | 5.27E-14 |
|  | KO young | 6 | 8.21E-13 | 3.43E-13 | 1.4E-13 | 6 | 5.78E-13 | 1.59E-13 | 6.49E-14 |
|  | KO old | 9 | 3.71E-13 | 1.38E-13 | 4.61E-14 | 9 | 2.95E-13 | 9.39E-14 | 3.13E-14 |
|  | ABHD12 KO | 15 | 5.51E-13 | 3.24E-13 | 8.36E-14 | 15 | 4.08E-13 | 1.86E-13 | 4.81E-14 |
|  | Young | 12 | 8.14E-13 | 2.43E-13 | 7.02E-14 | 12 | 5.91E-13 | 1.27E-13 | 3.67E-14 |
|  | Old | 15 | 3.71E-13 | 1.22E-13 | 3.15E-14 | 15 | 3.09E-13 | 1.09E-13 | 2.81E-14 |
| *N*-arachidonoyl GABA | WT young | 6 | 1.27E-11 | 9.57E-13 | 3.91E-13 | 6 | 4.55E-12 | 2.97E-13 | 1.21E-13 |
|  | WT old | 6 | 7.31E-12 | 8.61E-13 | 3.52E-13 | 6 | 2.80E-12 | 4.24E-13 | 1.73E-13 |
|  | WT | 12 | 1.00E-11 | 2.94E-12 | 8.49E-13 | 12 | 3.67E-12 | 9.81E-13 | 2.83E-13 |
|  | KO young | 6 | 1.60E-11 | 3.30E-12 | 1.35E-12 | 6 | 6.10E-12 | 7.63E-13 | 3.11E-13 |
|  | KO old | 9 | 8.99E-12 | 7.97E-13 | 2.66E-13 | 9 | 3.60E-12 | 4.13E-13 | 1.38E-13 |
|  | ABHD12 KO | 15 | 1.18E-11 | 4.09E-12 | 1.06E-12 | 15 | 4.60E-12 | 1.38E-12 | 3.56E-13 |
|  | Young | 12 | 1.43E-11 | 2.87E-12 | 8.3E-13 | 12 | 5.32E-12 | 9.79E-13 | 2.83E-13 |
|  | Old | 15 | 8.32E-12 | 1.16E-12 | 3E-13 | 15 | 3.28E-12 | 5.74E-13 | 1.48E-13 |
| *N*-docosahexaenoyl GABA | WT young | 6 | 2.21E-12 | 4.32E-13 | 1.76E-13 | 6 | 1.49E-12 | 2.93E-13 | 1.19E-13 |
|  | WT old | 6 | 1.39E-12 | 6.65E-14 | 2.71E-14 | 6 | 9.47E-13 | 6.70E-14 | 2.74E-14 |
|  | WT | 12 | 1.80E-12 | 5.23E-13 | 1.51E-13 | 12 | 1.22E-12 | 3.49E-13 | 1.01E-13 |
|  | KO young | 6 | 2.41E-12 | 9.38E-13 | 3.83E-13 | 6 | 1.55E-12 | 3.95E-13 | 1.61E-13 |
|  | KO old | 9 | 1.34E-12 | 1.78E-13 | 5.94E-14 | 9 | 8.43E-13 | 1.44E-13 | 4.81E-14 |
|  | ABHD12 KO | 15 | 1.77E-12 | 7.92E-13 | 2.05E-13 | 15 | 1.13E-12 | 4.42E-13 | 1.14E-13 |
|  | Young | 12 | 2.31E-12 | 7.04E-13 | 2.03E-13 | 12 | 1.52E-12 | 3.33E-13 | 9.61E-14 |
|  | Old | 15 | 1.36E-12 | 1.42E-13 | 3.68E-14 | 15 | 8.85E-13 | 1.28E-13 | 3.30E-14 |

Supplemental Table 14: Mean levels of *N*-acyl glycines in the striatum and hippocampus

| Lipid Species | Group | Striatum | | | | Hippocampus | | | |
| --- | --- | --- | --- | --- | --- | --- | --- | --- | --- |
|  |  | N | Mean | SD | SE | N | Mean | SD | SE |
| *N*-palmitoyl glycine | WT young | 6 | 7.90E-11 | 5.86E-12 | 2.39E-12 | 6 | 3.94E-11 | 3.83E-12 | 1.56E-12 |
|  | WT old | 6 | 7.20E-11 | 1.04E-11 | 4.23E-12 | 6 | 3.28E-11 | 1.92E-12 | 7.85E-13 |
|  | WT | 12 | 7.55E-11 | 8.80E-12 | 2.54E-12 | 12 | 3.61E-11 | 4.48E-12 | 1.29E-12 |
|  | KO young | 6 | 7.16E-11 | 1.21E-11 | 4.94E-12 | 6 | 3.65E-11 | 5.38E-12 | 2.19E-12 |
|  | KO old | 9 | 7.10E-11 | 8.46E-12 | 2.82E-12 | 9 | 3.42E-11 | 3.86E-12 | 1.29E-12 |
|  | ABHD12 KO | 15 | 7.12E-11 | 9.66E-12 | 2.49E-12 | 15 | 3.51E-11 | 4.50E-12 | 1.16E-12 |
|  | Young | 12 | 7.53E-11 | 9.85E-12 | 2.84E-12 | 12 | 3.79E-11 | 4.69E-12 | 1.35E-12 |
|  | Old | 15 | 7.14E-11 | 8.92E-12 | 2.30E-12 | 15 | 3.36E-11 | 3.21E-12 | 8.29E-13 |
| *N*-stearoyl glycine | WT young | 6 | 3.20E-11 | 3.31E-12 | 1.35E-12 | 6 | 1.96E-11 | 3.11E-12 | 1.27E-12 |
|  | WT old | 6 | 2.82E-11 | 6.32E-12 | 2.58E-12 | 6 | 1.56E-11 | 1.23E-12 | 5.04E-13 |
|  | WT | 12 | 3.01E-11 | 5.21E-12 | 1.50E-12 | 12 | 1.76E-11 | 3.06E-12 | 8.83E-13 |
|  | KO young | 6 | 3.24E-11 | 6.00E-12 | 2.45E-12 | 6 | 2.00E-11 | 2.86E-12 | 1.17E-12 |
|  | KO old | 9 | 2.84E-11 | 3.57E-12 | 1.19E-12 | 9 | 1.57E-11 | 2.23E-12 | 7.44E-13 |
|  | ABHD12 KO | 15 | 3.00E-11 | 4.93E-12 | 1.27E-12 | 15 | 1.74E-11 | 3.24E-12 | 8.37E-13 |
|  | Young | 12 | 3.22E-11 | 4.62E-12 | 1.33E-12 | 12 | 1.98E-11 | 2.86E-12 | 8.25E-13 |
|  | Old | 15 | 2.83E-11 | 4.64E-12 | 1.20E-12 | 15 | 1.57E-11 | 1.84E-12 | 4.76E-13 |
| *N*-oleoyl glycine | WT young | 6 | 2.92E-11 | 2.59E-12 | 1.06E-12 | 6 | 1.99E-11 | 1.89E-12 | 7.70E-13 |
|  | WT old | 6 | 2.48E-11 | 3.22E-12 | 1.32E-12 | 6 | 1.58E-11 | 8.81E-13 | 3.60E-13 |
|  | WT | 12 | 2.70E-11 | 3.62E-12 | 1.04E-12 | 12 | 1.78E-11 | 2.56E-12 | 7.39E-13 |
|  | KO young | 6 | 2.74E-11 | 4.83E-12 | 1.97E-12 | 6 | 1.86E-11 | 2.66E-12 | 1.08E-12 |
|  | KO old | 9 | 2.56E-11 | 3.22E-12 | 1.07E-12 | 9 | 1.53E-11 | 1.27E-12 | 4.25E-13 |
|  | ABHD12 KO | 15 | 2.63E-11 | 3.89E-12 | 1.00E-12 | 15 | 1.66E-11 | 2.50E-12 | 6.46E-13 |
|  | Young | 12 | 2.83E-11 | 3.82E-12 | 1.10E-12 | 12 | 1.92E-11 | 2.29E-12 | 6.62E-13 |
|  | Old | 15 | 2.53E-11 | 3.13E-12 | 8.08E-13 | 15 | 1.55E-11 | 1.12E-12 | 2.90E-13 |
| *N*-linoleoyl glycine | WT young | 6 | 4.84E-12 | 7.34E-13 | 3.00E-13 | 6 | 3.70E-12 | 5.34E-13 | 2.18E-13 |
|  | WT old | 6 | 3.58E-12 | 1.42E-12 | 5.78E-13 | 6 | 2.56E-12 | 5.92E-13 | 2.42E-13 |
|  | WT | 12 | 4.21E-12 | 1.26E-12 | 3.64E-13 | 12 | 3.13E-12 | 8.01E-13 | 2.31E-13 |
|  | KO young | 6 | 4.27E-12 | 1.29E-12 | 5.27E-13 | 6 | 3.46E-12 | 6.50E-13 | 2.65E-13 |
|  | KO old | 9 | 3.93E-12 | 5.34E-13 | 1.78E-13 | 9 | 2.53E-12 | 4.89E-13 | 1.63E-13 |
|  | ABHD12 KO | 15 | 4.06E-12 | 8.88E-13 | 2.29E-13 | 15 | 2.90E-12 | 7.15E-13 | 1.85E-13 |
|  | Young | 12 | 4.55E-12 | 1.04E-12 | 3.02E-13 | 12 | 3.58E-12 | 5.80E-13 | 1.68E-13 |
|  | Old | 15 | 3.79E-12 | 9.54E-13 | 2.46E-13 | 15 | 2.54E-12 | 5.12E-13 | 1.32E-13 |
| *N*-arachidonoyl glycine | WT young | 6 | 5.38E-11 | 3.56E-12 | 1.45E-12 | 6 | 5.02E-11 | 7.93E-12 | 3.24E-12 |
|  | WT old | 6 | 4.59E-11 | 6.38E-12 | 2.60E-12 | 6 | 3.77E-11 | 2.04E-12 | 8.31E-13 |
|  | WT | 12 | 4.99E-11 | 6.43E-12 | 1.86E-12 | 12 | 4.40E-11 | 8.55E-12 | 2.47E-12 |
|  | KO young | 6 | 5.09E-11 | 8.05E-12 | 3.29E-12 | 6 | 5.33E-11 | 6.89E-12 | 2.81E-12 |
|  | KO old | 9 | 4.66E-11 | 5.00E-12 | 1.67E-12 | 9 | 4.05E-11 | 3.55E-12 | 1.18E-12 |
|  | ABHD12 KO | 15 | 4.83E-11 | 6.49E-12 | 1.68E-12 | 15 | 4.56E-11 | 8.13E-12 | 2.10E-12 |
|  | Young | 12 | 5.24E-11 | 6.13E-12 | 1.77E-12 | 12 | 5.18E-11 | 7.26E-12 | 2.10E-12 |
|  | Old | 15 | 4.63E-11 | 5.38E-12 | 1.39E-12 | 15 | 3.94E-11 | 3.28E-12 | 8.46E-13 |
| *N*-docosahexaenoyl glycine | WT young | 6 | 2.42E-11 | 9.21E-13 | 3.76E-13 | 6 | 1.61E-11 | 2.71E-12 | 1.11E-12 |
|  | WT old | 6 | 2.19E-11 | 2.63E-12 | 1.07E-12 | 6 | 1.25E-11 | 6.58E-13 | 2.69E-13 |
|  | WT | 12 | 2.31E-11 | 2.23E-12 | 6.45E-13 | 12 | 1.43E-11 | 2.66E-12 | 7.67E-13 |
|  | KO young | 6 | 2.20E-11 | 3.61E-12 | 1.47E-12 | 6 | 1.77E-11 | 2.83E-12 | 1.16E-12 |
|  | KO old | 9 | 2.20E-11 | 4.06E-12 | 1.35E-12 | 9 | 1.27E-11 | 1.17E-12 | 3.90E-13 |
|  | ABHD12 KO | 15 | 2.20E-11 | 3.75E-12 | 9.69E-13 | 15 | 1.47E-11 | 3.17E-12 | 8.19E-13 |
|  | Young | 12 | 2.31E-11 | 2.76E-12 | 7.98E-13 | 12 | 1.69E-11 | 2.77E-12 | 7.99E-13 |
|  | Old | 15 | 2.20E-11 | 3.45E-12 | 8.91E-13 | 15 | 1.26E-11 | 9.72E-13 | 2.51E-13 |

Supplemental Table 15: Mean levels of *N*-acyl glycines in the cerebellum and thalamus

| Lipid Species | Group | Cerebellum | | | | Thalamus | | | |
| --- | --- | --- | --- | --- | --- | --- | --- | --- | --- |
|  |  | N | Mean | SD | SE | N | Mean | SD | SE |
| *N*-palmitoyl glycine | WT young | 6 | 6.70E-11 | 6.09E-12 | 2.49E-12 | 6 | 4.47E-11 | 4.27E-12 | 1.75E-12 |
|  | WT old | 6 | 5.90E-11 | 5.56E-12 | 2.27E-12 | 6 | 3.99E-11 | 5.06E-12 | 2.07E-12 |
|  | WT | 12 | 6.30E-11 | 6.95E-12 | 2.01E-12 | 12 | 4.23E-11 | 5.14E-12 | 1.48E-12 |
|  | KO young | 6 | 6.45E-11 | 5.77E-12 | 2.35E-12 | 6 | 4.66E-11 | 7.50E-12 | 3.06E-12 |
|  | KO old | 9 | 5.69E-11 | 5.73E-12 | 1.91E-12 | 9 | 4.66E-11 | 8.41E-12 | 2.80E-12 |
|  | ABHD12 KO | 15 | 5.99E-11 | 6.73E-12 | 1.74E-12 | 15 | 4.66E-11 | 7.78E-12 | 2.01E-12 |
|  | Young | 12 | 6.57E-11 | 5.80E-12 | 1.68E-12 | 12 | 4.56E-11 | 5.90E-12 | 1.70E-12 |
|  | Old | 15 | 5.78E-11 | 5.56E-12 | 1.44E-12 | 15 | 4.39E-11 | 7.84E-12 | 2.02E-12 |
| *N*-stearoyl glycine | WT young | 6 | 4.15E-11 | 5.87E-12 | 2.40E-12 | 6 | 2.52E-11 | 3.46E-12 | 1.41E-12 |
|  | WT old | 6 | 3.62E-11 | 2.80E-12 | 1.14E-12 | 6 | 2.09E-11 | 1.72E-12 | 7.04E-13 |
|  | WT | 12 | 3.88E-11 | 5.16E-12 | 1.49E-12 | 12 | 2.31E-11 | 3.44E-12 | 9.93E-13 |
|  | KO young | 6 | 4.01E-11 | 8.30E-12 | 3.39E-12 | 6 | 2.67E-11 | 3.93E-12 | 1.60E-12 |
|  | KO old | 9 | 3.57E-11 | 3.56E-12 | 1.19E-12 | 9 | 2.28E-11 | 3.86E-12 | 1.29E-12 |
|  | ABHD12 KO | 15 | 3.74E-11 | 6.08E-12 | 1.57E-12 | 15 | 2.44E-11 | 4.24E-12 | 1.09E-12 |
|  | Young | 12 | 4.08E-11 | 6.89E-12 | 1.99E-12 | 12 | 2.60E-11 | 3.62E-12 | 1.04E-12 |
|  | Old | 15 | 3.59E-11 | 3.18E-12 | 8.22E-13 | 15 | 2.21E-11 | 3.24E-12 | 8.37E-13 |
| *N*-oleoyl glycine | WT young | 6 | 3.23E-11 | 4.28E-12 | 1.75E-12 | 6 | 2.19E-11 | 1.26E-12 | 5.14E-13 |
|  | WT old | 6 | 2.93E-11 | 2.34E-12 | 9.57E-13 | 6 | 1.88E-11 | 1.65E-12 | 6.72E-13 |
|  | WT | 12 | 3.08E-11 | 3.64E-12 | 1.05E-12 | 12 | 2.03E-11 | 2.13E-12 | 6.15E-13 |
|  | KO young | 6 | 3.00E-11 | 5.01E-12 | 2.05E-12 | 6 | 2.30E-11 | 3.12E-12 | 1.27E-12 |
|  | KO old | 9 | 2.71E-11 | 3.04E-12 | 1.01E-12 | 9 | 2.05E-11 | 2.87E-12 | 9.57E-13 |
|  | ABHD12 KO | 15 | 2.82E-11 | 4.05E-12 | 1.05E-12 | 15 | 2.15E-11 | 3.13E-12 | 8.07E-13 |
|  | Young | 12 | 3.11E-11 | 4.61E-12 | 1.33E-12 | 12 | 2.24E-11 | 2.34E-12 | 6.76E-13 |
|  | Old | 15 | 2.80E-11 | 2.92E-12 | 7.54E-13 | 15 | 1.98E-11 | 2.54E-12 | 6.55E-13 |
| *N*-linoleoyl glycine | WT young | 6 | 4.90E-12 | 6.33E-13 | 2.58E-13 | 6 | 2.96E-12 | 4.59E-13 | 1.87E-13 |
|  | WT old | 6 | 4.94E-12 | 1.06E-12 | 4.35E-13 | 6 | 2.24E-12 | 1.55E-13 | 6.33E-14 |
|  | WT | 12 | 4.92E-12 | 8.35E-13 | 2.41E-13 | 12 | 2.60E-12 | 4.97E-13 | 1.43E-13 |
|  | KO young | 6 | 5.13E-12 | 9.14E-13 | 3.73E-13 | 6 | 3.23E-12 | 4.71E-13 | 1.92E-13 |
|  | KO old | 9 | 4.85E-12 | 5.95E-13 | 1.98E-13 | 9 | 2.44E-12 | 6.54E-13 | 2.18E-13 |
|  | ABHD12 KO | 15 | 4.96E-12 | 7.22E-13 | 1.86E-13 | 15 | 2.75E-12 | 6.96E-13 | 1.80E-13 |
|  | Young | 12 | 5.01E-12 | 7.59E-13 | 2.19E-13 | 12 | 3.10E-12 | 4.65E-13 | 1.34E-13 |
|  | Old | 15 | 4.89E-12 | 7.81E-13 | 2.02E-13 | 15 | 2.36E-12 | 5.13E-13 | 1.32E-13 |
| *N*-arachidonoyl glycine | WT young | 6 | 4.71E-11 | 1.16E-12 | 4.72E-13 | 6 | 2.71E-11 | 1.30E-12 | 5.30E-13 |
|  | WT old | 6 | 4.39E-11 | 2.50E-12 | 1.02E-12 | 6 | 2.31E-11 | 3.22E-12 | 1.31E-12 |
|  | WT | 12 | 4.55E-11 | 2.48E-12 | 7.16E-13 | 12 | 2.51E-11 | 3.15E-12 | 9.09E-13 |
|  | KO young | 6 | 4.89E-11 | 6.54E-12 | 2.67E-12 | 6 | 3.16E-11 | 4.78E-12 | 1.95E-12 |
|  | KO old | 9 | 4.65E-11 | 5.00E-12 | 1.67E-12 | 9 | 2.64E-11 | 3.70E-12 | 1.23E-12 |
|  | ABHD12 KO | 15 | 4.75E-11 | 5.57E-12 | 1.44E-12 | 15 | 2.85E-11 | 4.78E-12 | 1.24E-12 |
|  | Young | 12 | 4.80E-11 | 4.58E-12 | 1.32E-12 | 12 | 2.94E-11 | 4.08E-12 | 1.18E-12 |
|  | Old | 15 | 4.55E-11 | 4.27E-12 | 1.10E-12 | 15 | 2.51E-11 | 3.80E-12 | 9.80E-13 |
| *N*-docosahexaenoyl glycine | WT young | 6 | 3.18E-11 | 2.80E-12 | 1.14E-12 | 6 | 1.31E-11 | 1.14E-12 | 4.64E-13 |
|  | WT old | 6 | 2.95E-11 | 2.40E-12 | 9.79E-13 | 6 | 1.13E-11 | 1.61E-12 | 6.58E-13 |
|  | WT | 12 | 3.06E-11 | 2.76E-12 | 7.98E-13 | 12 | 1.22E-11 | 1.63E-12 | 4.71E-13 |
|  | KO young | 6 | 2.95E-11 | 3.73E-12 | 1.52E-12 | 6 | 1.44E-11 | 2.43E-12 | 9.94E-13 |
|  | KO old | 9 | 2.98E-11 | 2.76E-12 | 9.19E-13 | 9 | 1.20E-11 | 2.00E-12 | 6.65E-13 |
|  | ABHD12 KO | 15 | 2.96E-11 | 3.06E-12 | 7.89E-13 | 15 | 1.30E-11 | 2.41E-12 | 6.21E-13 |
|  | Young | 12 | 3.06E-11 | 3.37E-12 | 9.74E-13 | 12 | 1.37E-11 | 1.94E-12 | 5.59E-13 |
|  | Old | 15 | 2.97E-11 | 2.53E-12 | 6.54E-13 | 15 | 1.17E-11 | 1.83E-12 | 4.74E-13 |

Supplemental Table 16: Mean levels of *N*-acyl glycines in the cortex and hypothalamus

| Lipid Species | Group | Cortex | | | | Hypothalamus | | | |
| --- | --- | --- | --- | --- | --- | --- | --- | --- | --- |
|  |  | N | Mean | SD | SE | N | Mean | SD | SE |
| *N*-palmitoyl glycine | WT young | 6 | 3.60E-11 | 7.70E-12 | 3.14E-12 | 6 | 3.95E-11 | 8.17E-12 | 3.33E-12 |
|  | WT old | 6 | 2.79E-11 | 1.75E-12 | 7.13E-13 | 5 | 3.35E-11 | 6.66E-12 | 2.98E-12 |
|  | WT | 12 | 3.19E-11 | 6.80E-12 | 1.96E-12 | 11 | 3.68E-11 | 7.79E-12 | 2.35E-12 |
|  | KO young | 6 | 3.56E-11 | 5.72E-12 | 2.34E-12 | 6 | 3.26E-11 | 5.41E-12 | 2.21E-12 |
|  | KO old | 9 | 2.89E-11 | 2.17E-12 | 7.23E-13 | 7 | 2.90E-11 | 2.62E-12 | 9.92E-13 |
|  | ABHD12 KO | 15 | 3.16E-11 | 5.08E-12 | 1.31E-12 | 13 | 3.07E-11 | 4.36E-12 | 1.21E-12 |
|  | Young | 12 | 3.58E-11 | 6.47E-12 | 1.87E-12 | 12 | 3.60E-11 | 7.52E-12 | 2.17E-12 |
|  | Old | 15 | 2.85E-11 | 2.01E-12 | 5.20E-13 | 12 | 3.09E-11 | 5.02E-12 | 1.45E-12 |
| *N*-stearoyl glycine | WT young | 6 | 2.47E-11 | 6.21E-12 | 2.54E-12 | 6 | 2.36E-11 | 6.11E-12 | 2.50E-12 |
|  | WT old | 6 | 1.72E-11 | 1.10E-12 | 4.49E-13 | 5 | 2.02E-11 | 4.42E-12 | 1.98E-12 |
|  | WT | 12 | 2.09E-11 | 5.81E-12 | 1.68E-12 | 11 | 2.20E-11 | 5.45E-12 | 1.64E-12 |
|  | KO young | 6 | 2.54E-11 | 5.29E-12 | 2.16E-12 | 6 | 1.86E-11 | 2.89E-12 | 1.18E-12 |
|  | KO old | 9 | 1.91E-11 | 1.95E-12 | 6.49E-13 | 7 | 1.72E-11 | 3.28E-12 | 1.24E-12 |
|  | ABHD12 KO | 15 | 2.17E-11 | 4.73E-12 | 1.22E-12 | 13 | 1.78E-11 | 3.06E-12 | 8.50E-13 |
|  | Young | 12 | 2.51E-11 | 5.51E-12 | 1.59E-12 | 12 | 2.11E-11 | 5.26E-12 | 1.52E-12 |
|  | Old | 15 | 1.83E-11 | 1.90E-12 | 4.91E-13 | 12 | 1.84E-11 | 3.92E-12 | 1.13E-12 |
| *N*-oleoyl glycine | WT young | 6 | 1.80E-11 | 3.71E-12 | 1.51E-12 | 6 | 1.24E-11 | 2.66E-12 | 1.09E-12 |
|  | WT old | 6 | 1.31E-11 | 7.84E-13 | 3.20E-13 | 5 | 9.56E-12 | 1.46E-12 | 6.54E-13 |
|  | WT | 12 | 1.56E-11 | 3.60E-12 | 1.04E-12 | 11 | 1.11E-11 | 2.56E-12 | 7.71E-13 |
|  | KO young | 6 | 1.80E-11 | 3.12E-12 | 1.27E-12 | 6 | 9.96E-12 | 1.97E-12 | 8.06E-13 |
|  | KO old | 9 | 1.40E-11 | 1.10E-12 | 3.68E-13 | 7 | 8.49E-12 | 1.39E-12 | 5.25E-13 |
|  | ABHD12 KO | 15 | 1.56E-11 | 2.91E-12 | 7.51E-13 | 13 | 9.17E-12 | 1.78E-12 | 4.94E-13 |
|  | Young | 12 | 1.80E-11 | 3.27E-12 | 9.43E-13 | 12 | 1.12E-11 | 2.56E-12 | 7.40E-13 |
|  | Old | 15 | 1.36E-11 | 1.04E-12 | 2.70E-13 | 12 | 8.93E-12 | 1.46E-12 | 4.22E-13 |
| *N*-linoleoyl glycine | WT young | 6 | 3.50E-12 | 1.08E-12 | 4.40E-13 | 6 | 1.99E-12 | 4.59E-13 | 1.87E-13 |
|  | WT old | 6 | 2.48E-12 | 1.75E-13 | 7.12E-14 | 5 | 1.53E-12 | 5.29E-13 | 2.37E-13 |
|  | WT | 12 | 2.99E-12 | 9.10E-13 | 2.63E-13 | 11 | 1.78E-12 | 5.25E-13 | 1.58E-13 |
|  | KO young | 6 | 3.66E-12 | 8.91E-13 | 3.64E-13 | 6 | 1.58E-12 | 5.67E-13 | 2.31E-13 |
|  | KO old | 9 | 2.76E-12 | 3.84E-13 | 1.28E-13 | 7 | 1.28E-12 | 3.20E-13 | 1.21E-13 |
|  | ABHD12 KO | 15 | 3.12E-12 | 7.60E-13 | 1.96E-13 | 13 | 1.42E-12 | 4.57E-13 | 1.27E-13 |
|  | Young | 12 | 3.58E-12 | 9.47E-13 | 2.73E-13 | 12 | 1.78E-12 | 5.37E-13 | 1.55E-13 |
|  | Old | 15 | 2.65E-12 | 3.38E-13 | 8.74E-14 | 12 | 1.39E-12 | 4.16E-13 | 1.20E-13 |
| *N*-arachidonoyl glycine | WT young | 6 | 3.98E-11 | 6.48E-12 | 2.64E-12 | 6 | 2.14E-11 | 3.58E-12 | 1.46E-12 |
|  | WT old | 6 | 3.11E-11 | 2.07E-12 | 8.46E-13 | 5 | 1.66E-11 | 4.21E-12 | 1.88E-12 |
|  | WT | 12 | 3.54E-11 | 6.44E-12 | 1.86E-12 | 11 | 1.92E-11 | 4.46E-12 | 1.35E-12 |
|  | KO young | 6 | 4.14E-11 | 4.76E-12 | 1.94E-12 | 6 | 2.10E-11 | 3.45E-12 | 1.41E-12 |
|  | KO old | 9 | 3.56E-11 | 3.51E-12 | 1.17E-12 | 7 | 1.72E-11 | 3.43E-12 | 1.30E-12 |
|  | ABHD12 KO | 15 | 3.79E-11 | 4.86E-12 | 1.26E-12 | 13 | 1.90E-11 | 3.83E-12 | 1.06E-12 |
|  | Young | 12 | 4.06E-11 | 5.49E-12 | 1.58E-12 | 12 | 2.12E-11 | 3.36E-12 | 9.69E-13 |
|  | Old | 15 | 3.38E-11 | 3.72E-12 | 9.60E-13 | 12 | 1.70E-11 | 3.60E-12 | 1.04E-12 |
| *N*-docosahexaenoyl glycine | WT young | 6 | 1.70E-11 | 3.28E-12 | 1.34E-12 | 6 | 9.41E-12 | 2.36E-12 | 9.62E-13 |
|  | WT old | 6 | 1.36E-11 | 1.21E-12 | 4.92E-13 | 5 | 6.54E-12 | 2.13E-12 | 9.52E-13 |
|  | WT | 12 | 1.53E-11 | 2.94E-12 | 8.48E-13 | 11 | 8.11E-12 | 2.61E-12 | 7.87E-13 |
|  | KO young | 6 | 1.70E-11 | 2.45E-12 | 1.00E-12 | 6 | 7.73E-12 | 2.25E-12 | 9.18E-13 |
|  | KO old | 9 | 1.46E-11 | 1.49E-12 | 4.98E-13 | 7 | 7.52E-12 | 7.35E-13 | 2.78E-13 |
|  | ABHD12 KO | 15 | 1.56E-11 | 2.21E-12 | 5.71E-13 | 13 | 7.62E-12 | 1.55E-12 | 4.29E-13 |
|  | Young | 12 | 1.70E-11 | 2.76E-12 | 7.97E-13 | 12 | 8.57E-12 | 2.36E-12 | 6.82E-13 |
|  | Old | 15 | 1.42E-11 | 1.43E-12 | 3.69E-13 | 12 | 7.11E-12 | 1.48E-12 | 4.28E-13 |

Supplemental Table 17: Mean levels of *N*-acyl glycines in the midbrain and brainstem

| Lipid Species | Group | Midbrain | | | | Brainstem | | | |
| --- | --- | --- | --- | --- | --- | --- | --- | --- | --- |
|  |  | N | Mean | SD | SE | N | Mean | SD | SE |
| *N*-palmitoyl glycine | WT young | 6 | 4.76E-11 | 2.08E-12 | 8.50E-13 | 6 | 5.20E-11 | 3.95E-12 | 1.61E-12 |
|  | WT old | 6 | 4.48E-11 | 3.71E-12 | 1.51E-12 | 6 | 4.96E-11 | 2.89E-12 | 1.18E-12 |
|  | WT | 12 | 4.62E-11 | 3.20E-12 | 9.25E-13 | 12 | 5.08E-11 | 3.53E-12 | 1.02E-12 |
|  | KO young | 6 | 4.79E-11 | 4.26E-12 | 1.74E-12 | 6 | 5.16E-11 | 5.74E-12 | 2.34E-12 |
|  | KO old | 9 | 4.70E-11 | 3.18E-12 | 1.06E-12 | 9 | 4.75E-11 | 3.27E-12 | 1.09E-12 |
|  | ABHD12 KO | 15 | 4.73E-11 | 3.53E-12 | 9.12E-13 | 15 | 4.91E-11 | 4.69E-12 | 1.21E-12 |
|  | Young | 12 | 4.77E-11 | 3.20E-12 | 9.24E-13 | 12 | 5.18E-11 | 4.70E-12 | 1.36E-12 |
|  | Old | 15 | 4.61E-11 | 3.45E-12 | 8.91E-13 | 15 | 4.84E-11 | 3.19E-12 | 8.24E-13 |
| *N*-stearoyl glycine | WT young | 6 | 2.26E-11 | 2.63E-12 | 1.07E-12 | 6 | 3.41E-11 | 4.48E-12 | 1.83E-12 |
|  | WT old | 6 | 1.75E-11 | 1.78E-12 | 7.26E-13 | 6 | 2.93E-11 | 1.62E-12 | 6.63E-13 |
|  | WT | 12 | 2.01E-11 | 3.42E-12 | 9.87E-13 | 12 | 3.17E-11 | 4.08E-12 | 1.18E-12 |
|  | KO young | 6 | 2.32E-11 | 3.23E-12 | 1.32E-12 | 6 | 3.76E-11 | 8.40E-12 | 3.43E-12 |
|  | KO old | 9 | 1.95E-11 | 1.66E-12 | 5.55E-13 | 9 | 2.94E-11 | 2.26E-12 | 7.54E-13 |
|  | ABHD12 KO | 15 | 2.10E-11 | 2.98E-12 | 7.69E-13 | 15 | 3.27E-11 | 6.73E-12 | 1.74E-12 |
|  | Young | 12 | 2.29E-11 | 2.82E-12 | 8.15E-13 | 12 | 3.58E-11 | 6.68E-12 | 1.93E-12 |
|  | Old | 15 | 1.87E-11 | 1.92E-12 | 4.95E-13 | 15 | 2.94E-11 | 1.97E-12 | 5.08E-13 |
| *N*-oleoyl glycine | WT young | 6 | 2.22E-11 | 1.50E-12 | 6.14E-13 | 6 | 2.81E-11 | 2.99E-12 | 1.22E-12 |
|  | WT old | 6 | 1.93E-11 | 1.12E-12 | 4.57E-13 | 6 | 2.53E-11 | 2.52E-12 | 1.03E-12 |
|  | WT | 12 | 2.08E-11 | 1.96E-12 | 5.67E-13 | 12 | 2.67E-11 | 3.01E-12 | 8.68E-13 |
|  | KO young | 6 | 2.19E-11 | 3.18E-12 | 1.30E-12 | 6 | 2.91E-11 | 4.72E-12 | 1.93E-12 |
|  | KO old | 9 | 1.98E-11 | 1.55E-12 | 5.17E-13 | 9 | 2.39E-11 | 2.35E-12 | 7.82E-13 |
|  | ABHD12 KO | 15 | 2.06E-11 | 2.47E-12 | 6.37E-13 | 15 | 2.60E-11 | 4.24E-12 | 1.10E-12 |
|  | Young | 12 | 2.20E-11 | 2.38E-12 | 6.86E-13 | 12 | 2.86E-11 | 3.80E-12 | 1.10E-12 |
|  | Old | 15 | 1.96E-11 | 1.37E-12 | 3.55E-13 | 15 | 2.45E-11 | 2.44E-12 | 6.29E-13 |
| *N*-linoleoyl glycine | WT young | 6 | 3.40E-12 | 5.03E-13 | 2.05E-13 | 6 | 5.15E-12 | 4.34E-13 | 1.77E-13 |
|  | WT old | 6 | 2.64E-12 | 4.88E-13 | 1.99E-13 | 6 | 4.57E-12 | 5.03E-13 | 2.05E-13 |
|  | WT | 12 | 3.02E-12 | 6.16E-13 | 1.78E-13 | 12 | 4.86E-12 | 5.42E-13 | 1.57E-13 |
|  | KO young | 6 | 3.32E-12 | 4.58E-13 | 1.87E-13 | 6 | 5.02E-12 | 7.56E-13 | 3.09E-13 |
|  | KO old | 9 | 2.95E-12 | 3.81E-13 | 1.27E-13 | 9 | 4.22E-12 | 5.05E-13 | 1.68E-13 |
|  | ABHD12 KO | 15 | 3.10E-12 | 4.39E-13 | 1.13E-13 | 15 | 4.54E-12 | 7.18E-13 | 1.85E-13 |
|  | Young | 12 | 3.36E-12 | 4.60E-13 | 1.33E-13 | 12 | 5.09E-12 | 5.92E-13 | 1.71E-13 |
|  | Old | 15 | 2.83E-12 | 4.38E-13 | 1.13E-13 | 15 | 4.36E-12 | 5.18E-13 | 1.34E-13 |
| *N*-arachidonoyl glycine | WT young | 6 | 3.30E-11 | 1.41E-12 | 5.78E-13 | 6 | 3.42E-11 | 1.87E-12 | 7.62E-13 |
|  | WT old | 6 | 3.00E-11 | 1.90E-12 | 7.76E-13 | 6 | 3.23E-11 | 1.24E-12 | 5.08E-13 |
|  | WT | 12 | 3.15E-11 | 2.26E-12 | 6.51E-13 | 12 | 3.32E-11 | 1.82E-12 | 5.26E-13 |
|  | KO young | 6 | 3.75E-11 | 4.63E-12 | 1.89E-12 | 6 | 3.86E-11 | 4.20E-12 | 1.72E-12 |
|  | KO old | 9 | 3.40E-11 | 3.23E-12 | 1.08E-12 | 9 | 3.60E-11 | 1.87E-12 | 6.25E-13 |
|  | ABHD12 KO | 15 | 3.54E-11 | 4.08E-12 | 1.05E-12 | 15 | 3.70E-11 | 3.18E-12 | 8.21E-13 |
|  | Young | 12 | 3.52E-11 | 4.00E-12 | 1.16E-12 | 12 | 3.64E-11 | 3.86E-12 | 1.12E-12 |
|  | Old | 15 | 3.24E-11 | 3.39E-12 | 8.75E-13 | 15 | 3.45E-11 | 2.47E-12 | 6.37E-13 |
| *N*-docosahexaenoyl glycine | WT young | 6 | 1.72E-11 | 7.75E-13 | 3.16E-13 | 6 | 2.12E-11 | 1.82E-12 | 7.42E-13 |
|  | WT old | 6 | 1.47E-11 | 1.96E-12 | 7.99E-13 | 6 | 2.03E-11 | 1.36E-12 | 5.56E-13 |
|  | WT | 12 | 1.59E-11 | 1.94E-12 | 5.59E-13 | 12 | 2.07E-11 | 1.62E-12 | 4.67E-13 |
|  | KO young | 6 | 1.71E-11 | 1.74E-12 | 7.11E-13 | 6 | 2.18E-11 | 2.84E-12 | 1.16E-12 |
|  | KO old | 9 | 1.57E-11 | 1.16E-12 | 3.87E-13 | 9 | 1.96E-11 | 1.47E-12 | 4.90E-13 |
|  | ABHD12 KO | 15 | 1.63E-11 | 1.53E-12 | 3.95E-13 | 15 | 2.05E-11 | 2.31E-12 | 5.98E-13 |
|  | Young | 12 | 1.71E-11 | 1.29E-12 | 3.71E-13 | 12 | 2.15E-11 | 2.29E-12 | 6.61E-13 |
|  | Old | 15 | 1.53E-11 | 1.56E-12 | 4.02E-13 | 15 | 1.99E-11 | 1.42E-12 | 3.66E-13 |

Supplemental Table 18: Mean levels of *N*-acyl leucines in the striatum and hippocampus

| Lipid Species | Group | Striatum | | | | Hippocampus | | | |
| --- | --- | --- | --- | --- | --- | --- | --- | --- | --- |
|  |  | N | Mean | SD | SE | N | Mean | SD | SE |
| *N*-palmitoyl leucine | WT young | 6 | 3.14E-12 | 9.17E-14 | 3.74E-14 | 6 | 2.07E-12 | 3.17E-13 | 1.29E-13 |
|  | WT old | 6 | 3.47E-12 | 1.29E-12 | 5.28E-13 | 6 | 1.84E-12 | 1.44E-13 | 5.88E-14 |
|  | WT | 12 | 3.31E-12 | 8.90E-13 | 2.57E-13 | 12 | 1.96E-12 | 2.64E-13 | 7.63E-14 |
|  | KO young | 6 | 2.91E-12 | 4.65E-13 | 1.90E-13 | 6 | 2.09E-12 | 1.02E-13 | 4.17E-14 |
|  | KO old | 9 | 2.91E-12 | 4.15E-13 | 1.38E-13 | 9 | 1.71E-12 | 2.59E-13 | 8.64E-14 |
|  | ABHD12 KO | 15 | 2.91E-12 | 4.19E-13 | 1.08E-13 | 15 | 1.86E-12 | 2.84E-13 | 7.34E-14 |
|  | Young | 12 | 3.03E-12 | 3.42E-13 | 9.88E-14 | 12 | 2.08E-12 | 2.25E-13 | 6.48E-14 |
|  | Old | 15 | 3.13E-12 | 8.82E-13 | 2.28E-13 | 15 | 1.76E-12 | 2.24E-13 | 5.79E-14 |
| *N*-stearoyl leucine | WT young | 6 | 2.47E-12 | 3.28E-13 | 1.34E-13 | 6 | 1.46E-12 | 2.45E-13 | 9.99E-14 |
|  | WT old | 6 | 2.21E-12 | 4.01E-13 | 1.64E-13 | 6 | 1.34E-12 | 1.60E-13 | 6.52E-14 |
|  | WT | 12 | 2.34E-12 | 3.75E-13 | 1.08E-13 | 12 | 1.40E-12 | 2.06E-13 | 5.95E-14 |
|  | KO young | 6 | 2.72E-12 | 3.38E-13 | 1.38E-13 | 6 | 1.53E-12 | 1.22E-13 | 4.96E-14 |
|  | KO old | 9 | 2.58E-12 | 5.67E-13 | 1.89E-13 | 9 | 1.24E-12 | 1.55E-13 | 5.16E-14 |
|  | ABHD12 KO | 15 | 2.64E-12 | 4.79E-13 | 1.24E-13 | 15 | 1.36E-12 | 2.01E-13 | 5.19E-14 |
|  | Young | 12 | 2.59E-12 | 3.43E-13 | 9.89E-14 | 12 | 1.50E-12 | 1.88E-13 | 5.43E-14 |
|  | Old | 15 | 2.43E-12 | 5.26E-13 | 1.36E-13 | 15 | 1.28E-12 | 1.59E-13 | 4.11E-14 |
| *N*-oleoyl leucine | WT young | 6 | 1.97E-12 | 1.28E-13 | 5.24E-14 | 6 | 1.14E-12 | 1.51E-13 | 6.15E-14 |
|  | WT old | 6 | 1.82E-12 | 4.30E-13 | 1.75E-13 | 6 | 8.99E-13 | 1.92E-13 | 7.86E-14 |
|  | WT | 12 | 1.89E-12 | 3.12E-13 | 9.01E-14 | 12 | 1.02E-12 | 2.09E-13 | 6.02E-14 |
|  | KO young | 6 | 1.81E-12 | 5.13E-13 | 2.09E-13 | 6 | 1.04E-12 | 1.73E-13 | 7.07E-14 |
|  | KO old | 9 | 1.75E-12 | 3.55E-13 | 1.18E-13 | 9 | 9.58E-13 | 2.16E-13 | 7.21E-14 |
|  | ABHD12 KO | 15 | 1.77E-12 | 4.09E-13 | 1.06E-13 | 15 | 9.92E-13 | 1.98E-13 | 5.12E-14 |
|  | Young | 12 | 1.89E-12 | 3.66E-13 | 1.06E-13 | 12 | 1.09E-12 | 1.64E-13 | 4.72E-14 |
|  | Old | 15 | 1.78E-12 | 3.73E-13 | 9.64E-14 | 15 | 9.34E-13 | 2.02E-13 | 5.22E-14 |
| *N*-linoleoyl leucine | WT young | 6 | 4.13E-13 | 1.29E-13 | 5.25E-14 | 6 | 2.67E-13 | 5.45E-14 | 2.23E-14 |
|  | WT old | 6 | 5.72E-13 | 3.43E-13 | 1.40E-13 | 6 | 2.00E-13 | 8.16E-14 | 3.33E-14 |
|  | WT | 12 | 4.92E-13 | 2.61E-13 | 7.53E-14 | 12 | 2.34E-13 | 7.49E-14 | 2.16E-14 |
|  | KO young | 6 | 4.28E-13 | 1.55E-13 | 6.31E-14 | 6 | 3.17E-13 | 4.21E-14 | 1.72E-14 |
|  | KO old | 9 | 3.23E-13 | 1.59E-13 | 5.29E-14 | 9 | 1.80E-13 | 6.68E-14 | 2.23E-14 |
|  | ABHD12 KO | 15 | 3.65E-13 | 1.61E-13 | 4.15E-14 | 15 | 2.35E-13 | 8.95E-14 | 2.31E-14 |
|  | Young | 12 | 4.21E-13 | 1.36E-13 | 3.92E-14 | 12 | 2.92E-13 | 5.32E-14 | 1.54E-14 |
|  | Old | 15 | 4.22E-13 | 2.69E-13 | 6.95E-14 | 15 | 1.88E-13 | 7.09E-14 | 1.83E-14 |
| *N*-docosahexaenoyl leucine | WT young | 6 | 4.08E-13 | 9.81E-14 | 4.01E-14 | 6 | 3.67E-13 | 7.21E-14 | 2.94E-14 |
|  | WT old | 6 | 3.87E-13 | 1.09E-13 | 4.46E-14 | 6 | 3.68E-13 | 4.75E-14 | 1.94E-14 |
|  | WT | 12 | 3.97E-13 | 9.95E-14 | 2.87E-14 | 12 | 3.67E-13 | 5.82E-14 | 1.68E-14 |
|  | KO young | 6 | 3.01E-13 | 7.99E-14 | 3.26E-14 | 6 | 3.03E-13 | 4.79E-14 | 1.96E-14 |
|  | KO old | 9 | 3.26E-13 | 1.05E-13 | 3.49E-14 | 9 | 2.45E-13 | 7.58E-14 | 2.53E-14 |
|  | ABHD12 KO | 15 | 3.16E-13 | 9.33E-14 | 2.41E-14 | 15 | 2.68E-13 | 7.06E-14 | 1.82E-14 |
|  | Young | 12 | 3.54E-13 | 1.02E-13 | 2.94E-14 | 12 | 3.35E-13 | 6.71E-14 | 1.94E-14 |
|  | Old | 15 | 3.50E-13 | 1.07E-13 | 2.77E-14 | 15 | 2.94E-13 | 8.92E-14 | 2.30E-14 |

Supplemental Table 19: Mean levels of *N*-acyl leucines in the cerebellum and thalamus

| Lipid Species | Group | Cerebellum | | | | Thalamus | | | |
| --- | --- | --- | --- | --- | --- | --- | --- | --- | --- |
|  |  | N | Mean | SD | SE | N | Mean | SD | SE |
| *N*-palmitoyl leucine | WT young | 6 | 2.05E-12 | 1.38E-13 | 5.64E-14 | 6 | 1.69E-12 | 2.10E-13 | 8.56E-14 |
|  | WT old | 6 | 2.18E-12 | 3.21E-13 | 1.31E-13 | 6 | 1.61E-12 | 4.74E-14 | 1.94E-14 |
|  | WT | 12 | 2.12E-12 | 2.46E-13 | 7.10E-14 | 12 | 1.65E-12 | 1.51E-13 | 4.36E-14 |
|  | KO young | 6 | 2.06E-12 | 2.72E-13 | 1.11E-13 | 6 | 1.66E-12 | 1.16E-13 | 4.74E-14 |
|  | KO old | 9 | 1.89E-12 | 1.58E-13 | 5.26E-14 | 9 | 1.81E-12 | 7.95E-13 | 2.65E-13 |
|  | ABHD12 KO | 15 | 1.96E-12 | 2.19E-13 | 5.64E-14 | 15 | 1.75E-12 | 6.09E-13 | 1.57E-13 |
|  | Young | 12 | 2.05E-12 | 2.06E-13 | 5.94E-14 | 12 | 1.67E-12 | 1.63E-13 | 4.70E-14 |
|  | Old | 15 | 2.01E-12 | 2.70E-13 | 6.96E-14 | 15 | 1.73E-12 | 6.09E-13 | 1.57E-13 |
| *N*-stearoyl leucine | WT young | 6 | 1.07E-12 | 8.96E-14 | 3.66E-14 | 6 | 8.79E-13 | 1.85E-13 | 7.55E-14 |
|  | WT old | 6 | 1.13E-12 | 1.51E-13 | 6.16E-14 | 6 | 8.22E-13 | 5.75E-14 | 2.35E-14 |
|  | WT | 12 | 1.10E-12 | 1.23E-13 | 3.55E-14 | 12 | 8.51E-13 | 1.34E-13 | 3.87E-14 |
|  | KO young | 6 | 1.22E-12 | 1.80E-13 | 7.33E-14 | 6 | 9.30E-13 | 1.51E-13 | 6.17E-14 |
|  | KO old | 9 | 1.05E-12 | 1.28E-13 | 4.26E-14 | 9 | 8.72E-13 | 2.17E-13 | 7.24E-14 |
|  | ABHD12 KO | 15 | 1.12E-12 | 1.67E-13 | 4.32E-14 | 15 | 8.95E-13 | 1.90E-13 | 4.90E-14 |
|  | Young | 12 | 1.14E-12 | 1.57E-13 | 4.53E-14 | 12 | 9.05E-13 | 1.63E-13 | 4.71E-14 |
|  | Old | 15 | 1.08E-12 | 1.38E-13 | 3.56E-14 | 15 | 8.52E-13 | 1.70E-13 | 4.38E-14 |
| *N*-oleoyl leucine | WT young | 6 | 1.28E-12 | 2.50E-13 | 1.02E-13 | 6 | 7.78E-13 | 8.65E-14 | 3.53E-14 |
|  | WT old | 6 | 1.24E-12 | 2.03E-13 | 8.29E-14 | 6 | 8.01E-13 | 2.59E-13 | 1.06E-13 |
|  | WT | 12 | 1.26E-12 | 2.19E-13 | 6.31E-14 | 12 | 7.89E-13 | 1.85E-13 | 5.33E-14 |
|  | KO young | 6 | 1.14E-12 | 1.74E-13 | 7.10E-14 | 6 | 8.05E-13 | 1.64E-13 | 6.68E-14 |
|  | KO old | 9 | 1.03E-12 | 2.06E-13 | 6.88E-14 | 9 | 7.28E-13 | 1.43E-13 | 4.75E-14 |
|  | ABHD12 KO | 15 | 1.07E-12 | 1.96E-13 | 5.07E-14 | 15 | 7.58E-13 | 1.51E-13 | 3.89E-14 |
|  | Young | 12 | 1.21E-12 | 2.18E-13 | 6.30E-14 | 12 | 7.91E-13 | 1.26E-13 | 3.63E-14 |
|  | Old | 15 | 1.11E-12 | 2.24E-13 | 5.79E-14 | 15 | 7.57E-13 | 1.92E-13 | 4.97E-14 |
| *N*-linoleoyl leucine | WT young | 6 | 4.04E-13 | 1.22E-13 | 4.99E-14 | 6 | 2.00E-13 | 1.19E-13 | 4.84E-14 |
|  | WT old | 6 | 2.13E-13 | 6.97E-14 | 2.85E-14 | 6 | 1.99E-13 | 8.35E-14 | 3.41E-14 |
|  | WT | 12 | 3.08E-13 | 1.38E-13 | 3.98E-14 | 12 | 2.00E-13 | 9.78E-14 | 2.82E-14 |
|  | KO young | 6 | 3.27E-13 | 1.38E-13 | 5.64E-14 | 6 | 1.99E-13 | 9.20E-14 | 3.76E-14 |
|  | KO old | 9 | 2.58E-13 | 6.65E-14 | 2.22E-14 | 9 | 1.58E-13 | 7.61E-14 | 2.54E-14 |
|  | ABHD12 KO | 15 | 2.86E-13 | 1.03E-13 | 2.65E-14 | 15 | 1.74E-13 | 8.22E-14 | 2.12E-14 |
|  | Young | 12 | 3.65E-13 | 1.31E-13 | 3.78E-14 | 12 | 1.99E-13 | 1.01E-13 | 2.92E-14 |
|  | Old | 15 | 2.40E-13 | 6.93E-14 | 1.79E-14 | 15 | 1.74E-13 | 7.90E-14 | 2.04E-14 |
| *N*-docosahexaenoyl leucine | WT young | 6 | 6.39E-13 | 1.38E-13 | 5.64E-14 | 6 | 3.87E-13 | 5.76E-14 | 2.35E-14 |
|  | WT old | 6 | 7.58E-13 | 1.58E-13 | 6.46E-14 | 6 | 3.10E-13 | 6.73E-14 | 2.75E-14 |
|  | WT | 12 | 6.99E-13 | 1.55E-13 | 4.46E-14 | 12 | 3.49E-13 | 7.18E-14 | 2.07E-14 |
|  | KO young | 6 | 5.36E-13 | 9.32E-14 | 3.81E-14 | 6 | 2.51E-13 | 6.11E-14 | 2.49E-14 |
|  | KO old | 9 | 5.47E-13 | 7.69E-14 | 2.56E-14 | 9 | 2.38E-13 | 5.03E-14 | 1.68E-14 |
|  | ABHD12 KO | 15 | 5.43E-13 | 8.07E-14 | 2.08E-14 | 15 | 2.43E-13 | 5.31E-14 | 1.37E-14 |
|  | Young | 12 | 5.88E-13 | 1.25E-13 | 3.60E-14 | 12 | 3.19E-13 | 9.09E-14 | 2.62E-14 |
|  | Old | 15 | 6.32E-13 | 1.54E-13 | 3.98E-14 | 15 | 2.67E-13 | 6.64E-14 | 1.72E-14 |

Supplemental Table 20: Mean levels of *N*-acyl leucines in the cortex and hypothalamus

| Lipid Species | Group | Cortex | | | | Hypothalamus | | | |
| --- | --- | --- | --- | --- | --- | --- | --- | --- | --- |
|  |  | N | Mean | SD | SE | N | Mean | SD | SE |
| *N*-palmitoyl leucine | WT young | 6 | 1.41E-12 | 1.49E-13 | 6.08E-14 | 6 | 2.96E-12 | 3.08E-13 | 1.26E-13 |
|  | WT old | 6 | 1.40E-12 | 1.25E-13 | 5.12E-14 | 5 | 2.40E-12 | 5.68E-13 | 2.54E-13 |
|  | WT | 12 | 1.41E-12 | 1.31E-13 | 3.79E-14 | 11 | 2.71E-12 | 5.14E-13 | 1.55E-13 |
|  | KO young | 6 | 1.40E-12 | 1.86E-13 | 7.61E-14 | 6 | 2.68E-12 | 3.51E-13 | 1.43E-13 |
|  | KO old | 9 | 1.45E-12 | 1.35E-13 | 4.51E-14 | 7 | 2.30E-12 | 5.14E-13 | 1.94E-13 |
|  | ABHD12 KO | 15 | 1.43E-12 | 1.53E-13 | 3.96E-14 | 13 | 2.48E-12 | 4.72E-13 | 1.31E-13 |
|  | Young | 12 | 1.41E-12 | 1.61E-13 | 4.65E-14 | 12 | 2.82E-12 | 3.47E-13 | 1.00E-13 |
|  | Old | 15 | 1.43E-12 | 1.29E-13 | 3.34E-14 | 12 | 2.34E-12 | 5.14E-13 | 1.48E-13 |
| *N*-stearoyl leucine | WT young | 6 | 1.43E-12 | 1.20E-13 | 4.90E-14 | 6 | 1.07E-12 | 1.49E-13 | 6.09E-14 |
|  | WT old | 6 | 1.37E-12 | 5.77E-14 | 2.36E-14 | 5 | 9.76E-13 | 1.83E-13 | 8.20E-14 |
|  | WT | 12 | 1.40E-12 | 9.43E-14 | 2.72E-14 | 11 | 1.03E-12 | 1.65E-13 | 4.97E-14 |
|  | KO young | 6 | 1.54E-12 | 1.59E-13 | 6.48E-14 | 6 | 1.09E-12 | 2.68E-13 | 1.10E-13 |
|  | KO old | 9 | 1.49E-12 | 1.04E-13 | 3.47E-14 | 7 | 8.91E-13 | 1.66E-13 | 6.29E-14 |
|  | ABHD12 KO | 15 | 1.51E-12 | 1.27E-13 | 3.27E-14 | 13 | 9.83E-13 | 2.34E-13 | 6.48E-14 |
|  | Young | 12 | 1.49E-12 | 1.47E-13 | 4.24E-14 | 12 | 1.08E-12 | 2.07E-13 | 5.98E-14 |
|  | Old | 15 | 1.44E-12 | 1.03E-13 | 2.67E-14 | 12 | 9.26E-13 | 1.71E-13 | 4.94E-14 |
| *N*-oleoyl leucine | WT young | 6 | 8.12E-13 | 9.74E-14 | 3.98E-14 | 6 | 1.05E-12 | 2.53E-13 | 1.03E-13 |
|  | WT old | 6 | 8.01E-13 | 1.08E-13 | 4.40E-14 | 5 | 9.60E-13 | 2.73E-13 | 1.22E-13 |
|  | WT | 12 | 8.07E-13 | 9.81E-14 | 2.83E-14 | 11 | 1.01E-12 | 2.54E-13 | 7.65E-14 |
|  | KO young | 6 | 8.54E-13 | 1.21E-13 | 4.94E-14 | 6 | 1.03E-12 | 3.24E-13 | 1.32E-13 |
|  | KO old | 9 | 8.23E-13 | 1.06E-13 | 3.53E-14 | 7 | 7.99E-13 | 1.66E-13 | 6.26E-14 |
|  | ABHD12 KO | 15 | 8.35E-13 | 1.09E-13 | 2.81E-14 | 13 | 9.06E-13 | 2.68E-13 | 7.43E-14 |
|  | Young | 12 | 8.33E-13 | 1.07E-13 | 3.09E-14 | 12 | 1.04E-12 | 2.77E-13 | 8.01E-14 |
|  | Old | 15 | 8.14E-13 | 1.03E-13 | 2.67E-14 | 12 | 8.66E-13 | 2.21E-13 | 6.39E-14 |
| *N*-linoleoyl leucine | WT young | 6 | 1.90E-13 | 4.52E-14 | 1.84E-14 | 6 | PISSR |  |  |
|  | WT old | 6 | 1.87E-13 | 3.99E-14 | 1.63E-14 | 5 | PISSR |  |  |
|  | WT | 12 | 1.88E-13 | 4.06E-14 | 1.17E-14 | 11 | PISSR |  |  |
|  | KO young | 6 | 1.83E-13 | 5.14E-14 | 2.10E-14 | 6 | PISSR |  |  |
|  | KO old | 9 | 1.54E-13 | 3.70E-14 | 1.23E-14 | 7 | PISSR |  |  |
|  | ABHD12 KO | 15 | 1.66E-13 | 4.40E-14 | 1.14E-14 | 13 | PISSR |  |  |
|  | Young | 12 | 1.86E-13 | 4.62E-14 | 1.34E-14 | 12 | PISSR |  |  |
|  | Old | 15 | 1.67E-13 | 4.03E-14 | 1.04E-14 | 12 | PISSR |  |  |
| *N*-docosahexaenoyl leucine | WT young | 6 | 5.65E-13 | 9.33E-14 | 3.81E-14 | 6 | 2.03E-13 | 5.25E-14 | 2.15E-14 |
|  | WT old | 6 | 5.49E-13 | 1.10E-13 | 4.49E-14 | 5 | 1.67E-13 | 4.99E-14 | 2.23E-14 |
|  | WT | 12 | 5.57E-13 | 9.76E-14 | 2.82E-14 | 11 | 1.86E-13 | 5.22E-14 | 1.57E-14 |
|  | KO young | 6 | 4.20E-13 | 5.23E-14 | 2.14E-14 | 6 | 3.22E-13 | 5.91E-14 | 2.41E-14 |
|  | KO old | 9 | 4.51E-13 | 6.07E-14 | 2.02E-14 | 7 | 2.26E-13 | 7.49E-14 | 2.83E-14 |
|  | ABHD12 KO | 15 | 4.38E-13 | 5.78E-14 | 1.49E-14 | 13 | 2.70E-13 | 8.23E-14 | 2.28E-14 |
|  | Young | 12 | 4.92E-13 | 1.05E-13 | 3.02E-14 | 12 | 2.62E-13 | 8.22E-14 | 2.37E-14 |
|  | Old | 15 | 4.90E-13 | 9.43E-14 | 2.43E-14 | 12 | 2.01E-13 | 7.00E-14 | 2.02E-14 |

Supplemental Table 21: Mean levels of *N*-acyl leucines in the midbrain and brainstem

| Lipid Species | Group | Midbrain | | | | Brainstem | | | |
| --- | --- | --- | --- | --- | --- | --- | --- | --- | --- |
|  |  | N | Mean | SD | SE | N | Mean | SD | SE |
| *N*-palmitoyl leucine | WT young | 6 | 1.59E-12 | 2.41E-13 | 9.85E-14 | 6 | 1.49E-12 | 1.48E-13 | 6.03E-14 |
|  | WT old | 6 | 1.49E-12 | 2.07E-13 | 8.45E-14 | 6 | 1.38E-12 | 8.69E-14 | 3.55E-14 |
|  | WT | 12 | 1.54E-12 | 2.21E-13 | 6.37E-14 | 12 | 1.43E-12 | 1.28E-13 | 3.69E-14 |
|  | KO young | 6 | 1.62E-12 | 1.68E-13 | 6.84E-14 | 6 | 1.36E-12 | 1.10E-13 | 4.47E-14 |
|  | KO old | 9 | 1.52E-12 | 1.89E-13 | 6.30E-14 | 9 | 1.37E-12 | 1.28E-13 | 4.27E-14 |
|  | ABHD12 KO | 15 | 1.56E-12 | 1.82E-13 | 4.71E-14 | 15 | 1.37E-12 | 1.17E-13 | 3.02E-14 |
|  | Young | 12 | 1.61E-12 | 1.99E-13 | 5.74E-14 | 12 | 1.42E-12 | 1.41E-13 | 4.06E-14 |
|  | Old | 15 | 1.51E-12 | 1.90E-13 | 4.90E-14 | 15 | 1.38E-12 | 1.10E-13 | 2.84E-14 |
| *N*-stearoyl leucine | WT young | 6 | 8.66E-13 | 9.97E-14 | 4.07E-14 | 6 | 8.62E-13 | 1.68E-13 | 6.87E-14 |
|  | WT old | 6 | 7.47E-13 | 7.62E-14 | 3.11E-14 | 6 | 7.07E-13 | 9.40E-14 | 3.84E-14 |
|  | WT | 12 | 8.07E-13 | 1.05E-13 | 3.03E-14 | 12 | 7.84E-13 | 1.53E-13 | 4.42E-14 |
|  | KO young | 6 | 8.05E-13 | 1.22E-13 | 4.96E-14 | 6 | 8.44E-13 | 1.83E-13 | 7.46E-14 |
|  | KO old | 9 | 7.85E-13 | 9.81E-14 | 3.27E-14 | 9 | 7.41E-13 | 1.32E-13 | 4.41E-14 |
|  | ABHD12 KO | 15 | 7.93E-13 | 1.04E-13 | 2.69E-14 | 15 | 7.82E-13 | 1.57E-13 | 4.06E-14 |
|  | Young | 12 | 8.35E-13 | 1.11E-13 | 3.20E-14 | 12 | 8.53E-13 | 1.68E-13 | 4.84E-14 |
|  | Old | 15 | 7.70E-13 | 8.91E-14 | 2.30E-14 | 15 | 7.27E-13 | 1.16E-13 | 3.00E-14 |
| *N*-oleoyl leucine | WT young | 6 | 8.64E-13 | 1.79E-13 | 7.30E-14 | 6 | 9.81E-13 | 1.52E-13 | 6.19E-14 |
|  | WT old | 6 | 9.32E-13 | 1.51E-13 | 6.17E-14 | 6 | 8.86E-13 | 1.76E-13 | 7.18E-14 |
|  | WT | 12 | 8.98E-13 | 1.62E-13 | 4.67E-14 | 12 | 9.33E-13 | 1.64E-13 | 4.74E-14 |
|  | KO young | 6 | 9.57E-13 | 9.01E-14 | 3.68E-14 | 6 | 1.03E-12 | 2.38E-13 | 9.71E-14 |
|  | KO old | 9 | 8.93E-13 | 1.66E-13 | 5.53E-14 | 9 | 8.62E-13 | 1.38E-13 | 4.60E-14 |
|  | ABHD12 KO | 15 | 9.18E-13 | 1.40E-13 | 3.63E-14 | 15 | 9.29E-13 | 1.96E-13 | 5.05E-14 |
|  | Young | 12 | 9.11E-13 | 1.43E-13 | 4.14E-14 | 12 | 1.01E-12 | 1.92E-13 | 5.54E-14 |
|  | Old | 15 | 9.08E-13 | 1.56E-13 | 4.03E-14 | 15 | 8.72E-13 | 1.49E-13 | 3.84E-14 |
| *N*-linoleoyl leucine | WT young | 6 | 2.05E-13 | 4.15E-14 | 1.69E-14 | 6 | 1.93E-13 | 9.22E-14 | 3.76E-14 |
|  | WT old | 6 | 1.78E-13 | 3.61E-14 | 1.47E-14 | 6 | 1.86E-13 | 3.75E-14 | 1.53E-14 |
|  | WT | 12 | 1.91E-13 | 3.96E-14 | 1.14E-14 | 12 | 1.90E-13 | 6.72E-14 | 1.94E-14 |
|  | KO young | 6 | 2.38E-13 | 5.49E-14 | 2.24E-14 | 6 | 2.11E-13 | 8.78E-14 | 3.58E-14 |
|  | KO old | 9 | 1.43E-13 | 4.98E-14 | 1.66E-14 | 9 | 1.67E-13 | 6.44E-14 | 2.15E-14 |
|  | ABHD12 KO | 15 | 1.81E-13 | 6.93E-14 | 1.79E-14 | 15 | 1.85E-13 | 7.50E-14 | 1.94E-14 |
|  | Young | 12 | 2.21E-13 | 4.96E-14 | 1.43E-14 | 12 | 2.02E-13 | 8.64E-14 | 2.49E-14 |
|  | Old | 15 | 1.57E-13 | 4.68E-14 | 1.21E-14 | 15 | 1.75E-13 | 5.45E-14 | 1.41E-14 |
| *N*-docosahexaenoyl leucine | WT young | 6 | 2.47E-13 | 3.57E-14 | 1.46E-14 | 6 | 2.64E-13 | 3.69E-14 | 1.51E-14 |
|  | WT old | 6 | 2.48E-13 | 2.63E-14 | 1.07E-14 | 6 | 2.84E-13 | 5.35E-14 | 2.19E-14 |
|  | WT | 12 | 2.48E-13 | 2.99E-14 | 8.64E-15 | 12 | 2.74E-13 | 4.51E-14 | 1.30E-14 |
|  | KO young | 6 | 2.17E-13 | 4.08E-14 | 1.67E-14 | 6 | 2.24E-13 | 3.77E-14 | 1.54E-14 |
|  | KO old | 9 | 1.64E-13 | 4.54E-14 | 1.51E-14 | 9 | 2.63E-13 | 6.99E-14 | 2.33E-14 |
|  | ABHD12 KO | 15 | 1.85E-13 | 5.02E-14 | 1.30E-14 | 15 | 2.48E-13 | 6.07E-14 | 1.57E-14 |
|  | Young | 12 | 2.32E-13 | 3.98E-14 | 1.15E-14 | 12 | 2.44E-13 | 4.11E-14 | 1.19E-14 |
|  | Old | 15 | 1.97E-13 | 5.70E-14 | 1.47E-14 | 15 | 2.71E-13 | 6.27E-14 | 1.62E-14 |

Supplemental Table 22: Mean levels of *N*-acyl methionines in the striatum and hippocampus

| Lipid Species | Group | Striatum | | | | Hippocampus | | | |
| --- | --- | --- | --- | --- | --- | --- | --- | --- | --- |
|  |  | N | Mean | SD | SE | N | Mean | SD | SE |
| *N*-palmitoyl methionine | WT young | 6 | 4.58E-12 | 5.03E-13 | 2.05E-13 | 6 | 2.29E-12 | 4.88E-13 | 1.99E-13 |
|  | WT old | 6 | 4.69E-12 | 2.04E-12 | 8.31E-13 | 6 | 1.99E-12 | 5.81E-13 | 2.37E-13 |
|  | WT | 12 | 4.63E-12 | 1.41E-12 | 4.08E-13 | 12 | 2.14E-12 | 5.34E-13 | 1.54E-13 |
|  | KO young | 6 | 4.84E-12 | 1.43E-12 | 5.85E-13 | 6 | 2.07E-12 | 5.07E-13 | 2.07E-13 |
|  | KO old | 9 | 4.40E-12 | 1.01E-12 | 3.37E-13 | 9 | 1.99E-12 | 7.08E-13 | 2.36E-13 |
|  | ABHD12 KO | 15 | 4.57E-12 | 1.17E-12 | 3.02E-13 | 15 | 2.02E-12 | 6.17E-13 | 1.59E-13 |
|  | Young | 12 | 4.71E-12 | 1.03E-12 | 2.98E-13 | 12 | 2.18E-12 | 4.88E-13 | 1.41E-13 |
|  | Old | 15 | 4.51E-12 | 1.44E-12 | 3.73E-13 | 15 | 1.99E-12 | 6.38E-13 | 1.65E-13 |
| *N*-stearoyl methionine | WT young | 6 | 3.48E-12 | 9.23E-13 | 3.77E-13 | 6 | 1.98E-12 | 4.39E-13 | 1.79E-13 |
|  | WT old | 6 | 3.35E-12 | 9.42E-13 | 3.85E-13 | 6 | 1.75E-12 | 3.78E-13 | 1.54E-13 |
|  | WT | 12 | 3.42E-12 | 8.92E-13 | 2.57E-13 | 12 | 1.87E-12 | 4.08E-13 | 1.18E-13 |
|  | KO young | 6 | 5.30E-12 | 1.30E-12 | 5.31E-13 | 6 | 2.52E-12 | 4.82E-13 | 1.97E-13 |
|  | KO old | 9 | 4.21E-12 | 6.39E-13 | 2.13E-13 | 9 | 1.91E-12 | 3.73E-13 | 1.24E-13 |
|  | ABHD12 KO | 15 | 4.65E-12 | 1.07E-12 | 2.76E-13 | 15 | 2.15E-12 | 5.06E-13 | 1.31E-13 |
|  | Young | 12 | 4.39E-12 | 1.43E-12 | 4.14E-13 | 12 | 2.25E-12 | 5.22E-13 | 1.51E-13 |
|  | Old | 15 | 3.87E-12 | 8.60E-13 | 2.22E-13 | 15 | 1.85E-12 | 3.71E-13 | 9.57E-14 |
| *N*-oleoyl methionine | WT young | 6 | 3.29E-12 | 1.13E-12 | 4.62E-13 | 6 | 1.03E-12 | 4.93E-13 | 2.01E-13 |
|  | WT old | 6 | 3.55E-12 | 7.35E-13 | 3.00E-13 | 6 | 1.12E-12 | 3.32E-13 | 1.35E-13 |
|  | WT | 12 | 3.42E-12 | 9.19E-13 | 2.65E-13 | 12 | 1.08E-12 | 4.03E-13 | 1.16E-13 |
|  | KO young | 6 | 3.22E-12 | 7.75E-13 | 3.16E-13 | 6 | 1.00E-12 | 3.09E-13 | 1.26E-13 |
|  | KO old | 9 | 2.68E-12 | 4.22E-13 | 1.41E-13 | 9 | 1.08E-12 | 3.01E-13 | 1.00E-13 |
|  | ABHD12 KO | 15 | 2.89E-12 | 6.26E-13 | 1.62E-13 | 15 | 1.05E-12 | 2.96E-13 | 7.64E-14 |
|  | Young | 12 | 3.25E-12 | 9.25E-13 | 2.67E-13 | 12 | 1.02E-12 | 3.93E-13 | 1.13E-13 |
|  | Old | 15 | 3.02E-12 | 7.01E-13 | 1.81E-13 | 15 | 1.10E-12 | 3.03E-13 | 7.81E-14 |
| *N*-linoleoyl methionine | WT young | 6 | BDL |  |  | 6 | BDL |  |  |
|  | WT old | 6 | BDL |  |  | 6 | BDL |  |  |
|  | WT | 12 | BDL |  |  | 12 | BDL |  |  |
|  | KO young | 6 | BDL |  |  | 6 | BDL |  |  |
|  | KO old | 9 | BDL |  |  | 9 | BDL |  |  |
|  | ABHD12 KO | 15 | BDL |  |  | 15 | BDL |  |  |
|  | Young | 12 | BDL |  |  | 12 | BDL |  |  |
|  | Old | 15 | BDL |  |  | 15 | BDL |  |  |
| *N*-arachidonoyl methionine | WT young | 6 | PISSR |  |  | 6 | PISSR |  |  |
|  | WT old | 6 | PISSR |  |  | 6 | PISSR |  |  |
|  | WT | 12 | PISSR |  |  | 12 | PISSR |  |  |
|  | KO young | 6 | PISSR |  |  | 6 | PISSR |  |  |
|  | KO old | 9 | PISSR |  |  | 9 | PISSR |  |  |
|  | ABHD12 KO | 15 | PISSR |  |  | 15 | PISSR |  |  |
|  | Young | 12 | PISSR |  |  | 12 | PISSR |  |  |
|  | Old | 15 | PISSR |  |  | 15 | PISSR |  |  |
| *N*-docosahexaenoyl methionine | WT young | 6 | PISSR |  |  | 6 | PISSR |  |  |
|  | WT old | 6 | PISSR |  |  | 6 | PISSR |  |  |
|  | WT | 12 | PISSR |  |  | 12 | PISSR |  |  |
|  | KO young | 6 | PISSR |  |  | 6 | PISSR |  |  |
|  | KO old | 9 | PISSR |  |  | 9 | PISSR |  |  |
|  | ABHD12 KO | 15 | PISSR |  |  | 15 | PISSR |  |  |
|  | Young | 12 | PISSR |  |  | 12 | PISSR |  |  |
|  | Old | 15 | PISSR |  |  | 15 | PISSR |  |  |

Supplemental Table 23: Mean levels of *N*-acyl methionines in the cerebellum and thalamus

| Lipid Species | Group | Cerebellum | | | | Thalamus | | | |
| --- | --- | --- | --- | --- | --- | --- | --- | --- | --- |
|  |  | N | Mean | SD | SE | N | Mean | SD | SE |
| *N*-palmitoyl methionine | WT young | 6 | 7.71E-12 | 1.60E-12 | 6.51E-13 | 6 | 4.45E-12 | 4.85E-13 | 1.98E-13 |
|  | WT old | 6 | 7.77E-12 | 3.76E-12 | 1.54E-12 | 6 | 4.74E-12 | 7.15E-13 | 2.92E-13 |
|  | WT | 12 | 7.74E-12 | 2.76E-12 | 7.96E-13 | 12 | 4.59E-12 | 6.02E-13 | 1.74E-13 |
|  | KO young | 6 | 9.56E-12 | 2.30E-12 | 9.37E-13 | 6 | 4.76E-12 | 9.49E-13 | 3.87E-13 |
|  | KO old | 9 | 7.98E-12 | 2.83E-12 | 9.43E-13 | 9 | 4.97E-12 | 9.53E-13 | 3.18E-13 |
|  | ABHD12 KO | 15 | 8.61E-12 | 2.67E-12 | 6.88E-13 | 15 | 4.89E-12 | 9.24E-13 | 2.38E-13 |
|  | Young | 12 | 8.64E-12 | 2.12E-12 | 6.11E-13 | 12 | 4.61E-12 | 7.36E-13 | 2.13E-13 |
|  | Old | 15 | 7.89E-12 | 3.11E-12 | 8.02E-13 | 15 | 4.88E-12 | 8.46E-13 | 2.19E-13 |
| *N*-stearoyl methionine | WT young | 6 | 1.83E-12 | 3.99E-13 | 1.63E-13 | 6 | 2.28E-12 | 1.66E-13 | 6.76E-14 |
|  | WT old | 6 | 2.16E-12 | 5.10E-13 | 2.08E-13 | 6 | 1.87E-12 | 4.39E-13 | 1.79E-13 |
|  | WT | 12 | 2.00E-12 | 4.70E-13 | 1.36E-13 | 12 | 2.07E-12 | 3.82E-13 | 1.10E-13 |
|  | KO young | 6 | 2.69E-12 | 6.62E-13 | 2.70E-13 | 6 | 2.84E-12 | 8.64E-13 | 3.53E-13 |
|  | KO old | 9 | 2.16E-12 | 7.71E-13 | 2.57E-13 | 9 | 2.20E-12 | 4.10E-13 | 1.37E-13 |
|  | ABHD12 KO | 15 | 2.37E-12 | 7.56E-13 | 1.95E-13 | 15 | 2.46E-12 | 6.86E-13 | 1.77E-13 |
|  | Young | 12 | 2.26E-12 | 6.90E-13 | 1.99E-13 | 12 | 2.56E-12 | 6.63E-13 | 1.91E-13 |
|  | Old | 15 | 2.16E-12 | 6.57E-13 | 1.70E-13 | 15 | 2.07E-12 | 4.39E-13 | 1.13E-13 |
| *N*-oleoyl methionine | WT young | 6 | 1.21E-12 | 2.65E-13 | 1.08E-13 | 6 | 9.83E-13 | 1.82E-13 | 7.41E-14 |
|  | WT old | 6 | 1.18E-12 | 2.93E-13 | 1.19E-13 | 6 | 9.93E-13 | 4.07E-13 | 1.66E-13 |
|  | WT | 12 | 1.19E-12 | 2.67E-13 | 7.70E-14 | 12 | 9.88E-13 | 3.01E-13 | 8.67E-14 |
|  | KO young | 6 | 1.29E-12 | 3.44E-13 | 1.40E-13 | 6 | 1.28E-12 | 4.67E-13 | 1.91E-13 |
|  | KO old | 9 | 1.16E-12 | 5.24E-13 | 1.75E-13 | 9 | 1.12E-12 | 2.33E-13 | 7.77E-14 |
|  | ABHD12 KO | 15 | 1.21E-12 | 4.50E-13 | 1.16E-13 | 15 | 1.18E-12 | 3.39E-13 | 8.76E-14 |
|  | Young | 12 | 1.25E-12 | 2.95E-13 | 8.52E-14 | 12 | 1.13E-12 | 3.71E-13 | 1.07E-13 |
|  | Old | 15 | 1.17E-12 | 4.33E-13 | 1.12E-13 | 15 | 1.07E-12 | 3.07E-13 | 7.93E-14 |
| *N*-linoleoyl methionine | WT young | 6 | BDL |  |  | 6 | BDL |  |  |
|  | WT old | 6 | BDL |  |  | 6 | BDL |  |  |
|  | WT | 12 | BDL |  |  | 12 | BDL |  |  |
|  | KO young | 6 | BDL |  |  | 6 | BDL |  |  |
|  | KO old | 9 | BDL |  |  | 9 | BDL |  |  |
|  | ABHD12 KO | 15 | BDL |  |  | 15 | BDL |  |  |
|  | Young | 12 | BDL |  |  | 12 | BDL |  |  |
|  | Old | 15 | BDL |  |  | 15 | BDL |  |  |
| *N*-arachidonoyl methionine | WT young | 6 | PISSR |  |  | 6 | PISSR |  |  |
|  | WT old | 6 | PISSR |  |  | 6 | PISSR |  |  |
|  | WT | 12 | PISSR |  |  | 12 | PISSR |  |  |
|  | KO young | 6 | PISSR |  |  | 6 | PISSR |  |  |
|  | KO old | 9 | PISSR |  |  | 9 | PISSR |  |  |
|  | ABHD12 KO | 15 | PISSR |  |  | 15 | PISSR |  |  |
|  | Young | 12 | PISSR |  |  | 12 | PISSR |  |  |
|  | Old | 15 | PISSR |  |  | 15 | PISSR |  |  |
| *N*-docosahexaenoyl methionine | WT young | 6 | PISSR |  |  | 6 | PISSR |  |  |
|  | WT old | 6 | PISSR |  |  | 6 | PISSR |  |  |
|  | WT | 12 | PISSR |  |  | 12 | PISSR |  |  |
|  | KO young | 6 | PISSR |  |  | 6 | PISSR |  |  |
|  | KO old | 9 | PISSR |  |  | 9 | PISSR |  |  |
|  | ABHD12 KO | 15 | PISSR |  |  | 15 | PISSR |  |  |
|  | Young | 12 | PISSR |  |  | 12 | PISSR |  |  |
|  | Old | 15 | PISSR |  |  | 15 | PISSR |  |  |

Supplemental Table 24: Mean levels of *N*-acyl methionines in the cortex and hypothalamus

| Lipid Species | Group | Cortex | | | | Hypothalamus | | | |
| --- | --- | --- | --- | --- | --- | --- | --- | --- | --- |
|  |  | N | Mean | SD | SE | N | Mean | SD | SE |
| *N*-palmitoyl methionine | WT young | 6 | 2.98E-12 | 1.05E-12 | 4.28E-13 | 6 | 2.41E-12 | 5.97E-13 | 2.44E-13 |
|  | WT old | 6 | 3.30E-12 | 1.25E-12 | 5.11E-13 | 5 | 2.14E-12 | 5.76E-13 | 2.58E-13 |
|  | WT | 12 | 3.14E-12 | 1.11E-12 | 3.22E-13 | 11 | 2.29E-12 | 5.74E-13 | 1.73E-13 |
|  | KO young | 6 | 3.76E-12 | 1.39E-12 | 5.69E-13 | 6 | 2.08E-12 | 7.11E-13 | 2.90E-13 |
|  | KO old | 9 | 3.81E-12 | 1.17E-12 | 3.88E-13 | 7 | 2.06E-12 | 1.10E-12 | 4.14E-13 |
|  | ABHD12 KO | 15 | 3.79E-12 | 1.21E-12 | 3.13E-13 | 13 | 2.07E-12 | 9.01E-13 | 2.50E-13 |
|  | Young | 12 | 3.37E-12 | 1.24E-12 | 3.59E-13 | 12 | 2.24E-12 | 6.49E-13 | 1.87E-13 |
|  | Old | 15 | 3.61E-12 | 1.18E-12 | 3.06E-13 | 12 | 2.09E-12 | 8.82E-13 | 2.55E-13 |
| *N*-stearoyl methionine | WT young | 6 | 1.91E-12 | 3.26E-13 | 1.33E-13 | 6 | 1.15E-12 | 5.38E-13 | 2.20E-13 |
|  | WT old | 6 | 1.85E-12 | 3.38E-13 | 1.38E-13 | 5 | 1.30E-12 | 4.25E-13 | 1.90E-13 |
|  | WT | 12 | 1.88E-12 | 3.18E-13 | 9.19E-14 | 11 | 1.22E-12 | 4.72E-13 | 1.42E-13 |
|  | KO young | 6 | 2.14E-12 | 3.60E-13 | 1.47E-13 | 6 | 1.25E-12 | 3.12E-13 | 1.27E-13 |
|  | KO old | 9 | 2.07E-12 | 2.65E-13 | 8.84E-14 | 7 | 9.66E-13 | 3.00E-13 | 1.13E-13 |
|  | ABHD12 KO | 15 | 2.10E-12 | 2.96E-13 | 7.65E-14 | 13 | 1.10E-12 | 3.27E-13 | 9.08E-14 |
|  | Young | 12 | 2.02E-12 | 3.49E-13 | 1.01E-13 | 12 | 1.20E-12 | 4.23E-13 | 1.22E-13 |
|  | Old | 15 | 1.98E-12 | 3.07E-13 | 7.92E-14 | 12 | 1.10E-12 | 3.79E-13 | 1.09E-13 |
| *N*-oleoyl methionine | WT young | 6 | 5.89E-13 | 2.12E-13 | 8.65E-14 | 6 | PISSR |  |  |
|  | WT old | 6 | 6.11E-13 | 2.33E-13 | 9.51E-14 | 5 | PISSR |  |  |
|  | WT | 12 | 6.00E-13 | 2.13E-13 | 6.14E-14 | 11 | PISSR |  |  |
|  | KO young | 6 | 7.14E-13 | 2.35E-13 | 9.59E-14 | 6 | PISSR |  |  |
|  | KO old | 9 | 5.88E-13 | 1.69E-13 | 5.64E-14 | 7 | PISSR |  |  |
|  | ABHD12 KO | 15 | 6.38E-13 | 2.00E-13 | 5.17E-14 | 13 | PISSR |  |  |
|  | Young | 12 | 6.51E-13 | 2.23E-13 | 6.44E-14 | 12 | PISSR |  |  |
|  | Old | 15 | 5.97E-13 | 1.89E-13 | 4.89E-14 | 12 | PISSR |  |  |
| *N*-linoleoyl methionine | WT young | 6 | BDL |  |  | 6 | BDL |  |  |
|  | WT old | 6 | BDL |  |  | 5 | BDL |  |  |
|  | WT | 12 | BDL |  |  | 11 | BDL |  |  |
|  | KO young | 6 | BDL |  |  | 6 | BDL |  |  |
|  | KO old | 9 | BDL |  |  | 7 | BDL |  |  |
|  | ABHD12 KO | 15 | BDL |  |  | 13 | BDL |  |  |
|  | Young | 12 | BDL |  |  | 12 | BDL |  |  |
|  | Old | 15 | BDL |  |  | 12 | BDL |  |  |
| *N*-arachidonoyl methionine | WT young | 6 | BDL |  |  | 6 | BDL |  |  |
|  | WT old | 6 | BDL |  |  | 5 | BDL |  |  |
|  | WT | 12 | BDL |  |  | 11 | BDL |  |  |
|  | KO young | 6 | BDL |  |  | 6 | BDL |  |  |
|  | KO old | 9 | BDL |  |  | 7 | BDL |  |  |
|  | ABHD12 KO | 15 | BDL |  |  | 13 | BDL |  |  |
|  | Young | 12 | BDL |  |  | 12 | BDL |  |  |
|  | Old | 15 | BDL |  |  | 12 | BDL |  |  |
| *N*-docosahexaenoyl methionine | WT young | 6 | BDL |  |  | 6 | BDL |  |  |
|  | WT old | 6 | BDL |  |  | 5 | BDL |  |  |
|  | WT | 12 | BDL |  |  | 11 | BDL |  |  |
|  | KO young | 6 | BDL |  |  | 6 | BDL |  |  |
|  | KO old | 9 | BDL |  |  | 7 | BDL |  |  |
|  | ABHD12 KO | 15 | BDL |  |  | 13 | BDL |  |  |
|  | Young | 12 | BDL |  |  | 12 | BDL |  |  |
|  | Old | 15 | BDL |  |  | 12 | BDL |  |  |

Supplemental Table 25: Mean levels of *N*-acyl methionines in the midbrain and brainstem

| Lipid Species | Group | Midbrain | | | | Brainstem | | | |
| --- | --- | --- | --- | --- | --- | --- | --- | --- | --- |
|  |  | N | Mean | SD | SE | N | Mean | SD | SE |
| *N*-palmitoyl methionine | WT young | 6 | 1.80E-12 | 3.57E-13 | 1.46E-13 | 6 | 5.71E-12 | 2.01E-12 | 8.22E-13 |
|  | WT old | 6 | 1.78E-12 | 4.28E-13 | 1.75E-13 | 6 | 4.29E-12 | 6.18E-13 | 2.52E-13 |
|  | WT | 12 | 1.79E-12 | 3.76E-13 | 1.09E-13 | 12 | 5.00E-12 | 1.60E-12 | 4.62E-13 |
|  | KO young | 6 | 1.96E-12 | 5.12E-13 | 2.09E-13 | 6 | 5.04E-12 | 1.03E-12 | 4.22E-13 |
|  | KO old | 9 | 1.77E-12 | 4.55E-13 | 1.52E-13 | 9 | 5.00E-12 | 1.75E-12 | 5.83E-13 |
|  | ABHD12 KO | 15 | 1.85E-12 | 4.71E-13 | 1.22E-13 | 15 | 5.02E-12 | 1.46E-12 | 3.77E-13 |
|  | Young | 12 | 1.88E-12 | 4.29E-13 | 1.24E-13 | 12 | 5.38E-12 | 1.57E-12 | 4.52E-13 |
|  | Old | 15 | 1.77E-12 | 4.29E-13 | 1.11E-13 | 15 | 4.72E-12 | 1.42E-12 | 3.67E-13 |
| *N*-stearoyl methionine | WT young | 6 | 1.81E-12 | 4.24E-13 | 1.73E-13 | 6 | 2.02E-12 | 2.23E-13 | 9.09E-14 |
|  | WT old | 6 | 1.75E-12 | 3.41E-13 | 1.39E-13 | 6 | 1.84E-12 | 2.11E-13 | 8.60E-14 |
|  | WT | 12 | 1.78E-12 | 3.68E-13 | 1.06E-13 | 12 | 1.93E-12 | 2.26E-13 | 6.53E-14 |
|  | KO young | 6 | 1.94E-12 | 2.95E-13 | 1.20E-13 | 6 | 2.21E-12 | 4.72E-13 | 1.93E-13 |
|  | KO old | 9 | 1.64E-12 | 3.48E-13 | 1.16E-13 | 9 | 1.95E-12 | 2.63E-13 | 8.78E-14 |
|  | ABHD12 KO | 15 | 1.76E-12 | 3.52E-13 | 9.09E-14 | 15 | 2.05E-12 | 3.69E-13 | 9.53E-14 |
|  | Young | 12 | 1.88E-12 | 3.55E-13 | 1.03E-13 | 12 | 2.11E-12 | 3.65E-13 | 1.05E-13 |
|  | Old | 15 | 1.68E-12 | 3.37E-13 | 8.71E-14 | 15 | 1.90E-12 | 2.42E-13 | 6.24E-14 |
| *N*-oleoyl methionine | WT young | 6 | 8.83E-13 | 3.83E-13 | 1.56E-13 | 6 | 1.11E-12 | 3.60E-13 | 1.47E-13 |
|  | WT old | 6 | 8.87E-13 | 2.13E-13 | 8.71E-14 | 6 | 7.86E-13 | 2.96E-13 | 1.21E-13 |
|  | WT | 12 | 8.85E-13 | 2.95E-13 | 8.53E-14 | 12 | 9.46E-13 | 3.56E-13 | 1.03E-13 |
|  | KO young | 6 | 9.83E-13 | 4.22E-13 | 1.72E-13 | 6 | 1.27E-12 | 2.73E-13 | 1.11E-13 |
|  | KO old | 9 | 9.05E-13 | 4.24E-13 | 1.41E-13 | 9 | 7.88E-13 | 1.45E-13 | 4.83E-14 |
|  | ABHD12 KO | 15 | 9.36E-13 | 4.10E-13 | 1.06E-13 | 15 | 9.79E-13 | 3.13E-13 | 8.07E-14 |
|  | Young | 12 | 9.33E-13 | 3.88E-13 | 1.12E-13 | 12 | 1.19E-12 | 3.16E-13 | 9.11E-14 |
|  | Old | 15 | 8.98E-13 | 3.45E-13 | 8.91E-14 | 15 | 7.87E-13 | 2.08E-13 | 5.37E-14 |
| *N*-linoleoyl methionine | WT young | 6 | BDL |  |  | 6 | BDL |  |  |
|  | WT old | 6 | BDL |  |  | 6 | BDL |  |  |
|  | WT | 12 | BDL |  |  | 12 | BDL |  |  |
|  | KO young | 6 | BDL |  |  | 6 | BDL |  |  |
|  | KO old | 9 | BDL |  |  | 9 | BDL |  |  |
|  | ABHD12 KO | 15 | BDL |  |  | 15 | BDL |  |  |
|  | Young | 12 | BDL |  |  | 12 | BDL |  |  |
|  | Old | 15 | BDL |  |  | 15 | BDL |  |  |
| *N*-arachidonoyl methionine | WT young | 6 | PISSR |  |  | 6 | PISSR |  |  |
|  | WT old | 6 | PISSR |  |  | 6 | PISSR |  |  |
|  | WT | 12 | PISSR |  |  | 12 | PISSR |  |  |
|  | KO young | 6 | PISSR |  |  | 6 | PISSR |  |  |
|  | KO old | 9 | PISSR |  |  | 9 | PISSR |  |  |
|  | ABHD12 KO | 15 | PISSR |  |  | 15 | PISSR |  |  |
|  | Young | 12 | PISSR |  |  | 12 | PISSR |  |  |
|  | Old | 15 | PISSR |  |  | 15 | PISSR |  |  |
| *N*-docosahexaenoyl methionine | WT young | 6 | BDL |  |  | 6 | BDL |  |  |
|  | WT old | 6 | BDL |  |  | 6 | BDL |  |  |
|  | WT | 12 | BDL |  |  | 12 | BDL |  |  |
|  | KO young | 6 | BDL |  |  | 6 | BDL |  |  |
|  | KO old | 9 | BDL |  |  | 9 | BDL |  |  |
|  | ABHD12 KO | 15 | BDL |  |  | 15 | BDL |  |  |
|  | Young | 12 | BDL |  |  | 12 | BDL |  |  |
|  | Old | 15 | BDL |  |  | 15 | BDL |  |  |

Supplemental Table 26: Mean levels of *N*-acyl phenylalanines in the striatum and hippocampus

| Lipid Species | Group | Striatum | | | | Hippocampus | | | |
| --- | --- | --- | --- | --- | --- | --- | --- | --- | --- |
|  |  | N | Mean | SD | SE | N | Mean | SD | SE |
| *N*-palmitoyl phenylalanine | WT young | 6 | 2.37E-12 | 4.75E-13 | 1.94E-13 | 6 | 1.50E-12 | 1.02E-13 | 4.16E-14 |
|  | WT old | 6 | 2.30E-12 | 5.50E-13 | 2.24E-13 | 6 | 1.32E-12 | 1.51E-13 | 6.18E-14 |
|  | WT | 12 | 2.33E-12 | 4.92E-13 | 1.42E-13 | 12 | 1.41E-12 | 1.56E-13 | 4.49E-14 |
|  | KO young | 6 | 2.95E-12 | 1.37E-12 | 5.59E-13 | 6 | 1.74E-12 | 7.58E-13 | 3.10E-13 |
|  | KO old | 9 | 2.23E-12 | 3.88E-13 | 1.29E-13 | 9 | 1.35E-12 | 1.35E-13 | 4.50E-14 |
|  | ABHD12 KO | 15 | 2.52E-12 | 9.43E-13 | 2.44E-13 | 15 | 1.51E-12 | 5.05E-13 | 1.30E-13 |
|  | Young | 12 | 2.66E-12 | 1.02E-12 | 2.96E-13 | 12 | 1.62E-12 | 5.31E-13 | 1.53E-13 |
|  | Old | 15 | 2.26E-12 | 4.42E-13 | 1.14E-13 | 15 | 1.34E-12 | 1.37E-13 | 3.54E-14 |
| *N*-stearoyl phenylalanine | WT young | 6 | 3.65E-12 | 7.97E-13 | 3.25E-13 | 6 | 2.08E-12 | 2.09E-13 | 8.54E-14 |
|  | WT old | 6 | 3.38E-12 | 5.16E-13 | 2.11E-13 | 6 | 1.95E-12 | 1.53E-13 | 6.25E-14 |
|  | WT | 12 | 3.52E-12 | 6.55E-13 | 1.89E-13 | 12 | 2.01E-12 | 1.87E-13 | 5.41E-14 |
|  | KO young | 6 | 4.69E-12 | 1.62E-12 | 6.62E-13 | 6 | 2.61E-12 | 9.72E-13 | 3.97E-13 |
|  | KO old | 9 | 3.72E-12 | 4.89E-13 | 1.63E-13 | 9 | 2.11E-12 | 3.63E-13 | 1.21E-13 |
|  | ABHD12 KO | 15 | 4.11E-12 | 1.15E-12 | 2.97E-13 | 15 | 2.31E-12 | 6.91E-13 | 1.79E-13 |
|  | Young | 12 | 4.17E-12 | 1.33E-12 | 3.85E-13 | 12 | 2.35E-12 | 7.26E-13 | 2.10E-13 |
|  | Old | 15 | 3.58E-12 | 5.10E-13 | 1.32E-13 | 15 | 2.05E-12 | 3.01E-13 | 7.76E-14 |
| *N*-oleoyl phenylalanine | WT young | 6 | 1.50E-12 | 2.07E-13 | 8.43E-14 | 6 | 8.36E-13 | 6.48E-14 | 2.64E-14 |
|  | WT old | 6 | 1.47E-12 | 3.16E-13 | 1.29E-13 | 6 | 7.72E-13 | 7.26E-14 | 2.97E-14 |
|  | WT | 12 | 1.49E-12 | 2.55E-13 | 7.35E-14 | 12 | 8.04E-13 | 7.37E-14 | 2.13E-14 |
|  | KO young | 6 | 1.68E-12 | 8.38E-13 | 3.42E-13 | 6 | 1.10E-12 | 3.70E-13 | 1.51E-13 |
|  | KO old | 9 | 1.32E-12 | 2.58E-13 | 8.60E-14 | 9 | 8.91E-13 | 7.82E-14 | 2.61E-14 |
|  | ABHD12 KO | 15 | 1.47E-12 | 5.67E-13 | 1.46E-13 | 15 | 9.76E-13 | 2.53E-13 | 6.54E-14 |
|  | Young | 12 | 1.59E-12 | 5.89E-13 | 1.70E-13 | 12 | 9.70E-13 | 2.90E-13 | 8.36E-14 |
|  | Old | 15 | 1.38E-12 | 2.82E-13 | 7.28E-14 | 15 | 8.43E-13 | 9.51E-14 | 2.46E-14 |
| *N*-linoleoyl phenylalanine | WT young | 6 | PISSR |  |  | 6 | PISSR |  |  |
|  | WT old | 6 | PISSR |  |  | 6 | PISSR |  |  |
|  | WT | 12 | PISSR |  |  | 12 | PISSR |  |  |
|  | KO young | 6 | PISSR |  |  | 6 | PISSR |  |  |
|  | KO old | 9 | PISSR |  |  | 9 | PISSR |  |  |
|  | ABHD12 KO | 15 | PISSR |  |  | 15 | PISSR |  |  |
|  | Young | 12 | PISSR |  |  | 12 | PISSR |  |  |
|  | Old | 15 | PISSR |  |  | 15 | PISSR |  |  |
| *N*-arachidonoyl phenylalanine | WT young | 6 | 1.10E-12 | 2.66E-13 | 1.09E-13 | 6 | 6.79E-13 | 1.32E-13 | 5.40E-14 |
|  | WT old | 6 | 1.04E-12 | 3.21E-13 | 1.31E-13 | 6 | 6.63E-13 | 9.03E-14 | 3.69E-14 |
|  | WT | 12 | 1.07E-12 | 2.83E-13 | 8.17E-14 | 12 | 6.71E-13 | 1.08E-13 | 3.13E-14 |
|  | KO young | 6 | 1.48E-12 | 7.15E-13 | 2.92E-13 | 6 | 8.21E-13 | 3.17E-13 | 1.29E-13 |
|  | KO old | 9 | 1.11E-12 | 2.29E-13 | 7.65E-14 | 9 | 8.25E-13 | 9.01E-14 | 3.00E-14 |
|  | ABHD12 KO | 15 | 1.26E-12 | 4.98E-13 | 1.29E-13 | 15 | 8.23E-13 | 2.01E-13 | 5.20E-14 |
|  | Young | 12 | 1.29E-12 | 5.50E-13 | 1.59E-13 | 12 | 7.50E-13 | 2.43E-13 | 7.02E-14 |
|  | Old | 15 | 1.08E-12 | 2.61E-13 | 6.74E-14 | 15 | 7.60E-13 | 1.19E-13 | 3.08E-14 |
| *N*-docosahexaenoyl phenylalanine | WT young | 6 | 9.95E-13 | 3.78E-13 | 1.54E-13 | 6 | 7.54E-13 | 2.42E-13 | 9.87E-14 |
|  | WT old | 6 | 7.77E-13 | 2.85E-13 | 1.16E-13 | 6 | 6.18E-13 | 1.70E-13 | 6.96E-14 |
|  | WT | 12 | 8.86E-13 | 3.39E-13 | 9.79E-14 | 12 | 6.86E-13 | 2.12E-13 | 6.11E-14 |
|  | KO young | 6 | 1.31E-12 | 6.22E-13 | 2.54E-13 | 6 | 9.34E-13 | 3.24E-13 | 1.32E-13 |
|  | KO old | 9 | 9.05E-13 | 2.61E-13 | 8.71E-14 | 9 | 6.48E-13 | 1.26E-13 | 4.19E-14 |
|  | ABHD12 KO | 15 | 1.07E-12 | 4.69E-13 | 1.21E-13 | 15 | 7.62E-13 | 2.60E-13 | 6.70E-14 |
|  | Young | 12 | 1.15E-12 | 5.18E-13 | 1.50E-13 | 12 | 8.44E-13 | 2.88E-13 | 8.32E-14 |
|  | Old | 15 | 8.54E-13 | 2.69E-13 | 6.94E-14 | 15 | 6.36E-13 | 1.40E-13 | 3.62E-14 |

Supplemental Table 27: Mean levels of *N*-acyl phenylalanines in the cerebellum and thalamus

| Lipid Species | Group | Cerebellum | | | | Thalamus | | | |
| --- | --- | --- | --- | --- | --- | --- | --- | --- | --- |
|  |  | N | Mean | SD | SE | N | Mean | SD | SE |
| *N*-palmitoyl phenylalanine | WT young | 6 | 1.44E-12 | 3.81E-13 | 1.56E-13 | 6 | 9.91E-13 | 9.92E-14 | 4.05E-14 |
|  | WT old | 6 | 1.35E-12 | 2.96E-13 | 1.21E-13 | 6 | 1.02E-12 | 2.03E-13 | 8.29E-14 |
|  | WT | 12 | 1.40E-12 | 3.29E-13 | 9.49E-14 | 12 | 1.00E-12 | 1.53E-13 | 4.41E-14 |
|  | KO young | 6 | 1.86E-12 | 8.37E-13 | 3.42E-13 | 6 | 1.40E-12 | 4.15E-13 | 1.69E-13 |
|  | KO old | 9 | 1.25E-12 | 1.82E-13 | 6.08E-14 | 9 | 1.12E-12 | 2.82E-13 | 9.40E-14 |
|  | ABHD12 KO | 15 | 1.50E-12 | 6.05E-13 | 1.56E-13 | 15 | 1.23E-12 | 3.56E-13 | 9.20E-14 |
|  | Young | 12 | 1.65E-12 | 6.58E-13 | 1.90E-13 | 12 | 1.20E-12 | 3.58E-13 | 1.03E-13 |
|  | Old | 15 | 1.29E-12 | 2.30E-13 | 5.94E-14 | 15 | 1.08E-12 | 2.51E-13 | 6.48E-14 |
| *N*-stearoyl phenylalanine | WT young | 6 | 2.17E-12 | 2.51E-13 | 1.02E-13 | 6 | 1.43E-12 | 2.61E-13 | 1.07E-13 |
|  | WT old | 6 | 2.16E-12 | 5.48E-14 | 2.24E-14 | 6 | 1.44E-12 | 1.59E-13 | 6.49E-14 |
|  | WT | 12 | 2.16E-12 | 1.73E-13 | 5.00E-14 | 12 | 1.44E-12 | 2.06E-13 | 5.96E-14 |
|  | KO young | 6 | 2.72E-12 | 4.75E-13 | 1.94E-13 | 6 | 2.22E-12 | 8.02E-13 | 3.27E-13 |
|  | KO old | 9 | 2.09E-12 | 2.56E-13 | 8.52E-14 | 9 | 1.60E-12 | 3.52E-13 | 1.17E-13 |
|  | ABHD12 KO | 15 | 2.34E-12 | 4.69E-13 | 1.21E-13 | 15 | 1.85E-12 | 6.30E-13 | 1.63E-13 |
|  | Young | 12 | 2.44E-12 | 4.63E-13 | 1.34E-13 | 12 | 1.82E-12 | 7.02E-13 | 2.03E-13 |
|  | Old | 15 | 2.12E-12 | 1.99E-13 | 5.15E-14 | 15 | 1.54E-12 | 2.94E-13 | 7.60E-14 |
| *N*-oleoyl phenylalanine | WT young | 6 | 1.13E-12 | 1.52E-13 | 6.19E-14 | 6 | 6.39E-13 | 4.91E-14 | 2.01E-14 |
|  | WT old | 6 | 1.13E-12 | 1.97E-13 | 8.04E-14 | 6 | 6.63E-13 | 1.01E-13 | 4.13E-14 |
|  | WT | 12 | 1.13E-12 | 1.68E-13 | 4.84E-14 | 12 | 6.51E-13 | 7.68E-14 | 2.22E-14 |
|  | KO young | 6 | 1.51E-12 | 7.30E-13 | 2.98E-13 | 6 | 9.39E-13 | 3.03E-13 | 1.24E-13 |
|  | KO old | 9 | 1.09E-12 | 1.68E-13 | 5.60E-14 | 9 | 7.32E-13 | 1.08E-13 | 3.58E-14 |
|  | ABHD12 KO | 15 | 1.26E-12 | 5.03E-13 | 1.30E-13 | 15 | 8.15E-13 | 2.25E-13 | 5.80E-14 |
|  | Young | 12 | 1.32E-12 | 5.41E-13 | 1.56E-13 | 12 | 7.89E-13 | 2.60E-13 | 7.50E-14 |
|  | Old | 15 | 1.10E-12 | 1.75E-13 | 4.51E-14 | 15 | 7.05E-13 | 1.07E-13 | 2.77E-14 |
| *N*-linoleoyl phenylalanine | WT young | 6 | 1.76E-13 | 8.36E-14 | 3.41E-14 | 6 | PISSR |  |  |
|  | WT old | 6 | 9.89E-14 | 7.30E-14 | 2.98E-14 | 6 | PISSR |  |  |
|  | WT | 12 | 1.38E-13 | 8.51E-14 | 2.46E-14 | 12 | PISSR |  |  |
|  | KO young | 6 | 1.92E-13 | 9.81E-14 | 4.00E-14 | 6 | PISSR |  |  |
|  | KO old | 9 | 1.55E-13 | 1.00E-13 | 3.34E-14 | 9 | PISSR |  |  |
|  | ABHD12 KO | 15 | 1.70E-13 | 9.77E-14 | 2.52E-14 | 15 | PISSR |  |  |
|  | Young | 12 | 1.84E-13 | 8.73E-14 | 2.52E-14 | 12 | PISSR |  |  |
|  | Old | 15 | 1.33E-13 | 9.20E-14 | 2.38E-14 | 15 | PISSR |  |  |
| *N*-arachidonoyl phenylalanine | WT young | 6 | 4.66E-13 | 1.53E-13 | 6.26E-14 | 6 | 2.97E-13 | 5.26E-14 | 2.15E-14 |
|  | WT old | 6 | 5.74E-13 | 1.59E-13 | 6.49E-14 | 6 | 4.14E-13 | 1.66E-13 | 6.79E-14 |
|  | WT | 12 | 5.20E-13 | 1.59E-13 | 4.60E-14 | 12 | 3.56E-13 | 1.33E-13 | 3.83E-14 |
|  | KO young | 6 | 1.05E-12 | 2.77E-13 | 1.13E-13 | 6 | 6.60E-13 | 1.46E-13 | 5.98E-14 |
|  | KO old | 9 | 6.10E-13 | 2.48E-13 | 8.27E-14 | 9 | 5.99E-13 | 1.43E-13 | 4.78E-14 |
|  | ABHD12 KO | 15 | 7.84E-13 | 3.34E-13 | 8.61E-14 | 15 | 6.23E-13 | 1.43E-13 | 3.68E-14 |
|  | Young | 12 | 7.55E-13 | 3.70E-13 | 1.07E-13 | 12 | 4.78E-13 | 2.17E-13 | 6.25E-14 |
|  | Old | 15 | 5.95E-13 | 2.11E-13 | 5.45E-14 | 15 | 5.25E-13 | 1.74E-13 | 4.50E-14 |
| *N*-docosahexaenoyl phenylalanine | WT young | 6 | 6.50E-13 | 3.02E-13 | 1.23E-13 | 6 | 3.16E-13 | 8.46E-14 | 3.45E-14 |
|  | WT old | 6 | 6.86E-13 | 2.61E-13 | 1.06E-13 | 6 | 3.95E-13 | 1.33E-13 | 5.44E-14 |
|  | WT | 12 | 6.68E-13 | 2.69E-13 | 7.78E-14 | 12 | 3.55E-13 | 1.14E-13 | 3.29E-14 |
|  | KO young | 6 | 8.86E-13 | 3.26E-13 | 1.33E-13 | 6 | 4.96E-13 | 1.24E-13 | 5.08E-14 |
|  | KO old | 9 | 6.81E-13 | 2.32E-13 | 7.72E-14 | 9 | 4.14E-13 | 1.47E-13 | 4.91E-14 |
|  | ABHD12 KO | 15 | 7.63E-13 | 2.82E-13 | 7.27E-14 | 15 | 4.47E-13 | 1.40E-13 | 3.62E-14 |
|  | Young | 12 | 7.68E-13 | 3.24E-13 | 9.34E-14 | 12 | 4.06E-13 | 1.38E-13 | 3.99E-14 |
|  | Old | 15 | 6.83E-13 | 2.34E-13 | 6.05E-14 | 15 | 4.06E-13 | 1.37E-13 | 3.55E-14 |

Supplemental Table 28: Mean levels of *N*-acyl phenylalanines in the cortex and hypothalamus

| Lipid Species | Group | Cortex | | | | Hypothalamus | | | |
| --- | --- | --- | --- | --- | --- | --- | --- | --- | --- |
|  |  | N | Mean | SD | SE | N | Mean | SD | SE |
| *N*-palmitoyl phenylalanine | WT young | 6 | 1.61E-12 | 9.64E-13 | 3.94E-13 | 6 | 1.28E-12 | 1.58E-13 | 6.46E-14 |
|  | WT old | 6 | 7.97E-13 | 1.29E-13 | 5.27E-14 | 5 | 1.22E-12 | 3.56E-13 | 1.59E-13 |
|  | WT | 12 | 1.20E-12 | 7.81E-13 | 2.25E-13 | 11 | 1.25E-12 | 2.54E-13 | 7.66E-14 |
|  | KO young | 6 | 1.98E-12 | 9.42E-13 | 3.84E-13 | 6 | 1.43E-12 | 5.19E-13 | 2.12E-13 |
|  | KO old | 9 | 8.95E-13 | 2.08E-13 | 6.95E-14 | 7 | 9.90E-13 | 1.27E-13 | 4.79E-14 |
|  | ABHD12 KO | 15 | 1.33E-12 | 8.02E-13 | 2.07E-13 | 13 | 1.19E-12 | 4.15E-13 | 1.15E-13 |
|  | Young | 12 | 1.79E-12 | 9.29E-13 | 2.68E-13 | 12 | 1.36E-12 | 3.74E-13 | 1.08E-13 |
|  | Old | 15 | 8.56E-13 | 1.82E-13 | 4.71E-14 | 12 | 1.08E-12 | 2.62E-13 | 7.56E-14 |
| *N*-stearoyl phenylalanine | WT young | 6 | 3.56E-12 | 1.22E-12 | 4.99E-13 | 6 | 1.38E-12 | 1.90E-13 | 7.75E-14 |
|  | WT old | 6 | 2.18E-12 | 1.23E-13 | 5.03E-14 | 5 | 1.18E-12 | 2.06E-13 | 9.23E-14 |
|  | WT | 12 | 2.87E-12 | 1.10E-12 | 3.17E-13 | 11 | 1.29E-12 | 2.13E-13 | 6.43E-14 |
|  | KO young | 6 | 4.34E-12 | 1.37E-12 | 5.61E-13 | 6 | 1.66E-12 | 2.98E-13 | 1.22E-13 |
|  | KO old | 9 | 2.41E-12 | 2.07E-13 | 6.92E-14 | 7 | 1.14E-12 | 2.97E-13 | 1.12E-13 |
|  | ABHD12 KO | 15 | 3.18E-12 | 1.29E-12 | 3.32E-13 | 13 | 1.38E-12 | 3.92E-13 | 1.09E-13 |
|  | Young | 12 | 3.95E-12 | 1.30E-12 | 3.76E-13 | 12 | 1.52E-12 | 2.80E-13 | 8.08E-14 |
|  | Old | 15 | 2.32E-12 | 2.09E-13 | 5.39E-14 | 12 | 1.16E-12 | 2.53E-13 | 7.31E-14 |
| *N*-oleoyl phenylalanine | WT young | 6 | 1.09E-12 | 5.66E-13 | 2.31E-13 | 6 | 7.14E-13 | 8.20E-14 | 3.35E-14 |
|  | WT old | 6 | 5.67E-13 | 1.31E-13 | 5.34E-14 | 5 | 7.57E-13 | 1.65E-13 | 7.39E-14 |
|  | WT | 12 | 8.28E-13 | 4.77E-13 | 1.38E-13 | 11 | 7.34E-13 | 1.22E-13 | 3.67E-14 |
|  | KO young | 6 | 1.54E-12 | 7.21E-13 | 2.94E-13 | 6 | 6.36E-13 | 2.12E-13 | 8.66E-14 |
|  | KO old | 9 | 6.69E-13 | 8.48E-14 | 2.83E-14 | 7 | 5.14E-13 | 1.22E-13 | 4.62E-14 |
|  | ABHD12 KO | 15 | 1.02E-12 | 6.19E-13 | 1.60E-13 | 13 | 5.70E-13 | 1.74E-13 | 4.82E-14 |
|  | Young | 12 | 1.31E-12 | 6.60E-13 | 1.91E-13 | 12 | 6.75E-13 | 1.59E-13 | 4.58E-14 |
|  | Old | 15 | 6.28E-13 | 1.14E-13 | 2.93E-14 | 12 | 6.15E-13 | 1.84E-13 | 5.31E-14 |
| *N*-linoleoyl phenylalanine | WT young | 6 | 6.23E-14 | 1.94E-14 | 7.93E-15 | 6 | PISSR |  |  |
|  | WT old | 6 | 4.70E-14 | 1.51E-14 | 6.17E-15 | 5 | PISSR |  |  |
|  | WT | 12 | 5.46E-14 | 1.84E-14 | 5.31E-15 | 11 | PISSR |  |  |
|  | KO young | 6 | 1.23E-13 | 4.17E-14 | 1.70E-14 | 6 | PISSR |  |  |
|  | KO old | 9 | 6.50E-14 | 2.03E-14 | 6.77E-15 | 7 | PISSR |  |  |
|  | ABHD12 KO | 15 | 8.81E-14 | 4.14E-14 | 1.07E-14 | 13 | PISSR |  |  |
|  | Young | 12 | 9.25E-14 | 4.43E-14 | 1.28E-14 | 12 | PISSR |  |  |
|  | Old | 15 | 5.78E-14 | 2.00E-14 | 5.17E-15 | 12 | PISSR |  |  |
| *N*-arachidonoyl phenylalanine | WT young | 6 | 3.25E-13 | 7.61E-14 | 3.11E-14 | 6 | 4.77E-13 | 2.22E-13 | 9.05E-14 |
|  | WT old | 6 | 2.15E-13 | 4.44E-14 | 1.81E-14 | 5 | 4.60E-13 | 2.14E-13 | 9.59E-14 |
|  | WT | 12 | 2.70E-13 | 8.28E-14 | 2.39E-14 | 11 | 4.70E-13 | 2.07E-13 | 6.25E-14 |
|  | KO young | 6 | 6.32E-13 | 1.40E-13 | 5.71E-14 | 6 | 7.14E-13 | 2.53E-13 | 1.03E-13 |
|  | KO old | 9 | 3.31E-13 | 6.45E-14 | 2.15E-14 | 7 | 4.11E-13 | 1.37E-13 | 5.18E-14 |
|  | ABHD12 KO | 15 | 4.51E-13 | 1.81E-13 | 4.67E-14 | 13 | 5.51E-13 | 2.46E-13 | 6.83E-14 |
|  | Young | 12 | 4.78E-13 | 1.93E-13 | 5.57E-14 | 12 | 5.96E-13 | 2.58E-13 | 7.46E-14 |
|  | Old | 15 | 2.84E-13 | 8.09E-14 | 2.09E-14 | 12 | 4.32E-13 | 1.66E-13 | 4.79E-14 |
| *N*-docosahexaenoyl phenylalanine | WT young | 6 | 2.71E-13 | 4.50E-14 | 1.84E-14 | 6 | PISSR |  |  |
|  | WT old | 6 | 3.17E-13 | 7.66E-14 | 3.13E-14 | 5 | PISSR |  |  |
|  | WT | 12 | 2.94E-13 | 6.45E-14 | 1.86E-14 | 11 | PISSR |  |  |
|  | KO young | 6 | 5.16E-13 | 1.47E-13 | 6.00E-14 | 6 | PISSR |  |  |
|  | KO old | 9 | 3.04E-13 | 1.18E-13 | 3.93E-14 | 7 | PISSR |  |  |
|  | ABHD12 KO | 15 | 3.89E-13 | 1.65E-13 | 4.26E-14 | 13 | PISSR |  |  |
|  | Young | 12 | 3.94E-13 | 1.65E-13 | 4.75E-14 | 12 | PISSR |  |  |
|  | Old | 15 | 3.09E-13 | 1.00E-13 | 2.59E-14 | 12 | PISSR |  |  |

Supplemental Table 29: Mean levels of *N*-acyl phenylalanines in the midbrain and brainstem

| Lipid Species | Group | Midbrain | | | | Brainstem | | | |
| --- | --- | --- | --- | --- | --- | --- | --- | --- | --- |
|  |  | N | Mean | SD | SE | N | Mean | SD | SE |
| *N*-palmitoyl phenylalanine | WT young | 6 | 1.21E-12 | 2.94E-13 | 1.20E-13 | 6 | 8.00E-13 | 9.48E-14 | 3.87E-14 |
|  | WT old | 6 | 1.33E-12 | 1.58E-13 | 6.44E-14 | 6 | 9.75E-13 | 1.74E-13 | 7.10E-14 |
|  | WT | 12 | 1.27E-12 | 2.33E-13 | 6.74E-14 | 12 | 8.88E-13 | 1.62E-13 | 4.67E-14 |
|  | KO young | 6 | 1.92E-12 | 6.65E-13 | 2.71E-13 | 6 | 9.47E-13 | 2.84E-13 | 1.16E-13 |
|  | KO old | 9 | 1.26E-12 | 1.33E-13 | 4.43E-14 | 9 | 1.03E-12 | 2.13E-13 | 7.09E-14 |
|  | ABHD12 KO | 15 | 1.53E-12 | 5.27E-13 | 1.36E-13 | 15 | 9.94E-13 | 2.37E-13 | 6.13E-14 |
|  | Young | 12 | 1.57E-12 | 6.13E-13 | 1.77E-13 | 12 | 8.73E-13 | 2.16E-13 | 6.24E-14 |
|  | Old | 15 | 1.29E-12 | 1.42E-13 | 3.67E-14 | 15 | 1.01E-12 | 1.93E-13 | 4.99E-14 |
| *N*-stearoyl phenylalanine | WT young | 6 | 1.91E-12 | 3.08E-13 | 1.26E-13 | 6 | 1.04E-12 | 1.40E-13 | 5.72E-14 |
|  | WT old | 6 | 1.71E-12 | 1.75E-13 | 7.15E-14 | 6 | 1.35E-12 | 2.14E-13 | 8.75E-14 |
|  | WT | 12 | 1.81E-12 | 2.61E-13 | 7.55E-14 | 12 | 1.19E-12 | 2.38E-13 | 6.88E-14 |
|  | KO young | 6 | 2.59E-12 | 1.03E-12 | 4.22E-13 | 6 | 1.39E-12 | 4.06E-13 | 1.66E-13 |
|  | KO old | 9 | 1.90E-12 | 3.05E-13 | 1.02E-13 | 9 | 1.51E-12 | 3.23E-13 | 1.08E-13 |
|  | ABHD12 KO | 15 | 2.17E-12 | 7.45E-13 | 1.92E-13 | 15 | 1.46E-12 | 3.50E-13 | 9.03E-14 |
|  | Young | 12 | 2.25E-12 | 8.07E-13 | 2.33E-13 | 12 | 1.21E-12 | 3.44E-13 | 9.93E-14 |
|  | Old | 15 | 1.82E-12 | 2.71E-13 | 7.00E-14 | 15 | 1.45E-12 | 2.88E-13 | 7.43E-14 |
| *N*-oleoyl phenylalanine | WT young | 6 | 9.14E-13 | 1.86E-13 | 7.61E-14 | 6 | 5.97E-13 | 8.05E-14 | 3.29E-14 |
|  | WT old | 6 | 9.02E-13 | 7.43E-14 | 3.03E-14 | 6 | 6.95E-13 | 1.33E-13 | 5.42E-14 |
|  | WT | 12 | 9.08E-13 | 1.35E-13 | 3.91E-14 | 12 | 6.46E-13 | 1.17E-13 | 3.36E-14 |
|  | KO young | 6 | 1.41E-12 | 6.94E-13 | 2.83E-13 | 6 | 6.76E-13 | 1.32E-13 | 5.38E-14 |
|  | KO old | 9 | 9.52E-13 | 1.26E-13 | 4.20E-14 | 9 | 7.73E-13 | 1.35E-13 | 4.51E-14 |
|  | ABHD12 KO | 15 | 1.13E-12 | 4.84E-13 | 1.25E-13 | 15 | 7.34E-13 | 1.38E-13 | 3.57E-14 |
|  | Young | 12 | 1.16E-12 | 5.49E-13 | 1.58E-13 | 12 | 6.36E-13 | 1.12E-13 | 3.23E-14 |
|  | Old | 15 | 9.32E-13 | 1.08E-13 | 2.79E-14 | 15 | 7.42E-13 | 1.35E-13 | 3.50E-14 |
| *N*-linoleoyl phenylalanine | WT young | 6 | 1.13E-13 | 8.81E-14 | 3.60E-14 | 6 | PISSR |  |  |
|  | WT old | 6 | 9.74E-14 | 6.57E-14 | 2.68E-14 | 6 | PISSR |  |  |
|  | WT | 12 | 1.05E-13 | 7.45E-14 | 2.15E-14 | 12 | PISSR |  |  |
|  | KO young | 6 | 1.25E-13 | 6.01E-14 | 2.45E-14 | 6 | PISSR |  |  |
|  | KO old | 9 | 1.11E-13 | 3.81E-14 | 1.27E-14 | 9 | PISSR |  |  |
|  | ABHD12 KO | 15 | 1.17E-13 | 4.66E-14 | 1.20E-14 | 15 | PISSR |  |  |
|  | Young | 12 | 1.19E-13 | 7.22E-14 | 2.08E-14 | 12 | PISSR |  |  |
|  | Old | 15 | 1.05E-13 | 4.91E-14 | 1.27E-14 | 15 | PISSR |  |  |
| *N*-arachidonoyl phenylalanine | WT young | 6 | 3.91E-13 | 1.64E-13 | 6.68E-14 | 6 | 2.88E-13 | 8.16E-14 | 3.33E-14 |
|  | WT old | 6 | 4.89E-13 | 1.25E-13 | 5.09E-14 | 6 | 3.20E-13 | 1.00E-13 | 4.10E-14 |
|  | WT | 12 | 4.40E-13 | 1.48E-13 | 4.27E-14 | 12 | 3.04E-13 | 8.89E-14 | 2.57E-14 |
|  | KO young | 6 | 9.32E-13 | 2.71E-13 | 1.11E-13 | 6 | 4.26E-13 | 7.28E-14 | 2.97E-14 |
|  | KO old | 9 | 7.23E-13 | 1.16E-13 | 3.87E-14 | 9 | 4.79E-13 | 1.80E-13 | 6.01E-14 |
|  | ABHD12 KO | 15 | 8.07E-13 | 2.12E-13 | 5.48E-14 | 15 | 4.58E-13 | 1.45E-13 | 3.76E-14 |
|  | Young | 12 | 6.62E-13 | 3.54E-13 | 1.02E-13 | 12 | 3.57E-13 | 1.03E-13 | 2.98E-14 |
|  | Old | 15 | 6.30E-13 | 1.66E-13 | 4.28E-14 | 15 | 4.15E-13 | 1.69E-13 | 4.37E-14 |
| *N*-docosahexaenoyl phenylalanine | WT young | 6 | 3.51E-13 | 1.39E-13 | 5.68E-14 | 6 | 2.78E-13 | 7.49E-14 | 3.06E-14 |
|  | WT old | 6 | 5.38E-13 | 9.11E-14 | 3.72E-14 | 6 | 3.26E-13 | 8.32E-14 | 3.40E-14 |
|  | WT | 12 | 4.45E-13 | 1.49E-13 | 4.29E-14 | 12 | 3.02E-13 | 7.95E-14 | 2.29E-14 |
|  | KO young | 6 | 6.37E-13 | 2.83E-13 | 1.16E-13 | 6 | 4.16E-13 | 9.99E-14 | 4.08E-14 |
|  | KO old | 9 | 5.34E-13 | 2.24E-13 | 7.47E-14 | 9 | 3.85E-13 | 1.50E-13 | 4.99E-14 |
|  | ABHD12 KO | 15 | 5.75E-13 | 2.45E-13 | 6.33E-14 | 15 | 3.98E-13 | 1.29E-13 | 3.33E-14 |
|  | Young | 12 | 4.94E-13 | 2.60E-13 | 7.50E-14 | 12 | 3.47E-13 | 1.11E-13 | 3.19E-14 |
|  | Old | 15 | 5.35E-13 | 1.78E-13 | 4.60E-14 | 15 | 3.61E-13 | 1.27E-13 | 3.28E-14 |

Supplemental Table 30: Mean levels of *N*-acyl prolines in the striatum and hippocampus

| Lipid Species | Group | Striatum | | | | Hippocampus | | | |
| --- | --- | --- | --- | --- | --- | --- | --- | --- | --- |
|  |  | N | Mean | SD | SE | N | Mean | SD | SE |
| *N*-palmitoyl proline | WT young | 6 | 2.33E-12 | 3.36E-13 | 1.37E-13 | 6 | 1.22E-12 | 2.91E-13 | 1.19E-13 |
|  | WT old | 6 | 2.82E-12 | 1.43E-12 | 5.86E-13 | 6 | 1.42E-12 | 1.97E-13 | 8.03E-14 |
|  | WT | 12 | 2.58E-12 | 1.03E-12 | 2.96E-13 | 12 | 1.32E-12 | 2.60E-13 | 7.50E-14 |
|  | KO young | 6 | 2.29E-12 | 3.86E-13 | 1.57E-13 | 6 | 1.70E-12 | 3.88E-13 | 1.58E-13 |
|  | KO old | 9 | 2.74E-12 | 5.11E-13 | 1.70E-13 | 9 | 1.46E-12 | 3.55E-13 | 1.18E-13 |
|  | ABHD12 KO | 15 | 2.56E-12 | 5.04E-13 | 1.30E-13 | 15 | 1.55E-12 | 3.74E-13 | 9.66E-14 |
|  | Young | 12 | 2.31E-12 | 3.46E-13 | 9.98E-14 | 12 | 1.46E-12 | 4.10E-13 | 1.18E-13 |
|  | Old | 15 | 2.77E-12 | 9.42E-13 | 2.43E-13 | 15 | 1.45E-12 | 2.94E-13 | 7.58E-14 |
| *N*-stearoyl proline | WT young | 6 | 9.89E-13 | 1.51E-13 | 6.16E-14 | 6 | 4.88E-13 | 1.39E-13 | 5.68E-14 |
|  | WT old | 6 | 1.15E-12 | 6.94E-13 | 2.83E-13 | 6 | 5.31E-13 | 5.34E-14 | 2.18E-14 |
|  | WT | 12 | 1.07E-12 | 4.86E-13 | 1.40E-13 | 12 | 5.09E-13 | 1.03E-13 | 2.97E-14 |
|  | KO young | 6 | 9.94E-13 | 1.91E-13 | 7.79E-14 | 6 | 7.27E-13 | 6.31E-14 | 2.57E-14 |
|  | KO old | 9 | 9.10E-13 | 1.67E-13 | 5.57E-14 | 9 | 5.76E-13 | 1.25E-13 | 4.17E-14 |
|  | ABHD12 KO | 15 | 9.44E-13 | 1.75E-13 | 4.53E-14 | 15 | 6.36E-13 | 1.27E-13 | 3.29E-14 |
|  | Young | 12 | 9.91E-13 | 1.64E-13 | 4.73E-14 | 12 | 6.07E-13 | 1.62E-13 | 4.67E-14 |
|  | Old | 15 | 1.01E-12 | 4.50E-13 | 1.16E-13 | 15 | 5.58E-13 | 1.02E-13 | 2.64E-14 |
| *N*-oleoyl proline | WT young | 6 | PISSR |  |  | 6 | PISSR |  |  |
|  | WT old | 6 | PISSR |  |  | 6 | PISSR |  |  |
|  | WT | 12 | PISSR |  |  | 12 | PISSR |  |  |
|  | KO young | 6 | PISSR |  |  | 6 | PISSR |  |  |
|  | KO old | 9 | PISSR |  |  | 9 | PISSR |  |  |
|  | ABHD12 KO | 15 | PISSR |  |  | 15 | PISSR |  |  |
|  | Young | 12 | PISSR |  |  | 12 | PISSR |  |  |
|  | Old | 15 | PISSR |  |  | 15 | PISSR |  |  |
| *N*-linoleoyl proline | WT young | 6 | BDL |  |  | 6 | BDL |  |  |
|  | WT old | 6 | BDL |  |  | 6 | BDL |  |  |
|  | WT | 12 | BDL |  |  | 12 | BDL |  |  |
|  | KO young | 6 | BDL |  |  | 6 | BDL |  |  |
|  | KO old | 9 | BDL |  |  | 9 | BDL |  |  |
|  | ABHD12 KO | 15 | BDL |  |  | 15 | BDL |  |  |
|  | Young | 12 | BDL |  |  | 12 | BDL |  |  |
|  | Old | 15 | BDL |  |  | 15 | BDL |  |  |
| *N*-arachidonoyl proline | WT young | 6 | BDL |  |  | 6 | BDL |  |  |
|  | WT old | 6 | BDL |  |  | 6 | BDL |  |  |
|  | WT | 12 | BDL |  |  | 12 | BDL |  |  |
|  | KO young | 6 | BDL |  |  | 6 | BDL |  |  |
|  | KO old | 9 | BDL |  |  | 9 | BDL |  |  |
|  | ABHD12 KO | 15 | BDL |  |  | 15 | BDL |  |  |
|  | Young | 12 | BDL |  |  | 12 | BDL |  |  |
|  | Old | 15 | BDL |  |  | 15 | BDL |  |  |
| *N*-docosahexaenoyl proline | WT young | 6 | BDL |  |  | 6 | BDL |  |  |
|  | WT old | 6 | BDL |  |  | 6 | BDL |  |  |
|  | WT | 12 | BDL |  |  | 12 | BDL |  |  |
|  | KO young | 6 | BDL |  |  | 6 | BDL |  |  |
|  | KO old | 9 | BDL |  |  | 9 | BDL |  |  |
|  | ABHD12 KO | 15 | BDL |  |  | 15 | BDL |  |  |
|  | Young | 12 | BDL |  |  | 12 | BDL |  |  |
|  | Old | 15 | BDL |  |  | 15 | BDL |  |  |

Supplemental Table 31: Mean levels of *N*-acyl prolines in the cerebellum and thalamus

| Lipid Species | Group | Cerebellum | | | | Thalamus | | | |
| --- | --- | --- | --- | --- | --- | --- | --- | --- | --- |
|  |  | N | Mean | SD | SE | N | Mean | SD | SE |
| *N*-palmitoyl proline | WT young | 6 | 1.78E-12 | 6.55E-13 | 2.68E-13 | 6 | 1.26E-12 | 1.72E-13 | 7.00E-14 |
|  | WT old | 6 | 2.20E-12 | 5.03E-13 | 2.06E-13 | 6 | 1.44E-12 | 3.76E-13 | 1.54E-13 |
|  | WT | 12 | 1.99E-12 | 6.00E-13 | 1.73E-13 | 12 | 1.35E-12 | 2.94E-13 | 8.48E-14 |
|  | KO young | 6 | 1.89E-12 | 3.96E-13 | 1.62E-13 | 6 | 1.31E-12 | 3.12E-13 | 1.27E-13 |
|  | KO old | 9 | 1.92E-12 | 3.62E-13 | 1.21E-13 | 9 | 1.92E-12 | 1.29E-12 | 4.31E-13 |
|  | ABHD12 KO | 15 | 1.91E-12 | 3.62E-13 | 9.35E-14 | 15 | 1.68E-12 | 1.04E-12 | 2.69E-13 |
|  | Young | 12 | 1.84E-12 | 5.20E-13 | 1.50E-13 | 12 | 1.29E-12 | 2.42E-13 | 6.98E-14 |
|  | Old | 15 | 2.03E-12 | 4.31E-13 | 1.11E-13 | 15 | 1.73E-12 | 1.03E-12 | 2.67E-13 |
| *N*-stearoyl proline | WT young | 6 | 1.02E-12 | 1.47E-13 | 6.01E-14 | 6 | 5.05E-13 | 5.53E-14 | 2.26E-14 |
|  | WT old | 6 | 1.24E-12 | 1.27E-13 | 5.17E-14 | 6 | 5.57E-13 | 1.72E-13 | 7.02E-14 |
|  | WT | 12 | 1.13E-12 | 1.75E-13 | 5.05E-14 | 12 | 5.31E-13 | 1.25E-13 | 3.60E-14 |
|  | KO young | 6 | 1.06E-12 | 3.32E-13 | 1.36E-13 | 6 | 6.75E-13 | 1.05E-13 | 4.28E-14 |
|  | KO old | 9 | 1.17E-12 | 2.74E-13 | 9.15E-14 | 9 | 6.92E-13 | 3.28E-13 | 1.09E-13 |
|  | ABHD12 KO | 15 | 1.13E-12 | 2.92E-13 | 7.55E-14 | 15 | 6.85E-13 | 2.56E-13 | 6.61E-14 |
|  | Young | 12 | 1.04E-12 | 2.46E-13 | 7.10E-14 | 12 | 5.90E-13 | 1.19E-13 | 3.44E-14 |
|  | Old | 15 | 1.20E-12 | 2.24E-13 | 5.79E-14 | 15 | 6.38E-13 | 2.77E-13 | 7.16E-14 |
| *N*-oleoyl proline | WT young | 6 | 7.78E-13 | 1.60E-13 | 6.53E-14 | 6 | 5.01E-13 | 1.25E-13 | 5.11E-14 |
|  | WT old | 6 | 1.06E-12 | 2.78E-13 | 1.13E-13 | 6 | 5.11E-13 | 1.58E-13 | 6.44E-14 |
|  | WT | 12 | 9.20E-13 | 2.62E-13 | 7.57E-14 | 12 | 5.06E-13 | 1.36E-13 | 3.92E-14 |
|  | KO young | 6 | 8.70E-13 | 2.30E-13 | 9.38E-14 | 6 | 6.38E-13 | 2.41E-13 | 9.83E-14 |
|  | KO old | 9 | 9.46E-13 | 2.56E-13 | 8.54E-14 | 9 | 6.36E-13 | 2.21E-13 | 7.37E-14 |
|  | ABHD12 KO | 15 | 9.16E-13 | 2.41E-13 | 6.21E-14 | 15 | 6.37E-13 | 2.20E-13 | 5.69E-14 |
|  | Young | 12 | 8.24E-13 | 1.95E-13 | 5.62E-14 | 12 | 5.69E-13 | 1.96E-13 | 5.67E-14 |
|  | Old | 15 | 9.93E-13 | 2.62E-13 | 6.76E-14 | 15 | 5.86E-13 | 2.02E-13 | 5.22E-14 |
| *N*-linoleoyl proline | WT young | 6 | BDL |  |  | 6 | BDL |  |  |
|  | WT old | 6 | BDL |  |  | 6 | BDL |  |  |
|  | WT | 12 | BDL |  |  | 12 | BDL |  |  |
|  | KO young | 6 | BDL |  |  | 6 | BDL |  |  |
|  | KO old | 9 | BDL |  |  | 9 | BDL |  |  |
|  | ABHD12 KO | 15 | BDL |  |  | 15 | BDL |  |  |
|  | Young | 12 | BDL |  |  | 12 | BDL |  |  |
|  | Old | 15 | BDL |  |  | 15 | BDL |  |  |
| *N*-arachidonoyl proline | WT young | 6 | BDL |  |  | 6 | PISSR |  |  |
|  | WT old | 6 | BDL |  |  | 6 | PISSR |  |  |
|  | WT | 12 | BDL |  |  | 12 | PISSR |  |  |
|  | KO young | 6 | BDL |  |  | 6 | PISSR |  |  |
|  | KO old | 9 | BDL |  |  | 9 | PISSR |  |  |
|  | ABHD12 KO | 15 | BDL |  |  | 15 | PISSR |  |  |
|  | Young | 12 | BDL |  |  | 12 | PISSR |  |  |
|  | Old | 15 | BDL |  |  | 15 | PISSR |  |  |
| *N*-docosahexaenoyl proline | WT young | 6 | PISSR |  |  | 6 | BDL |  |  |
|  | WT old | 6 | PISSR |  |  | 6 | BDL |  |  |
|  | WT | 12 | PISSR |  |  | 12 | BDL |  |  |
|  | KO young | 6 | PISSR |  |  | 6 | BDL |  |  |
|  | KO old | 9 | PISSR |  |  | 9 | BDL |  |  |
|  | ABHD12 KO | 15 | PISSR |  |  | 15 | BDL |  |  |
|  | Young | 12 | PISSR |  |  | 12 | BDL |  |  |
|  | Old | 15 | PISSR |  |  | 15 | BDL |  |  |

Supplemental Table 32: Mean levels of *N*-acyl prolines in the cortex and hypothalamus

| Lipid Species | Group | Cortex | | | | Hypothalamus | | | |
| --- | --- | --- | --- | --- | --- | --- | --- | --- | --- |
|  |  | N | Mean | SD | SE | N | Mean | SD | SE |
| *N*-palmitoyl proline | WT young | 6 | 7.91E-13 | 1.05E-13 | 4.30E-14 | 6 | 2.36E-12 | 6.05E-13 | 2.47E-13 |
|  | WT old | 6 | 8.05E-13 | 2.40E-13 | 9.79E-14 | 5 | 2.38E-12 | 7.36E-13 | 3.29E-13 |
|  | WT | 12 | 7.98E-13 | 1.77E-13 | 5.10E-14 | 11 | 2.37E-12 | 6.33E-13 | 1.91E-13 |
|  | KO young | 6 | 8.00E-13 | 1.37E-13 | 5.61E-14 | 6 | 2.02E-12 | 3.73E-13 | 1.52E-13 |
|  | KO old | 9 | 1.01E-12 | 2.43E-13 | 8.11E-14 | 7 | 2.31E-12 | 6.60E-13 | 2.49E-13 |
|  | ABHD12 KO | 15 | 9.25E-13 | 2.27E-13 | 5.87E-14 | 13 | 2.17E-12 | 5.46E-13 | 1.51E-13 |
|  | Young | 12 | 7.96E-13 | 1.17E-13 | 3.37E-14 | 12 | 2.19E-12 | 5.11E-13 | 1.48E-13 |
|  | Old | 15 | 9.27E-13 | 2.55E-13 | 6.58E-14 | 12 | 2.34E-12 | 6.60E-13 | 1.91E-13 |
| *N*-stearoyl proline | WT young | 6 | 4.36E-13 | 7.95E-14 | 3.24E-14 | 6 | 8.96E-13 | 2.26E-13 | 9.24E-14 |
|  | WT old | 6 | 4.51E-13 | 8.25E-14 | 3.37E-14 | 5 | 8.30E-13 | 2.69E-13 | 1.20E-13 |
|  | WT | 12 | 4.44E-13 | 7.76E-14 | 2.24E-14 | 11 | 8.66E-13 | 2.36E-13 | 7.11E-14 |
|  | KO young | 6 | 5.48E-13 | 6.97E-14 | 2.84E-14 | 6 | 9.45E-13 | 1.75E-13 | 7.13E-14 |
|  | KO old | 9 | 5.47E-13 | 1.31E-13 | 4.35E-14 | 7 | 6.91E-13 | 1.26E-13 | 4.77E-14 |
|  | ABHD12 KO | 15 | 5.47E-13 | 1.07E-13 | 2.77E-14 | 13 | 8.08E-13 | 1.95E-13 | 5.41E-14 |
|  | Young | 12 | 4.92E-13 | 9.22E-14 | 2.66E-14 | 12 | 9.20E-13 | 1.95E-13 | 5.62E-14 |
|  | Old | 15 | 5.09E-13 | 1.20E-13 | 3.11E-14 | 12 | 7.49E-13 | 2.00E-13 | 5.78E-14 |
| *N*-oleoyl proline | WT young | 6 | 3.53E-13 | 8.06E-14 | 3.29E-14 | 6 | PISSR |  |  |
|  | WT old | 6 | 3.69E-13 | 9.63E-14 | 3.93E-14 | 5 | PISSR |  |  |
|  | WT | 12 | 3.61E-13 | 8.51E-14 | 2.46E-14 | 11 | PISSR |  |  |
|  | KO young | 6 | 2.93E-13 | 7.50E-14 | 3.06E-14 | 6 | PISSR |  |  |
|  | KO old | 9 | 3.73E-13 | 9.84E-14 | 3.28E-14 | 7 | PISSR |  |  |
|  | ABHD12 KO | 15 | 3.41E-13 | 9.57E-14 | 2.47E-14 | 13 | PISSR |  |  |
|  | Young | 12 | 3.23E-13 | 8.04E-14 | 2.32E-14 | 12 | PISSR |  |  |
|  | Old | 15 | 3.71E-13 | 9.41E-14 | 2.43E-14 | 12 | PISSR |  |  |
| *N*-linoleoyl proline | WT young | 6 | PISSR |  |  | 6 | BDL |  |  |
|  | WT old | 6 | PISSR |  |  | 5 | BDL |  |  |
|  | WT | 12 | PISSR |  |  | 11 | BDL |  |  |
|  | KO young | 6 | PISSR |  |  | 6 | BDL |  |  |
|  | KO old | 9 | PISSR |  |  | 7 | BDL |  |  |
|  | ABHD12 KO | 15 | PISSR |  |  | 13 | BDL |  |  |
|  | Young | 12 | PISSR |  |  | 12 | BDL |  |  |
|  | Old | 15 | PISSR |  |  | 12 | BDL |  |  |
| *N*-arachidonoyl proline | WT young | 6 | BDL |  |  | 6 | PISSR |  |  |
|  | WT old | 6 | BDL |  |  | 5 | PISSR |  |  |
|  | WT | 12 | BDL |  |  | 11 | PISSR |  |  |
|  | KO young | 6 | BDL |  |  | 6 | PISSR |  |  |
|  | KO old | 9 | BDL |  |  | 7 | PISSR |  |  |
|  | ABHD12 KO | 15 | BDL |  |  | 13 | PISSR |  |  |
|  | Young | 12 | BDL |  |  | 12 | PISSR |  |  |
|  | Old | 15 | BDL |  |  | 12 | PISSR |  |  |
| *N*-docosahexaenoyl proline | WT young | 6 | BDL |  |  | 6 | BDL |  |  |
|  | WT old | 6 | BDL |  |  | 5 | BDL |  |  |
|  | WT | 12 | BDL |  |  | 11 | BDL |  |  |
|  | KO young | 6 | BDL |  |  | 6 | BDL |  |  |
|  | KO old | 9 | BDL |  |  | 7 | BDL |  |  |
|  | ABHD12 KO | 15 | BDL |  |  | 13 | BDL |  |  |
|  | Young | 12 | BDL |  |  | 12 | BDL |  |  |
|  | Old | 15 | BDL |  |  | 12 | BDL |  |  |

Supplemental Table 33: Mean levels of *N*-acyl prolines in the midbrain and brainstem

| Lipid Species | Group | Midbrain | | | | Brainstem | | | |
| --- | --- | --- | --- | --- | --- | --- | --- | --- | --- |
|  |  | N | Mean | SD | SE | N | Mean | SD | SE |
| *N*-palmitoyl proline | WT young | 6 | 1.04E-12 | 3.19E-13 | 1.30E-13 | 6 | 1.54E-12 | 3.64E-13 | 1.49E-13 |
|  | WT old | 6 | 1.04E-12 | 2.24E-13 | 9.14E-14 | 6 | 1.52E-12 | 9.49E-14 | 3.87E-14 |
|  | WT | 12 | 1.04E-12 | 2.63E-13 | 7.59E-14 | 12 | 1.53E-12 | 2.54E-13 | 7.32E-14 |
|  | KO young | 6 | 1.26E-12 | 1.44E-13 | 5.90E-14 | 6 | 1.69E-12 | 3.36E-13 | 1.37E-13 |
|  | KO old | 9 | 1.06E-12 | 2.97E-13 | 9.90E-14 | 9 | 1.51E-12 | 2.57E-13 | 8.57E-14 |
|  | ABHD12 KO | 15 | 1.14E-12 | 2.61E-13 | 6.73E-14 | 15 | 1.58E-12 | 2.95E-13 | 7.61E-14 |
|  | Young | 12 | 1.15E-12 | 2.62E-13 | 7.58E-14 | 12 | 1.62E-12 | 3.44E-13 | 9.92E-14 |
|  | Old | 15 | 1.05E-12 | 2.62E-13 | 6.76E-14 | 15 | 1.51E-12 | 2.02E-13 | 5.23E-14 |
| *N*-stearoyl proline | WT young | 6 | 5.15E-13 | 9.80E-14 | 4.00E-14 | 6 | 8.12E-13 | 1.17E-13 | 4.76E-14 |
|  | WT old | 6 | 5.51E-13 | 8.98E-14 | 3.67E-14 | 6 | 7.99E-13 | 8.65E-14 | 3.53E-14 |
|  | WT | 12 | 5.33E-13 | 9.16E-14 | 2.64E-14 | 12 | 8.05E-13 | 9.81E-14 | 2.83E-14 |
|  | KO young | 6 | 5.77E-13 | 1.47E-13 | 6.00E-14 | 6 | 9.02E-13 | 1.78E-13 | 7.29E-14 |
|  | KO old | 9 | 5.69E-13 | 9.70E-14 | 3.23E-14 | 9 | 8.13E-13 | 1.15E-13 | 3.84E-14 |
|  | ABHD12 KO | 15 | 5.72E-13 | 1.15E-13 | 2.96E-14 | 15 | 8.49E-13 | 1.45E-13 | 3.74E-14 |
|  | Young | 12 | 5.46E-13 | 1.23E-13 | 3.57E-14 | 12 | 8.57E-13 | 1.51E-13 | 4.37E-14 |
|  | Old | 15 | 5.62E-13 | 9.13E-14 | 2.36E-14 | 15 | 8.07E-13 | 1.02E-13 | 2.62E-14 |
| *N*-oleoyl proline | WT young | 6 | 3.82E-13 | 7.45E-14 | 3.04E-14 | 6 | 6.84E-13 | 1.89E-13 | 7.73E-14 |
|  | WT old | 6 | 3.71E-13 | 1.42E-13 | 5.81E-14 | 6 | 6.99E-13 | 8.86E-14 | 3.62E-14 |
|  | WT | 12 | 3.77E-13 | 1.08E-13 | 3.13E-14 | 12 | 6.91E-13 | 1.41E-13 | 4.07E-14 |
|  | KO young | 6 | 2.86E-13 | 7.11E-14 | 2.90E-14 | 6 | 8.63E-13 | 2.96E-13 | 1.21E-13 |
|  | KO old | 9 | 4.23E-13 | 1.52E-13 | 5.06E-14 | 9 | 7.07E-13 | 1.57E-13 | 5.24E-14 |
|  | ABHD12 KO | 15 | 3.68E-13 | 1.41E-13 | 3.63E-14 | 15 | 7.69E-13 | 2.27E-13 | 5.87E-14 |
|  | Young | 12 | 3.34E-13 | 8.57E-14 | 2.47E-14 | 12 | 7.73E-13 | 2.55E-13 | 7.35E-14 |
|  | Old | 15 | 4.02E-13 | 1.45E-13 | 3.75E-14 | 15 | 7.04E-13 | 1.30E-13 | 3.36E-14 |
| *N*-linoleoyl proline | WT young | 6 | BDL |  |  | 6 | PISSR |  |  |
|  | WT old | 6 | BDL |  |  | 6 | PISSR |  |  |
|  | WT | 12 | BDL |  |  | 12 | PISSR |  |  |
|  | KO young | 6 | BDL |  |  | 6 | PISSR |  |  |
|  | KO old | 9 | BDL |  |  | 9 | PISSR |  |  |
|  | ABHD12 KO | 15 | BDL |  |  | 15 | PISSR |  |  |
|  | Young | 12 | BDL |  |  | 12 | PISSR |  |  |
|  | Old | 15 | BDL |  |  | 15 | PISSR |  |  |
| *N*-arachidonoyl proline | WT young | 6 | BDL |  |  | 6 | PISSR |  |  |
|  | WT old | 6 | BDL |  |  | 6 | PISSR |  |  |
|  | WT | 12 | BDL |  |  | 12 | PISSR |  |  |
|  | KO young | 6 | BDL |  |  | 6 | PISSR |  |  |
|  | KO old | 9 | BDL |  |  | 9 | PISSR |  |  |
|  | ABHD12 KO | 15 | BDL |  |  | 15 | PISSR |  |  |
|  | Young | 12 | BDL |  |  | 12 | PISSR |  |  |
|  | Old | 15 | BDL |  |  | 15 | PISSR |  |  |
| *N*-docosahexaenoyl proline | WT young | 6 | BDL |  |  | 6 | PISSR |  |  |
|  | WT old | 6 | BDL |  |  | 6 | PISSR |  |  |
|  | WT | 12 | BDL |  |  | 12 | PISSR |  |  |
|  | KO young | 6 | BDL |  |  | 6 | PISSR |  |  |
|  | KO old | 9 | BDL |  |  | 9 | PISSR |  |  |
|  | ABHD12 KO | 15 | BDL |  |  | 15 | PISSR |  |  |
|  | Young | 12 | BDL |  |  | 12 | PISSR |  |  |
|  | Old | 15 | BDL |  |  | 15 | PISSR |  |  |

Supplemental Table 34: Mean levels of *N*-acyl serines in the striatum and hippocampus

| Lipid Species | Group | Striatum | | | | Hippocampus | | | |
| --- | --- | --- | --- | --- | --- | --- | --- | --- | --- |
|  |  | N | Mean | SD | SE | N | Mean | SD | SE |
| *N*-palmitoyl serine | WT young | 6 | 1.33E-10 | 1.66E-11 | 6.76E-12 | 6 | 6.69E-11 | 5.82E-12 | 2.37E-12 |
|  | WT old | 6 | 1.30E-10 | 1.96E-11 | 7.99E-12 | 6 | 7.01E-11 | 5.76E-12 | 2.35E-12 |
|  | WT | 12 | 1.32E-10 | 1.74E-11 | 5.02E-12 | 12 | 6.85E-11 | 5.77E-12 | 1.66E-12 |
|  | KO young | 6 | 1.05E-10 | 1.10E-11 | 4.50E-12 | 6 | 5.51E-11 | 3.01E-12 | 1.23E-12 |
|  | KO old | 9 | 1.20E-10 | 1.62E-11 | 5.39E-12 | 9 | 6.59E-11 | 6.45E-12 | 2.15E-12 |
|  | ABHD12 KO | 15 | 1.14E-10 | 1.60E-11 | 4.13E-12 | 15 | 6.16E-11 | 7.54E-12 | 1.95E-12 |
|  | Young | 12 | 1.19E-10 | 2.01E-11 | 5.80E-12 | 12 | 6.10E-11 | 7.56E-12 | 2.18E-12 |
|  | Old | 15 | 1.24E-10 | 1.76E-11 | 4.54E-12 | 15 | 6.76E-11 | 6.33E-12 | 1.64E-12 |
| *N*-stearoyl serine | WT young | 6 | 6.74E-11 | 3.82E-12 | 1.56E-12 | 6 | 3.59E-11 | 3.58E-12 | 1.46E-12 |
|  | WT old | 6 | 6.81E-11 | 1.10E-11 | 4.50E-12 | 6 | 3.99E-11 | 4.22E-12 | 1.72E-12 |
|  | WT | 12 | 6.78E-11 | 7.88E-12 | 2.27E-12 | 12 | 3.79E-11 | 4.25E-12 | 1.23E-12 |
|  | KO young | 6 | 6.27E-11 | 6.18E-12 | 2.52E-12 | 6 | 3.48E-11 | 5.78E-12 | 2.36E-12 |
|  | KO old | 9 | 6.90E-11 | 8.13E-12 | 2.71E-12 | 9 | 3.72E-11 | 6.28E-12 | 2.09E-12 |
|  | ABHD12 KO | 15 | 6.65E-11 | 7.86E-12 | 2.03E-12 | 15 | 3.62E-11 | 5.99E-12 | 1.55E-12 |
|  | Young | 12 | 6.50E-11 | 5.49E-12 | 1.58E-12 | 12 | 3.54E-11 | 4.62E-12 | 1.33E-12 |
|  | Old | 15 | 6.86E-11 | 9.02E-12 | 2.33E-12 | 15 | 3.83E-11 | 5.54E-12 | 1.43E-12 |
| *N*-oleoyl serine | WT young | 6 | 8.60E-11 | 1.29E-11 | 5.28E-12 | 6 | 4.39E-11 | 5.75E-12 | 2.35E-12 |
|  | WT old | 6 | 8.07E-11 | 1.07E-11 | 4.36E-12 | 6 | 4.77E-11 | 2.64E-12 | 1.08E-12 |
|  | WT | 12 | 8.34E-11 | 1.16E-11 | 3.36E-12 | 12 | 4.58E-11 | 4.71E-12 | 1.36E-12 |
|  | KO young | 6 | 7.31E-11 | 1.10E-11 | 4.49E-12 | 6 | 3.99E-11 | 3.33E-12 | 1.36E-12 |
|  | KO old | 9 | 7.77E-11 | 1.10E-11 | 3.68E-12 | 9 | 4.65E-11 | 3.85E-12 | 1.28E-12 |
|  | ABHD12 KO | 15 | 7.59E-11 | 1.09E-11 | 2.81E-12 | 15 | 4.39E-11 | 4.88E-12 | 1.26E-12 |
|  | Young | 12 | 7.96E-11 | 1.33E-11 | 3.83E-12 | 12 | 4.19E-11 | 4.94E-12 | 1.43E-12 |
|  | Old | 15 | 7.89E-11 | 1.06E-11 | 2.74E-12 | 15 | 4.70E-11 | 3.36E-12 | 8.68E-13 |
| *N*-linoleoyl serine | WT young | 6 | 7.94E-12 | 1.10E-12 | 4.50E-13 | 6 | 5.03E-12 | 1.32E-12 | 5.40E-13 |
|  | WT old | 6 | 7.99E-12 | 1.27E-12 | 5.18E-13 | 6 | 3.17E-12 | 1.60E-12 | 6.55E-13 |
|  | WT | 12 | 7.97E-12 | 1.13E-12 | 3.27E-13 | 12 | 4.10E-12 | 1.70E-12 | 4.92E-13 |
|  | KO young | 6 | 7.97E-12 | 1.89E-12 | 7.70E-13 | 6 | 6.00E-12 | 1.42E-12 | 5.78E-13 |
|  | KO old | 9 | 7.56E-12 | 2.45E-12 | 8.16E-13 | 9 | 4.30E-12 | 1.19E-12 | 3.96E-13 |
|  | ABHD12 KO | 15 | 7.73E-12 | 2.18E-12 | 5.62E-13 | 15 | 4.98E-12 | 1.51E-12 | 3.89E-13 |
|  | Young | 12 | 7.96E-12 | 1.47E-12 | 4.25E-13 | 12 | 5.51E-12 | 1.40E-12 | 4.05E-13 |
|  | Old | 15 | 7.74E-12 | 2.01E-12 | 5.20E-13 | 15 | 3.85E-12 | 1.43E-12 | 3.70E-13 |
| *N*-arachidonoyl serine | WT young | 6 | 6.99E-12 | 6.05E-13 | 2.47E-13 | 6 | 4.82E-12 | 1.12E-12 | 4.55E-13 |
|  | WT old | 6 | 6.60E-12 | 1.19E-12 | 4.86E-13 | 6 | 4.16E-12 | 9.90E-13 | 4.04E-13 |
|  | WT | 12 | 6.79E-12 | 9.24E-13 | 2.67E-13 | 12 | 4.49E-12 | 1.06E-12 | 3.07E-13 |
|  | KO young | 6 | 8.54E-12 | 6.22E-13 | 2.54E-13 | 6 | 7.28E-12 | 1.18E-12 | 4.80E-13 |
|  | KO old | 9 | 9.20E-12 | 1.24E-12 | 4.14E-13 | 9 | 6.30E-12 | 1.22E-12 | 4.05E-13 |
|  | ABHD12 KO | 15 | 8.94E-12 | 1.06E-12 | 2.75E-13 | 15 | 6.70E-12 | 1.26E-12 | 3.25E-13 |
|  | Young | 12 | 7.76E-12 | 9.97E-13 | 2.88E-13 | 12 | 6.05E-12 | 1.69E-12 | 4.88E-13 |
|  | Old | 15 | 8.16E-12 | 1.77E-12 | 4.57E-13 | 15 | 5.44E-12 | 1.54E-12 | 3.98E-13 |
| *N*-docosahexaenoyl serine | WT young | 6 | 5.43E-11 | 1.04E-11 | 4.25E-12 | 6 | 2.17E-11 | 3.63E-12 | 1.48E-12 |
|  | WT old | 6 | 4.62E-11 | 2.14E-12 | 8.75E-13 | 6 | 1.93E-11 | 3.80E-12 | 1.55E-12 |
|  | WT | 12 | 5.02E-11 | 8.32E-12 | 2.40E-12 | 12 | 2.05E-11 | 3.75E-12 | 1.08E-12 |
|  | KO young | 6 | 4.64E-11 | 9.26E-12 | 3.78E-12 | 6 | 2.21E-11 | 4.68E-12 | 1.91E-12 |
|  | KO old | 9 | 4.81E-11 | 7.99E-12 | 2.66E-12 | 9 | 2.18E-11 | 3.44E-12 | 1.15E-12 |
|  | ABHD12 KO | 15 | 4.74E-11 | 8.24E-12 | 2.13E-12 | 15 | 2.19E-11 | 3.82E-12 | 9.87E-13 |
|  | Young | 12 | 5.04E-11 | 1.03E-11 | 2.96E-12 | 12 | 2.19E-11 | 4.00E-12 | 1.15E-12 |
|  | Old | 15 | 4.74E-11 | 6.25E-12 | 1.61E-12 | 15 | 2.08E-11 | 3.67E-12 | 9.48E-13 |

Supplemental Table 35: Mean levels of *N*-acyl serines in the cerebellum and thalamus

| Lipid Species | Group | Cerebellum | | | | Thalamus | | | |
| --- | --- | --- | --- | --- | --- | --- | --- | --- | --- |
|  |  | N | Mean | SD | SE | N | Mean | SD | SE |
| *N*-palmitoyl serine | WT young | 6 | 9.72E-11 | 7.92E-12 | 3.23E-12 | 6 | 1.06E-10 | 1.55E-11 | 6.34E-12 |
|  | WT old | 6 | 1.06E-10 | 5.46E-12 | 2.23E-12 | 6 | 1.07E-10 | 3.10E-11 | 1.27E-11 |
|  | WT | 12 | 1.01E-10 | 7.81E-12 | 2.26E-12 | 12 | 1.06E-10 | 2.34E-11 | 6.75E-12 |
|  | KO young | 6 | 8.59E-11 | 3.81E-12 | 1.56E-12 | 6 | 1.02E-10 | 1.48E-11 | 6.05E-12 |
|  | KO old | 9 | 9.37E-11 | 6.05E-12 | 2.02E-12 | 9 | 1.14E-10 | 1.69E-11 | 5.64E-12 |
|  | ABHD12 KO | 15 | 9.06E-11 | 6.47E-12 | 1.67E-12 | 15 | 1.09E-10 | 1.68E-11 | 4.33E-12 |
|  | Young | 12 | 9.15E-11 | 8.36E-12 | 2.41E-12 | 12 | 1.04E-10 | 1.46E-11 | 4.22E-12 |
|  | Old | 15 | 9.84E-11 | 8.21E-12 | 2.12E-12 | 15 | 1.11E-10 | 2.28E-11 | 5.90E-12 |
| *N*-stearoyl serine | WT young | 6 | 7.60E-11 | 3.65E-12 | 1.49E-12 | 6 | 6.83E-11 | 9.23E-12 | 3.77E-12 |
|  | WT old | 6 | 8.94E-11 | 7.22E-12 | 2.95E-12 | 6 | 7.10E-11 | 5.76E-12 | 2.35E-12 |
|  | WT | 12 | 8.27E-11 | 8.88E-12 | 2.56E-12 | 12 | 6.96E-11 | 7.47E-12 | 2.16E-12 |
|  | KO young | 6 | 7.57E-11 | 6.99E-12 | 2.85E-12 | 6 | 7.39E-11 | 6.38E-12 | 2.60E-12 |
|  | KO old | 9 | 8.49E-11 | 7.01E-12 | 2.34E-12 | 9 | 7.28E-11 | 7.32E-12 | 2.44E-12 |
|  | ABHD12 KO | 15 | 8.12E-11 | 8.22E-12 | 2.12E-12 | 15 | 7.32E-11 | 6.74E-12 | 1.74E-12 |
|  | Young | 12 | 7.58E-11 | 5.32E-12 | 1.54E-12 | 12 | 7.11E-11 | 8.10E-12 | 2.34E-12 |
|  | Old | 15 | 8.67E-11 | 7.20E-12 | 1.86E-12 | 15 | 7.21E-11 | 6.58E-12 | 1.70E-12 |
| *N*-oleoyl serine | WT young | 6 | 8.03E-11 | 6.48E-12 | 2.65E-12 | 6 | 8.72E-11 | 6.27E-12 | 2.56E-12 |
|  | WT old | 6 | 8.45E-11 | 6.23E-12 | 2.54E-12 | 6 | 9.07E-11 | 1.25E-11 | 5.12E-12 |
|  | WT | 12 | 8.24E-11 | 6.45E-12 | 1.86E-12 | 12 | 8.89E-11 | 9.62E-12 | 2.78E-12 |
|  | KO young | 6 | 6.93E-11 | 5.57E-12 | 2.28E-12 | 6 | 8.36E-11 | 8.45E-12 | 3.45E-12 |
|  | KO old | 9 | 7.99E-11 | 7.16E-12 | 2.39E-12 | 9 | 9.58E-11 | 9.72E-12 | 3.24E-12 |
|  | ABHD12 KO | 15 | 7.56E-11 | 8.30E-12 | 2.14E-12 | 15 | 9.09E-11 | 1.09E-11 | 2.81E-12 |
|  | Young | 12 | 7.48E-11 | 8.12E-12 | 2.34E-12 | 12 | 8.54E-11 | 7.35E-12 | 2.12E-12 |
|  | Old | 15 | 8.17E-11 | 6.97E-12 | 1.80E-12 | 15 | 9.38E-11 | 1.08E-11 | 2.79E-12 |
| *N*-linoleoyl serine | WT young | 6 | 1.40E-11 | 3.01E-12 | 1.23E-12 | 6 | 5.37E-12 | 8.04E-13 | 3.28E-13 |
|  | WT old | 6 | 1.34E-11 | 1.90E-12 | 7.76E-13 | 6 | 4.94E-12 | 2.24E-12 | 9.13E-13 |
|  | WT | 12 | 1.37E-11 | 2.42E-12 | 7.00E-13 | 12 | 5.15E-12 | 1.62E-12 | 4.67E-13 |
|  | KO young | 6 | 1.50E-11 | 1.29E-12 | 5.25E-13 | 6 | 5.37E-12 | 8.93E-13 | 3.65E-13 |
|  | KO old | 9 | 1.38E-11 | 2.62E-12 | 8.74E-13 | 9 | 5.38E-12 | 1.54E-12 | 5.13E-13 |
|  | ABHD12 KO | 15 | 1.42E-11 | 2.21E-12 | 5.71E-13 | 15 | 5.37E-12 | 1.28E-12 | 3.31E-13 |
|  | Young | 12 | 1.45E-11 | 2.26E-12 | 6.53E-13 | 12 | 5.37E-12 | 8.10E-13 | 2.34E-13 |
|  | Old | 15 | 1.36E-11 | 2.29E-12 | 5.92E-13 | 15 | 5.20E-12 | 1.79E-12 | 4.61E-13 |
| *N*-arachidonoyl serine | WT young | 6 | 1.03E-11 | 1.01E-12 | 4.12E-13 | 6 | 3.29E-12 | 9.38E-13 | 3.83E-13 |
|  | WT old | 6 | 8.90E-12 | 9.56E-13 | 3.90E-13 | 6 | 3.43E-12 | 1.44E-12 | 5.88E-13 |
|  | WT | 12 | 9.61E-12 | 1.20E-12 | 3.46E-13 | 12 | 3.36E-12 | 1.16E-12 | 3.35E-13 |
|  | KO young | 6 | 1.34E-11 | 1.40E-12 | 5.72E-13 | 6 | 6.19E-12 | 1.48E-12 | 6.03E-13 |
|  | KO old | 9 | 1.28E-11 | 2.13E-12 | 7.09E-13 | 9 | 6.39E-12 | 1.02E-12 | 3.41E-13 |
|  | ABHD12 KO | 15 | 1.30E-11 | 1.84E-12 | 4.74E-13 | 15 | 6.31E-12 | 1.18E-12 | 3.04E-13 |
|  | Young | 12 | 1.18E-11 | 1.96E-12 | 5.67E-13 | 12 | 4.74E-12 | 1.92E-12 | 5.54E-13 |
|  | Old | 15 | 1.12E-11 | 2.61E-12 | 6.75E-13 | 15 | 5.21E-12 | 1.90E-12 | 4.90E-13 |
| *N*-docosahexaenoyl serine | WT young | 6 | 5.31E-11 | 6.29E-12 | 2.57E-12 | 6 | 1.61E-11 | 3.46E-12 | 1.41E-12 |
|  | WT old | 6 | 5.32E-11 | 3.87E-12 | 1.58E-12 | 6 | 1.69E-11 | 4.84E-12 | 1.98E-12 |
|  | WT | 12 | 5.31E-11 | 4.98E-12 | 1.44E-12 | 12 | 1.65E-11 | 4.04E-12 | 1.17E-12 |
|  | KO young | 6 | 5.19E-11 | 6.95E-12 | 2.84E-12 | 6 | 2.52E-11 | 6.59E-12 | 2.69E-12 |
|  | KO old | 9 | 5.25E-11 | 4.39E-12 | 1.46E-12 | 9 | 2.27E-11 | 3.96E-12 | 1.32E-12 |
|  | ABHD12 KO | 15 | 5.23E-11 | 5.32E-12 | 1.37E-12 | 15 | 2.37E-11 | 5.10E-12 | 1.32E-12 |
|  | Young | 12 | 5.25E-11 | 6.35E-12 | 1.83E-12 | 12 | 2.06E-11 | 6.90E-12 | 1.99E-12 |
|  | Old | 15 | 5.28E-11 | 4.06E-12 | 1.05E-12 | 15 | 2.04E-11 | 5.09E-12 | 1.31E-12 |

Supplemental Table 36: Mean levels of *N*-acyl serines in the cortex and hypothalamus

| Lipid Species | Group | Cortex | | | | Hypothalamus | | | |
| --- | --- | --- | --- | --- | --- | --- | --- | --- | --- |
|  |  | N | Mean | SD | SE | N | Mean | SD | SE |
| *N*-palmitoyl serine | WT young | 6 | 6.93E-11 | 5.11E-12 | 2.09E-12 | 6 | 3.02E-11 | 5.07E-12 | 2.07E-12 |
|  | WT old | 6 | 6.63E-11 | 5.30E-12 | 2.16E-12 | 5 | 2.88E-11 | 6.25E-12 | 2.80E-12 |
|  | WT | 12 | 6.78E-11 | 5.20E-12 | 1.50E-12 | 11 | 2.96E-11 | 5.39E-12 | 1.62E-12 |
|  | KO young | 6 | 6.78E-11 | 7.78E-12 | 3.17E-12 | 6 | 2.34E-11 | 2.84E-12 | 1.16E-12 |
|  | KO old | 9 | 6.40E-11 | 6.85E-12 | 2.28E-12 | 7 | 2.25E-11 | 4.53E-12 | 1.71E-12 |
|  | ABHD12 KO | 15 | 6.55E-11 | 7.22E-12 | 1.86E-12 | 13 | 2.29E-11 | 3.72E-12 | 1.03E-12 |
|  | Young | 12 | 6.85E-11 | 6.32E-12 | 1.83E-12 | 12 | 2.68E-11 | 5.29E-12 | 1.53E-12 |
|  | Old | 15 | 6.49E-11 | 6.19E-12 | 1.60E-12 | 12 | 2.52E-11 | 5.99E-12 | 1.73E-12 |
| *N*-stearoyl serine | WT young | 6 | 4.50E-11 | 2.72E-12 | 1.11E-12 | 6 | 2.76E-11 | 2.81E-12 | 1.15E-12 |
|  | WT old | 6 | 4.43E-11 | 3.22E-12 | 1.32E-12 | 5 | 2.77E-11 | 6.78E-12 | 3.03E-12 |
|  | WT | 12 | 4.46E-11 | 2.87E-12 | 8.27E-13 | 11 | 2.77E-11 | 4.72E-12 | 1.42E-12 |
|  | KO young | 6 | 4.33E-11 | 3.41E-12 | 1.39E-12 | 6 | 2.45E-11 | 2.63E-12 | 1.08E-12 |
|  | KO old | 9 | 4.63E-11 | 4.87E-12 | 1.62E-12 | 7 | 2.62E-11 | 5.46E-12 | 2.06E-12 |
|  | ABHD12 KO | 15 | 4.51E-11 | 4.47E-12 | 1.15E-12 | 13 | 2.54E-11 | 4.30E-12 | 1.19E-12 |
|  | Young | 12 | 4.42E-11 | 3.07E-12 | 8.85E-13 | 12 | 2.61E-11 | 3.06E-12 | 8.82E-13 |
|  | Old | 15 | 4.55E-11 | 4.28E-12 | 1.10E-12 | 12 | 2.68E-11 | 5.80E-12 | 1.67E-12 |
| *N*-oleoyl serine | WT young | 6 | 4.48E-11 | 3.19E-12 | 1.30E-12 | 6 | 1.87E-11 | 1.90E-12 | 7.78E-13 |
|  | WT old | 6 | 4.27E-11 | 2.59E-12 | 1.06E-12 | 5 | 1.97E-11 | 4.32E-12 | 1.93E-12 |
|  | WT | 12 | 4.38E-11 | 2.97E-12 | 8.58E-13 | 11 | 1.92E-11 | 3.10E-12 | 9.34E-13 |
|  | KO young | 6 | 4.31E-11 | 4.17E-12 | 1.70E-12 | 6 | 1.65E-11 | 1.92E-12 | 7.86E-13 |
|  | KO old | 9 | 4.49E-11 | 4.69E-12 | 1.56E-12 | 7 | 1.58E-11 | 3.18E-12 | 1.20E-12 |
|  | ABHD12 KO | 15 | 4.42E-11 | 4.44E-12 | 1.15E-12 | 13 | 1.61E-11 | 2.59E-12 | 7.20E-13 |
|  | Young | 12 | 4.39E-11 | 3.66E-12 | 1.06E-12 | 12 | 1.76E-11 | 2.15E-12 | 6.21E-13 |
|  | Old | 15 | 4.41E-11 | 4.02E-12 | 1.04E-12 | 12 | 1.75E-11 | 4.04E-12 | 1.17E-12 |
| *N*-linoleoyl serine | WT young | 6 | 6.85E-12 | 7.44E-13 | 3.04E-13 | 6 | PISSR |  |  |
|  | WT old | 6 | 4.76E-12 | 9.96E-13 | 4.07E-13 | 5 | PISSR |  |  |
|  | WT | 12 | 5.80E-12 | 1.37E-12 | 3.97E-13 | 11 | PISSR |  |  |
|  | KO young | 6 | 6.25E-12 | 8.71E-13 | 3.56E-13 | 6 | PISSR |  |  |
|  | KO old | 9 | 4.52E-12 | 9.06E-13 | 3.02E-13 | 7 | PISSR |  |  |
|  | ABHD12 KO | 15 | 5.21E-12 | 1.23E-12 | 3.17E-13 | 13 | PISSR |  |  |
|  | Young | 12 | 6.55E-12 | 8.32E-13 | 2.40E-13 | 12 | PISSR |  |  |
|  | Old | 15 | 4.62E-12 | 9.15E-13 | 2.36E-13 | 12 | PISSR |  |  |
| *N*-arachidonoyl serine | WT young | 6 | 4.48E-12 | 8.66E-13 | 3.53E-13 | 6 | 1.69E-12 | 3.41E-13 | 1.39E-13 |
|  | WT old | 6 | 3.34E-12 | 5.72E-13 | 2.34E-13 | 5 | 1.44E-12 | 3.91E-13 | 1.75E-13 |
|  | WT | 12 | 3.91E-12 | 9.17E-13 | 2.65E-13 | 11 | 1.58E-12 | 3.71E-13 | 1.12E-13 |
|  | KO young | 6 | 6.52E-12 | 1.02E-12 | 4.15E-13 | 6 | 1.97E-12 | 1.04E-12 | 4.23E-13 |
|  | KO old | 9 | 5.82E-12 | 5.75E-13 | 1.92E-13 | 7 | 1.72E-12 | 8.16E-13 | 3.08E-13 |
|  | ABHD12 KO | 15 | 6.10E-12 | 8.28E-13 | 2.14E-13 | 13 | 1.83E-12 | 8.93E-13 | 2.48E-13 |
|  | Young | 12 | 5.50E-12 | 1.40E-12 | 4.03E-13 | 12 | 1.83E-12 | 7.50E-13 | 2.16E-13 |
|  | Old | 15 | 4.83E-12 | 1.37E-12 | 3.54E-13 | 12 | 1.60E-12 | 6.63E-13 | 1.91E-13 |
| *N*-docosahexaenoyl serine | WT young | 6 | 2.44E-11 | 3.65E-12 | 1.49E-12 | 6 | 1.26E-11 | 2.07E-12 | 8.44E-13 |
|  | WT old | 6 | 2.32E-11 | 3.06E-12 | 1.25E-12 | 5 | 8.69E-12 | 2.58E-12 | 1.15E-12 |
|  | WT | 12 | 2.38E-11 | 3.27E-12 | 9.44E-13 | 11 | 1.08E-11 | 3.00E-12 | 9.04E-13 |
|  | KO young | 6 | 3.21E-11 | 6.60E-12 | 2.69E-12 | 6 | 1.20E-11 | 6.00E-12 | 2.45E-12 |
|  | KO old | 9 | 2.65E-11 | 3.55E-12 | 1.18E-12 | 7 | 8.20E-12 | 4.02E-12 | 1.52E-12 |
|  | ABHD12 KO | 15 | 2.87E-11 | 5.57E-12 | 1.44E-12 | 13 | 9.95E-12 | 5.19E-12 | 1.44E-12 |
|  | Young | 12 | 2.83E-11 | 6.50E-12 | 1.88E-12 | 12 | 1.23E-11 | 4.29E-12 | 1.24E-12 |
|  | Old | 15 | 2.52E-11 | 3.65E-12 | 9.42E-13 | 12 | 8.40E-12 | 3.36E-12 | 9.71E-13 |

Supplemental Table 37: Mean levels of *N*-acyl serines in the midbrain and brainstem

| Lipid Species | Group | Midbrain | | | | Brainstem | | | |
| --- | --- | --- | --- | --- | --- | --- | --- | --- | --- |
|  |  | N | Mean | SD | SE | N | Mean | SD | SE |
| *N*-palmitoyl serine | WT young | 6 | 9.32E-11 | 1.83E-11 | 7.48E-12 | 6 | 9.65E-11 | 5.27E-12 | 2.15E-12 |
|  | WT old | 6 | 1.19E-10 | 1.29E-11 | 5.27E-12 | 6 | 1.09E-10 | 4.74E-12 | 1.94E-12 |
|  | WT | 12 | 1.06E-10 | 2.02E-11 | 5.83E-12 | 12 | 1.03E-10 | 7.91E-12 | 2.28E-12 |
|  | KO young | 6 | 8.91E-11 | 2.59E-12 | 1.06E-12 | 6 | 8.57E-11 | 4.76E-12 | 1.94E-12 |
|  | KO old | 9 | 1.04E-10 | 9.18E-12 | 3.06E-12 | 9 | 9.72E-11 | 3.98E-12 | 1.33E-12 |
|  | ABHD12 KO | 15 | 9.80E-11 | 1.04E-11 | 2.67E-12 | 15 | 9.26E-11 | 7.15E-12 | 1.84E-12 |
|  | Young | 12 | 9.11E-11 | 1.27E-11 | 3.66E-12 | 12 | 9.11E-11 | 7.40E-12 | 2.14E-12 |
|  | Old | 15 | 1.10E-10 | 1.28E-11 | 3.31E-12 | 15 | 1.02E-10 | 7.10E-12 | 1.83E-12 |
| *N*-stearoyl serine | WT young | 6 | 5.09E-11 | 4.57E-12 | 1.87E-12 | 6 | 6.21E-11 | 4.88E-12 | 1.99E-12 |
|  | WT old | 6 | 5.79E-11 | 7.48E-12 | 3.05E-12 | 6 | 6.79E-11 | 3.07E-12 | 1.25E-12 |
|  | WT | 12 | 5.44E-11 | 6.95E-12 | 2.01E-12 | 12 | 6.50E-11 | 4.95E-12 | 1.43E-12 |
|  | KO young | 6 | 5.05E-11 | 4.30E-12 | 1.75E-12 | 6 | 5.95E-11 | 5.04E-12 | 2.06E-12 |
|  | KO old | 9 | 6.17E-11 | 3.22E-12 | 1.07E-12 | 9 | 6.76E-11 | 4.94E-12 | 1.65E-12 |
|  | ABHD12 KO | 15 | 5.72E-11 | 6.68E-12 | 1.73E-12 | 15 | 6.44E-11 | 6.34E-12 | 1.64E-12 |
|  | Young | 12 | 5.07E-11 | 4.24E-12 | 1.22E-12 | 12 | 6.08E-11 | 4.93E-12 | 1.42E-12 |
|  | Old | 15 | 6.02E-11 | 5.43E-12 | 1.40E-12 | 15 | 6.78E-11 | 4.16E-12 | 1.07E-12 |
| *N*-oleoyl serine | WT young | 6 | 7.37E-11 | 7.05E-12 | 2.88E-12 | 6 | 7.09E-11 | 4.01E-12 | 1.64E-12 |
|  | WT old | 6 | 8.80E-11 | 6.16E-12 | 2.51E-12 | 6 | 7.82E-11 | 7.33E-12 | 2.99E-12 |
|  | WT | 12 | 8.09E-11 | 9.79E-12 | 2.83E-12 | 12 | 7.45E-11 | 6.79E-12 | 1.96E-12 |
|  | KO young | 6 | 7.38E-11 | 4.53E-12 | 1.85E-12 | 6 | 6.35E-11 | 4.37E-12 | 1.78E-12 |
|  | KO old | 9 | 8.05E-11 | 4.92E-12 | 1.64E-12 | 9 | 6.66E-11 | 4.09E-12 | 1.36E-12 |
|  | ABHD12 KO | 15 | 7.78E-11 | 5.72E-12 | 1.48E-12 | 15 | 6.53E-11 | 4.35E-12 | 1.12E-12 |
|  | Young | 12 | 7.37E-11 | 5.65E-12 | 1.63E-12 | 12 | 6.72E-11 | 5.59E-12 | 1.61E-12 |
|  | Old | 15 | 8.35E-11 | 6.48E-12 | 1.67E-12 | 15 | 7.12E-11 | 7.96E-12 | 2.05E-12 |
| *N*-linoleoyl serine | WT young | 6 | 6.25E-12 | 8.78E-13 | 3.59E-13 | 6 | 6.77E-12 | 1.54E-12 | 6.29E-13 |
|  | WT old | 6 | 7.22E-12 | 1.71E-12 | 6.98E-13 | 6 | 6.87E-12 | 1.27E-12 | 5.17E-13 |
|  | WT | 12 | 6.74E-12 | 1.39E-12 | 4.02E-13 | 12 | 6.82E-12 | 1.35E-12 | 3.88E-13 |
|  | KO young | 6 | 7.95E-12 | 1.26E-12 | 5.16E-13 | 6 | 9.31E-12 | 1.07E-12 | 4.37E-13 |
|  | KO old | 9 | 6.63E-12 | 2.28E-12 | 7.60E-13 | 9 | 7.04E-12 | 8.60E-13 | 2.87E-13 |
|  | ABHD12 KO | 15 | 7.16E-12 | 2.00E-12 | 5.16E-13 | 15 | 7.95E-12 | 1.47E-12 | 3.79E-13 |
|  | Young | 12 | 7.10E-12 | 1.36E-12 | 3.94E-13 | 12 | 8.04E-12 | 1.83E-12 | 5.29E-13 |
|  | Old | 15 | 6.87E-12 | 2.03E-12 | 5.23E-13 | 15 | 6.97E-12 | 1.00E-12 | 2.59E-13 |
| *N*-arachidonoyl serine | WT young | 6 | 3.92E-12 | 4.79E-13 | 1.96E-13 | 6 | 4.53E-12 | 1.07E-12 | 4.36E-13 |
|  | WT old | 6 | 4.53E-12 | 5.07E-13 | 2.07E-13 | 6 | 5.15E-12 | 4.95E-13 | 2.02E-13 |
|  | WT | 12 | 4.22E-12 | 5.69E-13 | 1.64E-13 | 12 | 4.84E-12 | 8.57E-13 | 2.47E-13 |
|  | KO young | 6 | 6.58E-12 | 1.28E-12 | 5.21E-13 | 6 | 7.43E-12 | 1.32E-12 | 5.41E-13 |
|  | KO old | 9 | 8.08E-12 | 1.18E-12 | 3.93E-13 | 9 | 7.01E-12 | 8.49E-13 | 2.83E-13 |
|  | ABHD12 KO | 15 | 7.48E-12 | 1.40E-12 | 3.61E-13 | 15 | 7.18E-12 | 1.04E-12 | 2.69E-13 |
|  | Young | 12 | 5.25E-12 | 1.67E-12 | 4.81E-13 | 12 | 5.98E-12 | 1.90E-12 | 5.48E-13 |
|  | Old | 15 | 6.66E-12 | 2.03E-12 | 5.25E-13 | 15 | 6.26E-12 | 1.18E-12 | 3.04E-13 |
| *N*-docosahexaenoyl serine | WT young | 6 | 2.31E-11 | 5.39E-12 | 2.20E-12 | 6 | 2.00E-11 | 2.76E-12 | 1.13E-12 |
|  | WT old | 6 | 2.45E-11 | 2.53E-12 | 1.03E-12 | 6 | 1.94E-11 | 1.55E-12 | 6.31E-13 |
|  | WT | 12 | 2.38E-11 | 4.07E-12 | 1.18E-12 | 12 | 1.97E-11 | 2.16E-12 | 6.23E-13 |
|  | KO young | 6 | 2.12E-11 | 2.63E-12 | 1.07E-12 | 6 | 2.29E-11 | 4.53E-12 | 1.85E-12 |
|  | KO old | 9 | 2.56E-11 | 2.65E-12 | 8.82E-13 | 9 | 2.28E-11 | 2.79E-12 | 9.29E-13 |
|  | ABHD12 KO | 15 | 2.39E-11 | 3.38E-12 | 8.73E-13 | 15 | 2.28E-11 | 3.43E-12 | 8.86E-13 |
|  | Young | 12 | 2.22E-11 | 4.16E-12 | 1.20E-12 | 12 | 2.15E-11 | 3.89E-12 | 1.12E-12 |
|  | Old | 15 | 2.52E-11 | 2.58E-12 | 6.65E-13 | 15 | 2.14E-11 | 2.87E-12 | 7.42E-13 |

Supplemental Table 38: Mean levels of *N*-acyl taurines in the striatum and hippocampus

| Lipid Species | Group | Striatum | | | | Hippocampus | | | |
| --- | --- | --- | --- | --- | --- | --- | --- | --- | --- |
|  |  | N | Mean | SD | SE | N | Mean | SD | SE |
| *N*-palmitoyl taurine | WT young | 6 | 5.50E-10 | 4.59E-11 | 1.87E-11 | 6 | 3.55E-10 | 5.66E-11 | 2.31E-11 |
|  | WT old | 6 | 4.44E-10 | 8.47E-11 | 3.46E-11 | 6 | 4.17E-10 | 5.02E-11 | 2.05E-11 |
|  | WT | 12 | 4.97E-10 | 8.50E-11 | 2.45E-11 | 12 | 3.86E-10 | 6.05E-11 | 1.75E-11 |
|  | KO young | 6 | 4.83E-10 | 6.96E-11 | 2.84E-11 | 6 | 2.96E-10 | 7.01E-11 | 2.86E-11 |
|  | KO old | 9 | 5.09E-10 | 9.25E-11 | 3.08E-11 | 9 | 3.32E-10 | 6.54E-11 | 2.18E-11 |
|  | ABHD12 KO | 15 | 4.98E-10 | 8.25E-11 | 2.13E-11 | 15 | 3.17E-10 | 6.73E-11 | 1.74E-11 |
|  | Young | 12 | 5.16E-10 | 6.62E-11 | 1.91E-11 | 12 | 3.25E-10 | 6.81E-11 | 1.97E-11 |
|  | Old | 15 | 4.83E-10 | 9.23E-11 | 2.38E-11 | 15 | 3.66E-10 | 7.22E-11 | 1.86E-11 |
| *N*-stearoyl taurine | WT young | 6 | 4.15E-10 | 5.20E-11 | 2.12E-11 | 6 | 1.73E-10 | 1.80E-11 | 7.35E-12 |
|  | WT old | 6 | 3.28E-10 | 5.13E-11 | 2.10E-11 | 6 | 1.78E-10 | 9.78E-12 | 3.99E-12 |
|  | WT | 12 | 3.72E-10 | 6.70E-11 | 1.93E-11 | 12 | 1.75E-10 | 1.40E-11 | 4.05E-12 |
|  | KO young | 6 | 4.11E-10 | 6.80E-11 | 2.78E-11 | 6 | 1.49E-10 | 2.34E-11 | 9.53E-12 |
|  | KO old | 9 | 3.95E-10 | 9.10E-11 | 3.03E-11 | 9 | 1.82E-10 | 2.56E-11 | 8.53E-12 |
|  | ABHD12 KO | 15 | 4.01E-10 | 8.03E-11 | 2.07E-11 | 15 | 1.69E-10 | 2.93E-11 | 7.56E-12 |
|  | Young | 12 | 4.13E-10 | 5.77E-11 | 1.67E-11 | 12 | 1.61E-10 | 2.37E-11 | 6.83E-12 |
|  | Old | 15 | 3.68E-10 | 8.24E-11 | 2.13E-11 | 15 | 1.80E-10 | 2.03E-11 | 5.25E-12 |
| *N*-oleoyl taurine | WT young | 6 | 1.22E-10 | 1.40E-11 | 5.72E-12 | 6 | 9.05E-11 | 1.22E-11 | 4.98E-12 |
|  | WT old | 6 | 1.02E-10 | 2.33E-11 | 9.50E-12 | 6 | 9.62E-11 | 1.74E-11 | 7.11E-12 |
|  | WT | 12 | 1.12E-10 | 2.12E-11 | 6.12E-12 | 12 | 9.34E-11 | 1.46E-11 | 4.23E-12 |
|  | KO young | 6 | 1.19E-10 | 2.96E-11 | 1.21E-11 | 6 | 7.34E-11 | 1.12E-11 | 4.58E-12 |
|  | KO old | 9 | 1.15E-10 | 2.45E-11 | 8.15E-12 | 9 | 8.46E-11 | 1.52E-11 | 5.06E-12 |
|  | ABHD12 KO | 15 | 1.16E-10 | 2.57E-11 | 6.63E-12 | 15 | 8.01E-11 | 1.44E-11 | 3.73E-12 |
|  | Young | 12 | 1.20E-10 | 2.22E-11 | 6.40E-12 | 12 | 8.20E-11 | 1.43E-11 | 4.13E-12 |
|  | Old | 15 | 1.10E-10 | 2.41E-11 | 6.22E-12 | 15 | 8.93E-11 | 1.66E-11 | 4.28E-12 |
| *N*-arachidonoyl taurine | WT young | 6 | 3.08E-10 | 3.25E-11 | 1.33E-11 | 6 | 7.40E-10 | 1.33E-10 | 5.43E-11 |
|  | WT old | 6 | 2.76E-10 | 6.53E-11 | 2.67E-11 | 6 | 7.16E-10 | 9.33E-11 | 3.81E-11 |
|  | WT | 12 | 2.92E-10 | 5.20E-11 | 1.50E-11 | 12 | 7.28E-10 | 1.10E-10 | 3.18E-11 |
|  | KO young | 6 | 3.51E-10 | 5.84E-11 | 2.38E-11 | 6 | 6.78E-10 | 1.57E-10 | 6.40E-11 |
|  | KO old | 9 | 3.44E-10 | 5.79E-11 | 1.93E-11 | 9 | 6.60E-10 | 1.29E-10 | 4.31E-11 |
|  | ABHD12 KO | 15 | 3.46E-10 | 5.61E-11 | 1.45E-11 | 15 | 6.67E-10 | 1.36E-10 | 3.50E-11 |
|  | Young | 12 | 3.29E-10 | 5.02E-11 | 1.45E-11 | 12 | 7.09E-10 | 1.42E-10 | 4.11E-11 |
|  | Old | 15 | 3.17E-10 | 6.79E-11 | 1.75E-11 | 15 | 6.82E-10 | 1.16E-10 | 3.00E-11 |

Supplemental Table 39: Mean levels of *N*-acyl taurines in the cerebellum and thalamus

| Lipid Species | Group | Cerebellum | | | | Thalamus | | | |
| --- | --- | --- | --- | --- | --- | --- | --- | --- | --- |
|  |  | N | Mean | SD | SE | N | Mean | SD | SE |
| *N*-palmitoyl taurine | WT young | 6 | 2.92E-10 | 1.38E-11 | 5.61E-12 | 6 | 4.12E-10 | 3.89E-11 | 1.59E-11 |
|  | WT old | 6 | 2.58E-10 | 2.33E-11 | 9.50E-12 | 6 | 3.90E-10 | 4.72E-11 | 1.93E-11 |
|  | WT | 12 | 2.75E-10 | 2.53E-11 | 7.31E-12 | 12 | 4.01E-10 | 4.28E-11 | 1.24E-11 |
|  | KO young | 6 | 2.36E-10 | 2.41E-11 | 9.83E-12 | 6 | 3.51E-10 | 6.28E-11 | 2.57E-11 |
|  | KO old | 9 | 2.27E-10 | 3.11E-11 | 1.04E-11 | 9 | 4.09E-10 | 4.21E-11 | 1.40E-11 |
|  | ABHD12 KO | 15 | 2.30E-10 | 2.79E-11 | 7.21E-12 | 15 | 3.86E-10 | 5.74E-11 | 1.48E-11 |
|  | Young | 12 | 2.64E-10 | 3.48E-11 | 1.00E-11 | 12 | 3.81E-10 | 5.90E-11 | 1.70E-11 |
|  | Old | 15 | 2.39E-10 | 3.16E-11 | 8.17E-12 | 15 | 4.01E-10 | 4.37E-11 | 1.13E-11 |
| *N*-stearoyl taurine | WT young | 6 | 2.19E-10 | 3.11E-11 | 1.27E-11 | 6 | 2.41E-10 | 2.75E-11 | 1.12E-11 |
|  | WT old | 6 | 1.79E-10 | 5.93E-12 | 2.42E-12 | 6 | 2.14E-10 | 3.33E-11 | 1.36E-11 |
|  | WT | 12 | 1.99E-10 | 2.96E-11 | 8.54E-12 | 12 | 2.28E-10 | 3.22E-11 | 9.28E-12 |
|  | KO young | 6 | 2.13E-10 | 3.65E-11 | 1.49E-11 | 6 | 2.51E-10 | 5.31E-11 | 2.17E-11 |
|  | KO old | 9 | 1.94E-10 | 2.16E-11 | 7.20E-12 | 9 | 2.52E-10 | 2.80E-11 | 9.34E-12 |
|  | ABHD12 KO | 15 | 2.02E-10 | 2.89E-11 | 7.46E-12 | 15 | 2.52E-10 | 3.82E-11 | 9.86E-12 |
|  | Young | 12 | 2.16E-10 | 3.24E-11 | 9.36E-12 | 12 | 2.46E-10 | 4.07E-11 | 1.18E-11 |
|  | Old | 15 | 1.88E-10 | 1.83E-11 | 4.73E-12 | 15 | 2.37E-10 | 3.48E-11 | 8.98E-12 |
| *N*-oleoyl taurine | WT young | 6 | 1.08E-10 | 1.14E-11 | 4.66E-12 | 6 | 1.24E-10 | 1.07E-11 | 4.36E-12 |
|  | WT old | 6 | 9.96E-11 | 1.57E-11 | 6.43E-12 | 6 | 1.01E-10 | 1.70E-11 | 6.94E-12 |
|  | WT | 12 | 1.04E-10 | 1.38E-11 | 3.98E-12 | 12 | 1.12E-10 | 1.80E-11 | 5.19E-12 |
|  | KO young | 6 | 9.80E-11 | 7.50E-12 | 3.06E-12 | 6 | 1.12E-10 | 1.56E-11 | 6.38E-12 |
|  | KO old | 9 | 9.19E-11 | 1.73E-11 | 5.76E-12 | 9 | 1.16E-10 | 1.26E-11 | 4.19E-12 |
|  | ABHD12 KO | 15 | 9.44E-11 | 1.42E-11 | 3.65E-12 | 15 | 1.14E-10 | 1.35E-11 | 3.47E-12 |
|  | Young | 12 | 1.03E-10 | 1.05E-11 | 3.04E-12 | 12 | 1.18E-10 | 1.41E-11 | 4.07E-12 |
|  | Old | 15 | 9.50E-11 | 1.66E-11 | 4.28E-12 | 15 | 1.10E-10 | 1.58E-11 | 4.09E-12 |
| *N*-arachidonoyl taurine | WT young | 6 | 2.84E-10 | 2.61E-11 | 1.07E-11 | 6 | 2.04E-10 | 6.19E-12 | 2.53E-12 |
|  | WT old | 6 | 2.39E-10 | 3.03E-11 | 1.24E-11 | 6 | 1.82E-10 | 2.70E-11 | 1.10E-11 |
|  | WT | 12 | 2.61E-10 | 3.57E-11 | 1.03E-11 | 12 | 1.93E-10 | 2.20E-11 | 6.36E-12 |
|  | KO young | 6 | 3.06E-10 | 4.07E-11 | 1.66E-11 | 6 | 2.32E-10 | 3.49E-11 | 1.43E-11 |
|  | KO old | 9 | 2.82E-10 | 2.66E-11 | 8.86E-12 | 9 | 2.32E-10 | 2.78E-11 | 9.27E-12 |
|  | ABHD12 KO | 15 | 2.92E-10 | 3.39E-11 | 8.74E-12 | 15 | 2.32E-10 | 2.96E-11 | 7.65E-12 |
|  | Young | 12 | 2.95E-10 | 3.47E-11 | 1.00E-11 | 12 | 2.18E-10 | 2.80E-11 | 8.08E-12 |
|  | Old | 15 | 2.65E-10 | 3.49E-11 | 9.01E-12 | 15 | 2.12E-10 | 3.68E-11 | 9.50E-12 |

Supplemental Table 40: Mean levels of *N*-acyl taurines in the cortex and hypothalamus

| Lipid Species | Group | Cortex | | | | Hypothalamus | | | |
| --- | --- | --- | --- | --- | --- | --- | --- | --- | --- |
|  |  | N | Mean | SD | SE | N | Mean | SD | SE |
| *N*-palmitoyl taurine | WT young | 6 | 1.95E-10 | 1.60E-11 | 6.51E-12 | 6 | 9.18E-11 | 9.39E-12 | 3.83E-12 |
|  | WT old | 6 | 1.71E-10 | 1.58E-11 | 6.44E-12 | 5 | 9.00E-11 | 9.29E-12 | 4.15E-12 |
|  | WT | 12 | 1.83E-10 | 1.99E-11 | 5.76E-12 | 11 | 9.10E-11 | 8.91E-12 | 2.69E-12 |
|  | KO young | 6 | 1.84E-10 | 3.11E-11 | 1.27E-11 | 6 | 6.14E-11 | 9.18E-12 | 3.75E-12 |
|  | KO old | 9 | 1.80E-10 | 1.65E-11 | 5.50E-12 | 7 | 6.78E-11 | 1.65E-11 | 6.24E-12 |
|  | ABHD12 KO | 15 | 1.81E-10 | 2.24E-11 | 5.79E-12 | 13 | 6.49E-11 | 1.35E-11 | 3.75E-12 |
|  | Young | 12 | 1.90E-10 | 2.43E-11 | 7.02E-12 | 12 | 7.66E-11 | 1.82E-11 | 5.24E-12 |
|  | Old | 15 | 1.76E-10 | 1.63E-11 | 4.21E-12 | 12 | 7.70E-11 | 1.76E-11 | 5.08E-12 |
| *N*-stearoyl taurine | WT young | 6 | 1.05E-10 | 6.92E-12 | 2.82E-12 | 6 | 4.93E-11 | 7.60E-12 | 3.10E-12 |
|  | WT old | 6 | 9.81E-11 | 7.84E-12 | 3.20E-12 | 5 | 4.78E-11 | 1.04E-11 | 4.65E-12 |
|  | WT | 12 | 1.02E-10 | 7.99E-12 | 2.31E-12 | 11 | 4.86E-11 | 8.53E-12 | 2.57E-12 |
|  | KO young | 6 | 1.09E-10 | 1.84E-11 | 7.52E-12 | 6 | 2.98E-11 | 6.35E-12 | 2.59E-12 |
|  | KO old | 9 | 1.10E-10 | 9.58E-12 | 3.19E-12 | 7 | 4.29E-11 | 1.35E-11 | 5.12E-12 |
|  | ABHD12 KO | 15 | 1.10E-10 | 1.32E-11 | 3.41E-12 | 13 | 3.69E-11 | 1.24E-11 | 3.45E-12 |
|  | Young | 12 | 1.07E-10 | 1.34E-11 | 3.86E-12 | 12 | 3.95E-11 | 1.22E-11 | 3.52E-12 |
|  | Old | 15 | 1.06E-10 | 1.06E-11 | 2.75E-12 | 12 | 4.50E-11 | 1.21E-11 | 3.48E-12 |
| *N*-oleoyl taurine | WT young | 6 | 5.95E-11 | 4.70E-12 | 1.92E-12 | 6 | 3.16E-11 | 5.30E-12 | 2.16E-12 |
|  | WT old | 6 | 5.01E-11 | 4.00E-12 | 1.63E-12 | 5 | 2.41E-11 | 5.00E-12 | 2.24E-12 |
|  | WT | 12 | 5.48E-11 | 6.41E-12 | 1.85E-12 | 11 | 2.82E-11 | 6.25E-12 | 1.88E-12 |
|  | KO young | 6 | 5.57E-11 | 9.68E-12 | 3.95E-12 | 6 | 2.30E-11 | 4.53E-12 | 1.85E-12 |
|  | KO old | 9 | 5.81E-11 | 6.45E-12 | 2.15E-12 | 7 | 2.13E-11 | 5.09E-12 | 1.92E-12 |
|  | ABHD12 KO | 15 | 5.71E-11 | 7.66E-12 | 1.98E-12 | 13 | 2.20E-11 | 4.72E-12 | 1.31E-12 |
|  | Young | 12 | 5.76E-11 | 7.52E-12 | 2.17E-12 | 12 | 2.73E-11 | 6.50E-12 | 1.88E-12 |
|  | Old | 15 | 5.49E-11 | 6.77E-12 | 1.75E-12 | 12 | 2.25E-11 | 5.04E-12 | 1.46E-12 |
| *N*-arachidonoyl taurine | WT young | 6 | 2.76E-10 | 2.56E-11 | 1.05E-11 | 6 | 4.23E-11 | 6.01E-12 | 2.45E-12 |
|  | WT old | 6 | 2.10E-10 | 1.24E-11 | 5.04E-12 | 5 | 3.59E-11 | 5.42E-12 | 2.42E-12 |
|  | WT | 12 | 2.43E-10 | 3.93E-11 | 1.13E-11 | 11 | 3.94E-11 | 6.40E-12 | 1.93E-12 |
|  | KO young | 6 | 2.83E-10 | 7.86E-11 | 3.21E-11 | 6 | 3.84E-11 | 1.03E-11 | 4.21E-12 |
|  | KO old | 9 | 2.84E-10 | 3.62E-11 | 1.21E-11 | 7 | 4.54E-11 | 5.39E-12 | 2.04E-12 |
|  | ABHD12 KO | 15 | 2.83E-10 | 5.44E-11 | 1.40E-11 | 13 | 4.22E-11 | 8.47E-12 | 2.35E-12 |
|  | Young | 12 | 2.79E-10 | 5.58E-11 | 1.61E-11 | 12 | 4.04E-11 | 8.29E-12 | 2.39E-12 |
|  | Old | 15 | 2.54E-10 | 4.68E-11 | 1.21E-11 | 12 | 4.14E-11 | 7.09E-12 | 2.05E-12 |

Supplemental Table 41: Mean levels of *N*-acyl taurines in the midbrain and brainstem

| Lipid Species | Group | Midbrain | | | | Brainstem | | | |
| --- | --- | --- | --- | --- | --- | --- | --- | --- | --- |
|  |  | N | Mean | SD | SE | N | Mean | SD | SE |
| *N*-palmitoyl taurine | WT young | 6 | 1.42E-10 | 9.33E-12 | 3.81E-12 | 6 | 1.87E-10 | 1.62E-11 | 6.61E-12 |
|  | WT old | 6 | 1.41E-10 | 7.95E-12 | 3.25E-12 | 6 | 1.95E-10 | 7.03E-12 | 2.87E-12 |
|  | WT | 12 | 1.42E-10 | 8.29E-12 | 2.39E-12 | 12 | 1.91E-10 | 1.26E-11 | 3.65E-12 |
|  | KO young | 6 | 1.23E-10 | 2.41E-11 | 9.83E-12 | 6 | 1.50E-10 | 1.50E-11 | 6.12E-12 |
|  | KO old | 9 | 1.34E-10 | 1.94E-11 | 6.47E-12 | 9 | 1.73E-10 | 1.08E-11 | 3.59E-12 |
|  | ABHD12 KO | 15 | 1.29E-10 | 2.13E-11 | 5.49E-12 | 15 | 1.64E-10 | 1.69E-11 | 4.36E-12 |
|  | Young | 12 | 1.33E-10 | 2.01E-11 | 5.80E-12 | 12 | 1.68E-10 | 2.44E-11 | 7.06E-12 |
|  | Old | 15 | 1.37E-10 | 1.59E-11 | 4.10E-12 | 15 | 1.82E-10 | 1.45E-11 | 3.74E-12 |
| *N*-stearoyl taurine | WT young | 6 | 6.49E-11 | 8.05E-12 | 3.29E-12 | 6 | 7.77E-11 | 6.79E-12 | 2.77E-12 |
|  | WT old | 6 | 6.43E-11 | 7.75E-12 | 3.16E-12 | 6 | 8.76E-11 | 4.49E-12 | 1.83E-12 |
|  | WT | 12 | 6.46E-11 | 7.54E-12 | 2.18E-12 | 12 | 8.27E-11 | 7.54E-12 | 2.18E-12 |
|  | KO young | 6 | 5.86E-11 | 1.72E-11 | 7.02E-12 | 6 | 6.44E-11 | 9.86E-12 | 4.03E-12 |
|  | KO old | 9 | 6.33E-11 | 5.94E-12 | 1.98E-12 | 9 | 7.47E-11 | 4.70E-12 | 1.57E-12 |
|  | ABHD12 KO | 15 | 6.14E-11 | 1.15E-11 | 2.96E-12 | 15 | 7.06E-11 | 8.66E-12 | 2.24E-12 |
|  | Young | 12 | 6.18E-11 | 1.32E-11 | 3.81E-12 | 12 | 7.10E-11 | 1.07E-11 | 3.08E-12 |
|  | Old | 15 | 6.37E-11 | 6.47E-12 | 1.67E-12 | 15 | 7.99E-11 | 7.92E-12 | 2.04E-12 |
| *N*-oleoyl taurine | WT young | 6 | 4.72E-11 | 5.78E-12 | 2.36E-12 | 6 | 5.55E-11 | 4.54E-12 | 1.85E-12 |
|  | WT old | 6 | 4.56E-11 | 2.27E-12 | 9.26E-13 | 6 | 6.05E-11 | 3.99E-12 | 1.63E-12 |
|  | WT | 12 | 4.64E-11 | 4.27E-12 | 1.23E-12 | 12 | 5.80E-11 | 4.85E-12 | 1.40E-12 |
|  | KO young | 6 | 4.34E-11 | 7.52E-12 | 3.07E-12 | 6 | 5.38E-11 | 5.59E-12 | 2.28E-12 |
|  | KO old | 9 | 4.43E-11 | 3.36E-12 | 1.12E-12 | 9 | 5.43E-11 | 4.74E-12 | 1.58E-12 |
|  | ABHD12 KO | 15 | 4.39E-11 | 5.19E-12 | 1.34E-12 | 15 | 5.41E-11 | 4.90E-12 | 1.27E-12 |
|  | Young | 12 | 4.53E-11 | 6.70E-12 | 1.93E-12 | 12 | 5.47E-11 | 4.94E-12 | 1.42E-12 |
|  | Old | 15 | 4.48E-11 | 2.95E-12 | 7.62E-13 | 15 | 5.68E-11 | 5.35E-12 | 1.38E-12 |
| *N*-arachidonoyl taurine | WT young | 6 | 5.73E-11 | 7.52E-12 | 3.07E-12 | 6 | 4.96E-11 | 6.17E-12 | 2.52E-12 |
|  | WT old | 6 | 5.27E-11 | 4.30E-12 | 1.75E-12 | 6 | 4.43E-11 | 4.64E-12 | 1.89E-12 |
|  | WT | 12 | 5.50E-11 | 6.31E-12 | 1.82E-12 | 12 | 4.70E-11 | 5.90E-12 | 1.70E-12 |
|  | KO young | 6 | 6.99E-11 | 1.43E-11 | 5.84E-12 | 6 | 5.65E-11 | 9.04E-12 | 3.69E-12 |
|  | KO old | 9 | 6.35E-11 | 1.02E-11 | 3.42E-12 | 9 | 5.72E-11 | 4.60E-12 | 1.53E-12 |
|  | ABHD12 KO | 15 | 6.60E-11 | 1.20E-11 | 3.09E-12 | 15 | 5.69E-11 | 6.44E-12 | 1.66E-12 |
|  | Young | 12 | 6.36E-11 | 1.27E-11 | 3.67E-12 | 12 | 5.31E-11 | 8.20E-12 | 2.37E-12 |
|  | Old | 15 | 5.92E-11 | 9.83E-12 | 2.54E-12 | 15 | 5.20E-11 | 7.91E-12 | 2.04E-12 |

Supplemental Table 42: Mean levels of *N*-acyl tryptophans in the striatum and hippocampus

| Lipid Species | Group | Striatum | | | | Hippocampus | | | |
| --- | --- | --- | --- | --- | --- | --- | --- | --- | --- |
|  |  | N | Mean | SD | SE | N | Mean | SD | SE |
| *N*-palmitoyl tryptophan | WT young | 6 | 7.36E-13 | 1.86E-13 | 7.60E-14 | 6 | 3.58E-13 | 1.14E-13 | 4.65E-14 |
|  | WT old | 6 | 7.20E-13 | 8.76E-14 | 3.58E-14 | 6 | 3.44E-13 | 7.19E-14 | 2.93E-14 |
|  | WT | 12 | 7.28E-13 | 1.39E-13 | 4.01E-14 | 12 | 3.51E-13 | 9.11E-14 | 2.63E-14 |
|  | KO young | 6 | 7.95E-13 | 3.86E-13 | 1.58E-13 | 6 | 3.80E-13 | 1.60E-13 | 6.54E-14 |
|  | KO old | 9 | 8.24E-13 | 1.91E-13 | 6.36E-14 | 9 | 3.26E-13 | 1.12E-13 | 3.74E-14 |
|  | ABHD12 KO | 15 | 8.12E-13 | 2.72E-13 | 7.03E-14 | 15 | 3.48E-13 | 1.31E-13 | 3.38E-14 |
|  | Young | 12 | 7.65E-13 | 2.91E-13 | 8.39E-14 | 12 | 3.69E-13 | 1.33E-13 | 3.84E-14 |
|  | Old | 15 | 7.83E-13 | 1.62E-13 | 4.19E-14 | 15 | 3.33E-13 | 9.55E-14 | 2.47E-14 |
| *N*-stearoyl tryptophan | WT young | 6 | 1.53E-12 | 4.12E-13 | 1.68E-13 | 6 | 1.05E-12 | 2.57E-13 | 1.05E-13 |
|  | WT old | 6 | 1.66E-12 | 4.57E-13 | 1.86E-13 | 6 | 1.07E-12 | 2.21E-13 | 9.00E-14 |
|  | WT | 12 | 1.59E-12 | 4.20E-13 | 1.21E-13 | 12 | 1.06E-12 | 2.28E-13 | 6.59E-14 |
|  | KO young | 6 | 1.93E-12 | 4.66E-13 | 1.90E-13 | 6 | 1.22E-12 | 3.75E-13 | 1.53E-13 |
|  | KO old | 9 | 1.74E-12 | 3.98E-13 | 1.33E-13 | 9 | 1.25E-12 | 2.37E-13 | 7.89E-14 |
|  | ABHD12 KO | 15 | 1.82E-12 | 4.21E-13 | 1.09E-13 | 15 | 1.24E-12 | 2.87E-13 | 7.41E-14 |
|  | Young | 12 | 1.73E-12 | 4.68E-13 | 1.35E-13 | 12 | 1.13E-12 | 3.19E-13 | 9.21E-14 |
|  | Old | 15 | 1.71E-12 | 4.08E-13 | 1.05E-13 | 15 | 1.18E-12 | 2.41E-13 | 6.23E-14 |
| *N*-oleoyl tryptophan | WT young | 6 | PISSR |  |  | 6 | PISSR |  |  |
|  | WT old | 6 | PISSR |  |  | 6 | PISSR |  |  |
|  | WT | 12 | PISSR |  |  | 12 | PISSR |  |  |
|  | KO young | 6 | PISSR |  |  | 6 | PISSR |  |  |
|  | KO old | 9 | PISSR |  |  | 9 | PISSR |  |  |
|  | ABHD12 KO | 15 | PISSR |  |  | 15 | PISSR |  |  |
|  | Young | 12 | PISSR |  |  | 12 | PISSR |  |  |
|  | Old | 15 | PISSR |  |  | 15 | PISSR |  |  |
| *N*-linoleoyl tryptophan | WT young | 6 | BDL |  |  | 6 | BDL |  |  |
|  | WT old | 6 | BDL |  |  | 6 | BDL |  |  |
|  | WT | 12 | BDL |  |  | 12 | BDL |  |  |
|  | KO young | 6 | BDL |  |  | 6 | BDL |  |  |
|  | KO old | 9 | BDL |  |  | 9 | BDL |  |  |
|  | ABHD12 KO | 15 | BDL |  |  | 15 | BDL |  |  |
|  | Young | 12 | BDL |  |  | 12 | BDL |  |  |
|  | Old | 15 | BDL |  |  | 15 | BDL |  |  |
| *N*-arachidonoyl tryptophan | WT young | 6 | BDL |  |  | 6 | BDL |  |  |
|  | WT old | 6 | BDL |  |  | 6 | BDL |  |  |
|  | WT | 12 | BDL |  |  | 12 | BDL |  |  |
|  | KO young | 6 | BDL |  |  | 6 | BDL |  |  |
|  | KO old | 9 | BDL |  |  | 9 | BDL |  |  |
|  | ABHD12 KO | 15 | BDL |  |  | 15 | BDL |  |  |
|  | Young | 12 | BDL |  |  | 12 | BDL |  |  |
|  | Old | 15 | BDL |  |  | 15 | BDL |  |  |
| *N*-docosahexaenoyl tryptophan | WT young | 6 | PISSR |  |  | 6 | BDL |  |  |
|  | WT old | 6 | PISSR |  |  | 6 | BDL |  |  |
|  | WT | 12 | PISSR |  |  | 12 | BDL |  |  |
|  | KO young | 6 | PISSR |  |  | 6 | BDL |  |  |
|  | KO old | 9 | PISSR |  |  | 9 | BDL |  |  |
|  | ABHD12 KO | 15 | PISSR |  |  | 15 | BDL |  |  |
|  | Young | 12 | PISSR |  |  | 12 | BDL |  |  |
|  | Old | 15 | PISSR |  |  | 15 | BDL |  |  |

Supplemental Table 43: Mean levels of *N*-acyl tryptophans in the cerebellum and thalamus

| Lipid Species | Group | Cerebellum | | | | Thalamus | | | |
| --- | --- | --- | --- | --- | --- | --- | --- | --- | --- |
|  |  | N | Mean | SD | SE | N | Mean | SD | SE |
| *N*-palmitoyl tryptophan | WT young | 6 | 2.00E-13 | 8.01E-14 | 3.27E-14 | 6 | 1.79E-13 | 7.55E-14 | 3.08E-14 |
|  | WT old | 6 | 2.12E-13 | 9.29E-14 | 3.79E-14 | 6 | 2.48E-13 | 1.02E-13 | 4.15E-14 |
|  | WT | 12 | 2.06E-13 | 8.30E-14 | 2.40E-14 | 12 | 2.14E-13 | 9.26E-14 | 2.67E-14 |
|  | KO young | 6 | 2.12E-13 | 8.69E-14 | 3.55E-14 | 6 | 3.14E-13 | 1.73E-13 | 7.06E-14 |
|  | KO old | 9 | 2.09E-13 | 8.37E-14 | 2.79E-14 | 9 | 2.02E-13 | 6.13E-14 | 2.04E-14 |
|  | ABHD12 KO | 15 | 2.10E-13 | 8.19E-14 | 2.11E-14 | 15 | 2.47E-13 | 1.27E-13 | 3.28E-14 |
|  | Young | 12 | 2.06E-13 | 8.00E-14 | 2.31E-14 | 12 | 2.47E-13 | 1.45E-13 | 4.20E-14 |
|  | Old | 15 | 2.11E-13 | 8.42E-14 | 2.17E-14 | 15 | 2.20E-13 | 8.00E-14 | 2.06E-14 |
| *N*-stearoyl tryptophan | WT young | 6 | 1.19E-12 | 1.93E-13 | 7.88E-14 | 6 | 7.49E-13 | 2.58E-13 | 1.05E-13 |
|  | WT old | 6 | 1.26E-12 | 2.54E-13 | 1.04E-13 | 6 | 8.50E-13 | 1.52E-13 | 6.19E-14 |
|  | WT | 12 | 1.23E-12 | 2.19E-13 | 6.31E-14 | 12 | 8.00E-13 | 2.09E-13 | 6.03E-14 |
|  | KO young | 6 | 1.27E-12 | 1.25E-13 | 5.11E-14 | 6 | 1.11E-12 | 4.88E-13 | 1.99E-13 |
|  | KO old | 9 | 1.08E-12 | 1.35E-13 | 4.49E-14 | 9 | 9.25E-13 | 2.25E-13 | 7.50E-14 |
|  | ABHD12 KO | 15 | 1.16E-12 | 1.58E-13 | 4.07E-14 | 15 | 9.97E-13 | 3.50E-13 | 9.03E-14 |
|  | Young | 12 | 1.23E-12 | 1.61E-13 | 4.64E-14 | 12 | 9.27E-13 | 4.16E-13 | 1.20E-13 |
|  | Old | 15 | 1.16E-12 | 2.05E-13 | 5.29E-14 | 15 | 8.95E-13 | 1.96E-13 | 5.07E-14 |
| *N*-oleoyl tryptophan | WT young | 6 | 8.63E-14 | 3.22E-14 | 1.32E-14 | 6 | PISSR |  |  |
|  | WT old | 6 | 9.39E-14 | 5.25E-14 | 2.14E-14 | 6 | PISSR |  |  |
|  | WT | 12 | 9.01E-14 | 4.17E-14 | 1.20E-14 | 12 | PISSR |  |  |
|  | KO young | 6 | 1.44E-13 | 9.20E-14 | 3.76E-14 | 6 | PISSR |  |  |
|  | KO old | 9 | 1.57E-13 | 6.89E-14 | 2.30E-14 | 9 | PISSR |  |  |
|  | ABHD12 KO | 15 | 1.52E-13 | 7.61E-14 | 1.96E-14 | 15 | PISSR |  |  |
|  | Young | 12 | 1.15E-13 | 7.22E-14 | 2.08E-14 | 12 | PISSR |  |  |
|  | Old | 15 | 1.32E-13 | 6.88E-14 | 1.78E-14 | 15 | PISSR |  |  |
| *N*-linoleoyl tryptophan | WT young | 6 | BDL |  |  | 6 | BDL |  |  |
|  | WT old | 6 | BDL |  |  | 6 | BDL |  |  |
|  | WT | 12 | BDL |  |  | 12 | BDL |  |  |
|  | KO young | 6 | BDL |  |  | 6 | BDL |  |  |
|  | KO old | 9 | BDL |  |  | 9 | BDL |  |  |
|  | ABHD12 KO | 15 | BDL |  |  | 15 | BDL |  |  |
|  | Young | 12 | BDL |  |  | 12 | BDL |  |  |
|  | Old | 15 | BDL |  |  | 15 | BDL |  |  |
| *N*-arachidonoyl tryptophan | WT young | 6 | PISSR |  |  | 6 | BDL |  |  |
|  | WT old | 6 | PISSR |  |  | 6 | BDL |  |  |
|  | WT | 12 | PISSR |  |  | 12 | BDL |  |  |
|  | KO young | 6 | PISSR |  |  | 6 | BDL |  |  |
|  | KO old | 9 | PISSR |  |  | 9 | BDL |  |  |
|  | ABHD12 KO | 15 | PISSR |  |  | 15 | BDL |  |  |
|  | Young | 12 | PISSR |  |  | 12 | BDL |  |  |
|  | Old | 15 | PISSR |  |  | 15 | BDL |  |  |
| *N*-docosahexaenoyl tryptophan | WT young | 6 | PISSR |  |  | 6 | BDL |  |  |
|  | WT old | 6 | PISSR |  |  | 6 | BDL |  |  |
|  | WT | 12 | PISSR |  |  | 12 | BDL |  |  |
|  | KO young | 6 | PISSR |  |  | 6 | BDL |  |  |
|  | KO old | 9 | PISSR |  |  | 9 | BDL |  |  |
|  | ABHD12 KO | 15 | PISSR |  |  | 15 | BDL |  |  |
|  | Young | 12 | PISSR |  |  | 12 | BDL |  |  |
|  | Old | 15 | PISSR |  |  | 15 | BDL |  |  |

Supplemental Table 44: Mean levels of *N*-acyl tryptophans in the cortex and hypothalamus

| Lipid Species | Group | Cortex | | | | Hypothalamus | | | |
| --- | --- | --- | --- | --- | --- | --- | --- | --- | --- |
|  |  | N | Mean | SD | SE | N | Mean | SD | SE |
| *N*-palmitoyl tryptophan | WT young | 6 | 1.18E-13 | 4.92E-14 | 2.01E-14 | 6 | PISSR |  |  |
|  | WT old | 6 | 1.25E-13 | 4.54E-14 | 1.85E-14 | 5 | PISSR |  |  |
|  | WT | 12 | 1.22E-13 | 4.53E-14 | 1.31E-14 | 11 | PISSR |  |  |
|  | KO young | 6 | 1.37E-13 | 9.50E-14 | 3.88E-14 | 6 | PISSR |  |  |
|  | KO old | 9 | 1.32E-13 | 4.85E-14 | 1.62E-14 | 7 | PISSR |  |  |
|  | ABHD12 KO | 15 | 1.34E-13 | 6.76E-14 | 1.75E-14 | 13 | PISSR |  |  |
|  | Young | 12 | 1.27E-13 | 7.28E-14 | 2.10E-14 | 12 | PISSR |  |  |
|  | Old | 15 | 1.29E-13 | 4.58E-14 | 1.18E-14 | 12 | PISSR |  |  |
| *N*-stearoyl tryptophan | WT young | 6 | 1.05E-12 | 2.14E-13 | 8.74E-14 | 6 | 5.15E-13 | 8.66E-14 | 3.53E-14 |
|  | WT old | 6 | 1.14E-12 | 1.78E-13 | 7.28E-14 | 5 | 5.24E-13 | 1.58E-13 | 7.05E-14 |
|  | WT | 12 | 1.09E-12 | 1.94E-13 | 5.60E-14 | 11 | 5.19E-13 | 1.17E-13 | 3.53E-14 |
|  | KO young | 6 | 1.37E-12 | 4.35E-13 | 1.77E-13 | 6 | 5.21E-13 | 1.27E-13 | 5.18E-14 |
|  | KO old | 9 | 1.21E-12 | 1.98E-13 | 6.60E-14 | 7 | 5.02E-13 | 7.47E-14 | 2.82E-14 |
|  | ABHD12 KO | 15 | 1.27E-12 | 3.10E-13 | 8.00E-14 | 13 | 5.11E-13 | 9.80E-14 | 2.72E-14 |
|  | Young | 12 | 1.21E-12 | 3.67E-13 | 1.06E-13 | 12 | 5.18E-13 | 1.04E-13 | 2.99E-14 |
|  | Old | 15 | 1.18E-12 | 1.88E-13 | 4.84E-14 | 12 | 5.11E-13 | 1.11E-13 | 3.19E-14 |
| *N*-oleoyl tryptophan | WT young | 6 | 6.22E-14 | 2.58E-14 | 1.05E-14 | 6 | PISSR |  |  |
|  | WT old | 6 | 5.99E-14 | 2.51E-14 | 1.03E-14 | 5 | PISSR |  |  |
|  | WT | 12 | 6.11E-14 | 2.43E-14 | 7.02E-15 | 11 | PISSR |  |  |
|  | KO young | 6 | 7.98E-14 | 4.89E-14 | 2.00E-14 | 6 | PISSR |  |  |
|  | KO old | 9 | 7.73E-14 | 2.23E-14 | 7.43E-15 | 7 | PISSR |  |  |
|  | ABHD12 KO | 15 | 7.83E-14 | 3.38E-14 | 8.72E-15 | 13 | PISSR |  |  |
|  | Young | 12 | 7.10E-14 | 3.84E-14 | 1.11E-14 | 12 | PISSR |  |  |
|  | Old | 15 | 7.03E-14 | 2.42E-14 | 6.25E-15 | 12 | PISSR |  |  |
| *N*-linoleoyl tryptophan | WT young | 6 | BDL |  |  | 6 | BDL |  |  |
|  | WT old | 6 | BDL |  |  | 5 | BDL |  |  |
|  | WT | 12 | BDL |  |  | 11 | BDL |  |  |
|  | KO young | 6 | BDL |  |  | 6 | BDL |  |  |
|  | KO old | 9 | BDL |  |  | 7 | BDL |  |  |
|  | ABHD12 KO | 15 | BDL |  |  | 13 | BDL |  |  |
|  | Young | 12 | BDL |  |  | 12 | BDL |  |  |
|  | Old | 15 | BDL |  |  | 12 | BDL |  |  |
| *N*-arachidonoyl tryptophan | WT young | 6 | PISSR |  |  | 6 | BDL |  |  |
|  | WT old | 6 | PISSR |  |  | 5 | BDL |  |  |
|  | WT | 12 | PISSR |  |  | 11 | BDL |  |  |
|  | KO young | 6 | PISSR |  |  | 6 | BDL |  |  |
|  | KO old | 9 | PISSR |  |  | 7 | BDL |  |  |
|  | ABHD12 KO | 15 | PISSR |  |  | 13 | BDL |  |  |
|  | Young | 12 | PISSR |  |  | 12 | BDL |  |  |
|  | Old | 15 | PISSR |  |  | 12 | BDL |  |  |
| *N*-docosahexaenoyl tryptophan | WT young | 6 | BDL |  |  | 6 | BDL |  |  |
|  | WT old | 6 | BDL |  |  | 5 | BDL |  |  |
|  | WT | 12 | BDL |  |  | 11 | BDL |  |  |
|  | KO young | 6 | BDL |  |  | 6 | BDL |  |  |
|  | KO old | 9 | BDL |  |  | 7 | BDL |  |  |
|  | ABHD12 KO | 15 | BDL |  |  | 13 | BDL |  |  |
|  | Young | 12 | BDL |  |  | 12 | BDL |  |  |
|  | Old | 15 | BDL |  |  | 12 | BDL |  |  |

Supplemental Table 45: Mean levels of *N*-acyl tryptophans in the midbrain and brainstem

| Lipid Species | Group | Midbrain | | | | Brainstem | | | |
| --- | --- | --- | --- | --- | --- | --- | --- | --- | --- |
|  |  | N | Mean | SD | SE | N | Mean | SD | SE |
| *N*-palmitoyl tryptophan | WT young | 6 | 9.10E-14 | 5.98E-14 | 2.44E-14 | 6 | 1.50E-13 | 2.60E-14 | 1.06E-14 |
|  | WT old | 6 | 1.24E-13 | 2.01E-14 | 8.22E-15 | 6 | 1.39E-13 | 7.58E-14 | 3.10E-14 |
|  | WT | 12 | 1.08E-13 | 4.60E-14 | 1.33E-14 | 12 | 1.44E-13 | 5.44E-14 | 1.57E-14 |
|  | KO young | 6 | 1.43E-13 | 4.30E-14 | 1.76E-14 | 6 | 1.89E-13 | 1.15E-13 | 4.68E-14 |
|  | KO old | 9 | 1.23E-13 | 5.19E-14 | 1.73E-14 | 9 | 1.09E-13 | 4.10E-14 | 1.37E-14 |
|  | ABHD12 KO | 15 | 1.31E-13 | 4.80E-14 | 1.24E-14 | 15 | 1.41E-13 | 8.55E-14 | 2.21E-14 |
|  | Young | 12 | 1.17E-13 | 5.65E-14 | 1.63E-14 | 12 | 1.70E-13 | 8.19E-14 | 2.37E-14 |
|  | Old | 15 | 1.23E-13 | 4.10E-14 | 1.06E-14 | 15 | 1.21E-13 | 5.69E-14 | 1.47E-14 |
| *N*-stearoyl tryptophan | WT young | 6 | 4.91E-13 | 1.28E-13 | 5.22E-14 | 6 | 4.35E-13 | 1.04E-13 | 4.24E-14 |
|  | WT old | 6 | 4.72E-13 | 1.10E-13 | 4.48E-14 | 6 | 4.50E-13 | 5.11E-14 | 2.09E-14 |
|  | WT | 12 | 4.81E-13 | 1.14E-13 | 3.30E-14 | 12 | 4.42E-13 | 7.84E-14 | 2.26E-14 |
|  | KO young | 6 | 5.70E-13 | 1.76E-13 | 7.17E-14 | 6 | 5.45E-13 | 1.77E-13 | 7.22E-14 |
|  | KO old | 9 | 5.32E-13 | 1.05E-13 | 3.50E-14 | 9 | 4.32E-13 | 9.10E-14 | 3.03E-14 |
|  | ABHD12 KO | 15 | 5.47E-13 | 1.33E-13 | 3.43E-14 | 15 | 4.77E-13 | 1.38E-13 | 3.57E-14 |
|  | Young | 12 | 5.31E-13 | 1.52E-13 | 4.39E-14 | 12 | 4.90E-13 | 1.50E-13 | 4.32E-14 |
|  | Old | 15 | 5.08E-13 | 1.07E-13 | 2.77E-14 | 15 | 4.39E-13 | 7.58E-14 | 1.96E-14 |
| *N*-oleoyl tryptophan | WT young | 6 | PISSR |  |  | 6 | 4.14E-14 | 1.45E-14 | 5.91E-15 |
|  | WT old | 6 | PISSR |  |  | 6 | 4.27E-14 | 1.57E-14 | 6.39E-15 |
|  | WT | 12 | PISSR |  |  | 12 | 4.21E-14 | 1.44E-14 | 4.16E-15 |
|  | KO young | 6 | PISSR |  |  | 6 | 1.05E-13 | 2.47E-14 | 1.01E-14 |
|  | KO old | 9 | PISSR |  |  | 9 | 7.69E-14 | 2.43E-14 | 8.11E-15 |
|  | ABHD12 KO | 15 | PISSR |  |  | 15 | 8.80E-14 | 2.75E-14 | 7.10E-15 |
|  | Young | 12 | PISSR |  |  | 12 | 7.31E-14 | 3.83E-14 | 1.11E-14 |
|  | Old | 15 | PISSR |  |  | 15 | 6.32E-14 | 2.69E-14 | 6.95E-15 |
| *N*-linoleoyl tryptophan | WT young | 6 | BDL |  |  | 6 | BDL |  |  |
|  | WT old | 6 | BDL |  |  | 6 | BDL |  |  |
|  | WT | 12 | BDL |  |  | 12 | BDL |  |  |
|  | KO young | 6 | BDL |  |  | 6 | BDL |  |  |
|  | KO old | 9 | BDL |  |  | 9 | BDL |  |  |
|  | ABHD12 KO | 15 | BDL |  |  | 15 | BDL |  |  |
|  | Young | 12 | BDL |  |  | 12 | BDL |  |  |
|  | Old | 15 | BDL |  |  | 15 | BDL |  |  |
| *N*-arachidonoyl tryptophan | WT young | 6 | PISSR |  |  | 6 | BDL |  |  |
|  | WT old | 6 | PISSR |  |  | 6 | BDL |  |  |
|  | WT | 12 | PISSR |  |  | 12 | BDL |  |  |
|  | KO young | 6 | PISSR |  |  | 6 | BDL |  |  |
|  | KO old | 9 | PISSR |  |  | 9 | BDL |  |  |
|  | ABHD12 KO | 15 | PISSR |  |  | 15 | BDL |  |  |
|  | Young | 12 | PISSR |  |  | 12 | BDL |  |  |
|  | Old | 15 | PISSR |  |  | 15 | BDL |  |  |
| *N*-docosahexaenoyl tryptophan | WT young | 6 | BDL |  |  | 6 | BDL |  |  |
|  | WT old | 6 | BDL |  |  | 6 | BDL |  |  |
|  | WT | 12 | BDL |  |  | 12 | BDL |  |  |
|  | KO young | 6 | BDL |  |  | 6 | BDL |  |  |
|  | KO old | 9 | BDL |  |  | 9 | BDL |  |  |
|  | ABHD12 KO | 15 | BDL |  |  | 15 | BDL |  |  |
|  | Young | 12 | BDL |  |  | 12 | BDL |  |  |
|  | Old | 15 | BDL |  |  | 15 | BDL |  |  |

Supplemental Table 46: Mean levels of *N*-acyl tyrosines in the striatum and hippocampus

| Lipid Species | Group | Striatum | | | | Hippocampus | | | |
| --- | --- | --- | --- | --- | --- | --- | --- | --- | --- |
|  |  | N | Mean | SD | SE | N | Mean | SD | SE |
| *N*-palmitoyl tyrosine | WT young | 6 | 1.33E-12 | 2.94E-13 | 1.20E-13 | 6 | 1.06E-12 | 9.38E-14 | 3.83E-14 |
|  | WT old | 6 | 1.32E-12 | 4.28E-13 | 1.75E-13 | 6 | 1.20E-12 | 1.00E-13 | 4.08E-14 |
|  | WT | 12 | 1.33E-12 | 3.50E-13 | 1.01E-13 | 12 | 1.13E-12 | 1.19E-13 | 3.43E-14 |
|  | KO young | 6 | 1.85E-12 | 1.10E-12 | 4.48E-13 | 6 | 1.50E-12 | 9.05E-13 | 3.69E-13 |
|  | KO old | 9 | 1.38E-12 | 2.36E-13 | 7.86E-14 | 9 | 1.08E-12 | 1.15E-13 | 3.83E-14 |
|  | ABHD12 KO | 15 | 1.57E-12 | 7.20E-13 | 1.86E-13 | 15 | 1.25E-12 | 5.86E-13 | 1.51E-13 |
|  | Young | 12 | 1.59E-12 | 8.12E-13 | 2.34E-13 | 12 | 1.28E-12 | 6.55E-13 | 1.89E-13 |
|  | Old | 15 | 1.36E-12 | 3.13E-13 | 8.09E-14 | 15 | 1.13E-12 | 1.21E-13 | 3.12E-14 |
| *N*-stearoyl tyrosine | WT young | 6 | 2.05E-13 | 5.44E-14 | 2.22E-14 | 6 | 1.14E-13 | 2.24E-14 | 9.14E-15 |
|  | WT old | 6 | 1.82E-13 | 9.12E-14 | 3.73E-14 | 6 | 9.65E-14 | 2.65E-14 | 1.08E-14 |
|  | WT | 12 | 1.93E-13 | 7.27E-14 | 2.10E-14 | 12 | 1.05E-13 | 2.51E-14 | 7.25E-15 |
|  | KO young | 6 | 2.80E-13 | 7.76E-14 | 3.17E-14 | 6 | 1.51E-13 | 7.02E-14 | 2.87E-14 |
|  | KO old | 9 | 2.07E-13 | 5.28E-14 | 1.76E-14 | 9 | 1.04E-13 | 2.79E-14 | 9.31E-15 |
|  | ABHD12 KO | 15 | 2.36E-13 | 7.14E-14 | 1.84E-14 | 15 | 1.23E-13 | 5.27E-14 | 1.36E-14 |
|  | Young | 12 | 2.42E-13 | 7.49E-14 | 2.16E-14 | 12 | 1.32E-13 | 5.33E-14 | 1.54E-14 |
|  | Old | 15 | 1.97E-13 | 6.88E-14 | 1.78E-14 | 15 | 1.01E-13 | 2.67E-14 | 6.89E-15 |
| *N*-oleoyl tyrosine | WT young | 6 | 4.15E-13 | 1.71E-13 | 6.98E-14 | 6 | 3.00E-13 | 1.36E-13 | 5.56E-14 |
|  | WT old | 6 | 3.61E-13 | 3.11E-13 | 1.27E-13 | 6 | 3.70E-13 | 1.91E-13 | 7.80E-14 |
|  | WT | 12 | 3.88E-13 | 2.41E-13 | 6.95E-14 | 12 | 3.35E-13 | 1.62E-13 | 4.69E-14 |
|  | KO young | 6 | 6.71E-13 | 2.31E-13 | 9.44E-14 | 6 | 4.78E-13 | 2.98E-13 | 1.22E-13 |
|  | KO old | 9 | 4.65E-13 | 1.80E-13 | 5.99E-14 | 9 | 5.05E-13 | 1.71E-13 | 5.71E-14 |
|  | ABHD12 KO | 15 | 5.47E-13 | 2.20E-13 | 5.68E-14 | 15 | 4.94E-13 | 2.21E-13 | 5.70E-14 |
|  | Young | 12 | 5.43E-13 | 2.36E-13 | 6.80E-14 | 12 | 3.89E-13 | 2.40E-13 | 6.92E-14 |
|  | Old | 15 | 4.23E-13 | 2.36E-13 | 6.09E-14 | 15 | 4.51E-13 | 1.86E-13 | 4.79E-14 |
| *N*-linoleoyl tyrosine | WT young | 6 | PISSR |  |  | 6 | PISSR |  |  |
|  | WT old | 6 | PISSR |  |  | 6 | PISSR |  |  |
|  | WT | 12 | PISSR |  |  | 12 | PISSR |  |  |
|  | KO young | 6 | PISSR |  |  | 6 | PISSR |  |  |
|  | KO old | 9 | PISSR |  |  | 9 | PISSR |  |  |
|  | ABHD12 KO | 15 | PISSR |  |  | 15 | PISSR |  |  |
|  | Young | 12 | PISSR |  |  | 12 | PISSR |  |  |
|  | Old | 15 | PISSR |  |  | 15 | PISSR |  |  |
| *N*-arachidonoyl tyrosine | WT young | 6 | 4.91E-13 | 1.24E-13 | 5.08E-14 | 6 | 5.88E-13 | 1.68E-13 | 6.84E-14 |
|  | WT old | 6 | 3.86E-13 | 1.07E-13 | 4.36E-14 | 6 | 5.68E-13 | 1.86E-13 | 7.61E-14 |
|  | WT | 12 | 4.38E-13 | 1.24E-13 | 3.57E-14 | 12 | 5.78E-13 | 1.69E-13 | 4.89E-14 |
|  | KO young | 6 | 7.96E-13 | 8.87E-14 | 3.62E-14 | 6 | 1.10E-12 | 1.83E-13 | 7.48E-14 |
|  | KO old | 9 | 6.55E-13 | 1.30E-13 | 4.34E-14 | 9 | 8.01E-13 | 2.82E-13 | 9.40E-14 |
|  | ABHD12 KO | 15 | 7.11E-13 | 1.33E-13 | 3.43E-14 | 15 | 9.19E-13 | 2.83E-13 | 7.30E-14 |
|  | Young | 12 | 6.44E-13 | 1.89E-13 | 5.47E-14 | 12 | 8.42E-13 | 3.14E-13 | 9.07E-14 |
|  | Old | 15 | 5.47E-13 | 1.80E-13 | 4.65E-14 | 15 | 7.07E-13 | 2.68E-13 | 6.92E-14 |
| *N*-docosahexaenoyl tyrosine | WT young | 6 | PISSR |  |  | 6 | 6.17E-13 | 2.55E-13 | 1.04E-13 |
|  | WT old | 6 | PISSR |  |  | 6 | 7.98E-13 | 2.27E-13 | 9.26E-14 |
|  | WT | 12 | PISSR |  |  | 12 | 7.08E-13 | 2.49E-13 | 7.18E-14 |
|  | KO young | 6 | PISSR |  |  | 6 | 1.18E-12 | 7.05E-13 | 2.88E-13 |
|  | KO old | 9 | PISSR |  |  | 9 | 8.16E-13 | 1.94E-13 | 6.46E-14 |
|  | ABHD12 KO | 15 | PISSR |  |  | 15 | 9.60E-13 | 4.82E-13 | 1.24E-13 |
|  | Young | 12 | PISSR |  |  | 12 | 8.97E-13 | 5.84E-13 | 1.68E-13 |
|  | Old | 15 | PISSR |  |  | 15 | 8.09E-13 | 2.00E-13 | 5.16E-14 |

Supplemental Table 47: Mean levels of *N*-acyl tyrosines in the cerebellum and thalamus

| Lipid Species | Group | Cerebellum | | | | Thalamus | | | |
| --- | --- | --- | --- | --- | --- | --- | --- | --- | --- |
|  |  | N | Mean | SD | SE | N | Mean | SD | SE |
| *N*-palmitoyl tyrosine | WT young | 6 | 1.17E-12 | 2.46E-13 | 1.00E-13 | 6 | 1.14E-12 | 1.34E-13 | 5.47E-14 |
|  | WT old | 6 | 1.09E-12 | 2.23E-13 | 9.12E-14 | 6 | 1.20E-12 | 2.61E-13 | 1.06E-13 |
|  | WT | 12 | 1.13E-12 | 2.28E-13 | 6.59E-14 | 12 | 1.17E-12 | 2.00E-13 | 5.77E-14 |
|  | KO young | 6 | 1.64E-12 | 9.58E-13 | 3.91E-13 | 6 | 1.80E-12 | 7.97E-13 | 3.26E-13 |
|  | KO old | 9 | 1.10E-12 | 1.50E-13 | 4.99E-14 | 9 | 1.40E-12 | 2.23E-13 | 7.42E-14 |
|  | ABHD12 KO | 15 | 1.32E-12 | 6.46E-13 | 1.67E-13 | 15 | 1.56E-12 | 5.44E-13 | 1.41E-13 |
|  | Young | 12 | 1.41E-12 | 7.11E-13 | 2.05E-13 | 12 | 1.47E-12 | 6.44E-13 | 1.86E-13 |
|  | Old | 15 | 1.09E-12 | 1.75E-13 | 4.52E-14 | 15 | 1.32E-12 | 2.51E-13 | 6.49E-14 |
| *N*-stearoyl tyrosine | WT young | 6 | 2.05E-13 | 6.09E-14 | 2.49E-14 | 6 | 1.33E-13 | 3.56E-14 | 1.45E-14 |
|  | WT old | 6 | 1.84E-13 | 6.59E-14 | 2.69E-14 | 6 | 1.21E-13 | 3.21E-14 | 1.31E-14 |
|  | WT | 12 | 1.95E-13 | 6.15E-14 | 1.77E-14 | 12 | 1.27E-13 | 3.30E-14 | 9.54E-15 |
|  | KO young | 6 | 2.67E-13 | 8.13E-14 | 3.32E-14 | 6 | 2.09E-13 | 8.03E-14 | 3.28E-14 |
|  | KO old | 9 | 1.82E-13 | 4.49E-14 | 1.50E-14 | 9 | 1.46E-13 | 2.64E-14 | 8.81E-15 |
|  | ABHD12 KO | 15 | 2.16E-13 | 7.33E-14 | 1.89E-14 | 15 | 1.71E-13 | 6.10E-14 | 1.58E-14 |
|  | Young | 12 | 2.36E-13 | 7.56E-14 | 2.18E-14 | 12 | 1.71E-13 | 7.12E-14 | 2.06E-14 |
|  | Old | 15 | 1.83E-13 | 5.20E-14 | 1.34E-14 | 15 | 1.36E-13 | 3.06E-14 | 7.89E-15 |
| *N*-oleoyl tyrosine | WT young | 6 | 6.38E-13 | 1.64E-13 | 6.70E-14 | 6 | 5.09E-13 | 8.96E-14 | 3.66E-14 |
|  | WT old | 6 | 5.99E-13 | 2.35E-13 | 9.58E-14 | 6 | 5.63E-13 | 1.41E-13 | 5.76E-14 |
|  | WT | 12 | 6.19E-13 | 1.94E-13 | 5.60E-14 | 12 | 5.36E-13 | 1.16E-13 | 3.35E-14 |
|  | KO young | 6 | 9.34E-13 | 4.58E-13 | 1.87E-13 | 6 | 7.47E-13 | 1.68E-13 | 6.86E-14 |
|  | KO old | 9 | 6.16E-13 | 2.17E-13 | 7.25E-14 | 9 | 5.80E-13 | 1.26E-13 | 4.20E-14 |
|  | ABHD12 KO | 15 | 7.43E-13 | 3.58E-13 | 9.24E-14 | 15 | 6.47E-13 | 1.62E-13 | 4.19E-14 |
|  | Young | 12 | 7.86E-13 | 3.63E-13 | 1.05E-13 | 12 | 6.28E-13 | 1.79E-13 | 5.16E-14 |
|  | Old | 15 | 6.09E-13 | 2.16E-13 | 5.58E-14 | 15 | 5.73E-13 | 1.27E-13 | 3.29E-14 |
| *N*-linoleoyl tyrosine | WT young | 6 | BDL |  |  | 6 | PISSR |  |  |
|  | WT old | 6 | BDL |  |  | 6 | PISSR |  |  |
|  | WT | 12 | BDL |  |  | 12 | PISSR |  |  |
|  | KO young | 6 | BDL |  |  | 6 | PISSR |  |  |
|  | KO old | 9 | BDL |  |  | 9 | PISSR |  |  |
|  | ABHD12 KO | 15 | BDL |  |  | 15 | PISSR |  |  |
|  | Young | 12 | BDL |  |  | 12 | PISSR |  |  |
|  | Old | 15 | BDL |  |  | 15 | PISSR |  |  |
| *N*-arachidonoyl tyrosine | WT young | 6 | 9.98E-13 | 3.16E-13 | 1.29E-13 | 6 | 5.53E-13 | 8.64E-14 | 3.53E-14 |
|  | WT old | 6 | 8.05E-13 | 4.97E-14 | 2.03E-14 | 6 | 5.12E-13 | 8.30E-14 | 3.39E-14 |
|  | WT | 12 | 9.01E-13 | 2.38E-13 | 6.88E-14 | 12 | 5.32E-13 | 8.35E-14 | 2.41E-14 |
|  | KO young | 6 | 2.37E-12 | 8.53E-13 | 3.48E-13 | 6 | 1.14E-12 | 3.73E-13 | 1.52E-13 |
|  | KO old | 9 | 1.50E-12 | 2.84E-13 | 9.48E-14 | 9 | 9.86E-13 | 8.44E-14 | 2.81E-14 |
|  | ABHD12 KO | 15 | 1.85E-12 | 7.09E-13 | 1.83E-13 | 15 | 1.05E-12 | 2.45E-13 | 6.32E-14 |
|  | Young | 12 | 1.68E-12 | 9.43E-13 | 2.72E-13 | 12 | 8.48E-13 | 4.01E-13 | 1.16E-13 |
|  | Old | 15 | 1.22E-12 | 4.12E-13 | 1.06E-13 | 15 | 7.96E-13 | 2.54E-13 | 6.55E-14 |
| *N*-docosahexaenoyl tyrosine | WT young | 6 | 1.46E-12 | 4.99E-13 | 2.04E-13 | 6 | 4.81E-13 | 9.50E-14 | 3.88E-14 |
|  | WT old | 6 | 1.62E-12 | 2.20E-13 | 8.96E-14 | 6 | 5.64E-13 | 1.29E-13 | 5.28E-14 |
|  | WT | 12 | 1.54E-12 | 3.77E-13 | 1.09E-13 | 12 | 5.22E-13 | 1.17E-13 | 3.37E-14 |
|  | KO young | 6 | 1.69E-12 | 6.02E-13 | 2.46E-13 | 6 | 8.41E-13 | 2.53E-13 | 1.03E-13 |
|  | KO old | 9 | 1.30E-12 | 3.63E-13 | 1.21E-13 | 9 | 9.00E-13 | 2.37E-13 | 7.89E-14 |
|  | ABHD12 KO | 15 | 1.46E-12 | 4.93E-13 | 1.27E-13 | 15 | 8.77E-13 | 2.36E-13 | 6.10E-14 |
|  | Young | 12 | 1.57E-12 | 5.40E-13 | 1.56E-13 | 12 | 6.61E-13 | 2.62E-13 | 7.57E-14 |
|  | Old | 15 | 1.43E-12 | 3.45E-13 | 8.91E-14 | 15 | 7.66E-13 | 2.59E-13 | 6.69E-14 |

Supplemental Table 48: Mean levels of *N*-acyl tyrosines in the cortex and hypothalamus

| Lipid Species | Group | Cortex | | | | Hypothalamus | | | |
| --- | --- | --- | --- | --- | --- | --- | --- | --- | --- |
|  |  | N | Mean | SD | SE | N | Mean | SD | SE |
| *N*-palmitoyl tyrosine | WT young | 6 | 1.15E-12 | 1.22E-13 | 4.99E-14 | 6 | 5.65E-13 | 1.33E-13 | 5.42E-14 |
|  | WT old | 6 | 7.01E-13 | 1.03E-13 | 4.20E-14 | 5 | 5.11E-13 | 1.71E-13 | 7.66E-14 |
|  | WT | 12 | 9.28E-13 | 2.60E-13 | 7.51E-14 | 11 | 5.40E-13 | 1.46E-13 | 4.40E-14 |
|  | KO young | 6 | 1.52E-12 | 9.53E-13 | 3.89E-13 | 6 | 6.41E-13 | 1.20E-13 | 4.90E-14 |
|  | KO old | 9 | 7.80E-13 | 1.29E-13 | 4.31E-14 | 7 | 6.48E-13 | 1.79E-13 | 6.76E-14 |
|  | ABHD12 KO | 15 | 1.08E-12 | 6.89E-13 | 1.78E-13 | 13 | 6.45E-13 | 1.48E-13 | 4.12E-14 |
|  | Young | 12 | 1.34E-12 | 6.75E-13 | 1.95E-13 | 12 | 6.03E-13 | 1.27E-13 | 3.67E-14 |
|  | Old | 15 | 7.48E-13 | 1.22E-13 | 3.15E-14 | 12 | 5.91E-13 | 1.82E-13 | 5.25E-14 |
| *N*-stearoyl tyrosine | WT young | 6 | 2.41E-13 | 5.07E-14 | 2.07E-14 | 6 | PISSR |  |  |
|  | WT old | 6 | 1.87E-13 | 3.53E-14 | 1.44E-14 | 5 | PISSR |  |  |
|  | WT | 12 | 2.14E-13 | 5.01E-14 | 1.44E-14 | 11 | PISSR |  |  |
|  | KO young | 6 | 3.06E-13 | 1.11E-13 | 4.52E-14 | 6 | PISSR |  |  |
|  | KO old | 9 | 1.81E-13 | 3.50E-14 | 1.17E-14 | 7 | PISSR |  |  |
|  | ABHD12 KO | 15 | 2.31E-13 | 9.53E-14 | 2.46E-14 | 13 | PISSR |  |  |
|  | Young | 12 | 2.73E-13 | 8.89E-14 | 2.57E-14 | 12 | PISSR |  |  |
|  | Old | 15 | 1.83E-13 | 3.40E-14 | 8.78E-15 | 12 | PISSR |  |  |
| *N*-oleoyl tyrosine | WT young | 6 | 4.09E-13 | 1.11E-13 | 4.53E-14 | 6 | PISSR |  |  |
|  | WT old | 6 | 3.35E-13 | 5.48E-14 | 2.24E-14 | 5 | PISSR |  |  |
|  | WT | 12 | 3.72E-13 | 9.18E-14 | 2.65E-14 | 11 | PISSR |  |  |
|  | KO young | 6 | 6.96E-13 | 4.84E-13 | 1.97E-13 | 6 | PISSR |  |  |
|  | KO old | 9 | 3.21E-13 | 6.46E-14 | 2.15E-14 | 7 | PISSR |  |  |
|  | ABHD12 KO | 15 | 4.71E-13 | 3.50E-13 | 9.03E-14 | 13 | PISSR |  |  |
|  | Young | 12 | 5.52E-13 | 3.67E-13 | 1.06E-13 | 12 | PISSR |  |  |
|  | Old | 15 | 3.27E-13 | 5.93E-14 | 1.53E-14 | 12 | PISSR |  |  |
| *N*-linoleoyl tyrosine | WT young | 6 | BDL |  |  | 6 | BDL |  |  |
|  | WT old | 6 | BDL |  |  | 5 | BDL |  |  |
|  | WT | 12 | BDL |  |  | 11 | BDL |  |  |
|  | KO young | 6 | BDL |  |  | 6 | BDL |  |  |
|  | KO old | 9 | BDL |  |  | 7 | BDL |  |  |
|  | ABHD12 KO | 15 | BDL |  |  | 13 | BDL |  |  |
|  | Young | 12 | BDL |  |  | 12 | BDL |  |  |
|  | Old | 15 | BDL |  |  | 12 | BDL |  |  |
| *N*-arachidonoyl tyrosine | WT young | 6 | 8.11E-13 | 1.06E-13 | 4.32E-14 | 6 | PISSR |  |  |
|  | WT old | 6 | 5.17E-13 | 6.28E-14 | 2.56E-14 | 5 | PISSR |  |  |
|  | WT | 12 | 6.64E-13 | 1.75E-13 | 5.04E-14 | 11 | PISSR |  |  |
|  | KO young | 6 | 1.47E-12 | 4.95E-13 | 2.02E-13 | 6 | PISSR |  |  |
|  | KO old | 9 | 7.88E-13 | 7.00E-14 | 2.33E-14 | 7 | PISSR |  |  |
|  | ABHD12 KO | 15 | 1.06E-12 | 4.56E-13 | 1.18E-13 | 13 | PISSR |  |  |
|  | Young | 12 | 1.14E-12 | 4.83E-13 | 1.39E-13 | 12 | PISSR |  |  |
|  | Old | 15 | 6.80E-13 | 1.52E-13 | 3.93E-14 | 12 | PISSR |  |  |
| *N*-docosahexaenoyl tyrosine | WT young | 6 | 5.44E-13 | 8.52E-14 | 3.48E-14 | 6 | PISSR |  |  |
|  | WT old | 6 | 2.62E-13 | 5.76E-14 | 2.35E-14 | 5 | PISSR |  |  |
|  | WT | 12 | 4.03E-13 | 1.63E-13 | 4.71E-14 | 11 | PISSR |  |  |
|  | KO young | 6 | 6.97E-13 | 1.37E-13 | 5.60E-14 | 6 | PISSR |  |  |
|  | KO old | 9 | 4.76E-13 | 9.05E-14 | 3.02E-14 | 7 | PISSR |  |  |
|  | ABHD12 KO | 15 | 5.64E-13 | 1.55E-13 | 3.99E-14 | 13 | PISSR |  |  |
|  | Young | 12 | 6.20E-13 | 1.35E-13 | 3.89E-14 | 12 | PISSR |  |  |
|  | Old | 15 | 3.91E-13 | 1.33E-13 | 3.44E-14 | 12 | PISSR |  |  |

Supplemental Table 49: Mean levels of *N*-acyl tyrosines in the midbrain and brainstem

| Lipid Species | Group | Midbrain | | | | Brainstem | | | |
| --- | --- | --- | --- | --- | --- | --- | --- | --- | --- |
|  |  | N | Mean | SD | SE | N | Mean | SD | SE |
| *N*-palmitoyl tyrosine | WT young | 6 | 9.65E-13 | 1.25E-13 | 5.10E-14 | 6 | 7.35E-13 | 1.12E-13 | 4.56E-14 |
|  | WT old | 6 | 1.08E-12 | 1.01E-13 | 4.12E-14 | 6 | 7.69E-13 | 6.43E-14 | 2.63E-14 |
|  | WT | 12 | 1.02E-12 | 1.23E-13 | 3.56E-14 | 12 | 7.52E-13 | 8.87E-14 | 2.56E-14 |
|  | KO young | 6 | 1.58E-12 | 7.07E-13 | 2.88E-13 | 6 | 9.65E-13 | 3.62E-13 | 1.48E-13 |
|  | KO old | 9 | 1.18E-12 | 2.18E-13 | 7.27E-14 | 9 | 7.57E-13 | 6.55E-14 | 2.18E-14 |
|  | ABHD12 KO | 15 | 1.34E-12 | 4.97E-13 | 1.28E-13 | 15 | 8.40E-13 | 2.46E-13 | 6.34E-14 |
|  | Young | 12 | 1.27E-12 | 5.80E-13 | 1.68E-13 | 12 | 8.50E-13 | 2.82E-13 | 8.14E-14 |
|  | Old | 15 | 1.14E-12 | 1.83E-13 | 4.72E-14 | 15 | 7.62E-13 | 6.30E-14 | 1.63E-14 |
| *N*-stearoyl tyrosine | WT young | 6 | 1.00E-13 | 2.22E-14 | 9.06E-15 | 6 | 7.64E-14 | 6.92E-15 | 2.82E-15 |
|  | WT old | 6 | 1.03E-13 | 2.47E-14 | 1.01E-14 | 6 | 1.05E-13 | 1.99E-14 | 8.14E-15 |
|  | WT | 12 | 1.02E-13 | 2.24E-14 | 6.47E-15 | 12 | 9.08E-14 | 2.07E-14 | 5.97E-15 |
|  | KO young | 6 | 1.45E-13 | 4.57E-14 | 1.86E-14 | 6 | 1.26E-13 | 5.65E-14 | 2.31E-14 |
|  | KO old | 9 | 1.22E-13 | 2.75E-14 | 9.16E-15 | 9 | 1.01E-13 | 1.63E-14 | 5.45E-15 |
|  | ABHD12 KO | 15 | 1.31E-13 | 3.63E-14 | 9.38E-15 | 15 | 1.11E-13 | 3.82E-14 | 9.87E-15 |
|  | Young | 12 | 1.23E-13 | 4.15E-14 | 1.20E-14 | 12 | 1.01E-13 | 4.64E-14 | 1.34E-14 |
|  | Old | 15 | 1.14E-13 | 2.72E-14 | 7.03E-15 | 15 | 1.03E-13 | 1.73E-14 | 4.47E-15 |
| *N*-oleoyl tyrosine | WT young | 6 | 5.86E-13 | 2.21E-13 | 9.02E-14 | 6 | 3.49E-13 | 6.60E-14 | 2.70E-14 |
|  | WT old | 6 | 6.02E-13 | 1.07E-13 | 4.35E-14 | 6 | 4.43E-13 | 2.90E-14 | 1.19E-14 |
|  | WT | 12 | 5.94E-13 | 1.66E-13 | 4.78E-14 | 12 | 3.96E-13 | 6.88E-14 | 1.99E-14 |
|  | KO young | 6 | 8.81E-13 | 3.67E-13 | 1.50E-13 | 6 | 4.84E-13 | 1.45E-13 | 5.92E-14 |
|  | KO old | 9 | 6.19E-13 | 1.45E-13 | 4.85E-14 | 9 | 3.14E-13 | 7.69E-14 | 2.56E-14 |
|  | ABHD12 KO | 15 | 7.24E-13 | 2.79E-13 | 7.20E-14 | 15 | 3.82E-13 | 1.35E-13 | 3.49E-14 |
|  | Young | 12 | 7.34E-13 | 3.27E-13 | 9.45E-14 | 12 | 4.17E-13 | 1.28E-13 | 3.71E-14 |
|  | Old | 15 | 6.12E-13 | 1.27E-13 | 3.29E-14 | 15 | 3.66E-13 | 8.89E-14 | 2.30E-14 |
| *N*-linoleoyl tyrosine | WT young | 6 | BDL |  |  | 6 | BDL |  |  |
|  | WT old | 6 | BDL |  |  | 6 | BDL |  |  |
|  | WT | 12 | BDL |  |  | 12 | BDL |  |  |
|  | KO young | 6 | BDL |  |  | 6 | BDL |  |  |
|  | KO old | 9 | BDL |  |  | 9 | BDL |  |  |
|  | ABHD12 KO | 15 | BDL |  |  | 15 | BDL |  |  |
|  | Young | 12 | BDL |  |  | 12 | BDL |  |  |
|  | Old | 15 | BDL |  |  | 15 | BDL |  |  |
| *N*-arachidonoyl tyrosine | WT young | 6 | 4.55E-13 | 7.83E-14 | 3.20E-14 | 6 | 5.04E-13 | 8.00E-14 | 3.27E-14 |
|  | WT old | 6 | 4.28E-13 | 1.19E-13 | 4.87E-14 | 6 | 4.69E-13 | 7.80E-14 | 3.19E-14 |
|  | WT | 12 | 4.41E-13 | 9.72E-14 | 2.81E-14 | 12 | 4.87E-13 | 7.74E-14 | 2.24E-14 |
|  | KO young | 6 | 1.07E-12 | 3.07E-13 | 1.25E-13 | 6 | 8.94E-13 | 1.66E-13 | 6.77E-14 |
|  | KO old | 9 | 7.44E-13 | 7.66E-14 | 2.55E-14 | 9 | 7.17E-13 | 1.26E-13 | 4.21E-14 |
|  | ABHD12 KO | 15 | 8.75E-13 | 2.54E-13 | 6.56E-14 | 15 | 7.88E-13 | 1.64E-13 | 4.24E-14 |
|  | Young | 12 | 7.63E-13 | 3.86E-13 | 1.12E-13 | 12 | 6.99E-13 | 2.39E-13 | 6.89E-14 |
|  | Old | 15 | 6.18E-13 | 1.85E-13 | 4.76E-14 | 15 | 6.18E-13 | 1.65E-13 | 4.25E-14 |
| *N*-docosahexaenoyl tyrosine | WT young | 6 | 7.73E-13 | 2.30E-13 | 9.38E-14 | 6 | 3.50E-13 | 7.42E-14 | 3.03E-14 |
|  | WT old | 6 | 7.91E-13 | 1.26E-13 | 5.16E-14 | 6 | 4.84E-13 | 1.28E-13 | 5.24E-14 |
|  | WT | 12 | 7.82E-13 | 1.77E-13 | 5.11E-14 | 12 | 4.17E-13 | 1.22E-13 | 3.52E-14 |
|  | KO young | 6 | 1.21E-12 | 4.44E-13 | 1.81E-13 | 6 | 7.89E-13 | 9.00E-14 | 3.67E-14 |
|  | KO old | 9 | 9.66E-13 | 2.94E-13 | 9.80E-14 | 9 | 5.77E-13 | 1.76E-13 | 5.87E-14 |
|  | ABHD12 KO | 15 | 1.06E-12 | 3.68E-13 | 9.49E-14 | 15 | 6.62E-13 | 1.79E-13 | 4.63E-14 |
|  | Young | 12 | 9.91E-13 | 4.07E-13 | 1.17E-13 | 12 | 5.69E-13 | 2.42E-13 | 6.99E-14 |
|  | Old | 15 | 8.96E-13 | 2.51E-13 | 6.48E-14 | 15 | 5.40E-13 | 1.61E-13 | 4.15E-14 |

Supplemental Table 50: Mean levels of *N*-acyl valines in the striatum and hippocampus

| Lipid Species | Group | Striatum | | | | Hippocampus | | | |
| --- | --- | --- | --- | --- | --- | --- | --- | --- | --- |
|  |  | N | Mean | SD | SE | N | Mean | SD | SE |
| *N*-palmitoyl valine | WT young | 6 | 7.99E-13 | 1.20E-13 | 4.91E-14 | 6 | 5.35E-13 | 1.59E-13 | 6.48E-14 |
|  | WT old | 6 | 1.21E-12 | 8.38E-13 | 3.42E-13 | 6 | 6.15E-13 | 1.41E-13 | 5.75E-14 |
|  | WT | 12 | 1.01E-12 | 6.11E-13 | 1.76E-13 | 12 | 5.75E-13 | 1.49E-13 | 4.30E-14 |
|  | KO young | 6 | 9.44E-13 | 3.41E-13 | 1.39E-13 | 6 | 5.25E-13 | 6.98E-14 | 2.85E-14 |
|  | KO old | 9 | 7.02E-13 | 1.19E-13 | 3.97E-14 | 9 | 5.59E-13 | 1.30E-13 | 4.34E-14 |
|  | ABHD12 KO | 15 | 7.99E-13 | 2.54E-13 | 6.56E-14 | 15 | 5.46E-13 | 1.08E-13 | 2.79E-14 |
|  | Young | 12 | 8.71E-13 | 2.55E-13 | 7.36E-14 | 12 | 5.30E-13 | 1.17E-13 | 3.38E-14 |
|  | Old | 15 | 9.07E-13 | 5.72E-13 | 1.48E-13 | 15 | 5.81E-13 | 1.32E-13 | 3.42E-14 |
| *N*-stearoyl valine | WT young | 6 | 5.83E-13 | 1.41E-13 | 5.75E-14 | 6 | 3.78E-13 | 8.15E-14 | 3.33E-14 |
|  | WT old | 6 | 5.77E-13 | 1.45E-13 | 5.91E-14 | 6 | 3.25E-13 | 1.52E-13 | 6.21E-14 |
|  | WT | 12 | 5.80E-13 | 1.36E-13 | 3.93E-14 | 12 | 3.51E-13 | 1.20E-13 | 3.45E-14 |
|  | KO young | 6 | 7.87E-13 | 1.40E-13 | 5.73E-14 | 6 | 4.32E-13 | 9.05E-14 | 3.69E-14 |
|  | KO old | 9 | 7.06E-13 | 1.29E-13 | 4.32E-14 | 9 | 3.70E-13 | 1.21E-13 | 4.04E-14 |
|  | ABHD12 KO | 15 | 7.38E-13 | 1.35E-13 | 3.49E-14 | 15 | 3.95E-13 | 1.11E-13 | 2.87E-14 |
|  | Young | 12 | 6.85E-13 | 1.71E-13 | 4.94E-14 | 12 | 4.05E-13 | 8.68E-14 | 2.51E-14 |
|  | Old | 15 | 6.54E-13 | 1.46E-13 | 3.78E-14 | 15 | 3.52E-13 | 1.31E-13 | 3.38E-14 |
| *N*-oleoyl valine | WT young | 6 | 6.52E-13 | 1.45E-13 | 5.90E-14 | 6 | 3.76E-13 | 1.01E-13 | 4.12E-14 |
|  | WT old | 6 | 5.72E-13 | 2.72E-13 | 1.11E-13 | 6 | 3.98E-13 | 7.54E-14 | 3.08E-14 |
|  | WT | 12 | 6.12E-13 | 2.12E-13 | 6.12E-14 | 12 | 3.87E-13 | 8.57E-14 | 2.47E-14 |
|  | KO young | 6 | 5.34E-13 | 1.53E-13 | 6.24E-14 | 6 | 3.18E-13 | 1.18E-13 | 4.84E-14 |
|  | KO old | 9 | 5.93E-13 | 1.28E-13 | 4.26E-14 | 9 | 3.68E-13 | 9.51E-14 | 3.17E-14 |
|  | ABHD12 KO | 15 | 5.69E-13 | 1.36E-13 | 3.52E-14 | 15 | 3.48E-13 | 1.04E-13 | 2.69E-14 |
|  | Young | 12 | 5.93E-13 | 1.55E-13 | 4.47E-14 | 12 | 3.47E-13 | 1.09E-13 | 3.15E-14 |
|  | Old | 15 | 5.84E-13 | 1.90E-13 | 4.89E-14 | 15 | 3.80E-13 | 8.62E-14 | 2.23E-14 |
| *N*-linoleoyl valine | WT young | 6 | PISSR |  |  | 6 | PISSR |  |  |
|  | WT old | 6 | PISSR |  |  | 6 | PISSR |  |  |
|  | WT | 12 | PISSR |  |  | 12 | PISSR |  |  |
|  | KO young | 6 | PISSR |  |  | 6 | PISSR |  |  |
|  | KO old | 9 | PISSR |  |  | 9 | PISSR |  |  |
|  | ABHD12 KO | 15 | PISSR |  |  | 15 | PISSR |  |  |
|  | Young | 12 | PISSR |  |  | 12 | PISSR |  |  |
|  | Old | 15 | PISSR |  |  | 15 | PISSR |  |  |
| *N*-docosahexaenoyl valine | WT young | 6 | PISSR |  |  | 6 | PISSR |  |  |
|  | WT old | 6 | PISSR |  |  | 6 | PISSR |  |  |
|  | WT | 12 | PISSR |  |  | 12 | PISSR |  |  |
|  | KO young | 6 | PISSR |  |  | 6 | PISSR |  |  |
|  | KO old | 9 | PISSR |  |  | 9 | PISSR |  |  |
|  | ABHD12 KO | 15 | PISSR |  |  | 15 | PISSR |  |  |
|  | Young | 12 | PISSR |  |  | 12 | PISSR |  |  |
|  | Old | 15 | PISSR |  |  | 15 | PISSR |  |  |

Supplemental Table 51: Mean levels of *N*-acyl valines in the cerebellum and thalamus

| Lipid Species | Group | Cerebellum | | | | Thalamus | | | |
| --- | --- | --- | --- | --- | --- | --- | --- | --- | --- |
|  |  | N | Mean | SD | SE | N | Mean | SD | SE |
| *N*-palmitoyl valine | WT young | 6 | 6.77E-13 | 1.88E-13 | 7.68E-14 | 6 | 7.43E-13 | 1.84E-13 | 7.51E-14 |
|  | WT old | 6 | 8.92E-13 | 3.15E-13 | 1.29E-13 | 6 | 6.91E-13 | 1.31E-13 | 5.33E-14 |
|  | WT | 12 | 7.84E-13 | 2.72E-13 | 7.84E-14 | 12 | 7.17E-13 | 1.54E-13 | 4.46E-14 |
|  | KO young | 6 | 7.46E-13 | 1.25E-13 | 5.10E-14 | 6 | 7.57E-13 | 1.15E-13 | 4.70E-14 |
|  | KO old | 9 | 7.09E-13 | 1.68E-13 | 5.60E-14 | 9 | 8.22E-13 | 2.97E-13 | 9.91E-14 |
|  | ABHD12 KO | 15 | 7.24E-13 | 1.48E-13 | 3.83E-14 | 15 | 7.96E-13 | 2.37E-13 | 6.13E-14 |
|  | Young | 12 | 7.11E-13 | 1.57E-13 | 4.52E-14 | 12 | 7.50E-13 | 1.46E-13 | 4.23E-14 |
|  | Old | 15 | 7.82E-13 | 2.45E-13 | 6.33E-14 | 15 | 7.70E-13 | 2.47E-13 | 6.38E-14 |
| *N*-stearoyl valine | WT young | 6 | 4.74E-13 | 1.35E-13 | 5.49E-14 | 6 | 4.48E-13 | 1.49E-13 | 6.09E-14 |
|  | WT old | 6 | 5.11E-13 | 1.34E-13 | 5.46E-14 | 6 | 3.52E-13 | 9.56E-14 | 3.90E-14 |
|  | WT | 12 | 4.92E-13 | 1.29E-13 | 3.73E-14 | 12 | 4.00E-13 | 1.29E-13 | 3.74E-14 |
|  | KO young | 6 | 4.16E-13 | 6.32E-14 | 2.58E-14 | 6 | 5.60E-13 | 1.74E-13 | 7.09E-14 |
|  | KO old | 9 | 5.02E-13 | 1.03E-13 | 3.42E-14 | 9 | 4.88E-13 | 2.20E-13 | 7.32E-14 |
|  | ABHD12 KO | 15 | 4.68E-13 | 9.66E-14 | 2.50E-14 | 15 | 5.17E-13 | 1.99E-13 | 5.15E-14 |
|  | Young | 12 | 4.45E-13 | 1.05E-13 | 3.02E-14 | 12 | 5.04E-13 | 1.65E-13 | 4.77E-14 |
|  | Old | 15 | 5.05E-13 | 1.12E-13 | 2.88E-14 | 15 | 4.34E-13 | 1.89E-13 | 4.87E-14 |
| *N*-oleoyl valine | WT young | 6 | 4.80E-13 | 9.32E-14 | 3.80E-14 | 6 | 4.14E-13 | 1.94E-13 | 7.93E-14 |
|  | WT old | 6 | 4.79E-13 | 2.55E-13 | 1.04E-13 | 6 | 4.43E-13 | 1.80E-13 | 7.36E-14 |
|  | WT | 12 | 4.80E-13 | 1.83E-13 | 5.28E-14 | 12 | 4.28E-13 | 1.79E-13 | 5.17E-14 |
|  | KO young | 6 | 5.31E-13 | 2.35E-13 | 9.60E-14 | 6 | 4.01E-13 | 8.65E-14 | 3.53E-14 |
|  | KO old | 9 | 3.87E-13 | 1.14E-13 | 3.81E-14 | 9 | 3.83E-13 | 1.32E-13 | 4.41E-14 |
|  | ABHD12 KO | 15 | 4.44E-13 | 1.80E-13 | 4.66E-14 | 15 | 3.90E-13 | 1.13E-13 | 2.92E-14 |
|  | Young | 12 | 5.05E-13 | 1.73E-13 | 4.98E-14 | 12 | 4.07E-13 | 1.43E-13 | 4.14E-14 |
|  | Old | 15 | 4.24E-13 | 1.81E-13 | 4.68E-14 | 15 | 4.07E-13 | 1.50E-13 | 3.87E-14 |
| *N*-linoleoyl valine | WT young | 6 | PISSR |  |  | 6 | PISSR |  |  |
|  | WT old | 6 | PISSR |  |  | 6 | PISSR |  |  |
|  | WT | 12 | PISSR |  |  | 12 | PISSR |  |  |
|  | KO young | 6 | PISSR |  |  | 6 | PISSR |  |  |
|  | KO old | 9 | PISSR |  |  | 9 | PISSR |  |  |
|  | ABHD12 KO | 15 | PISSR |  |  | 15 | PISSR |  |  |
|  | Young | 12 | PISSR |  |  | 12 | PISSR |  |  |
|  | Old | 15 | PISSR |  |  | 15 | PISSR |  |  |
| *N*-docosahexaenoyl valine | WT young | 6 | PISSR |  |  | 6 | PISSR |  |  |
|  | WT old | 6 | PISSR |  |  | 6 | PISSR |  |  |
|  | WT | 12 | PISSR |  |  | 12 | PISSR |  |  |
|  | KO young | 6 | PISSR |  |  | 6 | PISSR |  |  |
|  | KO old | 9 | PISSR |  |  | 9 | PISSR |  |  |
|  | ABHD12 KO | 15 | PISSR |  |  | 15 | PISSR |  |  |
|  | Young | 12 | PISSR |  |  | 12 | PISSR |  |  |
|  | Old | 15 | PISSR |  |  | 15 | PISSR |  |  |

Supplemental Table 52: Mean levels of *N*-acyl valines in the cortex and hypothalamus

| Lipid Species | Group | Cortex | | | | Hypothalamus | | | |
| --- | --- | --- | --- | --- | --- | --- | --- | --- | --- |
|  |  | N | Mean | SD | SE | N | Mean | SD | SE |
| *N*-palmitoyl valine | WT young | 6 | 5.47E-13 | 7.62E-14 | 3.11E-14 | 6 | 5.01E-13 | 1.36E-13 | 5.56E-14 |
|  | WT old | 6 | 5.88E-13 | 8.21E-14 | 3.35E-14 | 5 | 5.87E-13 | 1.30E-13 | 5.81E-14 |
|  | WT | 12 | 5.67E-13 | 7.84E-14 | 2.26E-14 | 11 | 5.40E-13 | 1.34E-13 | 4.05E-14 |
|  | KO young | 6 | 5.98E-13 | 6.24E-14 | 2.55E-14 | 6 | 6.35E-13 | 1.69E-13 | 6.88E-14 |
|  | KO old | 9 | 4.74E-13 | 1.09E-13 | 3.64E-14 | 7 | 6.30E-13 | 1.51E-13 | 5.69E-14 |
|  | ABHD12 KO | 15 | 5.24E-13 | 1.10E-13 | 2.84E-14 | 13 | 6.32E-13 | 1.52E-13 | 4.22E-14 |
|  | Young | 12 | 5.73E-13 | 7.14E-14 | 2.06E-14 | 12 | 5.68E-13 | 1.62E-13 | 4.67E-14 |
|  | Old | 15 | 5.20E-13 | 1.12E-13 | 2.89E-14 | 12 | 6.12E-13 | 1.38E-13 | 3.98E-14 |
| *N*-stearoyl valine | WT young | 6 | 4.94E-13 | 9.93E-14 | 4.06E-14 | 6 | 3.18E-13 | 9.83E-14 | 4.01E-14 |
|  | WT old | 6 | 3.71E-13 | 6.01E-14 | 2.46E-14 | 5 | 3.56E-13 | 1.77E-13 | 7.90E-14 |
|  | WT | 12 | 4.32E-13 | 1.01E-13 | 2.93E-14 | 11 | 3.35E-13 | 1.33E-13 | 4.01E-14 |
|  | KO young | 6 | 5.27E-13 | 8.86E-14 | 3.62E-14 | 6 | 2.91E-13 | 2.95E-14 | 1.20E-14 |
|  | KO old | 9 | 4.93E-13 | 1.04E-13 | 3.46E-14 | 7 | 3.14E-13 | 1.55E-13 | 5.87E-14 |
|  | ABHD12 KO | 15 | 5.07E-13 | 9.62E-14 | 2.48E-14 | 13 | 3.03E-13 | 1.12E-13 | 3.11E-14 |
|  | Young | 12 | 5.10E-13 | 9.14E-14 | 2.64E-14 | 12 | 3.04E-13 | 7.06E-14 | 2.04E-14 |
|  | Old | 15 | 4.44E-13 | 1.06E-13 | 2.75E-14 | 12 | 3.31E-13 | 1.58E-13 | 4.56E-14 |
| *N*-oleoyl valine | WT young | 6 | 4.01E-13 | 8.26E-14 | 3.37E-14 | 6 | PISSR |  |  |
|  | WT old | 6 | 3.55E-13 | 4.91E-14 | 2.00E-14 | 5 | PISSR |  |  |
|  | WT | 12 | 3.78E-13 | 6.90E-14 | 1.99E-14 | 11 | PISSR |  |  |
|  | KO young | 6 | 3.50E-13 | 6.17E-14 | 2.52E-14 | 6 | PISSR |  |  |
|  | KO old | 9 | 3.72E-13 | 9.36E-14 | 3.12E-14 | 7 | PISSR |  |  |
|  | ABHD12 KO | 15 | 3.63E-13 | 8.05E-14 | 2.08E-14 | 13 | PISSR |  |  |
|  | Young | 12 | 3.75E-13 | 7.44E-14 | 2.15E-14 | 12 | PISSR |  |  |
|  | Old | 15 | 3.65E-13 | 7.70E-14 | 1.99E-14 | 12 | PISSR |  |  |
| *N*-linoleoyl valine | WT young | 6 | PISSR |  |  | 6 | PISSR |  |  |
|  | WT old | 6 | PISSR |  |  | 5 | PISSR |  |  |
|  | WT | 12 | PISSR |  |  | 11 | PISSR |  |  |
|  | KO young | 6 | PISSR |  |  | 6 | PISSR |  |  |
|  | KO old | 9 | PISSR |  |  | 7 | PISSR |  |  |
|  | ABHD12 KO | 15 | PISSR |  |  | 13 | PISSR |  |  |
|  | Young | 12 | PISSR |  |  | 12 | PISSR |  |  |
|  | Old | 15 | PISSR |  |  | 12 | PISSR |  |  |
| *N*-docosahexaenoyl valine | WT young | 6 | PISSR |  |  | 6 | PISSR |  |  |
|  | WT old | 6 | PISSR |  |  | 5 | PISSR |  |  |
|  | WT | 12 | PISSR |  |  | 11 | PISSR |  |  |
|  | KO young | 6 | PISSR |  |  | 6 | PISSR |  |  |
|  | KO old | 9 | PISSR |  |  | 7 | PISSR |  |  |
|  | ABHD12 KO | 15 | PISSR |  |  | 13 | PISSR |  |  |
|  | Young | 12 | PISSR |  |  | 12 | PISSR |  |  |
|  | Old | 15 | PISSR |  |  | 12 | PISSR |  |  |

Supplemental Table 53: Mean levels of *N*-acyl valines in the midbrain and brainstem

| Lipid Species | Group | Midbrain | | | | Brainstem | | | |
| --- | --- | --- | --- | --- | --- | --- | --- | --- | --- |
|  |  | N | Mean | SD | SE | N | Mean | SD | SE |
| *N*-palmitoyl valine | WT young | 6 | 4.84E-13 | 6.82E-14 | 2.79E-14 | 6 | 5.70E-13 | 8.14E-14 | 3.32E-14 |
|  | WT old | 6 | 5.46E-13 | 1.05E-13 | 4.28E-14 | 6 | 4.82E-13 | 6.92E-14 | 2.82E-14 |
|  | WT | 12 | 5.15E-13 | 9.03E-14 | 2.61E-14 | 12 | 5.26E-13 | 8.55E-14 | 2.47E-14 |
|  | KO young | 6 | 6.18E-13 | 5.78E-14 | 2.36E-14 | 6 | 5.84E-13 | 8.44E-14 | 3.45E-14 |
|  | KO old | 9 | 5.22E-13 | 7.87E-14 | 2.62E-14 | 9 | 4.70E-13 | 1.07E-13 | 3.58E-14 |
|  | ABHD12 KO | 15 | 5.61E-13 | 8.42E-14 | 2.17E-14 | 15 | 5.16E-13 | 1.12E-13 | 2.89E-14 |
|  | Young | 12 | 5.51E-13 | 9.25E-14 | 2.67E-14 | 12 | 5.77E-13 | 7.94E-14 | 2.29E-14 |
|  | Old | 15 | 5.32E-13 | 8.72E-14 | 2.25E-14 | 15 | 4.75E-13 | 9.12E-14 | 2.36E-14 |
| *N*-stearoyl valine | WT young | 6 | 2.73E-13 | 6.03E-14 | 2.46E-14 | 6 | 2.92E-13 | 5.23E-14 | 2.13E-14 |
|  | WT old | 6 | 2.80E-13 | 6.89E-14 | 2.81E-14 | 6 | 3.00E-13 | 3.42E-14 | 1.40E-14 |
|  | WT | 12 | 2.76E-13 | 6.19E-14 | 1.79E-14 | 12 | 2.96E-13 | 4.23E-14 | 1.22E-14 |
|  | KO young | 6 | 3.22E-13 | 8.97E-14 | 3.66E-14 | 6 | 3.93E-13 | 7.58E-14 | 3.10E-14 |
|  | KO old | 9 | 2.63E-13 | 8.60E-14 | 2.87E-14 | 9 | 3.33E-13 | 5.93E-14 | 1.98E-14 |
|  | ABHD12 KO | 15 | 2.86E-13 | 8.96E-14 | 2.31E-14 | 15 | 3.57E-13 | 7.07E-14 | 1.82E-14 |
|  | Young | 12 | 2.98E-13 | 7.74E-14 | 2.23E-14 | 12 | 3.43E-13 | 8.13E-14 | 2.35E-14 |
|  | Old | 15 | 2.70E-13 | 7.75E-14 | 2.00E-14 | 15 | 3.19E-13 | 5.20E-14 | 1.34E-14 |
| *N*-oleoyl valine | WT young | 6 | 2.48E-13 | 2.62E-14 | 1.07E-14 | 6 | 2.99E-13 | 5.81E-14 | 2.37E-14 |
|  | WT old | 6 | 2.71E-13 | 1.06E-13 | 4.33E-14 | 6 | 3.23E-13 | 6.19E-14 | 2.53E-14 |
|  | WT | 12 | 2.59E-13 | 7.46E-14 | 2.15E-14 | 12 | 3.11E-13 | 5.86E-14 | 1.69E-14 |
|  | KO young | 6 | 2.82E-13 | 1.11E-13 | 4.54E-14 | 6 | 3.94E-13 | 7.70E-14 | 3.14E-14 |
|  | KO old | 9 | 2.49E-13 | 1.02E-13 | 3.39E-14 | 9 | 3.71E-13 | 7.57E-14 | 2.52E-14 |
|  | ABHD12 KO | 15 | 2.62E-13 | 1.03E-13 | 2.66E-14 | 15 | 3.80E-13 | 7.44E-14 | 1.92E-14 |
|  | Young | 12 | 2.65E-13 | 7.91E-14 | 2.28E-14 | 12 | 3.47E-13 | 8.21E-14 | 2.37E-14 |
|  | Old | 15 | 2.58E-13 | 1.00E-13 | 2.59E-14 | 15 | 3.52E-13 | 7.22E-14 | 1.87E-14 |
| *N*-linoleoyl valine | WT young | 6 | PISSR |  |  | 6 | PISSR |  |  |
|  | WT old | 6 | PISSR |  |  | 6 | PISSR |  |  |
|  | WT | 12 | PISSR |  |  | 12 | PISSR |  |  |
|  | KO young | 6 | PISSR |  |  | 6 | PISSR |  |  |
|  | KO old | 9 | PISSR |  |  | 9 | PISSR |  |  |
|  | ABHD12 KO | 15 | PISSR |  |  | 15 | PISSR |  |  |
|  | Young | 12 | PISSR |  |  | 12 | PISSR |  |  |
|  | Old | 15 | PISSR |  |  | 15 | PISSR |  |  |
| *N*-docosahexaenoyl valine | WT young | 6 | PISSR |  |  | 6 | PISSR |  |  |
|  | WT old | 6 | PISSR |  |  | 6 | PISSR |  |  |
|  | WT | 12 | PISSR |  |  | 12 | PISSR |  |  |
|  | KO young | 6 | PISSR |  |  | 6 | PISSR |  |  |
|  | KO old | 9 | PISSR |  |  | 9 | PISSR |  |  |
|  | ABHD12 KO | 15 | PISSR |  |  | 15 | PISSR |  |  |
|  | Young | 12 | PISSR |  |  | 12 | PISSR |  |  |
|  | Old | 15 | PISSR |  |  | 15 | PISSR |  |  |

Supplemental Table 54: Mean levels of 2-acyl glycerols in the striatum and hippocampus

| Lipid Species | Group | Striatum | | | | Hippocampus | | | |
| --- | --- | --- | --- | --- | --- | --- | --- | --- | --- |
|  |  | N | Mean | SD | SE | N | Mean | SD | SE |
| 2-palmitoyl glycerol | WT young | 6 | 8.43E-09 | 1.16E-09 | 4.75E-10 | 6 | 7.16E-09 | 1.91E-09 | 7.78E-10 |
|  | WT old | 6 | 6.36E-09 | 1.53E-09 | 6.26E-10 | 6 | 8.12E-09 | 1.09E-09 | 4.44E-10 |
|  | WT | 12 | 7.39E-09 | 1.69E-09 | 4.87E-10 | 12 | 7.64E-09 | 1.56E-09 | 4.51E-10 |
|  | KO young | 6 | 8.17E-09 | 1.93E-09 | 7.88E-10 | 6 | 7.20E-09 | 2.49E-09 | 1.02E-09 |
|  | KO old | 9 | 5.97E-09 | 1.55E-09 | 5.17E-10 | 9 | 6.93E-09 | 1.61E-09 | 5.35E-10 |
|  | ABHD12 KO | 15 | 6.85E-09 | 1.99E-09 | 5.14E-10 | 15 | 7.04E-09 | 1.92E-09 | 4.97E-10 |
|  | Young | 12 | 8.30E-09 | 1.53E-09 | 4.40E-10 | 12 | 7.18E-09 | 2.11E-09 | 6.10E-10 |
|  | Old | 15 | 6.12E-09 | 1.50E-09 | 3.88E-10 | 15 | 7.40E-09 | 1.50E-09 | 3.88E-10 |
| 2-oleoyl glycerol | WT young | 6 | 6.46E-09 | 9.57E-10 | 3.91E-10 | 6 | 4.24E-09 | 6.16E-10 | 2.52E-10 |
|  | WT old | 6 | 6.49E-09 | 1.69E-09 | 6.90E-10 | 6 | 4.13E-09 | 6.54E-10 | 2.67E-10 |
|  | WT | 12 | 6.47E-09 | 1.31E-09 | 3.78E-10 | 12 | 4.19E-09 | 6.09E-10 | 1.76E-10 |
|  | KO young | 6 | 5.22E-09 | 5.75E-10 | 2.35E-10 | 6 | 3.25E-09 | 8.39E-10 | 3.43E-10 |
|  | KO old | 9 | 5.15E-09 | 8.20E-10 | 2.73E-10 | 9 | 3.40E-09 | 3.29E-10 | 1.10E-10 |
|  | ABHD12 KO | 15 | 5.17E-09 | 7.09E-10 | 1.83E-10 | 15 | 3.34E-09 | 5.65E-10 | 1.46E-10 |
|  | Young | 12 | 5.84E-09 | 9.96E-10 | 2.87E-10 | 12 | 3.75E-09 | 8.74E-10 | 2.52E-10 |
|  | Old | 15 | 5.68E-09 | 1.37E-09 | 3.53E-10 | 15 | 3.69E-09 | 5.93E-10 | 1.53E-10 |
| 2-linoleoyl glycerol | WT young | 6 | 3.23E-10 | 6.66E-11 | 2.72E-11 | 6 | 2.00E-10 | 3.07E-11 | 1.25E-11 |
|  | WT old | 6 | 2.62E-10 | 8.18E-11 | 3.34E-11 | 6 | 1.58E-10 | 2.23E-11 | 9.09E-12 |
|  | WT | 12 | 2.93E-10 | 7.79E-11 | 2.25E-11 | 12 | 1.79E-10 | 3.35E-11 | 9.66E-12 |
|  | KO young | 6 | 3.62E-10 | 9.24E-11 | 3.77E-11 | 6 | 1.96E-10 | 4.69E-11 | 1.92E-11 |
|  | KO old | 9 | 2.57E-10 | 5.26E-11 | 1.75E-11 | 9 | 1.53E-10 | 2.93E-11 | 9.76E-12 |
|  | ABHD12 KO | 15 | 2.99E-10 | 8.66E-11 | 2.23E-11 | 15 | 1.70E-10 | 4.18E-11 | 1.08E-11 |
|  | Young | 12 | 3.43E-10 | 7.94E-11 | 2.29E-11 | 12 | 1.98E-10 | 3.79E-11 | 1.09E-11 |
|  | Old | 15 | 2.59E-10 | 6.31E-11 | 1.63E-11 | 15 | 1.55E-10 | 2.59E-11 | 6.70E-12 |
| 2-arachidonoyl glycerol | WT young | 6 | 2.37E-09 | 3.05E-10 | 1.25E-10 | 6 | 2.13E-09 | 1.80E-10 | 7.34E-11 |
|  | WT old | 6 | 1.94E-09 | 2.59E-10 | 1.06E-10 | 6 | 2.10E-09 | 1.42E-10 | 5.82E-11 |
|  | WT | 12 | 2.16E-09 | 3.50E-10 | 1.01E-10 | 12 | 2.11E-09 | 1.56E-10 | 4.49E-11 |
|  | KO young | 6 | 2.88E-09 | 4.67E-10 | 1.91E-10 | 6 | 2.70E-09 | 7.03E-10 | 2.87E-10 |
|  | KO old | 9 | 2.73E-09 | 5.48E-10 | 1.83E-10 | 9 | 2.14E-09 | 5.06E-10 | 1.69E-10 |
|  | ABHD12 KO | 15 | 2.79E-09 | 5.05E-10 | 1.30E-10 | 15 | 2.36E-09 | 6.37E-10 | 1.65E-10 |
|  | Young | 12 | 2.63E-09 | 4.62E-10 | 1.33E-10 | 12 | 2.42E-09 | 5.75E-10 | 1.66E-10 |
|  | Old | 15 | 2.42E-09 | 5.96E-10 | 1.54E-10 | 15 | 2.12E-09 | 3.93E-10 | 1.01E-10 |

Supplemental Table 55: Mean levels of 2-acyl glycerols in the cerebellum and thalamus

| Lipid Species | Group | Cerebellum | | | | Thalamus | | | |
| --- | --- | --- | --- | --- | --- | --- | --- | --- | --- |
|  |  | N | Mean | SD | SE | N | Mean | SD | SE |
| 2-palmitoyl glycerol | WT young | 6 | 5.46E-09 | 1.07E-09 | 4.36E-10 | 6 | 6.34E-09 | 1.77E-09 | 7.23E-10 |
|  | WT old | 6 | 8.28E-09 | 1.25E-09 | 5.10E-10 | 6 | 5.89E-09 | 1.43E-09 | 5.83E-10 |
|  | WT | 12 | 6.87E-09 | 1.84E-09 | 5.32E-10 | 12 | 6.12E-09 | 1.55E-09 | 4.48E-10 |
|  | KO young | 6 | 4.29E-09 | 8.83E-10 | 3.60E-10 | 6 | 6.57E-09 | 2.28E-09 | 9.29E-10 |
|  | KO old | 9 | 7.10E-09 | 1.29E-09 | 4.29E-10 | 9 | 5.30E-09 | 1.53E-09 | 5.11E-10 |
|  | ABHD12 KO | 15 | 5.98E-09 | 1.80E-09 | 4.66E-10 | 15 | 5.81E-09 | 1.90E-09 | 4.90E-10 |
|  | Young | 12 | 4.88E-09 | 1.12E-09 | 3.22E-10 | 12 | 6.45E-09 | 1.95E-09 | 5.62E-10 |
|  | Old | 15 | 7.57E-09 | 1.36E-09 | 3.52E-10 | 15 | 5.54E-09 | 1.47E-09 | 3.79E-10 |
| 2-oleoyl glycerol | WT young | 6 | 5.37E-09 | 1.11E-09 | 4.54E-10 | 6 | 7.52E-09 | 1.23E-09 | 5.03E-10 |
|  | WT old | 6 | 8.40E-09 | 9.36E-10 | 3.82E-10 | 6 | 8.76E-09 | 1.19E-09 | 4.84E-10 |
|  | WT | 12 | 6.89E-09 | 1.86E-09 | 5.37E-10 | 12 | 8.14E-09 | 1.32E-09 | 3.82E-10 |
|  | KO young | 6 | 4.52E-09 | 6.43E-10 | 2.62E-10 | 6 | 6.97E-09 | 6.63E-10 | 2.71E-10 |
|  | KO old | 9 | 7.76E-09 | 3.97E-10 | 1.32E-10 | 9 | 7.83E-09 | 7.65E-10 | 2.55E-10 |
|  | ABHD12 KO | 15 | 6.46E-09 | 1.72E-09 | 4.43E-10 | 15 | 7.49E-09 | 8.25E-10 | 2.13E-10 |
|  | Young | 12 | 4.95E-09 | 9.74E-10 | 2.81E-10 | 12 | 7.24E-09 | 9.85E-10 | 2.84E-10 |
|  | Old | 15 | 8.02E-09 | 7.13E-10 | 1.84E-10 | 15 | 8.20E-09 | 1.03E-09 | 2.65E-10 |
| 2-linoleoyl glycerol | WT young | 6 | 4.23E-10 | 5.79E-11 | 2.37E-11 | 6 | 3.65E-10 | 4.63E-11 | 1.89E-11 |
|  | WT old | 6 | 4.50E-10 | 4.68E-11 | 1.91E-11 | 6 | 3.11E-10 | 5.08E-11 | 2.07E-11 |
|  | WT | 12 | 4.36E-10 | 5.21E-11 | 1.50E-11 | 12 | 3.38E-10 | 5.45E-11 | 1.57E-11 |
|  | KO young | 6 | 4.60E-10 | 8.90E-11 | 3.63E-11 | 6 | 3.92E-10 | 9.29E-11 | 3.79E-11 |
|  | KO old | 9 | 4.84E-10 | 6.32E-11 | 2.11E-11 | 9 | 3.15E-10 | 5.69E-11 | 1.90E-11 |
|  | ABHD12 KO | 15 | 4.75E-10 | 7.25E-11 | 1.87E-11 | 15 | 3.46E-10 | 8.05E-11 | 2.08E-11 |
|  | Young | 12 | 4.42E-10 | 7.41E-11 | 2.14E-11 | 12 | 3.79E-10 | 7.14E-11 | 2.06E-11 |
|  | Old | 15 | 4.71E-10 | 5.81E-11 | 1.50E-11 | 15 | 3.13E-10 | 5.27E-11 | 1.36E-11 |
| 2-arachidonoyl glycerol | WT young | 6 | 4.47E-09 | 7.16E-10 | 2.92E-10 | 6 | 3.59E-09 | 5.25E-10 | 2.14E-10 |
|  | WT old | 6 | 5.32E-09 | 3.69E-10 | 1.51E-10 | 6 | 3.54E-09 | 5.27E-10 | 2.15E-10 |
|  | WT | 12 | 4.89E-09 | 6.99E-10 | 2.02E-10 | 12 | 3.56E-09 | 5.02E-10 | 1.45E-10 |
|  | KO young | 6 | 5.35E-09 | 9.08E-10 | 3.71E-10 | 6 | 4.64E-09 | 8.30E-10 | 3.39E-10 |
|  | KO old | 9 | 6.21E-09 | 6.83E-10 | 2.28E-10 | 9 | 4.55E-09 | 7.12E-10 | 2.37E-10 |
|  | ABHD12 KO | 15 | 5.86E-09 | 8.67E-10 | 2.24E-10 | 15 | 4.58E-09 | 7.33E-10 | 1.89E-10 |
|  | Young | 12 | 4.91E-09 | 9.03E-10 | 2.61E-10 | 12 | 4.11E-09 | 8.59E-10 | 2.48E-10 |
|  | Old | 15 | 5.85E-09 | 7.21E-10 | 1.86E-10 | 15 | 4.14E-09 | 8.08E-10 | 2.09E-10 |

Supplemental Table 56: Mean levels of 2-acyl glycerols in the cortex and hypothalamus

| Lipid Species | Group | Cortex | | | | Hypothalamus | | | |
| --- | --- | --- | --- | --- | --- | --- | --- | --- | --- |
|  |  | N | Mean | SD | SE | N | Mean | SD | SE |
| 2-palmitoyl glycerol | WT young | 6 | 4.30E-09 | 1.07E-09 | 4.38E-10 | 6 | 1.20E-08 | 3.65E-09 | 1.49E-09 |
|  | WT old | 6 | 5.86E-09 | 9.29E-10 | 3.79E-10 | 5 | 7.30E-09 | 2.11E-09 | 9.45E-10 |
|  | WT | 12 | 5.08E-09 | 1.26E-09 | 3.62E-10 | 11 | 9.85E-09 | 3.79E-09 | 1.14E-09 |
|  | KO young | 6 | 3.08E-09 | 1.07E-09 | 4.35E-10 | 6 | 1.11E-08 | 1.02E-09 | 4.16E-10 |
|  | KO old | 9 | 4.98E-09 | 7.17E-10 | 2.39E-10 | 7 | 6.22E-09 | 1.25E-09 | 4.71E-10 |
|  | ABHD12 KO | 15 | 4.22E-09 | 1.28E-09 | 3.30E-10 | 13 | 8.47E-09 | 2.77E-09 | 7.68E-10 |
|  | Young | 12 | 3.69E-09 | 1.20E-09 | 3.47E-10 | 12 | 1.15E-08 | 2.59E-09 | 7.49E-10 |
|  | Old | 15 | 5.33E-09 | 8.93E-10 | 2.31E-10 | 12 | 6.67E-09 | 1.67E-09 | 4.82E-10 |
| 2-oleoyl glycerol | WT young | 6 | 3.36E-09 | 9.69E-10 | 3.95E-10 | 6 | 5.83E-09 | 1.51E-09 | 6.15E-10 |
|  | WT old | 6 | 4.37E-09 | 6.16E-10 | 2.51E-10 | 5 | 5.11E-09 | 1.05E-09 | 4.69E-10 |
|  | WT | 12 | 3.86E-09 | 9.37E-10 | 2.70E-10 | 11 | 5.51E-09 | 1.31E-09 | 3.95E-10 |
|  | KO young | 6 | 2.56E-09 | 8.17E-10 | 3.34E-10 | 6 | 5.96E-09 | 1.55E-09 | 6.32E-10 |
|  | KO old | 9 | 4.00E-09 | 5.84E-10 | 1.95E-10 | 7 | 3.73E-09 | 1.10E-09 | 4.16E-10 |
|  | ABHD12 KO | 15 | 3.42E-09 | 9.85E-10 | 2.54E-10 | 13 | 4.76E-09 | 1.71E-09 | 4.75E-10 |
|  | Young | 12 | 2.96E-09 | 9.52E-10 | 2.75E-10 | 12 | 5.90E-09 | 1.46E-09 | 4.21E-10 |
|  | Old | 15 | 4.15E-09 | 6.05E-10 | 1.56E-10 | 12 | 4.31E-09 | 1.25E-09 | 3.61E-10 |
| 2-linoleoyl glycerol | WT young | 6 | 1.97E-10 | 4.60E-11 | 1.88E-11 | 6 | 2.62E-10 | 9.94E-11 | 4.06E-11 |
|  | WT old | 6 | 2.04E-10 | 2.52E-11 | 1.03E-11 | 5 | 1.88E-10 | 4.48E-11 | 2.00E-11 |
|  | WT | 12 | 2.01E-10 | 3.56E-11 | 1.03E-11 | 11 | 2.29E-10 | 8.51E-11 | 2.57E-11 |
|  | KO young | 6 | 1.81E-10 | 6.04E-11 | 2.47E-11 | 6 | 2.85E-10 | 7.73E-11 | 3.16E-11 |
|  | KO old | 9 | 1.89E-10 | 2.85E-11 | 9.49E-12 | 7 | 1.58E-10 | 2.72E-11 | 1.03E-11 |
|  | ABHD12 KO | 15 | 1.86E-10 | 4.23E-11 | 1.09E-11 | 13 | 2.17E-10 | 8.48E-11 | 2.35E-11 |
|  | Young | 12 | 1.89E-10 | 5.19E-11 | 1.50E-11 | 12 | 2.74E-10 | 8.57E-11 | 2.47E-11 |
|  | Old | 15 | 1.95E-10 | 2.73E-11 | 7.05E-12 | 12 | 1.71E-10 | 3.71E-11 | 1.07E-11 |
| 2-arachidonoyl glycerol | WT young | 6 | 2.01E-09 | 6.46E-10 | 2.64E-10 | 6 | 4.18E-09 | 8.30E-10 | 3.39E-10 |
|  | WT old | 6 | 2.43E-09 | 2.36E-10 | 9.63E-11 | 5 | 3.97E-09 | 1.07E-09 | 4.80E-10 |
|  | WT | 12 | 2.22E-09 | 5.13E-10 | 1.48E-10 | 11 | 4.08E-09 | 9.05E-10 | 2.73E-10 |
|  | KO young | 6 | 2.04E-09 | 6.54E-10 | 2.67E-10 | 6 | 4.71E-09 | 1.24E-09 | 5.07E-10 |
|  | KO old | 9 | 2.23E-09 | 4.72E-10 | 1.57E-10 | 7 | 2.93E-09 | 5.42E-10 | 2.05E-10 |
|  | ABHD12 KO | 15 | 2.16E-09 | 5.37E-10 | 1.39E-10 | 13 | 3.75E-09 | 1.28E-09 | 3.54E-10 |
|  | Young | 12 | 2.03E-09 | 6.20E-10 | 1.79E-10 | 12 | 4.45E-09 | 1.04E-09 | 3.01E-10 |
|  | Old | 15 | 2.31E-09 | 3.96E-10 | 1.02E-10 | 12 | 3.36E-09 | 9.28E-10 | 2.68E-10 |

Supplemental Table 57: Mean levels of 2-acyl glycerols in the midbrain and brainstem

| Lipid Species | Group | Midbrain | | | | Brainstem | | | |
| --- | --- | --- | --- | --- | --- | --- | --- | --- | --- |
|  |  | N | Mean | SD | SE | N | Mean | SD | SE |
| 2-palmitoyl glycerol | WT young | 6 | 4.80E-09 | 1.24E-09 | 5.06E-10 | 6 | 1.87E-09 | 4.93E-10 | 2.01E-10 |
|  | WT old | 6 | 5.91E-09 | 8.54E-10 | 3.49E-10 | 6 | 2.57E-09 | 4.90E-10 | 2.00E-10 |
|  | WT | 12 | 5.35E-09 | 1.17E-09 | 3.37E-10 | 12 | 2.22E-09 | 5.94E-10 | 1.71E-10 |
|  | KO young | 6 | 4.23E-09 | 5.78E-10 | 2.36E-10 | 6 | 2.10E-09 | 6.64E-10 | 2.71E-10 |
|  | KO old | 9 | 4.52E-09 | 6.40E-10 | 2.13E-10 | 9 | 1.71E-09 | 3.74E-10 | 1.25E-10 |
|  | ABHD12 KO | 15 | 4.41E-09 | 6.12E-10 | 1.58E-10 | 15 | 1.87E-09 | 5.27E-10 | 1.36E-10 |
|  | Young | 12 | 4.52E-09 | 9.69E-10 | 2.80E-10 | 12 | 1.99E-09 | 5.70E-10 | 1.65E-10 |
|  | Old | 15 | 5.08E-09 | 9.93E-10 | 2.57E-10 | 15 | 2.05E-09 | 5.98E-10 | 1.55E-10 |
| 2-oleoyl glycerol | WT young | 6 | 8.14E-09 | 1.88E-09 | 7.67E-10 | 6 | 6.29E-09 | 1.26E-09 | 5.13E-10 |
|  | WT old | 6 | 1.05E-08 | 9.24E-10 | 3.77E-10 | 6 | 8.07E-09 | 6.76E-10 | 2.76E-10 |
|  | WT | 12 | 9.31E-09 | 1.87E-09 | 5.38E-10 | 12 | 7.18E-09 | 1.34E-09 | 3.86E-10 |
|  | KO young | 6 | 7.34E-09 | 3.34E-10 | 1.37E-10 | 6 | 6.92E-09 | 6.71E-10 | 2.74E-10 |
|  | KO old | 9 | 8.79E-09 | 6.09E-10 | 2.03E-10 | 9 | 6.85E-09 | 6.74E-10 | 2.25E-10 |
|  | ABHD12 KO | 15 | 8.21E-09 | 8.90E-10 | 2.30E-10 | 15 | 6.88E-09 | 6.49E-10 | 1.68E-10 |
|  | Young | 12 | 7.74E-09 | 1.35E-09 | 3.91E-10 | 12 | 6.60E-09 | 1.02E-09 | 2.93E-10 |
|  | Old | 15 | 9.46E-09 | 1.12E-09 | 2.88E-10 | 15 | 7.34E-09 | 8.97E-10 | 2.32E-10 |
| 2-linoleoyl glycerol | WT young | 6 | 3.98E-10 | 3.27E-11 | 1.33E-11 | 6 | 3.23E-10 | 2.51E-11 | 1.02E-11 |
|  | WT old | 6 | 3.78E-10 | 3.93E-11 | 1.60E-11 | 6 | 3.13E-10 | 2.39E-11 | 9.74E-12 |
|  | WT | 12 | 3.88E-10 | 3.59E-11 | 1.04E-11 | 12 | 3.18E-10 | 2.39E-11 | 6.89E-12 |
|  | KO young | 6 | 4.30E-10 | 4.11E-11 | 1.68E-11 | 6 | 3.95E-10 | 7.40E-11 | 3.02E-11 |
|  | KO old | 9 | 3.69E-10 | 4.42E-11 | 1.47E-11 | 9 | 2.94E-10 | 4.19E-11 | 1.40E-11 |
|  | ABHD12 KO | 15 | 3.93E-10 | 5.16E-11 | 1.33E-11 | 15 | 3.34E-10 | 7.46E-11 | 1.93E-11 |
|  | Young | 12 | 4.14E-10 | 3.91E-11 | 1.13E-11 | 12 | 3.59E-10 | 6.47E-11 | 1.87E-11 |
|  | Old | 15 | 3.73E-10 | 4.11E-11 | 1.06E-11 | 15 | 3.01E-10 | 3.61E-11 | 9.31E-12 |
| 2-arachidonoyl glycerol | WT young | 6 | 4.06E-09 | 1.80E-10 | 7.33E-11 | 6 | 3.68E-09 | 4.75E-10 | 1.94E-10 |
|  | WT old | 6 | 4.39E-09 | 2.60E-10 | 1.06E-10 | 6 | 4.08E-09 | 2.07E-10 | 8.44E-11 |
|  | WT | 12 | 4.23E-09 | 2.75E-10 | 7.94E-11 | 12 | 3.88E-09 | 4.07E-10 | 1.18E-10 |
|  | KO young | 6 | 5.41E-09 | 9.86E-10 | 4.03E-10 | 6 | 5.23E-09 | 9.28E-10 | 3.79E-10 |
|  | KO old | 9 | 5.54E-09 | 5.40E-10 | 1.80E-10 | 9 | 4.77E-09 | 5.70E-10 | 1.90E-10 |
|  | ABHD12 KO | 15 | 5.49E-09 | 7.20E-10 | 1.86E-10 | 15 | 4.95E-09 | 7.41E-10 | 1.91E-10 |
|  | Young | 12 | 4.73E-09 | 9.75E-10 | 2.82E-10 | 12 | 4.46E-09 | 1.07E-09 | 3.09E-10 |
|  | Old | 15 | 5.08E-09 | 7.28E-10 | 1.88E-10 | 15 | 4.49E-09 | 5.66E-10 | 1.46E-10 |

Supplemental Table 58: Mean levels of free fatty acids in the striatum and hippocampus

| Lipid Species | Group | Striatum | | | | Hippocampus | | | |
| --- | --- | --- | --- | --- | --- | --- | --- | --- | --- |
|  |  | N | Mean | SD | SE | N | Mean | SD | SE |
| Oleic acid | WT young | 6 | 3.71E-09 | 5.05E-10 | 2.06E-10 | 6 | 2.87E-09 | 3.43E-10 | 1.40E-10 |
|  | WT old | 6 | 3.08E-09 | 5.65E-10 | 2.30E-10 | 6 | 2.18E-09 | 2.16E-10 | 8.83E-11 |
|  | WT | 12 | 3.39E-09 | 6.07E-10 | 1.75E-10 | 12 | 2.53E-09 | 4.50E-10 | 1.30E-10 |
|  | KO young | 6 | 3.55E-09 | 7.05E-10 | 2.88E-10 | 6 | 2.99E-09 | 4.53E-10 | 1.85E-10 |
|  | KO old | 9 | 2.95E-09 | 4.47E-10 | 1.49E-10 | 9 | 2.12E-09 | 2.51E-10 | 8.36E-11 |
|  | ABHD12 KO | 15 | 3.19E-09 | 6.18E-10 | 1.60E-10 | 15 | 2.47E-09 | 5.51E-10 | 1.42E-10 |
|  | Young | 12 | 3.63E-09 | 5.91E-10 | 1.70E-10 | 12 | 2.93E-09 | 3.89E-10 | 1.12E-10 |
|  | Old | 15 | 3.00E-09 | 4.82E-10 | 1.24E-10 | 15 | 2.15E-09 | 2.32E-10 | 5.98E-11 |
| Linoleic acid | WT young | 6 | 1.38E-09 | 1.68E-10 | 6.86E-11 | 6 | 1.01E-09 | 9.88E-11 | 4.03E-11 |
|  | WT old | 6 | 1.18E-09 | 2.49E-10 | 1.02E-10 | 6 | 7.48E-10 | 6.88E-11 | 2.81E-11 |
|  | WT | 12 | 1.28E-09 | 2.28E-10 | 6.58E-11 | 12 | 8.80E-10 | 1.60E-10 | 4.62E-11 |
|  | KO young | 6 | 1.32E-09 | 2.05E-10 | 8.36E-11 | 6 | 1.05E-09 | 1.42E-10 | 5.79E-11 |
|  | KO old | 9 | 1.13E-09 | 2.05E-10 | 6.83E-11 | 9 | 7.50E-10 | 8.11E-11 | 2.70E-11 |
|  | ABHD12 KO | 15 | 1.20E-09 | 2.19E-10 | 5.65E-11 | 15 | 8.72E-10 | 1.86E-10 | 4.80E-11 |
|  | Young | 12 | 1.35E-09 | 1.82E-10 | 5.24E-11 | 12 | 1.03E-09 | 1.19E-10 | 3.42E-11 |
|  | Old | 15 | 1.15E-09 | 2.16E-10 | 5.59E-11 | 15 | 7.49E-10 | 7.38E-11 | 1.91E-11 |
| Arachidonic acid | WT young | 6 | 5.10E-09 | 4.62E-10 | 1.88E-10 | 6 | 4.37E-09 | 3.87E-10 | 1.58E-10 |
|  | WT old | 6 | 4.45E-09 | 7.77E-10 | 3.17E-10 | 6 | 3.36E-09 | 1.51E-10 | 6.15E-11 |
|  | WT | 12 | 4.78E-09 | 6.97E-10 | 2.01E-10 | 12 | 3.86E-09 | 6.00E-10 | 1.73E-10 |
|  | KO young | 6 | 5.40E-09 | 7.95E-10 | 3.24E-10 | 6 | 4.88E-09 | 5.50E-10 | 2.25E-10 |
|  | KO old | 9 | 4.49E-09 | 6.30E-10 | 2.10E-10 | 9 | 3.64E-09 | 4.53E-10 | 1.51E-10 |
|  | ABHD12 KO | 15 | 4.86E-09 | 8.14E-10 | 2.10E-10 | 15 | 4.13E-09 | 7.88E-10 | 2.04E-10 |
|  | Young | 12 | 5.25E-09 | 6.39E-10 | 1.84E-10 | 12 | 4.63E-09 | 5.25E-10 | 1.51E-10 |
|  | Old | 15 | 4.48E-09 | 6.65E-10 | 1.72E-10 | 15 | 3.52E-09 | 3.82E-10 | 9.86E-11 |

Supplemental Table 59: Mean levels of free fatty acids in the cerebellum and thalamus

| Lipid Species | Group | Cerebellum | | | | Thalamus | | | |
| --- | --- | --- | --- | --- | --- | --- | --- | --- | --- |
|  |  | N | Mean | SD | SE | N | Mean | SD | SE |
| Oleic acid | WT young | 6 | 2.04E-09 | 1.90E-10 | 7.75E-11 | 6 | 2.83E-09 | 4.36E-10 | 1.78E-10 |
|  | WT old | 6 | 1.99E-09 | 1.08E-10 | 4.40E-11 | 6 | 2.04E-09 | 2.70E-10 | 1.10E-10 |
|  | WT | 12 | 2.01E-09 | 1.49E-10 | 4.30E-11 | 12 | 2.43E-09 | 5.35E-10 | 1.54E-10 |
|  | KO young | 6 | 2.05E-09 | 2.22E-10 | 9.07E-11 | 6 | 3.17E-09 | 7.65E-10 | 3.12E-10 |
|  | KO old | 9 | 1.96E-09 | 8.48E-11 | 2.83E-11 | 9 | 2.10E-09 | 3.55E-10 | 1.18E-10 |
|  | ABHD12 KO | 15 | 2.00E-09 | 1.54E-10 | 3.98E-11 | 15 | 2.53E-09 | 7.56E-10 | 1.95E-10 |
|  | Young | 12 | 2.04E-09 | 1.97E-10 | 5.69E-11 | 12 | 3.00E-09 | 6.19E-10 | 1.79E-10 |
|  | Old | 15 | 1.98E-09 | 9.21E-11 | 2.38E-11 | 15 | 2.08E-09 | 3.15E-10 | 8.13E-11 |
| Linoleic acid | WT young | 6 | 9.13E-10 | 8.66E-11 | 3.53E-11 | 6 | 1.02E-09 | 1.08E-10 | 4.43E-11 |
|  | WT old | 6 | 8.17E-10 | 7.00E-11 | 2.86E-11 | 6 | 6.97E-10 | 7.00E-11 | 2.86E-11 |
|  | WT | 12 | 8.65E-10 | 9.03E-11 | 2.61E-11 | 12 | 8.56E-10 | 1.88E-10 | 5.42E-11 |
|  | KO young | 6 | 9.44E-10 | 1.45E-10 | 5.92E-11 | 6 | 1.13E-09 | 2.37E-10 | 9.66E-11 |
|  | KO old | 9 | 8.46E-10 | 8.92E-11 | 2.97E-11 | 9 | 7.68E-10 | 1.55E-10 | 5.17E-11 |
|  | ABHD12 KO | 15 | 8.85E-10 | 1.21E-10 | 3.11E-11 | 15 | 9.14E-10 | 2.61E-10 | 6.74E-11 |
|  | Young | 12 | 9.29E-10 | 1.15E-10 | 3.32E-11 | 12 | 1.07E-09 | 1.86E-10 | 5.37E-11 |
|  | Old | 15 | 8.34E-10 | 8.07E-11 | 2.08E-11 | 15 | 7.39E-10 | 1.30E-10 | 3.35E-11 |
| Arachidonic acid | WT young | 6 | 2.93E-09 | 2.35E-10 | 9.61E-11 | 6 | 3.22E-09 | 4.08E-10 | 1.67E-10 |
|  | WT old | 6 | 2.73E-09 | 1.72E-10 | 7.01E-11 | 6 | 2.38E-09 | 2.11E-10 | 8.61E-11 |
|  | WT | 12 | 2.83E-09 | 2.21E-10 | 6.37E-11 | 12 | 2.80E-09 | 5.38E-10 | 1.55E-10 |
|  | KO young | 6 | 3.37E-09 | 4.49E-10 | 1.83E-10 | 6 | 3.89E-09 | 7.12E-10 | 2.91E-10 |
|  | KO old | 9 | 3.06E-09 | 1.64E-10 | 5.46E-11 | 9 | 2.78E-09 | 4.67E-10 | 1.56E-10 |
|  | ABHD12 KO | 15 | 3.18E-09 | 3.36E-10 | 8.69E-11 | 15 | 3.22E-09 | 7.87E-10 | 2.03E-10 |
|  | Young | 12 | 3.15E-09 | 4.14E-10 | 1.20E-10 | 12 | 3.55E-09 | 6.53E-10 | 1.88E-10 |
|  | Old | 15 | 2.93E-09 | 2.29E-10 | 5.92E-11 | 15 | 2.62E-09 | 4.26E-10 | 1.10E-10 |

Supplemental Table 60: Mean levels of free fatty acids in the cortex and hypothalamus

| Lipid Species | Group | Cortex | | | | Hypothalamus | | | |
| --- | --- | --- | --- | --- | --- | --- | --- | --- | --- |
|  |  | N | Mean | SD | SE | N | Mean | SD | SE |
| Oleic acid | WT young | 6 | 1.28E-09 | 1.24E-10 | 5.05E-11 | 6 | 2.50E-09 | 5.39E-10 | 2.20E-10 |
|  | WT old | 6 | 1.23E-09 | 6.30E-11 | 2.57E-11 | 5 | 2.11E-09 | 3.32E-10 | 1.49E-10 |
|  | WT | 12 | 1.25E-09 | 9.76E-11 | 2.82E-11 | 11 | 2.32E-09 | 4.81E-10 | 1.45E-10 |
|  | KO young | 6 | 1.41E-09 | 1.78E-10 | 7.28E-11 | 6 | 2.17E-09 | 3.47E-10 | 1.42E-10 |
|  | KO old | 9 | 1.28E-09 | 1.13E-10 | 3.77E-11 | 7 | 1.81E-09 | 2.13E-10 | 8.07E-11 |
|  | ABHD12 KO | 15 | 1.33E-09 | 1.53E-10 | 3.94E-11 | 13 | 1.98E-09 | 3.30E-10 | 9.15E-11 |
|  | Young | 12 | 1.35E-09 | 1.61E-10 | 4.65E-11 | 12 | 2.34E-09 | 4.65E-10 | 1.34E-10 |
|  | Old | 15 | 1.26E-09 | 9.65E-11 | 2.49E-11 | 12 | 1.93E-09 | 2.98E-10 | 8.61E-11 |
| Linoleic acid | WT young | 6 | 4.44E-10 | 5.55E-11 | 2.27E-11 | 6 | 1.12E-09 | 1.36E-10 | 5.54E-11 |
|  | WT old | 6 | 3.72E-10 | 2.90E-11 | 1.18E-11 | 5 | 9.50E-10 | 1.68E-10 | 7.53E-11 |
|  | WT | 12 | 4.08E-10 | 5.67E-11 | 1.64E-11 | 11 | 1.04E-09 | 1.69E-10 | 5.11E-11 |
|  | KO young | 6 | 4.45E-10 | 7.74E-11 | 3.16E-11 | 6 | 1.12E-09 | 1.47E-10 | 5.99E-11 |
|  | KO old | 9 | 4.01E-10 | 3.68E-11 | 1.23E-11 | 7 | 8.15E-10 | 8.68E-11 | 3.28E-11 |
|  | ABHD12 KO | 15 | 4.18E-10 | 5.84E-11 | 1.51E-11 | 13 | 9.58E-10 | 1.96E-10 | 5.44E-11 |
|  | Young | 12 | 4.44E-10 | 6.42E-11 | 1.85E-11 | 12 | 1.12E-09 | 1.35E-10 | 3.89E-11 |
|  | Old | 15 | 3.89E-10 | 3.59E-11 | 9.26E-12 | 12 | 8.72E-10 | 1.39E-10 | 4.01E-11 |
| Arachidonic acid | WT young | 6 | 1.91E-09 | 1.86E-10 | 7.61E-11 | 6 | 3.87E-09 | 6.18E-10 | 2.52E-10 |
|  | WT old | 6 | 1.77E-09 | 4.60E-11 | 1.88E-11 | 5 | 3.39E-09 | 6.98E-10 | 3.12E-10 |
|  | WT | 12 | 1.84E-09 | 1.50E-10 | 4.32E-11 | 11 | 3.65E-09 | 6.69E-10 | 2.02E-10 |
|  | KO young | 6 | 2.11E-09 | 2.25E-10 | 9.18E-11 | 6 | 3.66E-09 | 5.27E-10 | 2.15E-10 |
|  | KO old | 9 | 2.01E-09 | 1.37E-10 | 4.57E-11 | 7 | 3.10E-09 | 2.44E-10 | 9.20E-11 |
|  | ABHD12 KO | 15 | 2.05E-09 | 1.78E-10 | 4.60E-11 | 13 | 3.36E-09 | 4.78E-10 | 1.33E-10 |
|  | Young | 12 | 2.01E-09 | 2.23E-10 | 6.44E-11 | 12 | 3.76E-09 | 5.58E-10 | 1.61E-10 |
|  | Old | 15 | 1.91E-09 | 1.61E-10 | 4.17E-11 | 12 | 3.22E-09 | 4.81E-10 | 1.39E-10 |

Supplemental Table 61: Mean levels of free fatty acids in the midbrain and brainstem

| Lipid Species | Group | Midbrain | | | | Brainstem | | | |
| --- | --- | --- | --- | --- | --- | --- | --- | --- | --- |
|  |  | N | Mean | SD | SE | N | Mean | SD | SE |
| Oleic acid | WT young | 6 | 1.93E-09 | 1.18E-10 | 4.82E-11 | 6 | 1.76E-09 | 3.65E-10 | 1.49E-10 |
|  | WT old | 6 | 1.63E-09 | 2.09E-10 | 8.55E-11 | 6 | 1.38E-09 | 1.50E-10 | 6.11E-11 |
|  | WT | 12 | 1.78E-09 | 2.23E-10 | 6.44E-11 | 12 | 1.57E-09 | 3.32E-10 | 9.59E-11 |
|  | KO young | 6 | 2.03E-09 | 3.07E-10 | 1.25E-10 | 6 | 1.77E-09 | 5.32E-10 | 2.17E-10 |
|  | KO old | 9 | 1.61E-09 | 1.82E-10 | 6.08E-11 | 9 | 1.27E-09 | 1.08E-10 | 3.59E-11 |
|  | ABHD12 KO | 15 | 1.78E-09 | 3.11E-10 | 8.03E-11 | 15 | 1.47E-09 | 4.14E-10 | 1.07E-10 |
|  | Young | 12 | 1.98E-09 | 2.28E-10 | 6.58E-11 | 12 | 1.76E-09 | 4.35E-10 | 1.26E-10 |
|  | Old | 15 | 1.62E-09 | 1.87E-10 | 4.82E-11 | 15 | 1.31E-09 | 1.31E-10 | 3.39E-11 |
| Linoleic acid | WT young | 6 | 6.64E-10 | 4.76E-11 | 1.94E-11 | 6 | 6.28E-10 | 1.03E-10 | 4.21E-11 |
|  | WT old | 6 | 4.83E-10 | 5.30E-11 | 2.16E-11 | 6 | 4.48E-10 | 4.90E-11 | 2.00E-11 |
|  | WT | 12 | 5.73E-10 | 1.06E-10 | 3.06E-11 | 12 | 5.38E-10 | 1.22E-10 | 3.51E-11 |
|  | KO young | 6 | 7.13E-10 | 1.06E-10 | 4.33E-11 | 6 | 6.66E-10 | 1.56E-10 | 6.36E-11 |
|  | KO old | 9 | 5.58E-10 | 4.26E-11 | 1.42E-11 | 9 | 4.41E-10 | 3.73E-11 | 1.24E-11 |
|  | ABHD12 KO | 15 | 6.20E-10 | 1.06E-10 | 2.74E-11 | 15 | 5.31E-10 | 1.50E-10 | 3.86E-11 |
|  | Young | 12 | 6.89E-10 | 8.26E-11 | 2.38E-11 | 12 | 6.47E-10 | 1.27E-10 | 3.68E-11 |
|  | Old | 15 | 5.28E-10 | 5.91E-11 | 1.53E-11 | 15 | 4.44E-10 | 4.08E-11 | 1.05E-11 |
| Arachidonic acid | WT young | 6 | 2.20E-09 | 6.71E-11 | 2.74E-11 | 6 | 1.63E-09 | 1.91E-10 | 7.81E-11 |
|  | WT old | 6 | 1.93E-09 | 1.39E-10 | 5.66E-11 | 6 | 1.31E-09 | 7.13E-11 | 2.91E-11 |
|  | WT | 12 | 2.07E-09 | 1.75E-10 | 5.06E-11 | 12 | 1.47E-09 | 2.17E-10 | 6.28E-11 |
|  | KO young | 6 | 2.58E-09 | 3.83E-10 | 1.56E-10 | 6 | 2.09E-09 | 5.29E-10 | 2.16E-10 |
|  | KO old | 9 | 2.24E-09 | 2.03E-10 | 6.78E-11 | 9 | 1.52E-09 | 9.04E-11 | 3.01E-11 |
|  | ABHD12 KO | 15 | 2.37E-09 | 3.26E-10 | 8.41E-11 | 15 | 1.75E-09 | 4.36E-10 | 1.12E-10 |
|  | Young | 12 | 2.39E-09 | 3.27E-10 | 9.44E-11 | 12 | 1.86E-09 | 4.49E-10 | 1.30E-10 |
|  | Old | 15 | 2.11E-09 | 2.33E-10 | 6.01E-11 | 15 | 1.43E-09 | 1.33E-10 | 3.43E-11 |

Supplemental Table 62: Mean levels of phosphoLEA in the striatum and hippocampus

| Lipid Species | Group | Striatum | | | | Hippocampus | | | |
| --- | --- | --- | --- | --- | --- | --- | --- | --- | --- |
|  |  | N | Mean | SD | SE | N | Mean | SD | SE |
| phosphoLEA | WT young | 6 | 2.92E-11 | 9.40E-12 | 3.84E-12 | 6 | 1.22E-11 | 2.92E-12 | 1.19E-12 |
|  | WT old | 6 | 2.92E-11 | 9.92E-12 | 4.05E-12 | 6 | 1.15E-11 | 1.19E-12 | 4.88E-13 |
|  | WT | 12 | 2.92E-11 | 9.21E-12 | 2.66E-12 | 12 | 1.19E-11 | 2.15E-12 | 6.21E-13 |
|  | KO young | 6 | 3.27E-11 | 1.36E-11 | 5.53E-12 | 6 | 1.36E-11 | 4.45E-12 | 1.82E-12 |
|  | KO old | 9 | 3.10E-11 | 6.99E-12 | 2.33E-12 | 9 | 1.38E-11 | 4.78E-12 | 1.59E-12 |
|  | ABHD12 KO | 15 | 3.17E-11 | 9.71E-12 | 2.51E-12 | 15 | 1.38E-11 | 4.49E-12 | 1.16E-12 |
|  | Young | 12 | 3.10E-11 | 1.13E-11 | 3.25E-12 | 12 | 1.29E-11 | 3.67E-12 | 1.06E-12 |
|  | Old | 15 | 3.03E-11 | 7.99E-12 | 2.06E-12 | 15 | 1.29E-11 | 3.86E-12 | 9.98E-13 |

Supplemental Table 63: Mean levels of phosphoLEA in the cerebellum and thalamus

| Lipid Species | Group | Cerebellum | | | | Thalamus | | | |
| --- | --- | --- | --- | --- | --- | --- | --- | --- | --- |
|  |  | N | Mean | SD | SE | N | Mean | SD | SE |
| phosphoLEA | WT young | 6 | 2.68E-11 | 5.35E-12 | 2.18E-12 | 6 | 2.59E-11 | 2.04E-11 | 8.33E-12 |
|  | WT old | 6 | 3.38E-11 | 4.96E-12 | 2.02E-12 | 6 | 3.04E-11 | 2.43E-11 | 9.91E-12 |
|  | WT | 12 | 3.03E-11 | 6.13E-12 | 1.77E-12 | 12 | 2.81E-11 | 2.15E-11 | 6.21E-12 |
|  | KO young | 6 | 2.78E-11 | 5.07E-12 | 2.07E-12 | 6 | 2.66E-11 | 1.49E-11 | 6.08E-12 |
|  | KO old | 9 | 3.48E-11 | 9.02E-12 | 3.01E-12 | 9 | 2.81E-11 | 2.02E-11 | 6.74E-12 |
|  | ABHD12 KO | 15 | 3.20E-11 | 8.26E-12 | 2.13E-12 | 15 | 2.75E-11 | 1.77E-11 | 4.57E-12 |
|  | Young | 12 | 2.73E-11 | 5.00E-12 | 1.44E-12 | 12 | 2.62E-11 | 1.70E-11 | 4.92E-12 |
|  | Old | 15 | 3.44E-11 | 7.45E-12 | 1.92E-12 | 15 | 2.91E-11 | 2.11E-11 | 5.45E-12 |

Supplemental Table 64: Mean levels of phosphoLEA in the cortex and hypothalamus

| Lipid Species | Group | Cortex | | | | Hypothalamus | | | |
| --- | --- | --- | --- | --- | --- | --- | --- | --- | --- |
|  |  | N | Mean | SD | SE | N | Mean | SD | SE |
| phosphoLEA | WT young | 6 | 4.08E-11 | 1.11E-11 | 4.53E-12 | 6 | 3.06E-11 | 1.65E-11 | 6.74E-12 |
|  | WT old | 6 | 4.25E-11 | 1.25E-11 | 5.08E-12 | 5 | 3.17E-11 | 1.05E-11 | 4.71E-12 |
|  | WT | 12 | 4.17E-11 | 1.13E-11 | 3.26E-12 | 11 | 3.11E-11 | 1.35E-11 | 4.06E-12 |
|  | KO young | 6 | 4.51E-11 | 1.14E-11 | 4.66E-12 | 6 | 2.77E-11 | 1.39E-11 | 5.67E-12 |
|  | KO old | 9 | 5.23E-11 | 1.28E-11 | 4.28E-12 | 7 | 3.20E-11 | 6.36E-12 | 2.40E-12 |
|  | ABHD12 KO | 15 | 4.95E-11 | 1.24E-11 | 3.21E-12 | 13 | 3.00E-11 | 1.03E-11 | 2.85E-12 |
|  | Young | 12 | 4.30E-11 | 1.10E-11 | 3.17E-12 | 12 | 2.91E-11 | 1.46E-11 | 4.22E-12 |
|  | Old | 15 | 4.84E-11 | 1.32E-11 | 3.41E-12 | 12 | 3.18E-11 | 7.90E-12 | 2.28E-12 |

Supplemental Table 65: Mean levels of phosphoLEA in the midbrain and brainstem

| Lipid Species | Group | Midbrain | | | | Brainstem | | | |
| --- | --- | --- | --- | --- | --- | --- | --- | --- | --- |
|  |  | N | Mean | SD | SE | N | Mean | SD | SE |
| phosphoLEA | WT young | 6 | 1.01E-11 | 1.07E-12 | 4.37E-13 | 6 | 1.37E-11 | 3.50E-12 | 1.43E-12 |
|  | WT old | 6 | 1.18E-11 | 1.57E-12 | 6.41E-13 | 6 | 1.77E-11 | 1.85E-12 | 7.57E-13 |
|  | WT | 12 | 1.09E-11 | 1.57E-12 | 4.52E-13 | 12 | 1.57E-11 | 3.39E-12 | 9.79E-13 |
|  | KO young | 6 | 1.51E-11 | 2.21E-12 | 9.02E-13 | 6 | 2.17E-11 | 2.87E-12 | 1.17E-12 |
|  | KO old | 9 | 1.52E-11 | 2.95E-12 | 9.82E-13 | 9 | 2.10E-11 | 4.62E-12 | 1.54E-12 |
|  | ABHD12 KO | 15 | 1.51E-11 | 2.59E-12 | 6.69E-13 | 15 | 2.13E-11 | 3.90E-12 | 1.01E-12 |
|  | Young | 12 | 1.26E-11 | 3.08E-12 | 8.88E-13 | 12 | 1.77E-11 | 5.17E-12 | 1.49E-12 |
|  | Old | 15 | 1.38E-11 | 2.96E-12 | 7.63E-13 | 15 | 1.97E-11 | 4.03E-12 | 1.04E-12 |

Supplemental Table 66: Mean levels of prostaglandins in the striatum and hippocampus

| Lipid Species | Group | Striatum | | | | Hippocampus | | | |
| --- | --- | --- | --- | --- | --- | --- | --- | --- | --- |
|  |  | N | Mean | SD | SE | N | Mean | SD | SE |
| PGE_2_ | WT young | 6 | 8.99E-10 | 3.58E-11 | 1.46E-11 | 6 | 1.47E-09 | 2.75E-10 | 1.12E-10 |
|  | WT old | 6 | 8.77E-10 | 8.67E-11 | 3.54E-11 | 6 | 1.63E-09 | 3.25E-10 | 1.33E-10 |
|  | WT | 12 | 8.88E-10 | 6.43E-11 | 1.86E-11 | 12 | 1.55E-09 | 2.98E-10 | 8.61E-11 |
|  | KO young | 6 | 1.11E-09 | 1.86E-10 | 7.58E-11 | 6 | 1.78E-09 | 3.26E-10 | 1.33E-10 |
|  | KO old | 9 | 1.09E-09 | 1.16E-10 | 3.85E-11 | 9 | 1.61E-09 | 4.28E-10 | 1.43E-10 |
|  | ABHD12 KO | 15 | 1.10E-09 | 1.42E-10 | 3.66E-11 | 15 | 1.68E-09 | 3.87E-10 | 9.98E-11 |
|  | Young | 12 | 1.01E-09 | 1.70E-10 | 4.91E-11 | 12 | 1.63E-09 | 3.28E-10 | 9.48E-11 |
|  | Old | 15 | 1.01E-09 | 1.50E-10 | 3.87E-11 | 15 | 1.62E-09 | 3.77E-10 | 9.75E-11 |
| PGF_2α_ | WT young | 6 | 7.31E-10 | 7.96E-11 | 3.25E-11 | 6 | 9.69E-10 | 8.73E-11 | 3.56E-11 |
|  | WT old | 6 | 6.67E-10 | 1.05E-10 | 4.28E-11 | 6 | 1.21E-09 | 2.66E-10 | 1.08E-10 |
|  | WT | 12 | 6.99E-10 | 9.48E-11 | 2.74E-11 | 12 | 1.09E-09 | 2.27E-10 | 6.55E-11 |
|  | KO young | 6 | 9.77E-10 | 4.96E-11 | 2.02E-11 | 6 | 1.38E-09 | 1.56E-10 | 6.36E-11 |
|  | KO old | 9 | 8.70E-10 | 9.98E-11 | 3.33E-11 | 9 | 1.18E-09 | 2.60E-10 | 8.67E-11 |
|  | ABHD12 KO | 15 | 9.13E-10 | 9.75E-11 | 2.52E-11 | 15 | 1.26E-09 | 2.39E-10 | 6.18E-11 |
|  | Young | 12 | 8.54E-10 | 1.43E-10 | 4.14E-11 | 12 | 1.17E-09 | 2.45E-10 | 7.07E-11 |
|  | Old | 15 | 7.89E-10 | 1.42E-10 | 3.67E-11 | 15 | 1.19E-09 | 2.53E-10 | 6.54E-11 |
| 6-ketoPGF_1α_ | WT young | 6 | 5.05E-12 | 7.53E-13 | 3.08E-13 | 6 | 1.38E-11 | 2.51E-12 | 1.02E-12 |
|  | WT old | 6 | 6.91E-12 | 1.77E-12 | 7.23E-13 | 6 | 1.11E-11 | 2.60E-12 | 1.06E-12 |
|  | WT | 12 | 5.98E-12 | 1.62E-12 | 4.67E-13 | 12 | 1.24E-11 | 2.80E-12 | 8.07E-13 |
|  | KO young | 6 | 7.41E-12 | 9.22E-13 | 3.77E-13 | 6 | 3.12E-11 | 7.35E-12 | 3.00E-12 |
|  | KO old | 9 | 1.13E-11 | 2.51E-12 | 8.37E-13 | 9 | 2.18E-11 | 7.41E-12 | 2.47E-12 |
|  | ABHD12 KO | 15 | 9.74E-12 | 2.79E-12 | 7.21E-13 | 15 | 2.55E-11 | 8.58E-12 | 2.22E-12 |
|  | Young | 12 | 6.23E-12 | 1.47E-12 | 4.24E-13 | 12 | 2.25E-11 | 1.05E-11 | 3.03E-12 |
|  | Old | 15 | 9.54E-12 | 3.11E-12 | 8.03E-13 | 15 | 1.75E-11 | 7.93E-12 | 2.05E-12 |

Supplemental Table 67: Mean levels of prostaglandins in the cerebellum and thalamus

| Lipid Species | Group | Cerebellum | | | | Thalamus | | | |
| --- | --- | --- | --- | --- | --- | --- | --- | --- | --- |
|  |  | N | Mean | SD | SE | N | Mean | SD | SE |
| PGE_2_ | WT young | 6 | 8.44E-10 | 1.13E-10 | 4.63E-11 | 6 | 6.83E-10 | 5.14E-11 | 2.10E-11 |
|  | WT old | 6 | 7.55E-10 | 7.65E-11 | 3.12E-11 | 6 | 6.45E-10 | 6.82E-11 | 2.78E-11 |
|  | WT | 12 | 7.99E-10 | 1.03E-10 | 2.98E-11 | 12 | 6.64E-10 | 6.08E-11 | 1.76E-11 |
|  | KO young | 6 | 1.03E-09 | 1.49E-10 | 6.07E-11 | 6 | 9.35E-10 | 1.33E-10 | 5.42E-11 |
|  | KO old | 9 | 1.05E-09 | 1.73E-10 | 5.77E-11 | 9 | 9.47E-10 | 1.51E-10 | 5.02E-11 |
|  | ABHD12 KO | 15 | 1.04E-09 | 1.59E-10 | 4.10E-11 | 15 | 9.42E-10 | 1.39E-10 | 3.59E-11 |
|  | Young | 12 | 9.37E-10 | 1.59E-10 | 4.59E-11 | 12 | 8.09E-10 | 1.63E-10 | 4.70E-11 |
|  | Old | 15 | 9.34E-10 | 2.05E-10 | 5.30E-11 | 15 | 8.26E-10 | 1.95E-10 | 5.04E-11 |
| PGF_2α_ | WT young | 6 | 6.86E-10 | 7.60E-11 | 3.10E-11 | 6 | 6.76E-10 | 5.17E-11 | 2.11E-11 |
|  | WT old | 6 | 6.45E-10 | 1.25E-10 | 5.11E-11 | 6 | 6.39E-10 | 8.93E-11 | 3.65E-11 |
|  | WT | 12 | 6.66E-10 | 1.01E-10 | 2.92E-11 | 12 | 6.58E-10 | 7.22E-11 | 2.08E-11 |
|  | KO young | 6 | 9.03E-10 | 1.52E-10 | 6.21E-11 | 6 | 9.61E-10 | 1.08E-10 | 4.43E-11 |
|  | KO old | 9 | 9.46E-10 | 1.30E-10 | 4.34E-11 | 9 | 9.26E-10 | 7.78E-11 | 2.59E-11 |
|  | ABHD12 KO | 15 | 9.29E-10 | 1.36E-10 | 3.50E-11 | 15 | 9.40E-10 | 8.94E-11 | 2.31E-11 |
|  | Young | 12 | 7.94E-10 | 1.61E-10 | 4.65E-11 | 12 | 8.19E-10 | 1.70E-10 | 4.90E-11 |
|  | Old | 15 | 8.26E-10 | 1.96E-10 | 5.07E-11 | 15 | 8.11E-10 | 1.66E-10 | 4.27E-11 |
| 6-ketoPGF_1α_ | WT young | 6 | 1.46E-11 | 5.59E-12 | 2.28E-12 | 6 | 8.13E-12 | 9.64E-13 | 3.93E-13 |
|  | WT old | 6 | 1.43E-11 | 3.03E-12 | 1.24E-12 | 6 | 8.01E-12 | 9.25E-13 | 3.77E-13 |
|  | WT | 12 | 1.45E-11 | 4.29E-12 | 1.24E-12 | 12 | 8.07E-12 | 9.02E-13 | 2.61E-13 |
|  | KO young | 6 | 2.52E-11 | 5.16E-12 | 2.11E-12 | 6 | 1.92E-11 | 2.61E-12 | 1.06E-12 |
|  | KO old | 9 | 2.14E-11 | 4.67E-12 | 1.56E-12 | 9 | 1.64E-11 | 3.81E-12 | 1.27E-12 |
|  | ABHD12 KO | 15 | 2.29E-11 | 5.07E-12 | 1.31E-12 | 15 | 1.75E-11 | 3.55E-12 | 9.17E-13 |
|  | Young | 12 | 1.99E-11 | 7.52E-12 | 2.17E-12 | 12 | 1.36E-11 | 6.06E-12 | 1.75E-12 |
|  | Old | 15 | 1.85E-11 | 5.35E-12 | 1.38E-12 | 15 | 1.31E-11 | 5.18E-12 | 1.34E-12 |

Supplemental Table 68: Mean levels of prostaglandins in the cortex and hypothalamus

| Lipid Species | Group | Cortex | | | | Hypothalamus | | | |
| --- | --- | --- | --- | --- | --- | --- | --- | --- | --- |
|  |  | N | Mean | SD | SE | N | Mean | SD | SE |
| PGE_2_ | WT young | 6 | 1.20E-09 | 1.74E-10 | 7.10E-11 | 6 | 4.50E-10 | 6.43E-11 | 2.62E-11 |
|  | WT old | 6 | 1.06E-09 | 1.69E-10 | 6.92E-11 | 5 | 3.87E-10 | 6.59E-11 | 2.94E-11 |
|  | WT | 12 | 1.13E-09 | 1.78E-10 | 5.13E-11 | 11 | 4.21E-10 | 7.00E-11 | 2.11E-11 |
|  | KO young | 6 | 1.55E-09 | 1.53E-10 | 6.26E-11 | 6 | 4.96E-10 | 1.15E-10 | 4.71E-11 |
|  | KO old | 9 | 1.48E-09 | 1.82E-10 | 6.07E-11 | 7 | 5.49E-10 | 6.06E-11 | 2.29E-11 |
|  | ABHD12 KO | 15 | 1.51E-09 | 1.69E-10 | 4.37E-11 | 13 | 5.25E-10 | 9.02E-11 | 2.50E-11 |
|  | Young | 12 | 1.37E-09 | 2.42E-10 | 6.99E-11 | 12 | 4.73E-10 | 9.23E-11 | 2.66E-11 |
|  | Old | 15 | 1.31E-09 | 2.71E-10 | 7.01E-11 | 12 | 4.82E-10 | 1.03E-10 | 2.97E-11 |
| PGF_2α_ | WT young | 6 | 6.77E-10 | 1.04E-10 | 4.26E-11 | 6 | 4.85E-10 | 9.93E-11 | 4.06E-11 |
|  | WT old | 6 | 6.31E-10 | 3.97E-11 | 1.62E-11 | 5 | 4.30E-10 | 1.13E-10 | 5.05E-11 |
|  | WT | 12 | 6.54E-10 | 7.89E-11 | 2.28E-11 | 11 | 4.60E-10 | 1.04E-10 | 3.14E-11 |
|  | KO young | 6 | 9.11E-10 | 1.21E-10 | 4.93E-11 | 6 | 5.42E-10 | 8.50E-11 | 3.47E-11 |
|  | KO old | 9 | 8.59E-10 | 7.78E-11 | 2.59E-11 | 7 | 5.97E-10 | 8.92E-11 | 3.37E-11 |
|  | ABHD12 KO | 15 | 8.80E-10 | 9.68E-11 | 2.50E-11 | 13 | 5.72E-10 | 8.82E-11 | 2.45E-11 |
|  | Young | 12 | 7.94E-10 | 1.63E-10 | 4.70E-11 | 12 | 5.14E-10 | 9.31E-11 | 2.69E-11 |
|  | Old | 15 | 7.68E-10 | 1.32E-10 | 3.40E-11 | 12 | 5.27E-10 | 1.28E-10 | 3.69E-11 |
| 6-ketoPGF_1α_ | WT young | 6 | 6.25E-12 | 1.39E-12 | 5.69E-13 | 6 | PISSR |  |  |
|  | WT old | 6 | 4.42E-12 | 6.75E-13 | 2.76E-13 | 5 | PISSR |  |  |
|  | WT | 12 | 5.33E-12 | 1.42E-12 | 4.09E-13 | 11 | PISSR |  |  |
|  | KO young | 6 | 9.73E-12 | 2.18E-12 | 8.91E-13 | 6 | PISSR |  |  |
|  | KO old | 9 | 8.03E-12 | 1.59E-12 | 5.31E-13 | 7 | PISSR |  |  |
|  | ABHD12 KO | 15 | 8.71E-12 | 1.97E-12 | 5.09E-13 | 13 | PISSR |  |  |
|  | Young | 12 | 7.99E-12 | 2.52E-12 | 7.28E-13 | 12 | PISSR |  |  |
|  | Old | 15 | 6.59E-12 | 2.23E-12 | 5.76E-13 | 12 | PISSR |  |  |

Supplemental Table 69: Mean levels of prostaglandins in the midbrain and brainstem

| Lipid Species | Group | Midbrain | | | | Brainstem | | | |
| --- | --- | --- | --- | --- | --- | --- | --- | --- | --- |
|  |  | N | Mean | SD | SE | N | Mean | SD | SE |
| PGE_2_ | WT young | 6 | 8.76E-10 | 8.71E-11 | 3.56E-11 | 6 | 5.92E-10 | 8.38E-11 | 3.42E-11 |
|  | WT old | 6 | 7.32E-10 | 8.94E-11 | 3.65E-11 | 6 | 5.86E-10 | 8.55E-11 | 3.49E-11 |
|  | WT | 12 | 8.04E-10 | 1.13E-10 | 3.26E-11 | 12 | 5.89E-10 | 8.08E-11 | 2.33E-11 |
|  | KO young | 6 | 1.32E-09 | 3.73E-10 | 1.52E-10 | 6 | 8.31E-10 | 8.14E-11 | 3.32E-11 |
|  | KO old | 9 | 1.06E-09 | 1.78E-10 | 5.94E-11 | 9 | 8.66E-10 | 9.70E-11 | 3.23E-11 |
|  | ABHD12 KO | 15 | 1.17E-09 | 2.91E-10 | 7.51E-11 | 15 | 8.52E-10 | 8.97E-11 | 2.32E-11 |
|  | Young | 12 | 1.10E-09 | 3.47E-10 | 1.00E-10 | 12 | 7.12E-10 | 1.48E-10 | 4.26E-11 |
|  | Old | 15 | 9.32E-10 | 2.23E-10 | 5.75E-11 | 15 | 7.54E-10 | 1.68E-10 | 4.33E-11 |
| PGF_2α_ | WT young | 6 | 9.44E-10 | 6.89E-11 | 2.81E-11 | 6 | 3.87E-10 | 5.98E-11 | 2.44E-11 |
|  | WT old | 6 | 8.22E-10 | 9.89E-11 | 4.04E-11 | 6 | 4.38E-10 | 4.88E-11 | 1.99E-11 |
|  | WT | 12 | 8.83E-10 | 1.03E-10 | 2.98E-11 | 12 | 4.13E-10 | 5.84E-11 | 1.68E-11 |
|  | KO young | 6 | 1.51E-09 | 2.11E-10 | 8.60E-11 | 6 | 5.83E-10 | 7.95E-11 | 3.25E-11 |
|  | KO old | 9 | 1.23E-09 | 9.74E-11 | 3.25E-11 | 9 | 5.67E-10 | 5.93E-11 | 1.98E-11 |
|  | ABHD12 KO | 15 | 1.34E-09 | 2.05E-10 | 5.30E-11 | 15 | 5.74E-10 | 6.58E-11 | 1.70E-11 |
|  | Young | 12 | 1.23E-09 | 3.32E-10 | 9.60E-11 | 12 | 4.85E-10 | 1.22E-10 | 3.53E-11 |
|  | Old | 15 | 1.07E-09 | 2.27E-10 | 5.85E-11 | 15 | 5.16E-10 | 8.47E-11 | 2.19E-11 |
| 6-ketoPGF_1α_ | WT young | 6 | 1.33E-11 | 3.27E-12 | 1.33E-12 | 6 | 1.31E-11 | 2.65E-12 | 1.08E-12 |
|  | WT old | 6 | 1.06E-11 | 2.00E-12 | 8.15E-13 | 6 | 1.12E-11 | 4.38E-12 | 1.79E-12 |
|  | WT | 12 | 1.19E-11 | 2.96E-12 | 8.54E-13 | 12 | 1.21E-11 | 3.59E-12 | 1.04E-12 |
|  | KO young | 6 | 2.27E-11 | 6.63E-12 | 2.71E-12 | 6 | 2.79E-11 | 4.82E-12 | 1.97E-12 |
|  | KO old | 9 | 1.92E-11 | 5.53E-12 | 1.84E-12 | 9 | 2.21E-11 | 4.77E-12 | 1.59E-12 |
|  | ABHD12 KO | 15 | 2.06E-11 | 6.04E-12 | 1.56E-12 | 15 | 2.44E-11 | 5.46E-12 | 1.41E-12 |
|  | Young | 12 | 1.80E-11 | 6.99E-12 | 2.02E-12 | 12 | 2.05E-11 | 8.57E-12 | 2.47E-12 |
|  | Old | 15 | 1.57E-11 | 6.15E-12 | 1.59E-12 | 15 | 1.78E-11 | 7.11E-12 | 1.84E-12 |

Supplemental Table 70: Output from ANOVA for striatum

|  | Effect of genotype | | | Effect of age | | | Genotype x age | |
| --- | --- | --- | --- | --- | --- | --- | --- | --- |
| Analyte | F (1,23) | p | Mag | F (1,23) | p | Mag | F (3,23) | p |
| *N*-palmitoyl alanine | 0.079 | 0.781 |  | 1.18 | 0.288 |  | 0.407 | 0.749 |
| *N*-stearoyl alanine | 0.081 | 0.779 |  | 0.031 | 0.861 |  | 0.048 | 0.986 |
| *N*-oleoyl alanine | **3.133** | **0.09** | **0.88** | 1.89 | 0.183 |  | **2.73** | **0.067** |
| *N*-arachidonoyl alanine | **14.18** | **0.001** | **1.34** | **3.29** | **0.083** | **1.17** | **6.69** | **0.002** |
| *N*-palmitoyl ethanolamine | 2.78 | 0.109 |  | 0.767 | 0.39 |  | **3.06** | **0.048** |
| *N*-stearoyl ethanolamine | 0.124 | 0.728 |  | 1.88 | 0.184 |  | **5.36** | **0.006** |
| *N*-oleoyl ethanolamine | 0.251 | 0.621 |  | 1.66 | 0.211 |  | 1.37 | 0.278 |
| *N*-linoleoyl ethanolamine | 0.244 | 0.626 |  | **3.66** | **0.068** | **0.86** | 1.52 | 0.237 |
| *N*-arachidonoyl ethanolamine | **5.29** | **0.031** | **1.29** | 1.14 | 0.298 |  | 2.04 | 0.136 |
| *N*-docosahexaenoyl ethanolamine | **3.64** | **0.069** | **0.88** | **5.29** | **0.031** | **0.86** | **3.3** | **0.038** |
| *N*-palmitoyl GABA | **4.34** | **0.049** | **0.86** | **32.85** | **0** | **0.7** | **13.37** | **0** |
| *N*-stearoyl GABA | 1.68 | 0.208 |  | **34.1** | **0** | **0.71** | **12.5** | **0** |
| *N*-oleoyl GABA | 0.816 | 0.376 |  | **26.27** | **0** | **0.67** | **9.79** | **0** |
| *N*-arachidonoyl GABA | **7.57** | **0.011** | **1.17** | **26.36** | **0** | **0.75** | **10.71** | **0** |
| *N*-docosahexaenoyl GABA | **8.65** | **0.007** | **0.8** | **18.12** | **0** | **0.74** | **10.19** | **0** |
| *N*-palmitoyl glycine | 1.32 | 0.262 |  | 1.03 | 0.32 |  | 1.01 | 0.406 |
| *N*-stearoyl glycine | 0.025 | 0.877 |  | **4.32** | **0.049** | **0.88** | 1.46 | 0.252 |
| *N*-oleoyl glycine | 0.147 | 0.705 |  | **5.14** | **0.033** | **0.89** | 1.98 | 0.145 |
| *N*-linoleoyl glycine | 0.084 | 0.774 |  | **4.11** | **0.054** | **0.83** | 1.74 | 0.187 |
| *N*-arachidonoyl glycine | 0.231 | 0.635 |  | **7.09** | **0.014** | **0.89** | **2.62** | **0.075** |
| *N*-docosahexaenoyl glycine | 0.719 | 0.405 |  | 0.881 | 0.358 |  | 0.77 | 0.523 |
| *N*-palmitoyl leucine | 2.21 | 0.151 |  | 0.357 | 0.556 |  | 0.977 | 0.421 |
| *N*-stearoyl leucine | 3.17 | 0.088 | 1.13 | 1.32 | 0.262 |  | 1.44 | 0.256 |
| *N*-oleoyl leucine | 0.604 | 0.445 |  | 0.507 | 0.484 |  | 0.413 | 0.745 |
| *N*-linoleoyl leucine | 2.06 | 0.164 |  | 0.109 | 0.774 |  | 1.73 | 0.189 |
| *N*-docosahexaenoyl leucine | 4.65 | 0.042 | 0.8 | 0.003 | 0.955 |  | 1.61 | 0.215 |
| *N*-palmitoyl methionine | 0.001 | 0.977 |  | 0.113 | 0.74 |  | 0.148 | 0.93 |
| *N*-stearoyl methionine | **13.2** | **0.001** | **1.36** | 2.73 | 0.112 |  | **5.4** | **0.006** |
| *N*-oleoyl methionine | 2.49 | 0.128 |  | 0.222 | 0.642 |  | 1.76 | 0.182 |
| *N*-palmitoyl phenylalanine | 0.755 | 0.394 |  | 1.79 | 0.194 |  | 1.22 | 0.325 |
| *N*-stearoyl phenylalanine | **3.64** | **0.069** | **1.17** | **2.98** | **0.098** | **0.86** | **2.34** | **0.1** |
| *N*-oleoyl phenylalanine | 0.005 | 0.945 |  | 1.17 | 0.291 |  | 0.74 | 0.539 |
| *N*-arachidonoyl phenylalanine | 1.95 | 0.175 |  | 1.87 | 0.184 |  | 1.47 | 0.249 |
| *N*-docosahexaenoyl phenylalanine | 2.07 | 0.164 |  | **4.09** | **0.055** | **0.74** | 2.04 | 0.136 |
| *N*-palmitoyl proline | 0.043 | 0.837 |  | 2.44 | 0.132 |  | 0.816 | 0.498 |
| *N*-stearoyl proline | 0.697 | 0.412 |  | 0.073 | 0.826 |  | 0.538 | 0.661 |
| *N*-palmitoyl serine | **9.13** | **0.006** | **0.86** | 0.941 | 0.342 |  | **3.79** | **0.024** |
| *N*-stearoyl serine | 0.404 | 0.532 |  | 1.34 | 0.259 |  | 0.86 | 0.476 |
| *N*-oleoyl serine | **3.19** | **0.087** | **0.91** | 0.007 | 0.932 |  | 1.38 | 0.275 |
| *N*-linoleoyl serine | 0.074 | 0.788 |  | 0.061 | 0.808 |  | 0.096 | 0.961 |
| *N*-arachidonoyl serine | **27.96** | **0** | **1.32** | 0.116 | 0.736 |  | **10.79** | **0** |
| *N*-docosahexaenoyl serine | 0.88 | 0.358 |  | 1.02 | 0.324 |  | 1.32 | 0.292 |
| *N*-palmitoyl taurine | 0.002 | 0.963 |  | 1.69 | 0.206 |  | 1.96 | 0.148 |
| *N*-stearoyl taurine | 1.25 | 0.276 |  | **3.49** | **0.074** | **0.89** | 1.95 | 0.151 |
| *N-*oleoyl taurine | 0.29 | 0.596 |  | 1.74 | 0.2 |  | 0.866 | 0.473 |
| *N*-arachidonoyl taurine | **6.42** | **0.019** | **1.18** | 0.81 | 0.377 |  | **2.48** | **0.087** |
| *N*-palmitoyl tryptophan | 0.806 | 0.378 |  | 0.006 | 0.941 |  | 0.317 | 0.813 |
| *N*-stearoyl tryptophan | 2.09 | 0.161 |  | 0.038 | 0.846 |  | 0.921 | 0.446 |
| *N*-palmitoyl tyrosine | 1.6 | 0.218 |  | 1.11 | 0.3 |  | 1.16 | 0.346 |
| *N*-stearoyl tyrosine | **3.52** | **0.073** | **1.21** | **3.19** | **0.087** | **0.81** | 2.33 | 0.101 |
| *N-*oleoyl tyrosine | **4.25** | **0.051** | **1.41** | 2.21 | 0.15 |  | 2.21 | 0.115 |
| *N*-arachidonoyl tyrosine | **40.04** | **0** | **1.62** | **7.43** | **0.012** | **0.85** | **14.9** | **0** |
| *N*-palmitoyl valine | 1.19 | 0.287 |  | 0.264 | 0.612 |  | 1.82 | 0.171 |
| *N*-stearoyl valine | **9.59** | **0.005** | **1.27** | 0.652 | 0.428 |  | **3.36** | **0.036** |
| *N*-oleoyl valine | 0.492 | 0.49 |  | 0.024 | 0.877 |  | 0.467 | 0.708 |
| 2-palmitoyl glycerol | 0.277 | 0.603 |  | **12.19** | **0.002** | **0.74** | **4.39** | **0.014** |
| 2-oleoyl glycerol | **9.73** | **0.005** | **0.8** | 0.003 | 0.987 |  | **3.34** | **0.037** |
| 2-linoleoyl glycerol | 0.343 | 0.564 |  | **8.64** | **0.007** | **0.76** | **4.39** | **0.014** |
| 2-arachidonoyl glycerol | **14.82** | **0.001** | **1.29** | **2.93** | **0.1** | **0.92** | **5.92** | **0.004** |
| Oleic acid | 0.45 | 0.509 |  | **8.09** | **0.009** | **0.83** | **3.01** | **0.051** |
| Linoleic acid | 0.462 | 0.503 |  | **5.59** | **0.027** | **0.85** | 2.15 | 0.122 |
| Arachidonic acid | 0.414 | 0.526 |  | **8.68** | **0.007** | **0.85** | **3.12** | **0.046** |
| phosphoLEA | 0.482 | 0.495 |  | 0.05 | 0.826 |  | 0.18 | 0.91 |
| PGE_2_ | **21.81** | **0** | **1.24** | 0.221 | 0.643 |  | **7.33** | **0.001** |
| PGF_2α_ | **42.51** | **0** | **1.31** | **6.14** | **0.021** | **0.92** | **15.38** | **0** |
| 6-ketoPGF_1α_ | **23.36** | **0** | **1.63** | **16.92** | **0** | **1.53** | **16.63** | **0** |
| Sample Mass | 0.004 | 0.953 |  | 1.26 | 0.273 |  | 0.715 | 0.553 |

Supplemental Table 71: Output from ANOVA for hippocampus

|  | Effect of genotype | | | Effect of age | | | Genotype x age | |
| --- | --- | --- | --- | --- | --- | --- | --- | --- |
| Analyte | F (1,23) | p | Mag | F (1,23) | p | Mag | F (3,23) | p |
| *N*-palmitoyl alanine | 0.128 | 0.724 |  | **43.97** | **0** | **0.55** | **14.88** | **0** |
| *N*-stearoyl alanine | 1.13 | 0.3 |  | **41.69** | **0** | **0.66** | **14.2** | **0** |
| *N*-oleoyl alanine | 0.76 | 0.382 |  | **53.27** | **0** | **0.65** | **19.17** | **0** |
| *N*-linoleoyl alanine | 0.319 | 0.578 |  | **17.67** | **0** | **0.61** | **6.29** | **0.003** |
| *N*-arachidonoyl alanine | **3.56** | **0.072** | **1.05** | **61.09** | **0** | **0.69** | **20.88** | **0** |
| *N*-palmitoyl ethanolamine | 1.94 | 0.177 |  | 2.72 | 0.113 |  | 1.72 | 0.188 |
| *N*-stearoyl ethanolamine | 0.068 | 0.796 |  | **5.46** | **0.028** | **1.58** | 2.09 | 0.129 |
| *N*-oleoyl ethanolamine | 0.41 | 0.528 |  | **6.47** | **0.018** | **0.73** | **2.43** | **0.091** |
| *N*-linoleoyl ethanolamine | 0.017 | 0.897 |  | **61.51** | **0** | **0.53** | **20.72** | **0** |
| *N*-arachidonoyl ethanolamine | **12.31** | **0.002** | **1.24** | **39.92** | **0** | **0.63** | **16.5** | **0** |
| *N*-docosahexaenoyl ethanolamine | **15.07** | **0.001** | **0.8** | **69.14** | **0** | **0.66** | **30.61** | **0** |
| *N*-palmitoyl GABA | 2.87 | 0.104 |  | **70.1** | **0** | **0.6** | **25.4** | **0** |
| *N*-stearoyl GABA | 0.433 | 0.517 |  | **50.56** | **0** | **0.65** | **16.92** | **0** |
| *N*-oleoyl GABA | **3.6** | **0.071** | **0.83** | **50.38** | **0** | **0.6** | **19** | **0** |
| *N*-linoleoyl GABA | 1.47 | 0.238 |  | **19.86** | **0** | **0.55** | **8.06** | **0.001** |
| *N*-arachidonoyl GABA | 0.026 | 0.874 |  | **59.08** | **0** | **0.63** | **20.08** | **0** |
| *N*-docosahexaenoyl GABA | 0.207 | 0.653 |  | **46.37** | **0** | **0.61** | **15.98** | **0** |
| *N*-palmitoyl glycine | 0.226 | 0.639 |  | **8.34** | **0.008** | **0.89** | **3.33** | **0.037** |
| *N*-stearoyl glycine | 0.098 | 0.758 |  | **18.82** | **0** | **0.79** | **6.39** | **0.003** |
| *N*-oleoyl glycine | 1.57 | 0.222 |  | **29.54** | **0** | **0.81** | **10.88** | **0** |
| *N*-linoleoyl glycine | 0.376 | 0.546 |  | **22.38** | **0** | **0.71** | **7.83** | **0.001** |
| *N*-arachidonoyl glycine | 1.96 | 0.175 |  | **35.79** | **0** | **0.76** | **12.27** | **0** |
| *N*-docosahexaenoyl glycine | 1.25 | 0.275 |  | **30.76** | **0** | **0.75** | **11** | **0** |
| *N*-palmitoyl leucine | 0.379 | 0.544 |  | **12.15** | **0.002** | **0.85** | **4.9** | **0.009** |
| *N*-stearoyl leucine | 0.037 | 0.85 |  | **8.96** | **0.007** | **0.85** | **3.91** | **0.021** |
| *N*-oleoyl leucine | 0.081 | 0.779 |  | **4.96** | **0.036** | **0.85** | 1.97 | 0.146 |
| *N*-linoleoyl leucine | 0.37 | 0.549 |  | **16.93** | **0** | **0.64** | **6.71** | **0.002** |
| *N*-docosahexaenoyl leucine | **13.75** | **0.001** | **0.73** | 1.32 | 0.262 |  | **6.27** | **0.003** |
| *N*-palmitoyl methionine | 0.226 | 0.639 |  | 0.648 | 0.429 |  | 0.355 | 0.786 |
| *N*-stearoyl methionine | **4.65** | **0.042** | **1.15** | **6.59** | **0.017** | **0.82** | **3.93** | **0.028** |
| *N*-oleoyl methionine | 0.07 | 0.794 |  | 0.368 | 0.55 |  | 0.137 | 0.937 |
| *N*-palmitoyl phenylalanine | 0.842 | 0.368 |  | **3.84** | **0.062** | **0.83** | 1.69 | 0.197 |
| *N*-stearoyl phenylalanine | **2.95** | **0.099** | **1.15** | 2.47 | 0.13 |  | 1.94 | 0.152 |
| *N*-oleoyl phenylalanine | **7.26** | **0.013** | **1.21** | **3.71** | **0.066** | **0.87** | **3.68** | **0.027** |
| *N*-arachidonoyl phenylalanine | **5** | **0.035** | **1.23** | 0.008 | 0.93 |  | 1.71 | 0.192 |
| *N*-docosahexaenoyl phenylalanine | 1.52 | 0.229 |  | **6.16** | **0.021** | **0.75** | **2.74** | **0.067** |
| *N*-palmitoyl proline | **4.14** | **0.054** | **1.17** | 0.016 | 0.902 |  | 2.2 | 0.116 |
| *N*-stearoyl proline | **11.87** | **0.002** | **1.25** | 1.74 | 0.2 |  | **5.85** | **0.004** |
| *N*-palmitoyl serine | **13.38** | **0.001** | **0.9** | **10.33** | **0.004** | **1.11** | **8.23** | **0.001** |
| *N*-stearoyl serine | 0.854 | 0.365 |  | 2.37 | 0.138 |  | 1.03 | 0.399 |
| *N*-oleoyl serine | 2.7 | 0.114 |  | **11.13** | **0.003** | **1.12** | **4.7** | **0.011** |
| *N*-linoleoyl serine | **3.88** | **0.061** | **1.21** | **11.04** | **0.003** | **0.69** | **4.61** | **0.011** |
| *N*-arachidonoyl serine | **26.85** | **0** | **1.49** | **3.39** | **0.079** | **0.9** | **9.58** | **0** |
| *N*-docosahexaenoyl serine | 0.885 | 0.357 |  | 0.747 | 0.396 |  | 0.665 | 0.582 |
| *N*-palmitoyl taurine | **8.95** | **0.007** | **0.82** | **4.16** | **0.053** | **1.13** | **4.17** | **0.017** |
| *N*-stearoyl taurine | 1.55 | 0.225 |  | **5.45** | **0.029** | **1.12** | **3.36** | **0.036** |
| *N-*oleoyl taurine | **6.57** | **0.017** | **0.86** | 2.28 | 0.145 |  | **2.79** | **0.063** |
| *N*-arachidonoyl taurine | 1.36 | 0.255 |  | 0.172 | 0.682 |  | 0.549 | 0.654 |
| *N*-palmitoyl tryptophan | 0.002 | 0.966 |  | 0.544 | 0.468 |  | 0.269 | 0.847 |
| *N*-stearoyl tryptophan | 2.77 | 0.109 |  | 0.05 | 0.825 |  | 0.995 | 0.413 |
| *N*-palmitoyl tyrosine | 0.917 | 0.348 |  | 0.642 | 0.431 |  | 1.38 | 0.273 |
| *N*-stearoyl tyrosine | 2 | 0.173 |  | **4.28** | **0.051** | **0.77** | 2.26 | 0.108 |
| *N-*oleoyl tyrosine | **3.86** | **0.062** | **1.47** | 0.374 | 0.547 |  | 1.5 | 0.242 |
| *N*-arachidonoyl tyrosine | **18.52** | **0** | **1.59** | **3.37** | **0.08** | **0.84** | **7.5** | **0.001** |
| *N*-docosahexaenoyl tyrosine | **3.72** | **0.066** | **1.36** | 0.361 | 0.554 |  | 2.26 | 0.109 |
| *N*-palmitoyl valine | 0.421 | 0.523 |  | 1.25 | 0.276 |  | 0.571 | 0.64 |
| *N*- stearoyl valine | 1.2 | 0.286 |  | 1.65 | 0.212 |  | 0.874 | 0.469 |
| *N*-oleoyl valine | 1.33 | 0.26 |  | 0.871 | 0.36 |  | 0.716 | 0.552 |
| 2-palmitoyl glycerol | 0.661 | 0.425 |  | 0.239 | 0.629 |  | 0.554 | 0.651 |
| 2-oleoyl glycerol | **13.35** | **0.001** | **0.8** | 0.005 | 0.942 |  | **4.49** | **0.013** |
| 2-linoleoyl glycerol | 0.113 | 0.74 |  | **10.62** | **0.003** | **0.78** | **3.73** | **0.025** |
| 2-arachidonoyl glycerol | **3.01** | **0.096** | **1.12** | 2.83 | 0.106 |  | **2.55** | **0.081** |
| Oleic acid | 0.059 | 0.81 |  | **38.6** | **0** | **0.73** | **13.51** | **0** |
| Linoleic acid | 0.333 | 0.57 |  | **53.72** | **0** | **0.73** | **18.38** | **0** |
| Arachidonic acid | **5.77** | **0.025** | **1.07** | **47.69** | **0** | **0.76** | **17.4** | **0** |
| phosphoLEA | 1.6 | 0.219 |  | 0.027 | 0.871 |  | 0.584 | 0.631 |
| PGE_2_ | 1.08 | 0.31 |  | 0.002 | 0.966 |  | 0.729 | 0.545 |
| PGF_2α_ | **5.1** | **0.034** | **1.16** | 0.072 | 0.791 |  | **3.69** | **0.027** |
| 6-ketoPGF_1α_ | **38.3** | **0** | **2.06** | **7.11** | **0.014** | **0.77** | **14.71** | **0** |
| Sample Mass | 1.28 | 0.269 |  | 6.92 | 0.015 | 1.09 | **2.61** | **0.076** |

Supplemental Table 72: Output from ANOVA for cerebellum

|  | Effect of genotype | | | Effect of age | | | Genotype x age | |
| --- | --- | --- | --- | --- | --- | --- | --- | --- |
| Analyte | F (1,23) | p | Mag | F (1,23) | p | Mag | F (3,23) | p |
| *N*-palmitoyl alanine | 0.224 | 0.641 |  | 0.089 | 0.768 |  | 0.158 | 0.923 |
| *N*-stearoyl alanine | 0.008 | 0.93 |  | 0.158 | 0.695 |  | 0.093 | 0.963 |
| *N*-oleoyl alanine | **7.9** | **0.01** | **0.8** | 2.39 | 0.136 |  | **4.41** | **0.014** |
| *N*-linoleoyl alanine | 0.014 | 0.908 |  | 1.03 | 0.321 |  | 0.475 | 0.702 |
| *N*-arachidonoyl alanine | **25.16** | **0** | **1.36** | 0.237 | 0.631 |  | **15.51** | **0** |
| *N*-palmitoyl ethanolamine | 1.57 | 0.223 |  | **38.96** | **0** | **1.64** | **15.51** | **0** |
| *N*-stearoyl ethanolamine | 0.691 | 0.415 |  | **9.84** | **0.005** | **2.93** | **3.71** | **0.026** |
| *N*-oleoyl ethanolamine | 0.01 | 0.92 |  | **12.32** | **0.002** | **1.69** | **4.17** | **0.017** |
| *N*-linoleoyl ethanolamine | **8.64** | **0.007** | **1.23** | 0.036 | 0.852 |  | **2.94** | **0.054** |
| *N*-arachidonoyl ethanolamine | **62.85** | **0** | **1.8** | 1.93 | 0.178 |  | **23.16** | **0** |
| *N*-docosahexaenoyl ethanolamine | 0.06 | 0.809 |  | 0.95 | 0.34 |  | 0.447 | 0.722 |
| *N*-palmitoyl GABA | 2.49 | 0.128 |  | **18.19** | **0** | **0.65** | **7.41** | **0.001** |
| *N*-stearoyl GABA | 0.027 | 0.871 |  | **15.5** | **0.001** | **0.65** | **5.28** | **0.006** |
| *N*-oleoyl GABA | 2.13 | 0.158 |  | **18.41** | **0** | **0.58** | **7.32** | **0.001** |
| *N*-linoleoyl GABA | 1.12 | 0.302 |  | **28.92** | **0** | **0.49** | **10.5** | **0** |
| *N*-arachidonoyl GABA | **7.31** | **0.013** | **1.11** | **97.32** | **0** | **0.56** | **34.43** | **0** |
| *N*-docosahexaenoyl GABA | 2.13 | 0.158 |  | **39.82** | **0** | **0.62** | **15.04** | **0** |
| *N*-palmitoyl glycine | 1.03 | 0.32 |  | **11.81** | **0.002** | **0.88** | **4.57** | **0.012** |
| *N*-stearoyl glycine | 0.211 | 0.65 |  | **5.34** | **0.03** | **0.88** | 1.94 | 0.152 |
| *N*-oleoyl glycine | 2.46 | 0.131 |  | **4.1** | **0.055** | **0.9** | **2.43** | **0.091** |
| *N*-linoleoyl glycine | 0.044 | 0.835 |  | 0.137 | 0.714 |  | 0.152 | 0.927 |
| *N*-arachidonoyl glycine | 1.65 | 0.212 |  | 2.58 | 0.122 |  | 1.31 | 0.927 |
| *N*-docosahexaenoyl glycine | 0.802 | 0.38 |  | 0.762 | 0.392 |  | 0.885 | 0.464 |
| *N*-palmitoyl leucine | 2.5 | 0.128 |  | 0.032 | 0.86 |  | 2.06 | 0.133 |
| *N*-stearoyl leucine | 0.468 | 0.501 |  | 0.896 | 0.354 |  | 1.96 | 0.149 |
| *N*-oleoyl leucine | **4.57** | **0.043** | **0.85** | 1.01 | 0.33 |  | 2.19 | 0.117 |
| *N*-linoleoyl leucine | 0.167 | 0.687 |  | **11.04** | **0.003** | **0.66** | **4.34** | **0.015** |
| *N*-docosahexaenoyl leucine | **11.91** | **0.002** | **0.78** | 2.05 | 0.166 |  | **5.05** | **0.008** |
| *N*-palmitoyl methionine | 0.914 | 0.349 |  | 0.509 | 0.483 |  | 0.622 | 0.608 |
| *N*-stearoyl methionine | **3.06** | **0.094** | **1.19** | 0.177 | 0.678 |  | 1.97 | 0.147 |
| *N*-oleoyl methionine | 0.039 | 0.845 |  | 0.269 | 0.61 |  | 0.133 | 0.939 |
| *N*-palmitoyl phenylalanine | 0.785 | 0.385 |  | **3.77** | **0.065** | **0.78** | 2.23 | 0.111 |
| *N*-stearoyl phenylalanine | **4.41** | **0.047** | **1.08** | **7.73** | **0.011** | **0.87** | **6.35** | **0.003** |
| *N*-oleoyl phenylalanine | 1.36 | 0.256 |  | 2.12 | 0.159 |  | 1.82 | 0.172 |
| *N*-linoleoyl phenylalanine | 1.03 | 0.32 |  | 2.61 | 0.12 |  | 1.21 | 0.328 |
| *N*-arachidonoyl phenylalanine | **12.71** | **0.002** | **1.51** | **3.60** | **0.07** | **0.79** | **8.09** | **0.001** |
| *N*-docosahexaenoyl phenylalanine | 1.14 | 0.297 |  | 0.61 | 0.443 |  | 0.939 | 0.438 |
| *N*-palmitoyl proline | 0.208 | 0.653 |  | 1.47 | 0.238 |  | 0.86 | 0.476 |
| *N*-stearoyl proline | 0.04 | 0.844 |  | **3.05** | **0.094** | **1.15** | 1.08 | 0.375 |
| *N-*oleoyl proline | 0.017 | 0.897 |  | **3.76** | **0.065** | **1.21** | 1.55 | 0.228 |
| *N*-palmitoyl serine | **24.3** | **0** | **0.89** | **11.88** | **0** | **1.08** | **11.14** | **0** |
| *N*-stearoyl serine | 0.9 | 0.353 |  | **20.07** | **0** | **1.14** | **6.86** | **0.002** |
| *N*-oleoyl serine | **9.38** | **0.006** | **0.92** | **8.4** | **0.008** | **1.09** | **5.95** | **0.004** |
| *N*-linoleoyl serine | 0.528 | 0.475 |  | 1.03 | 0.322 |  | 0.516 | 0.675 |
| *N*-arachidonoyl serine | **32.45** | **0** | **1.35** | 2.67 | 0.116 |  | **11.66** | **0** |
| *N*-docosahexaenoyl serine | 0.189 | 0.668 |  | 0.023 | 0.881 |  | 0.07 | 0.96 |
| *N*-palmitoyl taurine | **20.23** | **0** | **0.84** | **4.79** | **0.039** | **0.91** | **9.13** | **0** |
| *N*-stearoyl taurine | 0.208 | 0.653 |  | **8.3** | **0.008** | **0.87** | **2.98** | **0.053** |
| *N-*oleoyl taurine | 2.5 | 0.127 |  | 1.65 | 0.212 |  | 1.52 | 0.236 |
| *N*-arachidonoyl taurine | **7.56** | **0.011** | **1.12** | **8.21** | **0.009** | **0.89** | **5.06** | **0.008** |
| *N*-palmitoyl tryptophan | 0.021 | 0.887 |  | 0.023 | 0.881 |  | 0.029 | 0.993 |
| *N*-stearoyl tryptophan | 0.529 | 0.474 |  | 0.61 | 0.443 |  | 1.82 | 0.172 |
| *N*-oleoyl tryptophan | **5.54** | **0.028** | **1.69** | 0.172 | 0.68 |  | 2.03 | 0.137 |
| *N*-palmitoyl tyrosine | 1.67 | 0.209 |  | 2.81 | 0.107 |  | 1.91 | 0.156 |
| *N*-stearoyl tyrosine | 1.47 | 0.237 |  | **4.75** | **0.04** | **0.78** | **2.6** | **0.077** |
| *N-*oleoyl tyrosine | 2 | 0.171 |  | 2.61 | 0.12 |  | 1.97 | 0.147 |
| *N*-arachidonoyl tyrosine | **33.39** | **0** | **2.06** | **8.94** | **0.007** | **0.73** | **14.07** | **0** |
| *N*-docosahexaenoyl tyrosine | 0.081 | 0.778 |  | 0.428 | 0.52 |  | 1.17 | 0.344 |
| *N*-palmitoyl valine | 0.498 | 0.488 |  | 1.23 | 0.279 |  | 1.32 | 0.293 |
| *N*- stearoyl valine | 0.584 | 0.453 |  | 1.98 | 0.173 |  | 0.929 | 0.443 |
| *N*-oleoyl valine | 0.087 | 0.771 |  | 1.06 | 0.314 |  | 0.852 | 0.48 |
| 2-palmitoyl glycerol | **6.74** | **0.016** | **0.87** | **38.94** | **0** | **1.55** | **14.41** | **0** |
| 2-oleoyl glycerol | **6.08** | **0.022** | **0.94** | **106.76** | **0** | **1.62** | **36.81** | **0** |
| 2-linoleoyl glycerol | 1.95 | 0.176 |  | 0.977 | 0.333 |  | 1.08 | 0.377 |
| 2-arachidonoyl glycerol | **10.55** | **0.004** | **1.2** | **9.83** | **0.005** | **1.19** | **7.63** | **0.001** |
| Oleic acid | 0.008 | 0.928 |  | 1.19 | 0.287 |  | 0.497 | 0.688 |
| Linoleic acid | 0.577 | 0.455 |  | **6.18** | **0.021** | **0.89** | 2.17 | 0.119 |
| Arachidonic acid | **13.53** | **0.001** | **1.12** | **5.91** | **0.023** | **0.93** | **6.06** | **0.003** |
| phosphoLEA | 0.155 | 0.897 |  | **7.04** | **0.014** | **1.26** | **2.52** | **0.084** |
| PGE_2_ | **19.87** | **0** | **1.3** | 0.356 | 0.556 |  | **7.32** | **0.001** |
| PGF_2α_ | **28.18** | **0** | **1.42** | 0.001 | 0.979 |  | **10.14** | **0** |
| 6-ketoPGF_1α_ | **22.87** | **0** | **1.58** | 1.27 | 0.271 |  | **7.89** | **0.001** |
| Sample Mass | 0.815 | 0.376 |  | 0.039 | 0.845 |  | 0.307 | 0.82 |

Supplemental Table 73: Output from ANOVA for thalamus

|  | Effect of genotype | | | Effect of age | | | Genotype x age | |
| --- | --- | --- | --- | --- | --- | --- | --- | --- |
| Analyte | F (1,23) | p | Mag | F (1,23) | p | Mag | F (3,23) | p |
| *N*-palmitoyl alanine | 2.7 | 0.114 |  | 0.147 | 0.705 |  | 0.995 | 0.413 |
| *N*-stearoyl alanine | **6.52** | **0.018** | **1.19** | 0.198 | 0.661 |  | **2.53** | **0.082** |
| *N*-oleoyl alanine | 0.043 | 0.838 |  | 0.16 | 0.693 |  | 0.601 | 0.621 |
| *N*-arachidonoyl alanine | **8.8** | **0.007** | **1.35** | 0.112 | 0.741 |  | **3.08** | **0.047** |
| *N*-palmitoyl ethanolamine | 0.888 | 0.356 |  | **9.29** | **0.006** | **1.27** | **3.81** | **0.024** |
| *N*-stearoyl ethanolamine | 2.67 | 0.116 |  | **13.8** | **0.001** | **2.6** | **6.48** | **0.002** |
| *N*-oleoyl ethanolamine | **3.85** | **0.062** | **1.2** | **3.18** | **0.088** | **1.19** | **2.58** | **0.078** |
| *N*-linoleoyl ethanolamine | **15.93** | **0.001** | **1.29** | **14.83** | **0.001** | **0.77** | **10.03** | **0** |
| *N*-arachidonoyl ethanolamine | **33.49** | **0** | **1.74** | **4.44** | **0.046** | **0.85** | **12.58** | **0** |
| *N*-docosahexaenoyl ethanolamine | **3.57** | **0.072** | **1.12** | **2.94** | **0.1** | **0.89** | **2.84** | **0.06** |
| *N*-palmitoyl GABA | 1.05 | 0.314 |  | **33.98** | **0** | **0.6** | **12.14** | **0** |
| *N*-stearoyl GABA | 0.001 | 0.98 |  | **9.79** | **0.005** | **0.73** | **3.32** | **0.038** |
| *N*-oleoyl GABA | 0.005 | 0.942 |  | **40.33** | **0** | **0.63** | **13.65** | **0** |
| *N*-linoleoyl GABA | 1.21 | 0.282 |  | **36.82** | **0** | **0.53** | **13.23** | **0** |
| *N*-arachidonoyl GABA | **10.76** | **0.003** | **1.17** | **89.18** | **0** | **0.53** | **32.49** | **0** |
| *N*-docosahexaenoyl GABA | 1.23 | 0.279 |  | **30.1** | **0** | **0.6** | **10.18** | **0** |
| *N*-palmitoyl glycine | 2.64 | 0.118 |  | 0.806 | 0.378 |  | 1.41 | 0.266 |
| *N*-stearoyl glycine | 1.59 | 0.22 |  | **9.35** | **0.006** | **0.85** | **3.44** | **0.034** |
| *N*-oleoyl glycine | 2.24 | 0.148 |  | **8.58** | **0.008** | **0.88** | **3.38** | **0.036** |
| *N*-linoleoyl glycine | 1.4 | 0.249 |  | **15** | **0.001** | **0.76** | **5.3** | **0.006** |
| *N*-arachidonoyl glycine | **8.16** | **0.009** | **1.14** | **11.25** | **0.003** | **0.85** | **6.01** | **0.004** |
| *N*-docosahexaenoyl glycine | 2.05 | 0.165 |  | **7.96** | **0.01** | **0.85** | **3.2** | **0.042** |
| *N*-palmitoyl leucine | 0.171 | 0.683 |  | 0.034 | 0.855 |  | 0.228 | 0.876 |
| *N*-stearoyl leucine | 0.563 | 0.46 |  | 0.737 | 0.399 |  | 0.398 | 0.756 |
| *N*-oleoyl leucine | 0.123 | 0.729 |  | 0.159 | 0.694 |  | 0.337 | 0.799 |
| *N*-linoleoyl leucine | 0.355 | 0.557 |  | 0.343 | 0.564 |  | 0.408 | 0.749 |
| *N*-docosahexaenoyl leucine | **21** | **0** | **0.7** | **3.84** | **0.062** | **0.84** | **9.09** | **0** |
| *N*-palmitoyl methionine | 0.716 | 0.406 |  | 0.61 | 0.443 |  | 0.487 | 0.695 |
| *N*-stearoyl methionine | **4.92** | **0.037** | **1.19** | **6.79** | **0.016** | **0.81** | **3.72** | **0.026** |
| *N*-oleoyl methionine | 2.63 | 0.119 |  | 0.317 | 0.579 |  | 1.03 | 0.398 |
| *N*-palmitoyl phenylalanine | **5.6** | **0.027** | **1.23** | 1.36 | 0.256 |  | **2.74** | **0.067** |
| *N*-stearoyl phenylalanine | **7.29** | **0.013** | **1.28** | 2.91 | 0.102 |  | **4.1** | **0.018** |
| *N*-oleoyl phenylalanine | **8.33** | **0.008** | **1.25** | 2.04 | 0.166 |  | **4.18** | **0.017** |
| *N*-arachidonoyl phenylalanine | **26.56** | **0** | **1.75** | 0.281 | 0.601 |  | **9.61** | **0** |
| *N*-docosahexaenoyl phenylalanine | **3.23** | **0.085** | **1.26** | 0.001 | 0.975 |  | 2 | 0.141 |
| *N*-palmitoyl proline | 0.744 | 0.397 |  | 1.56 | 0.224 |  | 1.11 | 0.365 |
| *N*-stearoyl proline | **3.23** | **0.085** | **1.29** | 0.163 | 0.69 |  | 1.19 | 0.337 |
| *N-*oleoyl proline | 2.92 | 0.101 |  | 0.003 | 0.959 |  | 0.992 | 0.414 |
| *N*-palmitoyl serine | 0.047 | 0.83 |  | 0.703 | 0.41 |  | 0.505 | 0.683 |
| *N*-stearoyl serine | 1.66 | 0.21 |  | 0.081 | 0.778 |  | 0.696 | 0.564 |
| *N*-oleoyl serine | 0.042 | 0.84 |  | **4.45** | **0.046** | **1.1** | 2.22 | 0.114 |
| *N*-linoleoyl serine | 0.137 | 0.715 |  | 0.131 | 0.721 |  | 0.131 | 0.941 |
| *N*-arachidonoyl serine | **38** | **0** | **1.88** | 0.131 | 0.721 |  | **13.13** | **0** |
| *N*-docosahexaenoyl serine | **15.96** | **0.001** | **1.44** | 0.187 | 0.67 |  | **5.42** | **0.006** |
| *N*-palmitoyl taurine | 1.19 | 0.286 |  | 0.941 | 0.342 |  | 2.19 | 0.117 |
| *N*-stearoyl taurine | **2.96** | **0.099** | **1.11** | 0.816 | 0.376 |  | 1.55 | 0.229 |
| *N-*oleoyl taurine | 0.1 | 0.754 |  | **3** | **0.097** | **0.93** | **2.76** | **0.065** |
| *N*-arachidonoyl taurine | **14.28** | **0.001** | **1.2** | 1.15 | 0.294 |  | **5.56** | **0.005** |
| *N*-palmitoyl tryptophan | 1.12 | 0.301 |  | 0.278 | 0.603 |  | 1.98 | 0.145 |
| *N*-stearoyl tryptophan | **3.41** | **0.078** | **1.25** | 0.117 | 0.736 |  | 1.53 | 0.234 |
| *N*-palmitoyl tyrosine | **6.96** | **0.015** | **1.33** | 1.1 | 0.306 |  | **3.06** | **0.049** |
| *N*-stearoyl tyrosine | **7.79** | **0.01** | **1.35** | **4.4** | **0.047** | **0.8** | **4.33** | **0.015** |
| *N-*oleoyl tyrosine | **6.04** | **0.022** | **1.21** | 1.18 | 0.289 |  | **3.61** | **0.029** |
| *N*-arachidonoyl tyrosine | **51.74** | **0** | **1.98** | 1.78 | 0.196 |  | **17.41** | **0** |
| *N*-docosahexaenoyl tyrosine | **20.35** | **0** | **1.68** | 0.841 | 0.369 |  | **7.43** | **0.001** |
| *N*-palmitoyl valine | 0.766 | 0.391 |  | 0.007 | 0.936 |  | 0.485 | 0.696 |
| *N*- stearoyl valine | **3.33** | **0.081** | **1.29** | 1.54 | 0.228 |  | 1.52 | 0.237 |
| *N*-oleoyl valine | 0.38 | 0.543 |  | 0.008 | 0.93 |  | 0.196 | 0.898 |
| 2-palmitoyl glycerol | 0.071 | 0.792 |  | 1.58 | 0.222 |  | 0.76 | 0.528 |
| 2-oleoyl glycerol | **3.77** | **0.064** | **0.92** | **7.71** | **0.011** | **1.13** | **3.59** | **0.029** |
| 2-linoleoyl glycerol | 0.399 | 0.534 |  | **7.11** | **0.014** | **0.83** | **2.57** | **0.079** |
| 2-arachidonoyl glycerol | **15.59** | **0.001** | **1.29** | 0.068 | 0.797 |  | **5.23** | **0.007** |
| Oleic acid | 1.14 | 0.298 |  | **24.41** | **0** | **0.69** | **8.71** | **0** |
| Linoleic acid | 2.43 | 0.133 |  | **31.71** | **0** | **0.69** | **11.15** | **0** |
| Arachidonic acid | **8** | **0.01** | **1.15** | **26.78** | **0** | **0.74** | **11.09** | **0** |
| phosphoLEA | 0.01 | 0.923 |  | 0.149 | 0.703 |  | 0.06 | 0.98 |
| PGE_2_ | **37.73** | **0** | **1.42** | 0.078 | 0.783 |  | **13.04** | **0** |
| PGF_2α_ | **76.74** | **0** | **1.43** | 1.25 | 0.276 |  | **25.82** | **0** |
| 6-ketoPGF_1α_ | **89.82** | **0** | **2.17** | 1.91 | 0.181 |  | **30.1** | **0** |
| Sample Mass | **5.91** | **0.023** | **0.89** | 0 | 0.987 |  | 2.05 | 0.135 |

Supplemental Table 74: Output from ANOVA for cortex

|  | Effect of genotype | | | Effect of age | | | Genotype x age | |
| --- | --- | --- | --- | --- | --- | --- | --- | --- |
| Analyte | F (1,23) | p | Mag | F (1,23) | p | Mag | F (3,23) | p |
| *N*-palmitoyl alanine | 0.581 | 0.454 |  | 1.69 | 0.207 |  | 1.73 | 0.188 |
| *N*-stearoyl alanine | **11.32** | **0.003** | **1.11** | **3.72** | **0.066** | **1.07** | **6.22** | **0.003** |
| *N*-oleoyl alanine | 1.12 | 0.301 |  | 2.02 | 0.169 |  | 1.76 | 0.183 |
| *N*-arachidonoyl alanine | **10.65** | **0.003** | **1.26** | 1.29 | 0.269 |  | **4.39** | **0.014** |
| *N*-docosahexaenoyl alanine | 0.744 | 0.397 |  | 0.313 | 0.581 |  | 1.06 | 0.386 |
| *N*-palmitoyl ethanolamine | 0.14 | 0.712 |  | **13.76** | **0.001** | **1.44** | **4.96** | **0.008** |
| *N*-stearoyl ethanolamine | 0.401 | 0.533 |  | **4.28** | **0.05** | **1.75** | 2.04 | 0.136 |
| *N*-oleoyl ethanolamine | 0.001 | 0.971 |  | **11.39** | **0.003** | **1.41** | **4.16** | **0.017** |
| *N*-linoleoyl ethanolamine | 1.48 | 0.237 |  | 1.87 | 0.184 |  | 1.04 | 0.392 |
| *N*-arachidonoyl ethanolamine | **23.9** | **0** | **1.6** | 0.003 | 0.957 |  | **8.1** | **0.001** |
| *N*-docosahexaenoyl ethanolamine | 0.217 | 0.646 |  | 0.714 | 0.407 |  | 0.337 | 0.799 |
| *N*-palmitoyl GABA | **3.14** | **0.09** | **0.75** | **25.68** | **0** | **0.5** | **10.33** | **0** |
| *N*-stearoyl GABA | 0.109 | 0.745 |  | **25.84** | **0** | **0.59** | **8.84** | **0** |
| *N*-oleoyl GABA | 1.29 | 0.267 |  | **30.56** | **0** | **0.53** | **11.1** | **0** |
| *N*-linoleoyl GABA | 0.352 | 0.559 |  | **28.7** | **0** | **0.43** | **10.12** | **0** |
| *N*-arachidonoyl GABA | **5.53** | **0.028** | **1.15** | **48.28** | **0** | **0.54** | **17.68** | **0** |
| *N*-docosahexaenoyl GABA | 0.62 | 0.439 |  | **30.08** | **0** | **0.54** | **10.63** | **0** |
| *N*-palmitoyl glycine | 0.028 | 0.868 |  | **16** | **0.001** | **0.8** | **5.35** | **0.006** |
| *N*-stearoyl glycine | 0.747 | 0.396 |  | **19.63** | **0** | **0.73** | **6.62** | **0.002** |
| *N*-oleoyl glycine | 0.224 | 0.64 |  | **23.06** | **0** | **0.76** | **7.69** | **0.001** |
| *N*-linoleoyl glycine | 0.628 | 0.436 |  | **12.57** | **0.002** | **0.74** | **4.26** | **0.016** |
| *N*-arachidonoyl glycine | **3.23** | **0.086** | **1.07** | **17.68** | **0** | **0.83** | **6.69** | **0.002** |
| *N*-docosahexaenoyl glycine | 0.33 | 0.571 |  | **11.41** | **0.003** | **0.84** | **3.86** | **0.023** |
| *N*-palmitoyl leucine | 0.1 | 0.755 |  | 0.099 | 0.756 |  | 0.19 | 0.902 |
| *N*-stearoyl leucine | **6.49** | **0.018** | **1.08** | 1.62 | 0.216 |  | **2.53** | **0.082** |
| *N*-oleoyl leucine | 0.547 | 0.467 |  | 0.246 | 0.625 |  | 0.26 | 0.854 |
| *N*-linoleoyl leucine | 1.35 | 0.257 |  | 0.872 | 0.36 |  | 1.15 | 0.352 |
| *N*-docosahexaenoyl leucine | **15.07** | **0.001** | **0.79** | 0.062 | 0.806 |  | **5.08** | **0.008** |
| *N*-palmitoyl methionine | 1.84 | 0.188 |  | 0.16 | 0.693 |  | 0.71 | 0.556 |
| *N*-stearoyl methionine | **3.39** | **0.079** | **1.12** | 0.269 | 0.609 |  | 1.18 | 0.341 |
| *N*-oleoyl methionine | 0.392 | 0.537 |  | 0.398 | 0.534 |  | 0.518 | 0.674 |
| *N*-palmitoyl phenylalanine | 0.863 | 0.362 |  | **14.2** | **0.001** | **0.48** | **5.08** | **0.008** |
| *N*-stearoyl phenylalanine | 2.19 | 0.152 |  | **23.81** | **0** | **0.59** | **8.74** | **0** |
| *N*-oleoyl phenylalanine | 2.62 | 0.119 |  | **16.77** | **0** | **0.48** | **6.65** | **0.002** |
| *N*-linoleoyl phenylalanine | **15.48** | **0.001** | **1.61** | **13.39** | **0.001** | **0.62** | **10.33** | **0** |
| *N*-arachidonoyl phenylalanine | **39.62** | **0** | **1.67** | **37.51** | **0** | **0.59** | **26.27** | **0** |
| *N*-docosahexaenoyl phenylalanine | **7.83** | **0.01** | **1.32** | **4.06** | **0.056** | **0.78** | **6.78** | **0.002** |
| *N*-palmitoyl proline | 1.88 | 0.184 |  | 2.05 | 0.166 |  | 2.23 | 0.112 |
| *N*-stearoyl proline | **7.16** | **0.014** | **1.23** | 0.033 | 0.857 |  | **2.45** | **0.089** |
| *N-*oleoyl proline | 0.638 | 0.433 |  | 1.88 | 0.184 |  | 1.08 | 0.376 |
| *N*-palmitoyl serine | 0.591 | 0.45 |  | 1.82 | 0.191 |  | 0.922 | 0.446 |
| *N*-stearoyl serine | 0.015 | 0.902 |  | 0.585 | 0.452 |  | 0.792 | 0.511 |
| *N*-oleoyl serine | 0.019 | 0.892 |  | 0.004 | 0.95 |  | 0.584 | 0.632 |
| *N*-linoleoyl serine | 1.44 | 0.243 |  | **30.31** | **0** | **0.71** | **11.08** | **0** |
| *N*-arachidonoyl serine | **58.29** | **0** | **1.56** | **9.68** | **0.005** | **0.88** | **21.86** | **0** |
| *N*-docosahexaenoyl serine | **10.6** | **0.003** | **1.21** | **4.98** | **0.055** | **0.89** | **5.02** | **0.008** |
| *N*-palmitoyl taurine | 0.024 | 0.877 |  | **3.26** | **0.084** | **0.93** | 1.55 | 0.228 |
| *N*-stearoyl taurine | 3.07 | 0.093 |  | 0.366 | 0.551 |  | 1.52 | 0.235 |
| *N-*oleoyl taurine | 0.657 | 0.426 |  | 1.81 | 0.192 |  | **2.47** | **0.087** |
| *N*-arachidonoyl taurine | **5.37** | **0.03** | **1.16** | **3.47** | **0.075** | **0.91** | **4.01** | **0.02** |
| *N*-palmitoyl tryptophan | 0.292 | 0.594 |  | 0.004 | 0.951 |  | 0.113 | 0.952 |
| *N*-stearoyl tryptophan | **3.53** | **0.073** | **1.17** | 0.086 | 0.772 |  | 1.53 | 0.233 |
| *N*-oleoyl tryptophan | 2.05 | 0.166 |  | 0.039 | 0.845 |  | 0.69 | 0.568 |
| *N*-palmitoyl tyrosine | 1.54 | 0.227 |  | **11.16** | **0.003** | **0.56** | **4.37** | **0.014** |
| *N*-stearoyl tyrosine | 1.42 | 0.245 |  | **13.2** | **0.001** | **0.67** | **5.64** | **0.005** |
| *N-*oleoyl tyrosine | 2.2 | 0.152 |  | **5.93** | **0.023** | **0.59** | **3.53** | **0.031** |
| *N*-arachidonoyl tyrosine | **24.08** | **0** | **1.61** | **26.54** | **0** | **0.6** | **16.9** | **0** |
| *N*-docosahexaenoyl tyrosine | **23.82** | **0** | **1.4** | **44.73** | **0** | **0.63** | **21.19** | **0** |
| *N*-palmitoyl valine | 0.835 | 0.37 |  | 1.47 | 0.238 |  | **3.12** | **0.046** |
| *N*- stearoyl valine | **4.72** | **0.04** | **1.17** | **4.8** | **0.039** | **0.87** | **3.44** | **0.034** |
| *N*-oleoyl valine | 0.335 | 0.568 |  | 0.153 | 0.7 |  | 0.535 | 0.663 |
| 2-palmitoyl glycerol | **8.26** | **0.009** | **0.83** | **22.66** | **0.002** | **1.44** | **9.71** | **0** |
| 2-oleoyl glycerol | **4.09** | **0.055** | **0.89** | **17.93** | **0** | **1.4** | **7.2** | **0.001** |
| 2-linoleoyl glycerol | 0.952 | 0.339 |  | 0.249 | 0.622 |  | 0.374 | 0.773 |
| 2-arachidonoyl glycerol | 0.151 | 0.701 |  | 2.2 | 0.152 |  | 0.825 | 0.493 |
| Oleic acid | **3.32** | **0.082** | **1.06** | **3.69** | **0.067** | **0.93** | **2.38** | **0.096** |
| Linoleic acid | 0.533 | 0.473 |  | **8.44** | **0.008** | **0.87** | **2.96** | **0.053** |
| Arachidonic acid | **12.3** | **0.002** | **1.11** | **4** | **0.057** | **0.95** | **5.12** | **0.007** |
| phosphoLEA | 2.25 | 0.148 |  | 0.875 | 0.359 |  | 1.37 | 0.277 |
| PGE_2_ | **32.96** | **0** | **1.34** | 2.32 | 0.141 |  | **11.59** | **0** |
| PGF_2α_ | **43.65** | **0** | **1.35** | 1.95 | 0.176 |  | **14.83** | **0** |
| 6-ketoPGF_1α_ | **33.8** | **0** | **1.63** | **8.34** | **0.008** | **0.82** | **13.2** | **0** |
| Sample Mass | 1.85 | 0.187 |  | 2.17 | 0.154 |  | **2.62** | **0.075** |

Supplemental Table 75: Output from ANOVA for hypothalamus

|  | Effect of genotype | | | Effect of age | | | Genotype x age | |
| --- | --- | --- | --- | --- | --- | --- | --- | --- |
| Analyte | F (1,20) | p | Mag | F (1,20) | p | Mag | F (3,20) | p |
| *N*-palmitoyl alanine | 1.9 | 0.183 |  | **10.78** | **0.004** | **0.82** | **4.54** | **0.014** |
| *N*-stearoyl alanine | 0.832 | 0.373 |  | 2.37 | 0.14 |  | 1.28 | 0.309 |
| *N*-oleoyl alanine | **21.19** | **0** | **0.75** | 0.037 | 0.849 |  | **7.29** | **0.002** |
| *N*-arachidonoyl alanine | 0.463 | 0.504 |  | 1.83 | 0.192 |  | 0.868 | 0.474 |
| *N*-palmitoyl ethanolamine | **5.38** | **0.031** | **0.76** | **3.22** | **0.088** | **0.81** | **3.18** | **0.046** |
| *N*-stearoyl ethanolamine | 0.768 | 0.391 |  | 1.55 | 0.227 |  | 0.958 | 0.432 |
| *N*-oleoyl ethanolamine | 1.93 | 0.18 |  | **3.93** | **0.061** | **0.76** | 2.12 | 0.129 |
| *N*-linoleoyl ethanolamine | 0.187 | 0.67 |  | **10.62** | **0.004** | **0.63** | **3.71** | **0.029** |
| *N*-arachidonoyl ethanolamine | **3.66** | **0.07** | **1.27** | **6.69** | **0.018** | **0.71** | **3.25** | **0.043** |
| *N*-docosahexaenoyl ethanolamine | 3.41 | 0.08 |  | **7.1** | **0.015** | **0.79** | **3.26** | **0.043** |
| *N*-palmitoyl GABA | **9.04** | **0.007** | **0.76** | **4.95** | **0.038** | **0.81** | **5.13** | **0.009** |
| *N*-stearoyl GABA | **8.01** | **0.01** | **0.8** | **5.79** | **0.026** | **0.82** | **6.17** | **0.004** |
| *N*-oleoyl GABA | **6.18** | **0.022** | **0.77** | 1.65 | 0.213 |  | **3.04** | **0.053** |
| *N*-arachidonoyl GABA | 0.711 | 0.409 |  | **3.7** | **0.069** | **0.81** | 1.6 | 0.222 |
| *N*-docosahexaenoyl GABA | 2.91 | 0.104 |  | **4.31** | **0.051** | **0.75** | **2.64** | **0.077** |
| *N*-palmitoyl glycine | **5.49** | **0.03** | **0.83** | **3.81** | **0.065** | **0.85** | **3.42** | **0.037** |
| *N*-stearoyl glycine | **5.16** | **0.034** | **0.81** | 1.84 | 0.19 |  | **2.6** | **0.081** |
| *N*-oleoyl glycine | **4.75** | **0.041** | **0.83** | **7.22** | **0.014** | **0.79** | **4.48** | **0.015** |
| *N*-linoleoyl glycine | 2.86 | 0.106 |  | **3.86** | **0.063** | **0.78** | **2.47** | **0.091** |
| *N*-arachidonoyl glycine | 0.006 | 0.937 |  | **8.32** | **0.009** | **0.8** | **2.79** | **0.067** |
| *N*-docosahexaenoyl glycine | 0.196 | 0.663 |  | **3.75** | **0.067** | **0.83** | 2.14 | 0.127 |
| *N*-palmitoyl leucine | 1.08 | 0.312 |  | **6.76** | **0.017** | **0.83** | **2.81** | **0.066** |
| *N*-stearoyl leucine | 0.18 | 0.676 |  | **3.37** | **0.081** | **0.86** | 1.45 | 0.26 |
| *N*-oleoyl leucine | 0.78 | 0.387 |  | 2.4 | 0.137 |  | 1.35 | 0.288 |
| *N*-docosahexaenoyl leucine | **12.63** | **0.002** | **1.45** | **6.94** | **0.016** | **0.77** | **6.74** | **0.003** |
| *N*-palmitoyl methionine | 0.389 | 0.54 |  | 0.189 | 0.668 |  | 0.246 | 0.863 |
| *N*-stearoyl methionine | 0.485 | 0.494 |  | 0.167 | 0.687 |  | 0.84 | 0.488 |
| *N*-palmitoyl phenylalanine | 0.095 | 0.76 |  | **3.66** | **0.07** | **0.79** | 2.11 | 0.131 |
| *N*-stearoyl phenylalanine | 1.29 | 0.269 |  | **11.43** | **0.003** | **0.76** | **5.17** | **0.008** |
| *N*-oleoyl phenylalanine | **6.67** | **0.018** | **0.78** | 0.402 | 0.533 |  | **3.09** | **0.05** |
| *N*-arachidonoyl phenylalanine | 1.2 | 0.286 |  | **3.5** | **0.076** | **0.72** | **2.6** | **0.081** |
| *N*-palmitoyl proline | 0.678 | 0.42 |  | 0.393 | 0.538 |  | 0.451 | 0.719 |
| *N*-stearoyl proline | 0.304 | 0.587 |  | **3.8** | **0.065** | **0.81** | 2.02 | 0.144 |
| *N*-palmitoyl serine | **11.27** | **0.003** | **0.77** | 0.32 | 0.578 |  | **4** | **0.022** |
| *N*-stearoyl serine | **1.47** | **0.24** | **0.84** | 0.216 | 0.647 |  | 0.599 | 0.623 |
| *N*-oleoyl serine | 6.34 | 0.02 |  | 0.029 | 0.866 |  | 2.27 | 0.111 |
| *N*-arachidonoyl serine | 0.876 | 0.361 |  | 0.733 | 0.402 |  | 0.497 | 0.689 |
| *N*-docosahexaenoyl serine | 0.113 | 0.74 |  | **5.41** | **0.031** | **0.68** | 1.91 | 0.161 |
| *N*-palmitoyl taurine | **28.73** | **0** | **0.71** | 0.221 | 0.643 |  | **9.86** | **0** |
| *N*-stearoyl taurine | **8.68** | **0.008** | **0.76** | 1.98 | 0.175 |  | **4.57** | **0.014** |
| *N-*oleoyl taurine | **7.82** | **0.011** | **0.78** | **4.93** | **0.038** | **0.82** | **5.14** | **0.009** |
| *N*-arachidonoyl taurine | 0.936 | 0.345 |  | 0.009 | 0.926 |  | 2.07 | 0.136 |
| *N*-stearoyl tryptophan | 0.029 | 0.866 |  | 0.012 | 0.915 |  | 0.048 | 0.986 |
| *N*-palmitoyl tyrosine | 2.86 | 0.106 |  | 0.143 | 0.71 |  | 1.04 | 0.396 |
| *N*-palmitoyl valine | 2.1 | 0.163 |  | 0.435 | 0.517 |  | 1.07 | 0.384 |
| *N*- stearoyl valine | 0.431 | 0.519 |  | 0.342 | 0.565 |  | 0.241 | 0.867 |
| 2-palmitoyl glycerol | 1.13 | 0.3 |  | **27.33** | **0** | **0.58** | **9.97** | **0** |
| 2-oleoyl glycerol | 1.33 | 0.263 |  | **7.35** | **0.013** | **0.73** | **3.95** | **0.023** |
| 2-linoleoyl glycerol | 0.019 | 0.892 |  | **13.01** | **0.002** | **0.62** | **4.93** | **0.01** |
| 2-arachidonoyl glycerol | 0.437 | 0.516 |  | **6.7** | **0.018** | **0.76** | **4.16** | **0.019** |
| Oleic acid | **4.21** | **0.054** | **0.85** | **6.06** | **0.023** | **0.83** | **3.74** | **0.028** |
| Linoleic acid | 1.49 | 0.237 |  | **19.21** | **0** | **0.78** | **8.11** | **0.001** |
| Arachidonic acid | 1.29 | 0.27 |  | **5.66** | **0.027** | **0.86** | **2.54** | **0.086** |
| phosphoLEA | 0.067 | 0.799 |  | 0.286 | 0.599 |  | 0.154 | 0.926 |
| PGE_2_ | **10.16** | **0.005** | **1.25** | 0.025 | 0.876 |  | **4.41** | **0.016** |
| PGF_2α_ | **8.11** | **0.01** | **1.24** | 0 | 0.995 |  | **3.35** | **0.04** |
| Sample Mass | 1.45 | 0.243 |  | 0.245 | 0.63 |  | 0.756 | 0.532 |

\

Supplemental Table 76: Output from ANOVA for midbrain

|  | Effect of genotype | | | Effect of age | | | Genotype x age | |
| --- | --- | --- | --- | --- | --- | --- | --- | --- |
| Analyte | F (1,23) | p | Mag | F (1,23) | p | Mag | F (3,23) | p |
| *N*-palmitoyl alanine | 1.77 | 0.197 |  | 0 | 0.99 |  | 0.846 | 0.483 |
| *N*-stearoyl alanine | **6.32** | **0.019** | **1.12** | 0.564 | 0.46 |  | **2.43** | **0.091** |
| *N*-oleoyl alanine | 2.05 | 0.166 |  | 0.944 | 0.341 |  | 1.87 | 0.163 |
| *N*-arachidonoyl alanine | **10.86** | **0.003** | **1.33** | 0.146 | 0.706 |  | **3.63** | **0.028** |
| *N*-palmitoyl ethanolamine | 0.861 | 0.363 |  | **29.41** | **0** | **1.59** | **9.88** | **0** |
| *N*-stearoyl ethanolamine | 3.38 | 0.144 |  | **56.89** | **0** | **4** | **21.07** | **0** |
| *N*-oleoyl ethanolamine | 0.571 | 0.457 |  | **23.59** | **0** | **1.59** | **8.48** | **0.001** |
| *N*-linoleoyl ethanolamine | **4.46** | **0.046** | **1.13** | **5.26** | **0.031** | **0.86** | **3.62** | **0.028** |
| *N*-arachidonoyl ethanolamine | **26.5** | **0** | **1.42** | 0.343 | 0.564 |  | **8.87** | **0** |
| *N*-docosahexaenoyl ethanolamine | 0.202 | 0.658 |  | 0.001 | 0.971 |  | 0.146 | 0.931 |
| *N*-palmitoyl GABA | **4.33** | **0.049** | **0.84** | **50.06** | **0** | **0.63** | **19.18** | **0** |
| *N*-stearoyl GABA | 0.417 | 0.525 |  | **34.58** | **0** | **0.65** | **12.06** | **0** |
| *N*-oleoyl GABA | 1.67 | 0.209 |  | **59.18** | **0** | **0.57** | **21.06** | **0** |
| *N*-linoleoyl GABA | 0.008 | 0.931 |  | **34.5** | **0** | **0.45** | **11.72** | **0** |
| *N*-arachidonoyl GABA | **13.51** | **0.001** | **1.18** | **84.7** | **0** | **0.58** | **31.96** | **0** |
| *N*-docosahexaenoyl GABA | 0.163 | 0.69 |  | **24.22** | **0** | **0.59** | **8.48** | **0.001** |
| *N*-palmitoyl glycine | 0.886 | 0.356 |  | 1.87 | 0.185 |  | 1 | 0.411 |
| *N*-stearoyl glycine | 1.83 | 0.19 |  | **23.44** | **0** | **0.82** | **8.15** | **0.001** |
| *N*-oleoyl glycine | 0.015 | 0.902 |  | **10.52** | **0.004** | **0.89** | **3.53** | **0.031** |
| *N*-linoleoyl glycine | 0.412 | 0.527 |  | **10.23** | **0.004** | **0.84** | **3.69** | **0.026** |
| *N*-arachidonoyl glycine | **12.45** | **0.002** | **1.12** | **7.24** | **0.013** | **0.92** | **6.04** | **0.003** |
| *N*-docosahexaenoyl glycine | 0.683 | 0.417 |  | **11.86** | **0.002** | **0.89** | **4.24** | **0.016** |
| *N*-palmitoyl leucine | 0.18 | 0.675 |  | 1.68 | 0.207 |  | 0.597 | 0.623 |
| *N*-stearoyl leucine | 0.09 | 0.77 |  | **3.16** | **0.089** | **0.92** | 1.51 | 0.238 |
| *N*-oleoyl leucine | 0.203 | 0.656 |  | 0.001 | 0.981 |  | 0.449 | 0.721 |
| *N*-linoleoyl leucine | 0.001 | 0.971 |  | **11.13** | **0.003** | **0.71** | **5.39** | **0.006** |
| *N*-docosahexaenoyl leucine | **14.19** | **0.001** | **0.75** | **3.09** | **0.092** | **0.85** | **8.08** | **0.001** |
| *N*-palmitoyl methionine | 0.191 | 0.666 |  | 0.378 | 0.545 |  | 0.26 | 0.853 |
| *N*-stearoyl methionine | 0.007 | 0.935 |  | 1.74 | 0.2 |  | 0.917 | 0.448 |
| *N*-oleoyl methionine | 0.162 | 0.991 |  | 0.063 | 0.805 |  | 0.093 | 0.963 |
| *N*-palmitoyl phenylalanine | **5.19** | **0.032** | **1.2** | **3.7** | **0.067** | **0.82** | **5.29** | **0.006** |
| *N*-stearoyl phenylalanine | **4.22** | **0.051** | **1.2** | **4.45** | **0.046** | **0.81** | **3.1** | **0.047** |
| *N*-oleoyl phenylalanine | **4.09** | **0.055** | **1.24** | **3.05** | **0.094** | **0.8** | **3.08** | **0.048** |
| *N*-linoleoyl phenylalanine | 0.272 | 0.607 |  | 0.393 | 0.537 |  | 0.204 | 0.893 |
| *N*-arachidonoyl phenylalanine | **33.04** | **0** | **1.83** | 0.671 | 0.421 |  | **12.12** | **0** |
| *N*-docosahexaenoyl phenylalanine | **3.16** | **0.089** | **1.29** | 0.278 | 0.603 |  | 2.09 | 0.13 |
| *N*-palmitoyl proline | 1.42 | 0.246 |  | 0.974 | 0.334 |  | 1.03 | 0.398 |
| *N*-stearoyl proline | 0.877 | 0.359 |  | 0.106 | 0.748 |  | 0.404 | 0.752 |
| *N-*oleoyl proline | 0.225 | 0.64 |  | 1.75 | 0.199 |  | 1.54 | 0.231 |
| *N*-palmitoyl serine | **4.2** | **0.052** | **0.92** | **19.14** | **0** | **1.21** | **7.59** | **0.001** |
| *N*-stearoyl serine | 0.731 | 0.401 |  | **22.25** | **0** | **1.19** | **8.87** | **0** |
| *N*-oleoyl serine | 2.85 | 0.105 |  | **22.67** | **0** | **1.13** | **8.77** | **0** |
| *N*-linoleoyl serine | 0.671 | 0.421 |  | 0.065 | 0.801 |  | 1.16 | 0.348 |
| *N*-arachidonoyl serine | **67.02** | **0** | **1.77** | **7.78** | **0.01** | **1.27** | **28.29** | **0** |
| *N*-docosahexaenoyl serine | 0.072 | 0.791 |  | **4.64** | **0.042** | **1.14** | **2.15** | **0.122** |
| *N*-palmitoyl taurine | **3.98** | **0.058** | **0.91** | 0.502 | 0.486 |  | 1.62 | 0.212 |
| *N*-stearoyl taurine | 0.84 | 0.369 |  | 0.257 | 0.617 |  | 0.474 | 0.703 |
| *N-*oleoyl taurine | 1.69 | 0.206 |  | 0.03 | 0.865 |  | 0.678 | 0.575 |
| *N*-arachidonoyl taurine | **9.21** | **0.006** | **1.2** | 2.02 | 0.169 |  | **3.51** | **0.031** |
| *N*-palmitoyl tryptophan | 1.871 | 0.185 |  | 0.136 | 0.715 |  | 1.26 | 0.313 |
| *N*-stearoyl tryptophan | 1.88 | 0.184 |  | 0.325 | 0.574 |  | 0.697 | 0.563 |
| *N*-palmitoyl tyrosine | **6.39** | **0.019** | **1.31** | 1.04 | 0.318 |  | **3.28** | **0.039** |
| *N*-stearoyl tyrosine | **6.98** | **0.015** | **1.28** | 0.783 | 0.385 |  | **2.74** | **0.067** |
| *N-*oleoyl tyrosine | **3.21** | **0.087** | **1.22** | 2 | 0.171 |  | **2.42** | **0.092** |
| *N*-arachidonoyl tyrosine | **52.7** | **0** | **1.98** | **7.57** | **0.011** | **0.81** | **20.25** | **0** |
| *N*-docosahexaenoyl tyrosine | **6.94** | **0.015** | **1.36** | 0.941 | 0.342 |  | **2.81** | **0.062** |
| *N*-palmitoyl valine | **3.21** | **0.086** | **1.09** | 0.297 | 0.591 |  | **3.11** | **0.046** |
| *N*- stearoyl valine | 0.276 | 0.605 |  | 0.733 | 0.401 |  | 0.746 | 0.536 |
| *N*-oleoyl valine | 0.023 | 0.88 |  | 0.018 | 0.894 |  | 0.207 | 0.89 |
| 2-palmitoyl glycerol | **8.79** | **0.007** | **0.82** | **4.49** | **0.045** | **1.12** | **4.68** | **0.011** |
| 2-oleoyl glycerol | **9.12** | **0.006** | **0.88** | **21.13** | **0** | **1.22** | **9.61** | **0** |
| 2-linoleoyl glycerol | 0.53 | 0.474 |  | **6.48** | **0.018** | **0.9** | **3** | **0.052** |
| 2-arachidonoyl glycerol | **30.43** | **0** | **1.3** | 1.07 | 0.312 |  | **10.95** | **0** |
| Oleic acid | 0.225 | 0.64 |  | **18.36** | **0** | **0.82** | **6.54** | **0.002** |
| Linoleic acid | 6.08 | 0.022 |  | **44.36** | **0** | **0.77** | **15.95** | **0** |
| Arachidonic acid | 14.62 | 0.001 |  | **11.96** | **0.002** | **0.88** | **8.17** | **0.001** |
| phosphoLEA | **23.29** | **0** | **1.39** | 1.12 | 0.3 |  | **8.56** | **0.001** |
| PGE_2_ | **22.15** | **0** | **1.46** | **5.84** | **0.024** | **0.84** | **8.78** | **0** |
| PGF_2α_ | **96.51** | **0** | **1.52** | **16.79** | **0** | **0.86** | **36** | **0** |
| 6-ketoPGF_1α_ | **22.62** | **0** | **1.73** | 2.82 | 0.107 |  | **8.07** | **0.001** |
| Sample Mass | **9.39** | **0.006** | **0.9** | 0.783 | 0.385 |  | **4.04** | **0.019** |

Supplemental Table 77: Output from ANOVA for brainstem

|  | Effect of genotype | | | Effect of age | | | Genotype x age | |
| --- | --- | --- | --- | --- | --- | --- | --- | --- |
| Analyte | F (1,23) | p | Mag | F (1,23) | p | Mag | F (3,23) | p |
| *N*-palmitoyl alanine | 2.07 | 0.164 |  | **14.05** | **0.001** | **0.82** | **5.09** | **0.008** |
| *N*-stearoyl alanine | 1.81 | 0.192 |  | **4.96** | **0.036** | **0.89** | 2.19 | 0.116 |
| *N*-oleoyl alanine | 0.318 | 0.578 |  | **3.66** | **0.068** | **0.89** | **3.7** | **0.026** |
| *N*-linoleoyl alanine | 0.267 | 0.61 |  | 0.962 | 0.337 |  | **2.84** | **0.06** |
| *N*-arachidonoyl alanine | **6.3** | **0.02** | **1.33** | **12.1** | **0.002** | **0.66** | **5.65** | **0.005** |
| *N*-docosahexaenoyl alanine | 2.25 | 0.148 |  | **7.69** | **0.011** | **0.72** | **4.3** | **0.015** |
| *N*-palmitoyl ethanolamine | **3.59** | **0.071** | **0.88** | **19.66** | **0** | **1.34** | **10.14** | **0** |
| *N*-stearoyl ethanolamine | 0.069 | 0.795 |  | **29.15** | **0** | **2.78** | **10.06** | **0** |
| *N*-oleoyl ethanolamine | 0.002 | 0.963 |  | **9.14** | **0.006** | **1.28** | **5.04** | **0.008** |
| *N*-linoleoyl ethanolamine | **5.73** | **0.025** | **1.18** | **4.21** | **0.052** | **0.84** | **4.75** | **0.01** |
| *N*-arachidonoyl ethanolamine | **29.19** | **0** | **1.52** | 0.342 | 0.564 |  | **10.34** | **0** |
| *N*-docosahexaenoyl ethanolamine | 0.113 | 0.74 |  | 0.012 | 0.914 |  | 0.416 | 0.743 |
| *N*-palmitoyl GABA | **3.85** | **0.062** | **0.85** | **81.86** | **0** | **0.59** | **30.89** | **0** |
| *N*-stearoyl GABA | 2.34 | 0.14 |  | **90.74** | **0** | **0.67** | **34.41** | **0** |
| *N*-oleoyl GABA | 0.945 | 0.341 |  | **82.49** | **0** | **0.56** | **31.57** | **0** |
| *N*-linoleoyl GABA | 0.408 | 0.529 |  | **34.58** | **0** | **0.52** | **12.18** | **0** |
| *N*-arachidonoyl GABA | **37.23** | **0** | **1.25** | **121.06** | **0** | **0.62** | **51.06** | **0** |
| *N*-docosahexaenoyl GABA | 0.063 | 0.804 |  | **41.96** | **0** | **0.58** | **14.99** | **0** |
| *N*-palmitoyl glycine | 0.657 | 0.426 |  | **4.2** | **0.052** | **0.93** | **1.95** | **0.15** |
| *N*-stearoyl glycine | 0.995 | 0.329 |  | **12.47** | **0.002** | **0.82** | **4.78** | **0.01** |
| *N*-oleoyl glycine | 0.035 | 0.854 |  | **10.27** | **0.004** | **0.86** | **4.09** | **0.018** |
| *N*-linoleoyl glycine | 1.25 | 0.275 |  | **10.22** | **0.004** | **0.86** | **4.37** | **0.014** |
| *N*-arachidonoyl glycine | **17.49** | **0** | **1.11** | **5.59** | **0.027** | **0.95** | **7.16** | **0.001** |
| *N*-docosahexaenoyl glycine | 0.007 | 0.935 |  | **4.61** | **0.043** | **0.93** | **1.93** | **0.153** |
| *N*-palmitoyl leucine | 2.11 | 0.16 |  | 0.977 | 0.333 |  | 1.45 | 0.255 |
| *N*-stearoyl leucine | 0.02 | 0.889 |  | **5.08** | **0.034** | **0.85** | 1.71 | 0.192 |
| *N*-oleoyl leucine | 0.032 | 0.86 |  | **3.67** | **0.068** | **0.86** | 1.39 | 0.271 |
| *N*-linoleoyl leucine | 0 | 0.995 |  | 0.799 | 0.381 |  | 0.459 | 0.714 |
| *N*-docosahexaenoyl leucine | 2.01 | 0.17 |  | 1.92 | 0.179 |  | 1.27 | 0.309 |
| *N*-palmitoyl methionine | 0.002 | 0.969 |  | 1.54 | 0.228 |  | 0.891 | 0.461 |
| *N*-stearoyl methionine | 1.52 | 0.23 |  | **3.31** | **0.082** | **0.9** | 1.54 | 0.23 |
| *N*-oleoyl methionine | 0.61 | 0.444 |  | **14.84** | **0.001** | **0.66** | **5.39** | **0.006** |
| *N*-palmitoyl phenylalanine | 1.52 | 0.23 |  | 2.53 | 0.126 |  | 1.51 | 0.238 |
| *N*-stearoyl phenylalanine | **4.99** | **0.036** | **1.23** | **3.55** | **0.072** | **1.2** | **3.19** | **0.043** |
| *N*-oleoyl phenylalanine | 2.63 | 0.119 |  | **4.04** | **0.056** | **1.17** | **2.48** | **0.087** |
| *N*-arachidonoyl phenylalanine | **8.99** | **0** | **1.51** | 0.74 | 0.399 |  | **3.54** | **0.03** |
| *N*-docosahexaenoyl phenylalanine | **5** | **0.035** | **1.32** | 0.036 | 0.85 |  | 1.86 | 0.164 |
| *N*-palmitoyl proline | 0.456 | 0.506 |  | 0.895 | 0.354 |  | 0.623 | 0.608 |
| *N*-stearoyl proline | 1.11 | 0.304 |  | 1.05 | 0.316 |  | 0.857 | 0.477 |
| *N-*oleoyl proline | 1.54 | 0.227 |  | 0.878 | 0.358 |  | 1.15 | 0.348 |
| *N*-palmitoyl serine | **37.7** | **0** | **0.9** | **42.49** | **0** | **1.12** | **24.51** | **0** |
| *N*-stearoyl serine | 0.664 | 0.423 |  | **15.17** | **0.001** | **1.12** | **5.44** | **0.006** |
| *N*-oleoyl serine | **23.63** | **0** | **0.88** | **6.99** | **0.015** | **1.06** | **10.05** | **0** |
| *N*-linoleoyl serine | **8.78** | **0.007** | **1.17** | **5.66** | **0.026** | **0.87** | **6.59** | **0.002** |
| *N*-arachidonoyl serine | **39.62** | **0** | **1.48** | 0.07 | 0.794 |  | **13.62** | **0** |
| *N*-docosahexaenoyl serine | **6.95** | **0.015** | **1.16** | 0.094 | 0.762 |  | **2.38** | **0.096** |
| *N*-palmitoyl taurine | **36.66** | **0** | **0.87** | **10.26** | **0.004** | **1.08** | **15.11** | **0** |
| *N*-stearoyl taurine | **26.15** | **0** | **0.85** | **15.56** | **0.001** | **1.13** | **12.79** | **0** |
| *N-*oleoyl taurine | **4.6** | **0.043** | **0.93** | 2.18 | 0.154 |  | **2.65** | **0.073** |
| *N*-arachidonoyl taurine | **16.75** | **0** | **1.21** | 0.91 | 0.35 |  | **6.53** | **0.002** |
| *N*-palmitoyl tryptophan | 0.031 | 0.861 |  | 2.8 | 0.108 |  | 1.62 | 0.211 |
| *N*-stearoyl tryptophan | 1.11 | 0.303 |  | 1.22 | 0.28 |  | 1.44 | 0.258 |
| *N*-oleoyl tryptophan | **35.65** | **0** | **2.09** | 2.65 | 0.117 |  | **12.91** | **0** |
| *N*-palmitoyl tyrosine | 2.23 | 0.141 |  | 1.47 | 0.237 |  | 2.09 | 0.129 |
| *N*-stearoyl tyrosine | **3.88** | **0.061** | **1.22** | 0.019 | 0.892 |  | **2.87** | **0.059** |
| *N-*oleoyl tyrosine | 0.009 | 0.925 |  | 1.24 | 0.277 |  | **5.64** | **0.005** |
| *N*-arachidonoyl tyrosine | **46.82** | **0** | **1.62** | **5.13** | **0.033** | **0.88** | **16.9** | **0** |
| *N*-docosahexaenoyl tyrosine | **26.7** | **0** | **1.59** | 0.567 | 0.459 |  | **11.82** | **0** |
| *N*-palmitoyl valine | 0.001 | 0.975 |  | **8.38** | **0.008** | **0.82** | **2.96** | **0.053** |
| *N*- stearoyl valine | **8.75** | **0.007** | **1.21** | 1.37 | 0.254 |  | **3.78** | **0.024** |
| *N*-oleoyl valine | **6.92** | **0.015** | **1.22** | 0 | 0.987 |  | **2.46** | **0.088** |
| 2-palmitoyl glycerol | 2.66 | 0.116 |  | 0.609 | 0.443 |  | **3.85** | **0.023** |
| 2-oleoyl glycerol | 0.793 | 0.382 |  | **6.84** | **0.015** | **1.11** | **4.84** | **0.009** |
| 2-linoleoyl glycerol | 2.23 | 0.149 |  | **9.68** | **0.005** | **0.84** | **6.25** | **0.003** |
| 2-arachidonoyl glycerol | **22.61** | **0** | **1.28** | 0.018 | 0.894 |  | **8.21** | **0.001** |
| Oleic acid | 0.116 | 0.736 |  | **12.72** | **0.002** | **0.74** | **4.64** | **0.011** |
| Linoleic acid | 0.188 | 0.668 |  | **31.23** | **0** | **0.69** | **10.84** | **0** |
| Arachidonic acid | **10.1** | **0.004** | **1.19** | **18.18** | **0** | **0.77** | **9.26** | **0** |
| phosphoLEA | **16.67** | **0** | **1.36** | 1.46 | 0.239 |  | **6.85** | **0.002** |
| PGE_2_ | **56.3** | **0** | **1.45** | 0.165 | 0.689 |  | **19.81** | **0** |
| PGF_2α_ | **44.57** | **0** | **1.39** | 0.51 | 0.482 |  | **15.58** | **0** |
| 6-ketoPGF_1α_ | **58.13** | **0** | **2.02** | **5.18** | **0.032** | **0.87** | **20.35** | **0** |
| Sample Mass | **5.66** | **0.026** | **0.92** | **4.69** | **0.041** | **1.08** | **3.18** | **0.043** |

Supplemental Table 78: Lipids that significantly changed in the younger adult ABHD12 KO striatum relative to the younger adult WT striatum

| Striatum | | | |
| --- | --- | --- | --- |
| Lipid | Direction of change in young ABHD12 KO relative to young WT | Magnitude of change in young ABHD12 KO relative to young WT | p |
| *N*-arachidonoyl alanine | ↑ | 1.29 | 0.039 |
| *N*-palmitoyl ethanolamine | ↓ | 0.73 | 0.01 |
| *N*-stearoyl ethanolamine | ↓ | 0.73 | 0.054 |
| *N*-docosahexaenoyl ethanolamine | ↓ | 0.85 | 0.087 |
| *N*-palmitoyl GABA | ↓ | 0.87 | 0.026 |
| *N*-docosahexaenoyl leucine | ↓ | 0.74 | 0.076 |
| *N*-stearoyl methionine | ↑↑ | 1.52 | 0.003 |
| *N*-stearoyl phenylalanine | ↑ | 1.28 | 0.062 |
| *N*-palmitoyl serine | ↓ | 0.79 | 0.005 |
| *N*-oleoyl serine | ↓ | 0.85 | 0.062 |
| *N*-arachidonoyl serine | ↑ | 1.22 | 0.014 |
| *N*-stearoyl tyrosine | ↑ | 1.37 | 0.072 |
| *N-*oleoyl tyrosine | ↑↑ | 1.62 | 0.059 |
| *N*-arachidonoyl tyrosine | ↑↑ | 1.62 | 0 |
| *N*- stearoyl valine | ↑ | 1.35 | 0.017 |
| 2-oleoyl glycerol | ↓ | 0.81 | 0.053 |
| 2-arachidonoyl glycerol | ↑ | 1.22 | 0.051 |
| PGE_2_ | ↑ | 1.23 | 0.004 |
| PGF_2α_ | ↑ | 1.34 | 0 |
| 6-ketoPGF_1α_ | ↑ | 1.47 | 0.032 |

Supplemental Table 79: Lipids that significantly changed in the older adult ABHD12 KO striatum relative to the older adult WT striatum

| Striatum | | | |
| --- | --- | --- | --- |
| Lipid | Direction of change in old ABHD12 KO relative to old WT | Magnitude of change in old ABHD12 KO relative to old WT | p |
| *N*-oleoyl alanine | ↓ | 0.81 | 0.014 |
| *N*-arachidonoyl alanine | ↑ | 1.34 | 0.004 |
| *N*-stearoyl ethanolamine | ↑ | 1.4 | 0.009 |
| *N*-arachidonoyl ethanolamine | ↑ | 1.37 | 0.078 |
| *N*-arachidonoyl GABA | ↑ | 1.32 | 0.012 |
| *N*-docosahexaenoyl GABA | ↓ | 0.77 | 0.021 |
| *N*-linoleoyl leucine | ↓↓ | 0.56 | 0.033 |
| *N*-stearoyl methionine | ↑ | 1.26 | 0.097 |
| *N*-oleoyl methionine | ↓ | 0.75 | 0.041 |
| *N*-arachidonoyl serine | ↑ | 1.39 | 0 |
| *N*-stearoyl taurine | ↑ | 1.2 | 0.091 |
| *N*-arachidonoyl taurine | ↑ | 1.25 | 0.03 |
| *N*-arachidonoyl tyrosine | ↑↑ | 1.7 | 0 |
| *N*-palmitoyl valine | ↓↓ | 0.58 | 0.034 |
| *N*- stearoyl valine | ↑ | 1.22 | 0.088 |
| 2-oleoyl glycerol | ↓ | 0.79 | 0.025 |
| 2-arachidonoyl glycerol | ↑ | 1.41 | 0.002 |
| PGE_2_ | ↑ | 1.24 | 0.002 |
| PGF_2α_ | ↑ | 1.3 | 0 |
| 6-ketoPGF_1α_ | ↑↑ | 1.64 | 0 |

Supplemental Table 80: Lipids that significantly changed in the older adult WT striatum relative to the younger adult WT striatum

| Striatum | | | |
| --- | --- | --- | --- |
| Lipid | Direction of change in old WT relative to young WT | Magnitude of change in old WT relative to young WT | p |
| *N*-oleoyl alanine | ↑ | 1.2 | 0.041 |
| *N*-linoleoyl ethanolamine | ↓ | 0.79 | 0.069 |
| *N*-docosahexaenoyl ethanolamine | ↓ | 0.82 | 0.051 |
| *N*-palmitoyl GABA | ↓↓ | 0.66 | 0 |
| *N*-stearoyl GABA | ↓ | 0.7 | 0 |
| *N*-oleoyl GABA | ↓ | 0.71 | 0.005 |
| *N*-arachidonoyl GABA | ↓↓ | 0.66 | 0 |
| *N*-docosahexaenoyl GABA | ↓ | 0.79 | 0.016 |
| *N*-oleoyl glycine | ↓ | 0.85 | 0.041 |
| *N*-linoleoyl glycine | ↓ | 0.74 | 0.042 |
| *N*-arachidonoyl glycine | ↓ | 0.85 | 0.028 |
| *N*-docosahexaenoyl serine | ↓ | 0.85 | 0.096 |
| *N*-palmitoyl taurine | ↓ | 0.81 | 0.028 |
| *N*-stearoyl taurine | ↓ | 0.79 | 0.045 |
| 2-palmitoyl glycerol | ↓ | 0.75 | 0.032 |
| Oleic acid | ↓ | 0.83 | 0.059 |
| 6-ketoPGF_1α_ | ↑ | 1.37 | 0.085 |

Supplemental Table 81: Lipids that significantly changed in the older adult ABHD12 KO striatum relative to the younger adult ABHD12 KO striatum

| Striatum | | | |
| --- | --- | --- | --- |
| Lipid | Direction of change in old ABHD12 KO relative to young ABHD12 KO | Magnitude of change in old ABHD12 KO relative to young ABHD12 KO | p |
| *N*-arachidonoyl alanine | ↑ | 1.16 | 0.096 |
| *N*-palmitoyl ethanolamine | ↑ | 1.31 | 0.02 |
| *N*-stearoyl ethanolamine | ↑↑ | 1.57 | 0.001 |
| *N*-oleoyl ethanolamine | ↑ | 1.26 | 0.057 |
| *N*-palmitoyl GABA | ↓ | 0.75 | 0.004 |
| *N*-stearoyl GABA | ↓ | 0.73 | 0.001 |
| *N*-oleoyl GABA | ↓↓ | 0.64 | 0 |
| *N*-arachidonoyl GABA | ↓ | 0.78 | 0.005 |
| *N*-docosahexaenoyl GABA | ↓ | 0.71 | 0.002 |
| *N*-stearoyl methionine | ↓ | 0.79 | 0.039 |
| *N*-palmitoyl phenylalanine | ↓ | 0.76 | 0.085 |
| *N*-stearoyl phenylalanine | ↓ | 0.79 | 0.056 |
| *N*-arachidonoyl phenylalanine | ↓ | 0.75 | 0.1 |
| *N*-docosahexaenoyl phenylalanine | ↓ | 0.73 | 0.063 |
| *N*-palmitoyl serine | ↑ | 1.14 | 0.078 |
| *N*-stearoyl tyrosine | ↓ | 0.74 | 0.057 |
| *N-*oleoyl tyrosine | ↓ | 0.69 | 0.094 |
| *N*-arachidonoyl tyrosine | ↓ | 0.82 | 0.03 |
| 2-palmitoy glycerol | ↓ | 0.73 | 0.024 |
| 2-linoleoyl glycerol | ↓ | 0.71 | 0.011 |
| Oleic acid | ↓ | 0.83 | 0.052 |
| Arachidonic acid | ↓ | 0.83 | 0.018 |
| PGF_2α_ | ↓ | 0.89 | 0.031 |
| 6-ketoPGF_1α_ | ↑↑ | 1.52 | 0 |

Supplemental Table 82: Lipids that significantly changed in the younger adult ABHD12 KO hippocampus relative to the younger adult WT hippocampus

| Hippocampus | | | |
| --- | --- | --- | --- |
| Lipid | Direction of change in young ABHD12 KO relative to young WT | Magnitude of change in young ABHD12 KO relative to young WT | p |
| *N*-arachidonoyl ethanolamine | ↑ | 1.29 | 0.01 |
| *N*-docosahexaenoyl ethanolamine | ↓ | 0.85 | 0.011 |
| *N*-palmitoyl GABA | ↓ | 0.88 | 0.093 |
| *N*-stearoyl methionine | ↑ | 1.27 | 0.035 |
| *N*-stearoyl phenylalanine | ↑ | 1.25 | 0.087 |
| *N*-oleoyl phenylalanine | ↑ | 1.31 | 0.019 |
| *N*-palmitoyl proline | ↑ | 1.39 | 0.018 |
| *N*-stearoyl proline | ↑ | 1.49 | 0.001 |
| *N*-palmitoyl serine | ↓ | 0.82 | 0.001 |
| *N*-oleoyl serine | ↓ | 0.91 | 0.099 |
| *N*-arachidonoyl serine | ↑↑ | 1.51 | 0.001 |
| *N*-stearoyl taurine | ↓ | 0.86 | 0.053 |
| *N-*oleoyl taurine | ↓ | 0.81 | 0.05 |
| *N*-palmitoyl tyrosine | ↑ | 1.42 | 0.091 |
| *N*-arachidonoyl tyrosine | ↑↑ | 1.86 | 0.001 |
| *N*-docosahexaenoyl tyrosine | ↑↑ | 1.89 | 0.019 |
| 2-oleoyl glycerol | ↓ | 0.77 | 0.009 |
| 2-arachidonoyl glycerol | ↑ | 1.27 | 0.039 |
| Arachidonic acid | ↑ | 1.12 | 0.048 |
| PGF_2α_ | ↑ | 1.42 | 0.003 |
| 6-ketoPGF_1α_ | ↑↑↑ | 2.26 | 0 |

Supplemental Table 83: Lipids that significantly changed in the older adult ABHD12 KO hippocampus relative to the older adult WT hippocampus

| Hippocampus | | | |
| --- | --- | --- | --- |
| Lipid | Direction of change in old ABHD12 KO relative to old WT | Magnitude of change in old ABHD12 KO relative to old WT | p |
| *N*-arachidonoyl ethanolamine | ↑ | 1.33 | 0.045 |
| *N*-docosahexaenoyl ethanolamine | ↓ | 0.81 | 0.012 |
| *N*-docosahexaenoyl leucine | ↓ | 0.67 | 0.001 |
| *N*-arachidonoyl phenylalanine | ↑ | 1.24 | 0.091 |
| *N*-arachidonoyl serine | ↑↑ | 1.51 | 0.002 |
| *N*-palmitoyl taurine | ↓ | 0.8 | 0.015 |
| *N*-arachidonoyl tyrosine | ↑ | 1.41 | 0.057 |
| 2-oleoyl glycerol | ↓ | 0.82 | 0.032 |
| 6-ketoPGF_1α_ | ↑↑ | 1.96 | 0.002 |

Supplemental Table 84: Lipids that significantly changed in the older adult WT hippocampus relative to the younger adult WT hippocampus

| Hippocampus | | | |
| --- | --- | --- | --- |
| Lipid | Direction of change in old WT relative to young WT | Magnitude of change in old WT relative to young WT | p |
| *N*-palmitoyl alanine | ↓↓ | 0.55 | 0 |
| *N*-stearoyl alanine | ↓↓ | 0.66 | 0 |
| *N*-oleoyl alanine | ↓ | 0.68 | 0 |
| *N*-linoleoyl alanine | ↓↓ | 0.63 | 0.01 |
| *N*-arachidonoyl alanine | ↓ | 0.67 | 0 |
| *N*-oleoyl ethanolamine | ↓ | 0.68 | 0.047 |
| *N*-linoleoyl ethanolamine | ↓↓ | 0.52 | 0 |
| *N*-arachidonoyl ethanolamine | ↓↓ | 0.6 | 0.001 |
| *N*-docosahexaenoyl ethanolamine | ↓ | 0.69 | 0 |
| *N*-palmitoyl GABA | ↓↓ | 0.59 | 0 |
| *N*-stearoyl GABA | ↓↓ | 0.63 | 0 |
| *N*-oleoyl GABA | ↓↓ | 0.6 | 0 |
| *N*-linoleoyl GABA | ↓↓ | 0.65 | 0.019 |
| *N*-arachidonoyl GABA | ↓↓ | 0.63 | 0 |
| *N*-docosahexaenoyl GABA | ↓↓ | 0.61 | 0 |
| *N*-palmitoyl glycine | ↓ | 0.83 | 0.008 |
| *N*-stearoyl glycine | ↓ | 0.8 | 0.01 |
| *N*-oleoyl glycine | ↓ | 0.79 | 0 |
| *N*-linoleoyl glycine | ↓ | 0.69 | 0.002 |
| *N*-arachidonoyl glycine | ↓ | 0.75 | 0.001 |
| *N*-docosahexaenoyl glycine | ↓ | 0.78 | 0.005 |
| *N*-palmitoyl leucine | ↓ | 0.89 | 0.09 |
| *N*-oleoyl leucine | ↓ | 0.79 | 0.035 |
| *N*-linoleoyl leucine | ↓ | 0.75 | 0.08 |
| *N*-linoleoyl serine | ↓↓ | 0.63 | 0.028 |
| *N*-palmitoyl taurine | ↑ | 1.17 | 0.094 |
| 2-linoleoyl glycerol | ↓ | 0.79 | 0.041 |
| Oleic acid | ↓ | 0.76 | 0.001 |
| Linoleic acid | ↓ | 0.74 | 0 |
| Arachidonic acid | ↓ | 0.77 | 0 |
| PGF_2α_ | ↑ | 1.26 | 0.062 |

Supplemental Table 85: Lipids that significantly changed in the older adult ABHD12 KO hippocampus relative to the younger adult ABHD12 KO hippocampus

| Hippocampus | | | |
| --- | --- | --- | --- |
| Lipid | Direction of change in old ABHD12 KO relative to young ABHD12 KO | Magnitude of change in old ABHD12 KO relative to young ABHD12 KO | p |
| *N*-palmitoyl alanine | ↓↓ | 0.54 | 0 |
| *N*-stearoyl alanine | ↓↓ | 0.65 | 0 |
| *N*-oleoyl alanine | ↓↓ | 0.63 | 0 |
| *N*-linoleoyl alanine | ↓↓ | 0.6 | 0.004 |
| *N*-arachidonoyl alanine | ↓ | 0.7 | 0 |
| *N*-stearoyl ethanolamine | ↑↑ | 1.68 | 0.046 |
| *N*-linoleoyl ethanolamine | ↓↓ | 0.54 | 0 |
| *N*-arachidonoyl ethanolamine | ↓↓ | 0.62 | 0 |
| *N*-docosahexaenoyl ethanolamine | ↓↓ | 0.66 | 0 |
| *N*-palmitoyl GABA | ↓↓ | 0.63 | 0 |
| *N*-stearoyl GABA | ↓↓ | 0.66 | 0 |
| *N*-oleoyl GABA | ↓↓ | 0.61 | 0 |
| *N*-linoleoyl GABA | ↓↓↓ | 0.48 | 0.001 |
| *N*-arachidonoyl GABA | ↓↓ | 0.62 | 0 |
| *N*-docosahexaenoyl GABA | ↓↓ | 0.61 | 0.001 |
| *N*-stearoyl glycine | ↓ | 0.79 | 0.003 |
| *N*-oleoyl glycine | ↓ | 0.82 | 0.002 |
| *N*-linoleoyl glycine | ↓ | 0.73 | 0.004 |
| *N*-arachidonoyl glycine | ↓ | 0.76 | 0 |
| *N*-docosahexaenoyl glycine | ↓ | 0.72 | 0 |
| *N*-palmitoyl leucine | ↓ | 0.81 | 0.004 |
| *N*-stearoyl leucine | ↓ | 0.81 | 0.004 |
| *N*-linoleoyl leucine | ↓↓ | 0.57 | 0 |
| *N*-docosahexaenoyl leucine | ↓ | 0.81 | 0.097 |
| *N*-stearoyl methionine | ↓ | 0.76 | 0.011 |
| *N*-palmitoyl phenylalanine | ↓ | 0.78 | 0.06 |
| *N*-stearoyl phenylalanine | ↓ | 0.81 | 0.077 |
| *N*-oleoyl phenylalanine | ↓ | 0.81 | 0.038 |
| *N*-docosahexaenoyl phenylalanine | ↓ | 0.69 | 0.02 |
| *N*-stearoyl proline | ↓ | 0.79 | 0.012 |
| *N*-palmitoyl serine | ↑ | 1.2 | 0.001 |
| *N*-oleoyl serine | ↑ | 1.17 | 0.005 |
| *N*-linoleoyl serine | ↓ | 0.72 | 0.027 |
| *N*-stearoyl taurine | ↑ | 1.22 | 0.006 |
| *N*-palmitoyl tyrosine | ↓ | 0.72 | 0.082 |
| *N*-stearoyl tyrosine | ↓ | 0.69 | 0.036 |
| *N*-arachidonoyl tyrosine | ↓ | 0.73 | 0.018 |
| *N*-docosahexaenoyl tyrosine | ↓ | 0.7 | 0.087 |
| 2-linoleoyl glycerol | ↓ | 0.78 | 0.022 |
| 2-arachidonoyl glycerol | ↓ | 0.79 | 0.03 |
| Oleic acid | ↓ | 0.71 | 0 |
| Linoleic acid | ↓ | 0.71 | 0 |
| Arachidonic acid | ↓ | 0.75 | 0 |
| PGF_2α_ | ↓ | 0.86 | 0.094 |
| 6-ketoPGF_1α_ | ↓ | 0.7 | 0.005 |
| Sample Mass | ↑ | 1.12 | 0.048 |

Supplemental Table 86: Lipids that significantly changed in the younger adult ABHD12 KO cerebellum relative to the younger adult WT cerebellum

| Cerebellum | | | |
| --- | --- | --- | --- |
| Lipid | Direction of change in young ABHD12 KO relative to young WT | Magnitude of change in young ABHD12 KO relative to young WT | p |
| *N*-arachidonoyl alanine | ↑ | 1.18 | 0.068 |
| *N*-palmitoyl ethanolamine | ↓ | 0.7 | 0.031 |
| *N*-linoleoyl ethanolamine | ↑ | 1.22 | 0.071 |
| *N*-arachidonoyl ethanolamine | ↑↑ | 1.77 | 0 |
| *N*-arachidonoyl GABA | ↑ | 1.18 | 0.023 |
| *N*-stearoyl leucine | ↑ | 1.14 | 0.072 |
| *N*-stearoyl methionine | ↑ | 1.47 | 0.025 |
| *N*-stearoyl phenylalanine | ↑ | 1.25 | 0.003 |
| *N*-oleoyl phenylalanine | ↑ | 1.34 | 0.089 |
| *N*-arachidonoyl phenylalanine | ↑↑↑ | 2.25 | 0 |
| *N*-palmitoyl serine | ↓ | 0.88 | 0.003 |
| *N*-oleoyl serine | ↓ | 0.86 | 0.008 |
| *N*-arachidonoyl serine | ↑ | 1.3 | 0.003 |
| *N*-palmitoyl taurine | ↓ | 0.81 | 0.001 |
| *N-*oleoyl tyrosine | ↑ | 1.46 | 0.083 |
| *N*-arachidonoyl tyrosine | ↑↑↑ | 2.37 | 0 |
| 2-palmitoyl glycerol | ↓ | 0.79 | 0.093 |
| 2-oleoyl glycerol | ↓ | 0.84 | 0.068 |
| 2-arachidonoyl glycerol | ↑ | 1.2 | 0.04 |
| Arachidonic acid | ↑ | 1.15 | 0.008 |
| PGE_2_ | ↑ | 1.23 | 0.03 |
| PGF_2α_ | ↑ | 1.32 | 0.006 |
| 6-ketoPGF_1α_ | ↑↑ | 1.72 | 0.001 |

Supplemental Table 87: Lipids that significantly changed in the older adult ABHD12 KO cerebellum relative to the older adult WT cerebellum

| Cerebellum | | | |
| --- | --- | --- | --- |
| Lipid | Direction of change in old ABHD12 KO relative to old WT | Magnitude of change in old ABHD12 KO relative to old WT | p |
| *N*-oleoyl alanine | ↓ | 0.71 | 0.002 |
| *N*-arachidonoyl alanine | ↑↑ | 1.55 | 0 |
| *N*-linoleoyl ethanolamine | ↑ | 1.24 | 0.031 |
| *N*-arachidonoyl ethanolamine | ↑↑ | 1.8 | 0 |
| *N*-palmitoyl leucine | ↓ | 0.87 | 0.023 |
| *N*-oleoyl leucine | ↓ | 0.83 | 0.072 |
| *N*-docosahexaenoyl leucine | ↓ | 0.72 | 0.002 |
| *N*-palmitoyl serine | ↓ | 0.89 | 0.001 |
| *N*-arachidonoyl serine | ↑ | 1.42 | 0 |
| *N*-palmitoyl taurine | ↓ | 0.88 | 0.025 |
| *N*-arachidonoyl taurine | ↑ | 1.18 | 0.014 |
| *N*-stearoyl tryptophan | ↓ | 0.86 | 0.066 |
| *N*-oleoyl tryptophan | ↑↑ | 1.67 | 0.08 |
| *N*-arachidonoyl tyrosine | ↑↑ | 1.88 | 0.009 |
| 2-palmitoyl glycerol | ↓ | 0.86 | 0.066 |
| 2-arachidonoyl glycerol | ↑ | 1.17 | 0.023 |
| Arachidonic acid | ↑ | 1.12 | 0.032 |
| PGE_2_ | ↑ | 1.4 | 0 |
| PGF_2α_ | ↑ | 1.47 | 0 |
| 6-ketoPGF_1α_ | ↑↑ | 1.5 | 0.009 |

Supplemental Table 88: Lipids that significantly changed in the older adult WT cerebellum relative to the younger adult WT cerebellum

| Cerebellum | | | |
| --- | --- | --- | --- |
| Lipid | Direction of change in old WT relative to young WT | Magnitude of change in old WT relative to young WT | p |
| *N*-oleoyl alanine | ↑ | 1.26 | 0.035 |
| *N*-arachidonoyl alanine | ↓ | 0.83 | 0.083 |
| *N*-palmitoyl ethanolamine | ↑ | 1.36 | 0.011 |
| *N*-stearoyl ethanolamine | ↑↑↑↑ | 4.02 | 0.031 |
| *N*-oleoyl ethanolamine | ↑↑ | 1.77 | 0.019 |
| *N*-palmitoyl GABA | ↓ | 0.67 | 0.006 |
| *N*-stearoyl GABA | ↓↓ | 0.64 | 0.01 |
| *N*-oleoyl GABA | ↓↓ | 0.6 | 0.005 |
| *N*-linoleoyl GABA | ↓↓ | 0.51 | 0.001 |
| *N*-arachidonoyl GABA | ↓↓ | 0.55 | 0 |
| *N*-docosahexaenoyl GABA | ↓↓ | 0.66 | 0 |
| *N*-palmitoyl glycine | ↓ | 0.88 | 0.026 |
| *N*-linoleoyl leucine | ↓↓ | 0.53 | 0.003 |
| *N*-docosahexaenoyl leucine | ↑ | 1.19 | 0.089 |
| *N-*oleoyl proline | ↑ | 1.36 | 0.05 |
| *N*-palmitoyl serine | ↑ | 1.09 | 0.024 |
| *N*-stearoyl serine | ↑ | 1.18 | 0.002 |
| *N*-palmitoyl taurine | ↓ | 0.88 | 0.028 |
| *N*-stearoyl taurine | ↓ | 0.82 | 0.015 |
| *N*-arachidonoyl taurine | ↓ | 0.84 | 0.019 |
| *N*-palmitoyl valine | ↑ | 1.32 | 0.084 |
| 2-palmitoyl glycerol | ↑↑ | 1.52 | 0 |
| 2-oleoyl glycerol | ↑↑ | 1.56 | 0 |
| 2-arachidonoyl glycerol | ↑ | 1.19 | 0.047 |
| phosphoLEA | ↑ | 1.26 | 0.085 |

Supplemental Table 89: Lipids that significantly changed in the older adult ABHD12 KO cerebellum relative to the younger adult ABHD12 KO cerebellum

| Cerebellum | | | |
| --- | --- | --- | --- |
| Lipid | Direction of change in old ABHD12 KO relative to young ABHD12 KO | Magnitude of change in old ABHD12 KO relative to young ABHD12 KO | p |
| *N*-palmitoyl ethanolamine | ↑↑↑ | 2.03 | 0 |
| *N*-stearoyl ethanolamine | ↑↑↑ | 2.31 | 0.043 |
| *N*-oleoyl ethanolamine | ↑↑ | 1.64 | 0.022 |
| *N*-palmitoyl GABA | ↓↓ | 0.66 | 0.006 |
| *N*-stearoyl GABA | ↓↓ | 0.66 | 0.011 |
| *N*-oleoyl GABA | ↓↓ | 0.58 | 0.006 |
| *N*-linoleoyl GABA | ↓↓↓ | 0.49 | 0.001 |
| *N*-arachidonoyl GABA | ↓↓ | 0.55 | 0 |
| *N*-docosahexaenoyl GABA | ↓↓↓ | 0.61 | 0 |
| *N*-palmitoyl glycine | ↓ | 0.88 | 0.021 |
| *N*-stearoyl leucine | ↓ | 0.86 | 0.034 |
| *N*-palmitoyl phenylalanine | ↓ | 0.67 | 0.019 |
| *N*-stearoyl phenylalanine | ↓ | 0.77 | 0 |
| *N*-oleoyl phenylalanine | ↓ | 0.72 | 0.041 |
| *N*-arachidonoyl phenylalanine | ↓↓ | 0.58 | 0.001 |
| *N*-palmitoyl serine | ↑ | 1.09 | 0.021 |
| *N*-stearoyl serine | ↑ | 1.12 | 0.012 |
| *N*-oleoyl serine | ↑ | 1.15 | 0.005 |
| *N*-stearoyl tryptophan | ↓ | 0.85 | 0.061 |
| *N*-palmitoyl tyrosine | ↓ | 0.67 | 0.043 |
| *N*-stearoyl tyrosine | ↓ | 0.68 | 0.016 |
| *N-*oleoyl tyrosine | ↓↓ | 0.66 | 0.044 |
| *N*-arachidonoyl tyrosine | ↓↓ | 1.88 | 0.009 |
| 2-palmitoyl glycerol | ↑↑ | 1.66 | 0 |
| 2-oleoyl glycerol | ↑↑ | 1.72 | 0 |
| 2-arachidonoyl glycerol | ↑ | 1.16 | 0.028 |
| Linoleic acid | ↓ | 0.9 | 0.075 |
| Arachidonic acid | ↓ | 0.91 | 0.035 |
| phosphoLEA | ↑ | 1.25 | 0.061 |

Supplemental Table 90: Lipids that significantly changed in the younger adult ABHD12 KO thalamus relative to the younger adult WT thalamus

| Thalamus | | | |
| --- | --- | --- | --- |
| Lipid | Direction of change in young ABHD12 KO relative to young WT | Magnitude of change in young ABHD12 KO relative to young WT | p |
| *N*-arachidonoyl alanine | ↑ | 1.36 | 0.054 |
| *N*-linoleoyl ethanolamine | ↑ | 1.4 | 0.001 |
| *N*-arachidonoyl ethanolamine | ↑↑ | 1.99 | 0 |
| *N*-docosahexaenoyl ethanolamine | ↑ | 1.27 | 0.027 |
| *N*-arachidonoyl GABA | ↑ | 1.24 | 0.009 |
| *N*-arachidonoyl glycine | ↑ | 1.17 | 0.037 |
| *N*-docosahexaenoyl leucine | ↓↓ | 0.65 | 0 |
| *N*-stearoyl methionine | ↑ | 1.25 | 0.07 |
| *N*-palmitoyl phenylalanine | ↑ | 1.41 | 0.018 |
| *N*-stearoyl phenylalanine | ↑↑ | 1.55 | 0.006 |
| *N*-oleoyl phenylalanine | ↑ | 1.47 | 0.004 |
| *N*-arachidonoyl phenylalanine | ↑↑↑ | 2.22 | 0 |
| *N*-docosahexaenoyl phenylalanine | ↑↑ | 1.57 | 0.023 |
| *N*-arachidonoyl serine | ↑↑ | 1.88 | 0 |
| *N*-docosahexaenoyl serine | ↑↑ | 1.57 | 0.003 |
| *N*-palmitoyl taurine | ↓ | 0.85 | 0.039 |
| *N*-arachidonoyl taurine | ↑ | 1.13 | 0.081 |
| *N*-palmitoyl tryptophan | ↑↑ | 1.75 | 0.038 |
| *N*-stearoyl tryptophan | ↑ | 1.48 | 0.05 |
| *N*-palmitoyl tyrosine | ↑↑ | 1.57 | 0.012 |
| *N*-stearoyl tyrosine | ↑↑ | 1.57 | 0.009 |
| *N-*oleoyl tyrosine | ↑ | 1.47 | 0.005 |
| *N*-arachidonoyl tyrosine | ↑↑↑ | 2.07 | 0 |
| *N*-docosahexaenoyl tyrosine | ↑↑ | 1.75 | 0.004 |
| 2-arachidonoyl glycerol | ↑ | 1.29 | 0.012 |
| Arachidonic acid | ↑ | 1.21 | 0.026 |
| PGE_2_ | ↑ | 1.37 | 0.001 |
| PGF_2α_ | ↑ | 1.42 | 0 |
| 6-ketoPGF_1α_ | ↑↑↑ | 2.36 | 0 |

Supplemental Table 91: Lipids that significantly changed in the older adult ABHD12 KO thalamus relative to the older adult WT thalamus

| Thalamus | | | |
| --- | --- | --- | --- |
| Lipid | Direction of change in old ABHD12 KO relative to old WT | Magnitude of change in old ABHD12 KO relative to old WT | p |
| *N*-stearoyl alanine | ↑ | 1.22 | 0.021 |
| *N*-arachidonoyl alanine | ↑ | 1.34 | 0.041 |
| *N*-stearoyl ethanolamine | ↑ | 1.49 | 0.088 |
| *N*-linoleoyl ethanolamine | ↑ | 1.24 | 0.066 |
| *N*-arachidonoyl ethanolamine | ↑↑ | 1.58 | 0.006 |
| *N*-arachidonoyl GABA | ↑ | 1.27 | 0.092 |
| *N*-palmitoyl glycine | ↑ | 1.17 | 0.071 |
| *N*-arachidonoyl glycine | ↑ | 1.14 | 0.083 |
| *N*-docosahexaenoyl leucine | ↓ | 0.76 | 0.027 |
| *N*-arachidonoyl phenylalanine | ↑ | 1.45 | 0.017 |
| *N*-arachidonoyl serine | ↑↑ | 1.86 | 0 |
| *N*-docosahexaenoyl serine | ↑ | 1.34 | 0.031 |
| *N*-stearoyl taurine | ↑ | 1.18 | 0.058 |
| *N-*oleoyl taurine | ↑ | 1.11 | 0.055 |
| *N*-arachidonoyl taurine | ↑ | 1.27 | 0.001 |
| *N*-arachidonoyl tyrosine | ↑↑ | 1.93 | 0 |
| *N*-docosahexaenoyl tyrosine | ↑↑ | 1.6 | 0.004 |
| 2-oleoyl glycerol | ↓ | 0.89 | 0.083 |
| 2-arachidonoyl glycerol | ↑ | 1.29 | 0.008 |
| PGE_2_ | ↑ | 1.47 | 0 |
| PGF_2α_ | ↑ | 1.45 | 0 |
| 6-ketoPGF_1α_ | ↑↑↑ | 2.05 | 0 |
| Sample Mass | ↓ | 0.89 | 0.065 |

Supplemental Table 92: Lipids that significantly changed in the older adult WT thalamus relative to the younger adult WT thalamus

| Thalamus | | | |
| --- | --- | --- | --- |
| Lipid | Direction of change in old WT relative to young WT | Magnitude of change in old WT relative to young WT | p |
| *N*-palmitoyl ethanolamine | ↑ | 1.22 | 0.086 |
| *N*-stearoyl ethanolamine | ↑↑↑ | 2.45 | 0.058 |
| *N*-palmitoyl GABA | ↓↓ | 0.59 | 0 |
| *N*-stearoyl GABA | ↓ | 0.67 | 0.019 |
| *N*-oleoyl GABA | ↓↓ | 0.63 | 0 |
| *N*-linoleoyl GABA | ↓↓ | 0.53 | 0 |
| *N*-arachidonoyl GABA | ↓↓ | 0.52 | 0 |
| *N*-docosahexaenoyl GABA | ↓↓ | 0.52 | 0 |
| *N*-stearoyl glycine | ↓ | 0.83 | 0.041 |
| *N*-oleoyl glycine | ↓ | 0.86 | 0.039 |
| *N*-linoleoyl glycine | ↓ | 0.76 | 0.02 |
| *N*-arachidonoyl glycine | ↓ | 0.85 | 0.059 |
| *N*-docosahexaenoyl leucine | ↓ | 0.8 | 0.003 |
| *N-*oleoyl taurine | ↓ | 0.81 | 0.01 |
| 2-oleoyl glycerol | ↑ | 1.16 | 0.036 |
| Oleic acid | ↓ | 0.72 | 0.009 |
| Linoleic acid | ↓ | 0.69 | 0.002 |
| Arachidonic acid | ↓ | 0.74 | 0.006 |

Supplemental Table 93: Lipids that significantly changed in the older adult ABHD12 KO thalamus relative to the younger adult ABHD12 KO thalamus

| Thalamus | | | |
| --- | --- | --- | --- |
| Lipid | Direction of change in old ABHD12 KO relative to young ABHD12 KO | Magnitude of change in old ABHD12 KO relative to young ABHD12 KO | p |
| *N*-palmitoyl ethanolamine | ↑ | 1.29 | 0.018 |
| *N*-stearoyl ethanolamine | ↑↑↑ | 2.55 | 0.003 |
| *N*-linoleoyl ethanolamine | ↓ | 0.72 | 0.001 |
| *N*-arachidonoyl ethanolamine | ↓ | 0.75 | 0.01 |
| *N*-docosahexaenoyl ethanolamine | ↓ | 0.8 | 0.022 |
| *N*-palmitoyl GABA | ↓↓ | 0.63 | 0.001 |
| *N*-stearoyl GABA | ↓ | 0.77 | 0.074 |
| *N*-oleoyl GABA | ↓↓ | 0.64 | 0 |
| *N*-linoleoyl GABA | ↓↓ | 0.54 | 0 |
| *N*-arachidonoyl GABA | ↓↓ | 0.52 | 0 |
| *N*-docosahexaenoyl GABA | ↓↓ | 0.64 | 0.002 |
| *N*-stearoyl glycine | ↓ | 0.85 | 0.042 |
| *N*-oleoyl glycine | ↓ | 0.89 | 0.064 |
| *N*-linoleoyl glycine | ↓ | 0.76 | 0.006 |
| *N*-arachidonoyl glycine | ↓ | 0.84 | 0.01 |
| *N*-docosahexaenoyl glycine | ↓ | 0.83 | 0.028 |
| *N*-stearoyl methionine | ↓ | 0.77 | 0.027 |
| *N*-palmitoyl phenylalanine | ↓ | 0.8 | 0.069 |
| *N*-stearoyl phenylalanine | ↓ | 0.73 | 0.017 |
| *N*-oleoyl phenylalanine | ↓ | 0.78 | 0.025 |
| *N*-oleoyl serine | ↑ | 1.15 | 0.023 |
| *N*-palmitoyl taurine | ↑ | 1.17 | 0.03 |
| *N*-palmitoyl tryptophan | ↓↓ | 0.64 | 0.056 |
| *N*-palmitoyl tyrosine | ↓ | 0.78 | 0.083 |
| *N*-stearoyl tyrosine | ↓ | 0.7 | 0.017 |
| *N-*oleoyl tyrosine | ↓ | 0.78 | 0.026 |
| 2-linoleoyl glycerol | ↓ | 0.8 | 0.03 |
| Oleic acid | ↓↓ | 0.66 | 0 |
| Linoleic acid | ↓ | 0.68 | 0 |
| Arachidonic acid | ↓ | 0.71 | 0 |
| 6-ketoPGF_1α_ | ↓ | 0.85 | 0.062 |

Supplemental Table 94: Lipids that significantly changed in the younger adult ABHD12 KO cortex relative to the younger adult WT cortex

| Cortex | | | |
| --- | --- | --- | --- |
| Lipid | Direction of change in young ABHD12 KO relative to young WT | Magnitude of change in young ABHD12 KO relative to young WT | p |
| *N*-arachidonoyl ethanolamine | ↑↑ | 1.62 | 0.003 |
| *N*-palmitoyl GABA | ↓ | 0.75 | 0.056 |
| *N*-arachidonoyl GABA | ↑ | 1.24 | 0.04 |
| *N*-stearoyl leucine | ↑ | 1.08 | 0.095 |
| *N*-docosahexaenoyl leucine | ↓ | 0.74 | 0.005 |
| *N*-oleoyl phenylalanine | ↑ | 1.41 | 0.088 |
| *N*-linoleoyl phenylalanine | ↑↑ | 1.98 | 0 |
| *N*-arachidonoyl phenylalanine | ↑↑ | 1.94 | 0 |
| *N*-docosahexaenoyl phenylalanine | ↑↑ | 1.9 | 0.001 |
| *N*-stearoyl proline | ↑ | 1.26 | 0.003 |
| *N*-arachidonoyl serine | ↑ | 1.45 | 0 |
| *N*-docosahexaenoyl serine | ↑ | 1.32 | 0.005 |
| *N*-stearoyl tryptophan | ↑ | 1.3 | 0.05 |
| *N*-stearoyl tyrosine | ↑ | 1.27 | 0.085 |
| *N-*oleoyl tyrosine | ↑↑ | 1.7 | 0.046 |
| *N*-arachidonoyl tyrosine | ↑↑ | 1.81 | 0 |
| *N*-docosahexaenoyl tyrosine | ↑ | 1.28 | 0.012 |
| 2-palmitoyl glycerol | ↓ | 0.72 | 0.033 |
| 2-oleoyl glycerol | ↓ | 0.76 | 0.033 |
| Oleic acid | ↑ | 1.1 | 0.086 |
| Arachidonic acid | ↑ | 1.1 | 0.041 |
| PGE_2_ | ↑ | 1.29 | 0.002 |
| PGF_2α_ | ↑ | 1.34 | 0 |
| 6-ketoPGF_1α_ | ↑↑ | 1.56 | 0.001 |

Supplemental Table 95: Lipids that significantly changed in the older adult ABHD12 KO cortex relative to the older adult WT cortex

| Cortex | | | |
| --- | --- | --- | --- |
| Lipid | Direction of change in old ABHD12 KO relative to old WT | Magnitude of change in old ABHD12 KO relative to old WT | p |
| *N*-stearoyl alanine | ↑ | 1.13 | 0.004 |
| *N*-oleoyl alanine | ↓ | 0.81 | 0.064 |
| *N*-arachidonoyl alanine | ↑ | 1.37 | 0.004 |
| *N*-arachidonoyl ethanolamine | ↑↑ | 1.59 | 0.002 |
| *N*-arachidonoyl glycine | ↑ | 1.14 | 0.062 |
| *N*-stearoyl leucine | ↑ | 1.09 | 0.074 |
| *N*-docosahexaenoyl leucine | ↓ | 0.82 | 0.03 |
| *N*-arachidonoyl phenylalanine | ↑↑ | 1.54 | 0.017 |
| *N*-palmitoyl proline | ↑ | 1.25 | 0.065 |
| *N*-stearoyl proline | ↑ | 1.21 | 0.081 |
| *N*-arachidonoyl serine | ↑↑ | 1.74 | 0 |
| *N*-stearoyl taurine | ↑ | 1.12 | 0.052 |
| *N-*oleoyl taurine | ↑ | 1.16 | 0.031 |
| *N*-arachidonoyl taurine | ↑ | 1.35 | 0.005 |
| *N*-arachidonoyl tyrosine | ↑↑ | 1.52 | 0.044 |
| *N*-docosahexaenoyl tyrosine | ↑↑ | 1.82 | 0 |
| *N*-palmitoyl valine | ↓ | 0.81 | 0.023 |
| *N*- stearoyl valine | ↑ | 1.33 | 0.018 |
| 2-palmitoyl glycerol | ↓ | 0.85 | 0.089 |
| Arachidonic acid | ↑ | 1.13 | 0.01 |
| PGE_2_ | ↑ | 1.39 | 0 |
| PGF_2α_ | ↑ | 1.36 | 0 |
| 6-ketoPGF_1α_ | ↑↑ | 1.82 | 0 |
| Sample Mass | ↓ | 0.92 | 0.042 |

Supplemental Table 96: Lipids that significantly changed in the older adult WT cortex relative to the younger adult WT cortex

| Cortex | | | |
| --- | --- | --- | --- |
| Lipid | Direction of change in old WT relative to young WT | Magnitude of change in old WT relative to young WT | p |
| *N*-oleoyl alanine | ↑ | 1.28 | 0.055 |
| *N*-palmitoyl ethanolamine | ↑ | 1.33 | 0.055 |
| *N*-oleoyl ethanolamine | ↑ | 1.32 | 0.076 |
| *N*-palmitoyl GABA | ↓↓↓ | 0.46 | 0 |
| *N*-stearoyl GABA | ↓↓ | 0.57 | 0.001 |
| *N*-oleoyl GABA | ↓↓ | 0.51 | 0 |
| *N*-linoleoyl GABA | ↓↓↓ | 0.35 | 0 |
| *N*-arachidonoyl GABA | ↓↓ | 0.54 | 0 |
| *N*-docosahexaenoyl GABA | ↓↓ | 0.54 | 0.001 |
| *N*-palmitoyl glycine | ↓ | 0.78 | 0.007 |
| *N*-stearoyl glycine | ↓ | 0.7 | 0.003 |
| *N*-oleoyl glycine | ↓ | 0.73 | 0.002 |
| *N*-linoleoyl glycine | ↓ | 0.79 | 0.018 |
| *N*-arachidonoyl glycine | ↓ | 0.78 | 0.002 |
| *N*-docosahexaenoyl glycine | ↓ | 0.8 | 0.013 |
| *N*-palmitoyl phenylalanine | ↓↓↓ | 0.5 | 0.039 |
| *N*-stearoyl phenylalanine | ↓↓ | 0.61 | 0.011 |
| *N*-oleoyl phenylalanine | ↓↓ | 0.52 | 0.048 |
| *N*-arachidonoyl phenylalanine | ↓↓ | 0.66 | 0.036 |
| *N*-linoleoyl serine | ↓ | 0.69 | 0 |
| *N*-arachidonoyl serine | ↓ | 0.75 | 0.016 |
| *N*-palmitoyl taurine | ↓ | 0.88 | 0.045 |
| *N-*oleoyl taurine | ↓ | 0.84 | 0.022 |
| *N*-arachidonoyl taurine | ↓ | 0.76 | 0.018 |
| *N*-palmitoyl tyrosine | ↓↓ | 0.61 | 0.099 |
| *N*-arachidonoyl tyrosine | ↓↓ | 0.64 | 0.046 |
| *N*-docosahexaenoyl tyrosine | ↓↓↓ | 0.48 | 0 |
| *N*- stearoyl valine | ↓ | 0.75 | 0.029 |
| 2-palmitoyl glycerol | ↑ | 1.36 | 0.008 |
| 2-oleoyl glycerol | ↑ | 1.3 | 0.027 |
| Linoleic acid | ↓ | 0.84 | 0.023 |
| 6-ketoPGF_1α_ | ↓ | 0.71 | 0.054 |

Supplemental Table 97: Lipids that significantly changed in the older adult ABHD12 KO cortex relative to the younger adult ABHD12 KO cortex

| Cortex | | | |
| --- | --- | --- | --- |
| Lipid | Direction of change in old ABHD12 KO relative to young ABHD12 KO | Magnitude of change in old ABHD12 KO relative to young ABHD12 KO | p |
| *N*-palmitoyl alanine | ↑ | 1.15 | 0.055 |
| *N*-stearoyl alanine | ↑ | 1.08 | 0.042 |
| *N*-palmitoyl ethanolamine | ↑↑ | 1.56 | 0.003 |
| *N*-stearoyl ethanolamine | ↑↑↑ | 2 | 0.044 |
| *N*-oleoyl ethanolamine | ↑↑ | 1.5 | 0.007 |
| *N*-palmitoyl GABA | ↓↓ | 0.55 | 0.009 |
| *N*-stearoyl GABA | ↓↓ | 0.6 | 0.002 |
| *N*-oleoyl GABA | ↓↓ | 0.57 | 0.002 |
| *N*-linoleoyl GABA | ↓↓ | 0.52 | 0.006 |
| *N*-arachidonoyl GABA | ↓↓ | 0.53 | 0 |
| *N*-docosahexaenoyl GABA | ↓↓ | 0.54 | 0.001 |
| *N*-palmitoyl glycine | ↓ | 0.81 | 0.013 |
| *N*-stearoyl glycine | ↓ | 0.75 | 0.007 |
| *N*-oleoyl glycine | ↓ | 0.78 | 0.003 |
| *N*-linoleoyl glycine | ↓ | 0.75 | 0.022 |
| *N*-arachidonoyl glycine | ↓ | 0.86 | 0.02 |
| *N*-docosahexaenoyl glycine | ↓ | 0.86 | 0.049 |
| *N*-palmitoyl phenylalanine | ↓↓↓ | 0.45 | 0.004 |
| *N*-stearoyl phenylalanine | ↓↓ | 0.56 | 0 |
| *N*-oleoyl phenylalanine | ↓↓↓ | 0.43 | 0.001 |
| *N*-linoleoyl phenylalanine | ↓↓ | 0.53 | 0 |
| *N*-arachidonoyl phenylalanine | ↓↓ | 0.52 | 0 |
| *N*-docosahexaenoyl phenylalanine | ↓↓ | 0.59 | 0.001 |
| *N*-palmitoyl proline | ↑ | 1.26 | 0.059 |
| *N*-linoleoyl serine | ↓ | 0.72 | 0.001 |
| *N*-arachidonoyl serine | ↓ | 0.89 | 0.09 |
| *N*-docosahexaenoyl serine | ↓ | 0.83 | 0.021 |
| *N*-palmitoyl tyrosine | ↓↓ | 0.51 | 0.005 |
| *N*-stearoyl tyrosine | ↓↓ | 0.59 | 0.001 |
| *N-*oleoyl tyrosine | ↓↓↓ | 0.46 | 0.006 |
| *N*-arachidonoyl tyrosine | ↓↓ | 0.54 | 0 |
| *N*-docosahexaenoyl tyrosine | ↓ | 0.68 | 0 |
| *N*-palmitoyl valine | ↓ | 0.79 | 0.014 |
| 2-palmitoyl glycerol | ↑↑ | 1.62 | 0.001 |
| 2-oleoyl glycerol | ↑↑ | 1.56 | 0.001 |
| Oleic acid | ↓ | 0.91 | 0.053 |
| 6-ketoPGF_1α_ | ↓ | 0.83 | 0.051 |
| Sample Mass | ↓ | 0.92 | 0.035 |

Supplemental Table 98: Lipids that significantly changed in the younger adult ABHD12 KO hypothalamus relative to the younger adult WT hypothalamus

| Hypothalamus | | | |
| --- | --- | --- | --- |
| Lipid | Direction of change in young ABHD12 KO relative to young WT | Magnitude of change in young ABHD12 KO relative to young WT | p |
| *N*-oleoyl alanine | ↓ | 0.79 | 0.014 |
| *N*-palmitoyl ethanolamine | ↓ | 0.75 | 0.055 |
| *N*-palmitoyl GABA | ↓ | 0.76 | 0.019 |
| *N*-stearoyl GABA | ↓ | 0.72 | 0.002 |
| *N*-oleoyl GABA | ↓ | 0.72 | 0.025 |
| *N*-palmitoyl glycine | ↓ | 0.83 | 0.057 |
| *N*-stearoyl glycine | ↓ | 0.79 | 0.057 |
| *N*-oleoyl glycine | ↓ | 0.77 | 0.027 |
| *N*-docosahexaenoyl leucine | ↑↑ | 1.59 | 0.003 |
| *N*-stearoyl phenylalanine | ↑ | 1.2 | 0.072 |
| *N*-arachidonoyl phenylalanine | ↑↑ | 1.5 | 0.063 |
| *N*-palmitoyl serine | ↓ | 0.77 | 0.022 |
| *N*-palmitoyl taurine | ↓↓ | 0.66 | 0 |
| *N*-stearoyl taurine | ↓↓ | 0.6 | 0.003 |
| *N-*oleoyl taurine | ↓ | 0.73 | 0.007 |

Supplemental Table 99: Lipids that significantly changed in the older adult ABHD12 KO hypothalamus relative to the older adult WT hypothalamus

| Hypothalamus | | | |
| --- | --- | --- | --- |
| Lipid | Direction of change in old ABHD12 KO relative to old WT | Magnitude of change in old ABHD12 KO relative to old WT | p |
| *N*-oleoyl alanine | ↓ | 0.71 | 0.001 |
| *N*-oleoyl phenylalanine | ↓ | 0.68 | 0.012 |
| *N*-palmitoyl serine | ↓ | 0.78 | 0.035 |
| *N*-oleoyl serine | ↓ | 0.8 | 0.034 |
| *N*-palmitoyl taurine | ↓ | 0.75 | 0.005 |
| *N*-arachidonoyl taurine | ↑ | 1.26 | 0.034 |
| 2-oleoyl glycerol | ↓ | 0.73 | 0.09 |
| 2-arachidonoyl glycerol | ↓ | 0.74 | 0.075 |
| Linoleic acid | ↓ | 0.86 | 0.1 |
| PGE_2_ | ↑ | 1.42 | 0.002 |
| PGF_2α_ | ↑ | 1.39 | 0.008 |

Supplemental Table 100: Lipids that significantly changed in the older adult WT hypothalamus relative to the younger adult WT hypothalamus

| Hypothalamus | | | |
| --- | --- | --- | --- |
| Lipid | Direction of change in old WT relative to young WT | Magnitude of change in old WT relative to young WT | p |
| *N*-palmitoyl alanine | ↓ | 0.83 | 0.037 |
| *N*-linoleoyl ethanolamine | ↓↓ | 0.62 | 0.026 |
| *N*-docosahexaenoyl ethanolamine | ↓ | 0.75 | 0.076 |
| *N*-palmitoyl GABA | ↓ | 0.82 | 0.073 |
| *N*-stearoyl GABA | ↓ | 0.74 | 0.007 |
| *N*-oleoyl glycine | ↓ | 0.77 | 0.027 |
| *N*-arachidonoyl glycine | ↓ | 0.78 | 0.04 |
| *N*-docosahexaenoyl glycine | ↓ | 0.7 | 0.024 |
| *N*-palmitoyl leucine | ↓ | 0.81 | 0.048 |
| *N-*oleoyl taurine | ↓ | 0.76 | 0.023 |
| 2-palmitoyl glycerol | ↓↓ | 0.61 | 0.002 |
| 2-linoleoyl glycerol | ↓ | 0.72 | 0.086 |
| Oleic acid | ↓ | 0.84 | 0.099 |
| Linoleic acid | ↓ | 0.85 | 0.045 |

Supplemental Table 101: Lipids that significantly changed in the older adult ABHD12 KO hypothalamus relative to the younger adult ABHD12 KO hypothalamus

| Hypothalamus | | | |
| --- | --- | --- | --- |
| Lipid | Direction of change in old ABHD12 KO relative to young ABHD12 KO | Magnitude of change in old ABHD12 KO relative to young ABHD12 KO | p |
| *N*-palmitoyl alanine | ↓ | 0.82 | 0.026 |
| *N*-linoleoyl ethanolamine | ↓↓ | 0.65 | 0.04 |
| *N*-arachidonoyl ethanolamine | ↓ | 0.71 | 0.054 |
| *N*-docosahexaenoyl ethanolamine | ↓ | 0.8 | 0.072 |
| *N*-arachidonoyl glycine | ↓ | 0.82 | 0.077 |
| *N*-stearoyl leucine | ↓ | 0.82 | 0.083 |
| *N*-docosahexaenoyl leucine | ↓ | 0.7 | 0.01 |
| *N*-palmitoyl phenylalanine | ↓ | 0.69 | 0.024 |
| *N*-stearoyl phenylalanine | ↓ | 0.69 | 0.002 |
| *N*-arachidonoyl phenylalanine | ↓↓ | 0.58 | 0.016 |
| *N*-stearoyl proline | ↓ | 0.73 | 0.033 |
| *N*-stearoyl taurine | ↑ | 1.44 | 0.029 |
| *N*-arachidonoyl taurine | ↑ | 1.18 | 0.094 |
| 2-palmitoyl glycerol | ↓↓ | 0.56 | 0.001 |
| 2-oleoyl glycerol | ↓↓ | 0.63 | 0.007 |
| 2-linoleoyl glycerol | ↓↓ | 0.55 | 0.003 |
| 2-arachidonoyl glycerol | ↓↓ | 0.62 | 0.003 |
| Oleic acid | ↓ | 9.83 | 0.094 |
| Linoleic acid | ↓ | 0.73 | 0 |
| Arachidonic acid | ↓ | 0.85 | 0.073 |

Supplemental Table 102: Lipids that significantly changed in the younger adult ABHD12 KO midbrain relative to the younger adult WT midbrain

| Midbrain | | | |
| --- | --- | --- | --- |
| Lipid | Direction of change in young ABHD12 KO relative to young WT | Magnitude of change in young ABHD12 KO relative to young WT | p |
| *N*-stearoyl alanine | ↑ | 1.12 | 0.08 |
| *N*-arachidonoyl alanine | ↑ | 1.34 | 0.03 |
| *N*-linoleoyl ethanolamine | ↑ | 1.26 | 0.028 |
| *N*-arachidonoyl ethanolamine | ↑↑ | 1.5 | 0.001 |
| *N*-palmitoyl GABA | ↓ | 0.86 | 0.054 |
| *N*-arachidonoyl GABA | ↑ | 1.26 | 0.003 |
| *N*-arachidonoyl glycine | ↑ | 1.14 | 0.02 |
| *N*-palmitoyl phenylalanine | ↑↑ | 1.59 | 0.002 |
| *N*-stearoyl phenylalanine | ↑ | 1.36 | 0.041 |
| *N*-oleoyl phenylalanine | ↑↑ | 1.55 | 0.021 |
| *N*-arachidonoyl phenylalanine | ↑↑↑ | 2.38 | 0 |
| *N*-docosahexaenoyl phenylalanine | ↑↑ | 1.81 | 0.023 |
| *N*-arachidonoyl serine | ↑↑ | 1.67 | 0 |
| *N*-palmitoyl taurine | ↓ | 0.87 | 0.062 |
| *N*-arachidonoyl taurine | ↑ | 1.22 | 0.037 |
| *N*-palmitoyl tryptophan | ↑↑ | 1.57 | 0.069 |
| *N*-palmitoyl tyrosine | ↑↑ | 1.63 | 0.007 |
| *N*-stearoyl tyrosine | ↑ | 1.45 | 0.019 |
| *N-*oleoyl tyrosine | ↑↑ | 1.5 | 0.031 |
| *N*-arachidonoyl tyrosine | ↑↑↑ | 2.38 | 0 |
| *N*-docosahexaenoyl tyrosine | ↑↑ | 1.56 | 0.018 |
| *N*-palmitoyl valine | ↑ | 1.28 | 0.007 |
| 2-arachidonoyl glycerol | ↑ | 1.33 | 0.001 |
| Arachidonic acid | ↑ | 1.17 | 0.009 |
| phosphoLEA | ↑↑ | 1.51 | 0.001 |
| PGE_2_ | ↑↑ | 1.51 | 0.001 |
| PGF_2α_ | ↑↑ | 1.6 | 0 |
| 6-ketoPGF_1α_ | ↑↑ | 1.71 | 0.003 |

Supplemental Table 103: Lipids that significantly changed in the older adult ABHD12 KO midbrain relative to the older adult WT midbrain

| Midbrain | | | |
| --- | --- | --- | --- |
| Lipid | Direction of change in old ABHD12 KO relative to old WT | Magnitude of change in old ABHD12 KO relative to old WT | p |
| *N*-stearoyl alanine | ↑ | 1.1 | 0.099 |
| *N*-oleoyl alanine | ↓ | 0.86 | 0.033 |
| *N*-arachidonoyl alanine | ↑ | 1.32 | 0.028 |
| *N*-arachidonoyl ethanolamine | ↑ | 1.37 | 0.004 |
| *N*-arachidonoyl GABA | ↑ | 1.23 | 0.077 |
| *N*-arachidonoyl glycine | ↑ | 1.13 | 0.02 |
| *N*-docosahexaenoyl leucine | ↓↓ | 0.66 | 0 |
| *N*-arachidonoyl phenylalanine | ↑ | 1.48 | 0.017 |
| *N*-palmitoyl serine | ↓ | 0.87 | 0.026 |
| *N*-oleoyl serine | ↓ | 0.91 | 0.019 |
| *N*-arachidonoyl serine | ↑↑ | 1.79 | 0 |
| *N*-arachidonoyl taurine | ↑ | 1.2 | 0.049 |
| *N*-arachidonoyl tyrosine | ↑↑ | 1.74 | 0.001 |
| 2-palmitoyl glycerol | ↓ | 0.76 | 0.005 |
| 2-oleoyl glycerol | ↓ | 0.84 | 0.006 |
| 2-arachidonoyl glycerol | ↑ | 1.26 | 0.001 |
| Linoleic acid | ↑ | 1.16 | 0.038 |
| Arachidonic acid | ↑ | 1.16 | 0.018 |
| phosphoLEA | ↑ | 1.29 | 0.008 |
| PGE_2_ | ↑ | 1.45 | 0.007 |
| PGF_2α_ | ↑↑ | 1.5 | 0 |
| 6-ketoPGF_1α_ | ↑↑ | 1.81 | 0.003 |
| Sample Mass | ↓ | 0.89 | 0.009 |

Supplemental Table 104: Lipids that significantly changed in the older adult WT midbrain relative to the younger adult WT midbrain

| Midbrain | | | |
| --- | --- | --- | --- |
| Lipid | Direction of change in old WT relative to young WT | Magnitude of change in old WT relative to young WT | p |
| *N*-oleoyl alanine | ↑ | 1.14 | 0.092 |
| *N*-palmitoyl ethanolamine | ↑↑ | 1.66 | 0 |
| *N*-stearoyl ethanolamine | ↑↑↑↑ | 4.73 | 0 |
| *N*-oleoyl ethanolamine | ↑↑ | 1.58 | 0.004 |
| *N*-palmitoyl GABA | ↓↓ | 0.62 | 0 |
| *N*-stearoyl GABA | ↓↓ | 0.65 | 0 |
| *N*-oleoyl GABA | ↓↓ | 0.55 | 0 |
| *N*-linoleoyl GABA | ↓↓↓ | 0.46 | 0.001 |
| *N*-arachidonoyl GABA | ↓↓ | 0.57 | 0 |
| *N*-docosahexaenoyl GABA | ↓↓ | 0.63 | 0.008 |
| *N*-stearoyl glycine | ↓ | 0.77 | 0.001 |
| *N*-oleoyl glycine | ↓ | 0.87 | 0.018 |
| *N*-linoleoyl glycine | ↓ | 0.78 | 0.008 |
| *N*-arachidonoyl glycine | ↓ | 0.91 | 0.1 |
| *N*-docosahexaenoyl glycine | ↓ | 0.85 | 0.006 |
| *N*-stearoyl leucine | ↓ | 0.86 | 0.05 |
| *N*-palmitoyl serine | ↑ | 1.28 | 0.001 |
| *N*-stearoyl serine | ↑ | 1.14 | 0.022 |
| *N*-oleoyl serine | ↑ | 1.19 | 0 |
| 2-palmitoyl glycerol | ↑ | 1.23 | 0.032 |
| 2-oleoyl glycerol | ↑ | 1.3 | 0.001 |
| Oleic acid | ↓ | 0.84 | 0.025 |
| Linoleic acid | ↓ | 0.73 | 0 |
| Arachidonic acid | ↓ | 0.88 | 0 |

Supplemental Table 105: Lipids that significantly changed in the older adult ABHD12 KO midbrain relative to the younger adult ABHD12 KO midbrain

| Midbrain | | | |
| --- | --- | --- | --- |
| Lipid | Direction of change in old ABHD12 KO relative to young ABHD12 KO | Magnitude of change in old ABHD12 KO relative to young ABHD12 KO | p |
| *N*-palmitoyl ethanolamine | ↑↑ | 1.54 | 0.002 |
| *N*-stearoyl ethanolamine | ↑↑↑↑ | 3.45 | 0 |
| *N*-oleoyl ethanolamine | ↑↑ | 1.56 | 0.001 |
| *N*-linoleoyl ethanolamine | ↓ | 0.78 | 0.012 |
| *N*-palmitoyl GABA | ↓↓ | 0.66 | 0 |
| *N*-stearoyl GABA | ↓↓ | 0.66 | 0 |
| *N*-oleoyl GABA | ↓↓ | 0.6 | 0 |
| *N*-linoleoyl GABA | ↓↓↓ | 0.45 | 0 |
| *N*-arachidonoyl GABA | ↓↓ | 0.56 | 0 |
| *N*-docosahexaenoyl GABA | ↓↓ | 0.56 | 0 |
| *N*-stearoyl glycine | ↓ | 0.77 | 0.001 |
| *N*-oleoyl glycine | ↓ | 0.9 | 0.056 |
| *N*-arachidonoyl glycine | ↓ | 0.91 | 0.046 |
| *N*-docosahexaenoyl glycine | ↓ | 0.92 | 0.088 |
| *N*-linoleoyl leucine | ↓↓ | 0.6 | 0.001 |
| *N*-docosahexaenoyl leucine | ↓↓ | 0.66 | 0.015 |
| *N*-palmitoyl phenylalanine | ↓↓ | 0.66 | 0.002 |
| *N*-stearoyl phenylalanine | ↓ | 0.73 | 0.024 |
| *N*-oleoyl phenylalanine | ↓ | 0.67 | 0.019 |
| *N*-arachidonoyl phenylalanine | ↓ | 0.77 | 0.031 |
| *N-*oleoyl proline | ↑ | 1.47 | 0.044 |
| *N*-palmitoyl serine | ↑ | 1.17 | 0.026 |
| *N*-stearoyl serine | ↑ | 1.22 | 0 |
| *N*-oleoyl serine | ↑ | 1.09 | 0.034 |
| *N*-arachidonoyl serine | ↑ | 1.23 | 0.007 |
| *N*-docosahexaenoyl serine | ↑ | 1.21 | 0.023 |
| *N*-palmitoyl tyrosine | ↓ | 0.75 | 0.047 |
| *N-*oleoyl tyrosine | ↓ | 0.7 | 0.036 |
| *N*-arachidonoyl tyrosine | ↓ | 0.69 | 0.001 |
| *N*-palmitoyl valine | ↓ | 0.84 | 0.031 |
| 2-oleoyl glycerol | ↑ | 1.2 | 0.016 |
| 2-linoleoyl glycerol | ↓ | 0.86 | 0.009 |
| Oleic acid | ↓ | 0.79 | 0.001 |
| Linoleic acid | ↓ | 0.78 | 0 |
| Arachidonic acid | ↓ | 0.87 | 0.009 |
| PGE_2_ | ↓ | 0.8 | 0.031 |
| PGF_2α_ | ↓ | 0.81 | 0 |

Supplemental Table 106: Lipids that significantly changed in the younger adult ABHD12 KO brainstem relative to the younger adult WT brainstem

| Brainstem | | | |
| --- | --- | --- | --- |
| Lipid | Direction of change in young ABHD12 KO relative to young WT | Magnitude of change in young ABHD12 KO relative to young WT | p |
| *N*-docosahexaenoyl alanine | ↑ | 1.4 | 0.038 |
| *N*-oleoyl ethanolamine | ↑ | 1.33 | 0.085 |
| *N*-linoleoyl ethanolamine | ↑ | 1.44 | 0.004 |
| *N*-arachidonoyl ethanolamine | ↑↑ | 1.77 | 0 |
| *N*-arachidonoyl GABA | ↑ | 1.34 | 0 |
| *N*-arachidonoyl glycine | ↑ | 1.13 | 0.005 |
| *N*-palmitoyl leucine | ↓ | 0.91 | 0.084 |
| *N*-stearoyl phenylalanine | ↑ | 1.34 | 0.048 |
| *N*-arachidonoyl phenylalanine | ↑ | 1.48 | 0.071 |
| *N*-docosahexaenoyl phenylalanine | ↑↑ | 1.5 | 0.045 |
| *N*-palmitoyl serine | ↓ | 0.89 | 0 |
| *N*-oleoyl serine | ↓ | 0.9 | 0.017 |
| *N*-linoleoyl serine | ↑ | 1.38 | 0.001 |
| *N*-arachidonoyl serine | ↑↑ | 1.64 | 0 |
| *N*-palmitoyl taurine | ↓ | 0.8 | 0 |
| *N*-stearoyl taurine | ↓ | 0.83 | 0.002 |
| *N*-arachidonoyl taurine | ↑ | 1.14 | 0.067 |
| *N*-oleoyl tryptophan | ↑↑↑ | 2.56 | 0 |
| *N*-palmitoyl tyrosine | ↑ | 1.31 | 0.04 |
| *N*-stearoyl tyrosine | ↑↑ | 1.66 | 0.008 |
| *N-*oleoyl tyrosine | ↑ | 1.39 | 0.014 |
| *N*-arachidonoyl tyrosine | ↑↑ | 1.77 | 0 |
| *N*-docosahexaenoyl tyrosine | ↑↑↑ | 2.23 | 0 |
| *N*- stearoyl valine | ↑ | 1.34 | 0.006 |
| *N*-oleoyl valine | ↑ | 1.32 | 0.026 |
| 2-linoleoyl glycerol | ↑ | 1.22 | 0.011 |
| 2-arachidonoyl glycerol | ↑ | 1.42 | 0 |
| Arachidonic acid | ↑ | 1.28 | 0.007 |
| phosphoLEA | ↑↑ | 1.58 | 0.001 |
| PGE_2_ | ↑ | 1.4 | 0 |
| PGF_2α_ | ↑↑ | 1.51 | 0 |
| 6-ketoPGF_1α_ | ↑↑↑ | 2.12 | 0 |

Supplemental Table 107: Lipids that significantly changed in the older adult ABHD12 KO brainstem relative to the older adult WT brainstem

| Brainstem | | | |
| --- | --- | --- | --- |
| Lipid | Direction of change in old ABHD12 KO relative to old WT | Magnitude of change in old ABHD12 KO relative to old WT | p |
| *N*-oleoyl alanine | ↓ | 0.85 | 0.041 |
| *N*-linoleoyl alanine | ↓ | 0.67 | 0.016 |
| *N*-arachidonoyl alanine | ↑↑ | 1.54 | 0.066 |
| *N*-palmitoyl ethanolamine | ↓ | 0.73 | 0.001 |
| *N*-oleoyl ethanolamine | ↓ | 0.8 | 0.053 |
| *N*-arachidonoyl ethanolamine | ↑ | 1.34 | 0.014 |
| *N*-palmitoyl GABA | ↓ | 0.83 | 0.069 |
| *N*-stearoyl GABA | ↓ | 0.86 | 0.037 |
| *N*-oleoyl GABA | ↓ | 0.78 | 0.04 |
| *N*-arachidonoyl GABA | ↑ | 1.29 | 0 |
| *N*-arachidonoyl glycine | ↑ | 1.11 | 0.01 |
| *N*-arachidonoyl phenylalanine | ↑↑ | 1.5 | 0.026 |
| *N*-palmitoyl serine | ↓ | 0.89 | 0 |
| *N*-oleoyl serine | ↓ | 0.85 | 0 |
| *N*-arachidonoyl serine | ↑ | 1.36 | 0.001 |
| *N*-docosahexaenoyl serine | ↑ | 1.18 | 0.046 |
| *N*-palmitoyl taurine | ↓ | 0.89 | 0.003 |
| *N*-stearoyl taurine | ↓ | 0.85 | 0.001 |
| *N-*oleoyl taurine | ↓ | 0.9 | 0.02 |
| *N*-arachidonoyl taurine | ↑ | 1.29 | 0.001 |
| *N*-oleoyl tryptophan | ↑↑ | 1.8 | 0.005 |
| *N-*oleoyl tyrosine | ↓ | 0.71 | 0.011 |
| *N*-arachidonoyl tyrosine | ↑↑ | 1.53 | 0.001 |
| 2-palmitoyl glycerol | ↓ | 0.67 | 0.003 |
| 2-oleoyl glycerol | ↓ | 0.85 | 0.011 |
| 2-arachidonoyl glycerol | ↑ | 1.17 | 0.042 |
| phosphoLEA | ↑ | 1.19 | 0.088 |
| PGE_2_ | ↑ | 1.48 | 0 |
| PGF_2α_ | ↑ | 1.29 | 0.001 |
| 6-ketoPGF_1α_ | ↑↑ | 1.97 | 0 |
| Sample Mass | ↓ | 0.91 | 0.07 |

Supplemental Table 108: Lipids that significantly changed in the older adult WT brainstem relative to the younger adult WT brainstem

| Brainstem | | | |
| --- | --- | --- | --- |
| Lipid | Direction of change in old WT relative to young WT | Magnitude of change in old WT relative to young WT | p |
| *N*-palmitoyl alanine | ↓ | 0.8 | 0.013 |
| *N*-stearoyl alanine | ↓ | 0.83 | 0.065 |
| *N*-arachidonoyl alanine | ↓↓ | 0.58 | 0.024 |
| *N*-palmitoyl ethanolamine | ↑↑ | 1.7 | 0 |
| *N*-stearoyl ethanolamine | ↑↑↑↑ | 3.99 | 0 |
| *N*-oleoyl ethanolamine | ↑↑ | 1.7 | 0.001 |
| *N*-palmitoyl GABA | ↓↓ | 0.64 | 0 |
| *N*-stearoyl GABA | ↓ | 0.73 | 0 |
| *N*-oleoyl GABA | ↓↓ | 0.66 | 0 |
| *N*-linoleoyl GABA | ↓↓ | 0.55 | 0 |
| *N*-arachidonoyl GABA | ↓↓ | 0.61 | 0 |
| *N*-docosahexaenoyl GABA | ↓↓ | 0.54 | 0 |
| *N*-stearoyl glycine | ↓ | 0.86 | 0.09 |
| *N*-linoleoyl glycine | ↓ | 0.88 | 0.082 |
| *N*-stearoyl leucine | ↓ | 0.82 | 0.08 |
| *N*-oleoyl methionine | ↓ | 0.71 | 0.048 |
| *N*-stearoyl phenylalanine | ↑ | 1.29 | 0.077 |
| *N*-palmitoyl serine | ↑ | 1.12 | 0 |
| *N*-stearoyl serine | ↑ | 1.09 | 0.038 |
| *N*-oleoyl serine | ↑ | 1.1 | 0.02 |
| *N*-stearoyl taurine | ↑ | 1.13 | 0.016 |
| *N-*oleoyl taurine | ↑ | 1.09 | 0.08 |
| *N-*oleoyl tyrosine | ↑ | 1.27 | 0.08 |
| *N*-docosahexaenoyl tyrosine | ↑ | 1.38 | 0.091 |
| 2-palmitoyl glycerol | ↑ | 1.37 | 0.024 |
| 2-oleoyl glycerol | ↑ | 1.28 | 0.001 |
| Oleic acid | ↓ | 0.78 | 0.048 |
| Linoleic acid | ↓ | 0.71 | 0.003 |
| Arachidonic acid | ↓ | 0.8 | 0.05 |
| phosphoLEA | ↑ | 1.29 | 0.063 |

Supplemental Table 109: Lipids that significantly changed in the older adult ABHD12 KO brainstem relative to the younger adult ABHD12 KO brainstem

| Brainstem | | | |
| --- | --- | --- | --- |
| Lipid | Direction of change in old ABHD12 KO relative to young ABHD12 KO | Magnitude of change in old ABHD12 KO relative to young ABHD12 KO | p |
| *N*-palmitoyl alanine | ↓ | 0.83 | 0.015 |
| *N*-oleoyl alanine | ↓ | 0.79 | 0.004 |
| *N*-linoleoyl alanine | ↓ | 0.67 | 0.006 |
| *N*-arachidonoyl alanine | ↓↓↓ | 0.45 | 0.02 |
| *N*-docosahexaenoyl alanine | ↓↓ | 0.6 | 0.003 |
| *N*-stearoyl ethanolamine | ↑↑↑ | 2.12 | 0.005 |
| *N*-linoleoyl ethanolamine | ↓ | 0.72 | 0.004 |
| *N*-arachidonoyl ethanolamine | ↓ | 0.85 | 0.087 |
| *N*-palmitoyl GABA | ↓↓ | 0.56 | 0 |
| *N*-stearoyl GABA | ↓↓ | 0.63 | 0 |
| *N*-oleoyl GABA | ↓↓↓ | 0.49 | 0 |
| *N*-linoleoyl GABA | ↓↓ | 0.51 | 0 |
| *N*-arachidonoyl GABA | ↓↓ | 0.59 | 0 |
| *N*-docosahexaenoyl GABA | ↓↓ | 0.54 | 0 |
| *N*-palmitoyl glycine | ↓ | 0.92 | 0.07 |
| *N*-stearoyl glycine | ↓ | 0.78 | 0.003 |
| *N*-oleoyl glycine | ↓ | 0.82 | 0.005 |
| *N*-linoleoyl glycine | ↓ | 0.84 | 0.012 |
| *N*-arachidonoyl glycine | ↓ | 0.93 | 0.055 |
| *N*-docosahexaenoyl glycine | ↓ | 0.9 | 0.038 |
| *N*-oleoyl leucine | ↓ | 0.83 | 0.083 |
| *N*-oleoyl methionine | ↓↓ | 0.62 | 0.001 |
| *N*-palmitoyl serine | ↑ | 1.13 | 0 |
| *N*-stearoyl serine | ↑ | 1.14 | 0.003 |
| *N*-linoleoyl serine | ↓ | 0.76 | 0.001 |
| *N*-palmitoyl taurine | ↑ | 1.15 | 0.002 |
| *N*-stearoyl taurine | ↑ | 1.16 | 0.006 |
| *N*-palmitoyl tryptophan | ↓↓ | 0.58 | 0.039 |
| *N*-stearoyl tryptophan | ↓ | 0.79 | 0.07 |
| *N*-oleoyl tryptophan | ↓ | 0.73 | 0.018 |
| *N*-palmitoyl tyrosine | ↓ | 0.78 | 0.042 |
| *N-*oleoyl tyrosine | ↓↓ | 0.65 | 0.001 |
| *N*-arachidonoyl tyrosine | ↓ | 0.8 | 0.01 |
| *N*-docosahexaenoyl tyrosine | ↓ | 0.73 | 0.006 |
| *N*-palmitoyl valine | ↓ | 0.8 | 0.023 |
| *N*- stearoyl valine | ↓ | 0.84 | 0.06 |
| 2-linoleoyl glycerol | ↓ | 0.74 | 0 |
| Oleic acid | ↓ | 0.72 | 0.006 |
| Linoleic acid | ↓↓ | 0.66 | 0 |
| Arachidonic acid | ↓ | 0.73 | 0 |
| 6-ketoPGF_1α_ | ↓ | 0.79 | 0.018 |
